# Sucrose versus Trehalose: Observations from Comparative Study Using Molecular Dynamics Simulations

**DOI:** 10.1021/acsomega.4c07314

**Published:** 2024-11-04

**Authors:** Inna Ermilova, Alexander Lyubartsev, Vitaly Kocherbitov

**Affiliations:** † Department of Biomedical Science, Malmö University, SE-205 06 Malmö, Sweden; ‡ Biofilms Research Center for Biointerfaces, Faculty of Health and Society, SE-205 06 Malmö, Sweden; § Department of Materials and Environmental Chemistry, Stockholm’s University, SE-114 18 Stockholm, Sweden

## Abstract

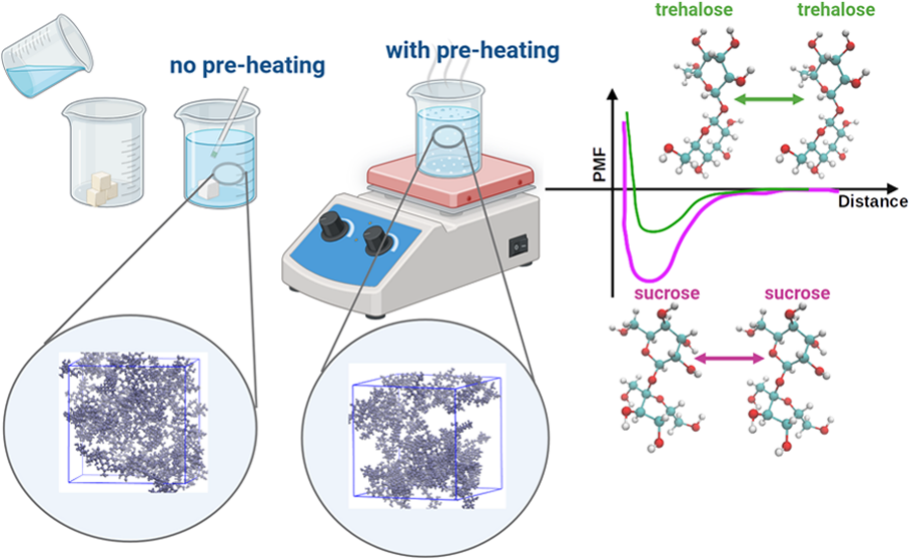

Binary mixtures of sucrose and trehalose in water were investigated using classical molecular dynamics (MD) simulations and free energy calculations. By classical MD simulations, the behavior of sugars was studied across the entire range of concentrations, from 0 to 100 wt % of water. Sugar–sugar and sugar–water affinities in diluted systems were in focus when using umbrella sampling and well-tempered metadynamics calculations. Moreover, in classical MD simulations, two approaches for system equilibration were applied: in the first, mixtures were preheated (using simulated annealing) before simulations under desired conditions, while in the second, no preliminary heating was used. It was discovered that sucrose has a stronger tendency to aggregate than trehalose, while the latter forms more hydrogen bonds with water. Below the concentration of 10 wt % of water, the number of hydrogen bonds between sugars is higher than the number of hydrogen bonds between sugars and water. The free energy calculations and hydrogen bonding analysis reveal certain dissimilarities in the hydration of oxygen-containing molecular groups. While there are noticeable differences in the hydration of various hydroxyl groups in sucrose and trehalose, all hydroxyl groups are clearly more hydrated than the ether oxygens in both sugars. Three factors contribute to the lower hydration of ether oxygens: they do not donate hydrogen bonds, they are slightly less polar than the oxygen atoms in hydroxyl groups, and they are less accessible to the solvent. Moreover, hydroxyl groups play the main role in binding water, and the geometry of trehalose is energetically preferable compared to the geometry of sucrose. Effects of preheating were demonstrated at water concentrations below 70 wt %, with more significant differences between mixtures observed at water concentrations below 40 wt %. Disaccharides bind stronger to each other and weaker with water molecules in preheated systems than in mixtures that were not preheated. The hydroxyl groups of sucrose and trehalose in preheated mixtures rotate slower than in systems that did not undergo thermal treatment. Therefore, while preheating is not necessary for liquid solutions, it is vital for the equilibration of samples in their amorphous solid state. In the experimental community, these findings are relevant for decision-making when choosing one of the disaccharides as a preservative.

## Introduction

Designing pharmaceutical formulations for various applications in the modern world requires consideration of their storage.^1,2^ Final products must be stable under conditions that have the lowest storage costs.^3−5^ Such challenges have already been faced with modern vaccines against COVID-19.^6−8^ Various preservatives are used in formulations to maintain their stability and efficacy for a desired time interval in a selected environment.^9−12^

**Figure 1:**
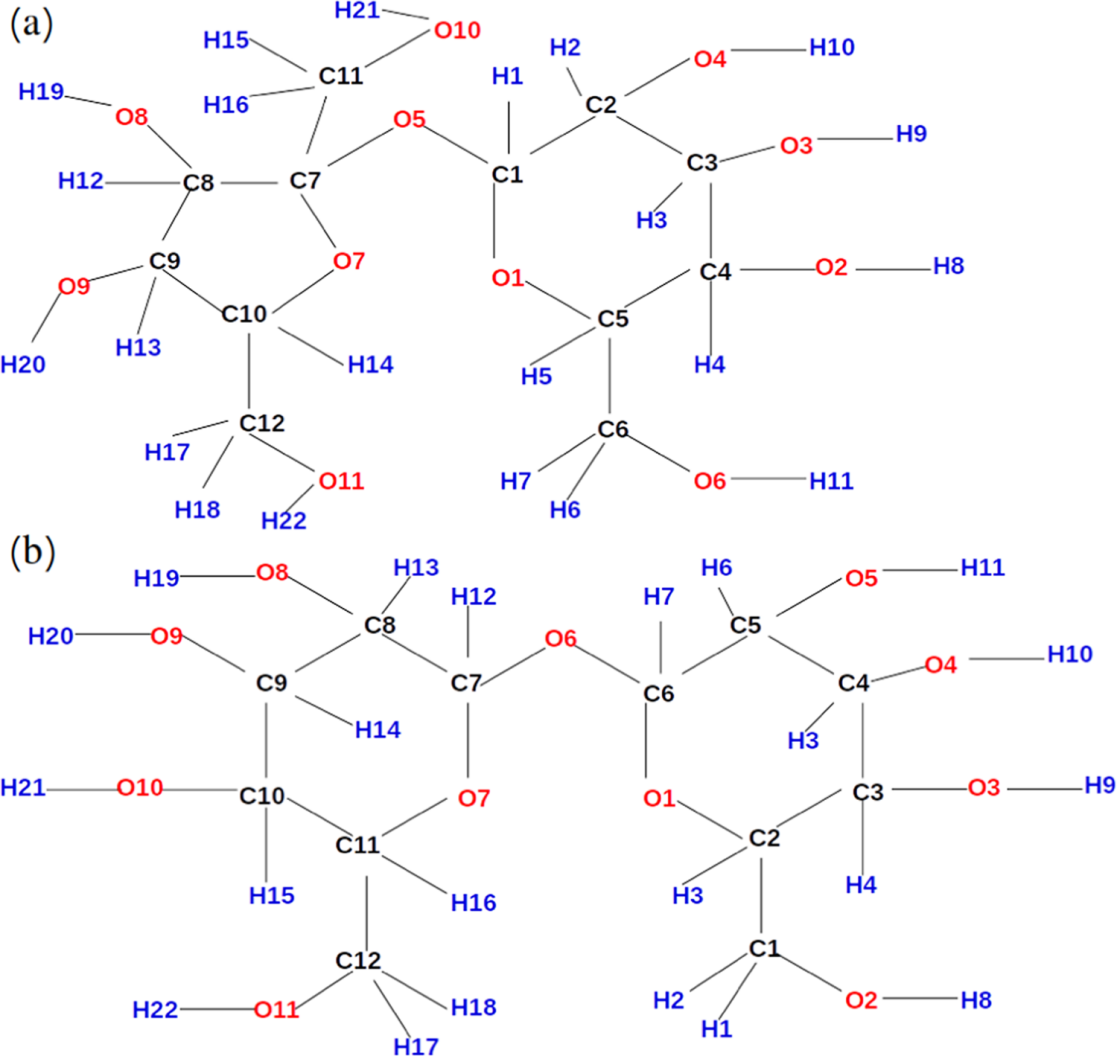
Sucrose (a) and trehalose (b). Labels on atoms will be used further in this work for the explanation of interactions and should not be confused with nomenclature. Note that these labels are representing atom names in itp-files for the published model by Ahlgren et al.^13^

In this work, preservatives used in biological applications are of interest, particularly sucrose and trehalose (see Figure 1). A large number of experimental and computational studies have been conducted regarding these sugars.^12−19^ Often, the main focus was to determine which of them is more effective for stabilizing large molecules, such as proteins,^20^ nucleic acids, and even lipid membranes.^13,18,21,22^ Additionally, these sugars have been investigated in solutions with water.^14,23,24^

In 1997, Liu et al.^23^ studied the behavior of single molecules of sucrose and trehalose in mixtures with water using MD simulations. In their work, they did not observe any differences between these disaccharides. Nevertheless, this could be due to the small system sizes (1 molecule of sugar in 512 molecules of water) and the short simulation times (130 ps).

Later, similar computational experiments were conducted by Lee et al.,^25^ but using a different force field and with one sugar molecule surrounded by 300 water molecules. It was discovered that the hydrogen bonds between trehalose and water were more stable than those between water molecules. However, the authors did not observe any other differences between the investigated sugars.

A. Simperler et al.^26^ studied the glass transition temperatures (*T*_g_) of glucose, sucrose, and trehalose by MD simulations. They found that molecular simulations can accurately reproduce trends in *T*_g_, with trehalose having the highest *T*_g_ and glucose the lowest *T*_g_. However, their findings show that MD simulations overestimate values of *T*_g_ by 12–13 K.

Olsson et al.^27^ investigated mixtures of sucrose and trehalose (with 33 wt % sugar) using neutron and X-ray diffraction experiments, which were interpreted by empirical potential structure refinement (EPSR) modeling.^28−30^ In their work, they discovered that sucrose was slightly less hydrated than trehalose. The latter could also perturb the structure of water to a greater extent than sucrose.

Affouard et al.^24^ have performed quasi-elastic neutron scattering, neutron spin echo, inelastic neutron scattering and MD studies for sucrose and trehalose for the sugar mass ratios in the range of 0–66% and for the time duration of 0.2–2 ns. It was also discovered that trehalose has a better ability than other sucrose to alter the structure of water and strongly interact with it. They claimed that trehalose could form a glassy shell which would be a reason behind its bioprotective properties.

Olgenblum et al.^31^ studied aqueous mixtures of trehalose and their glass transition using advanced molecular simulation methods. They also considered dehydrated systems in their work. They concluded that the glass transition of investigated mixtures was related to the transition from weakly to strongly correlated hydrogen bonds.

In a recent study by Kocherbitov et al.,^32^ researchers explored mixtures of trehalose and water in the amorphous solid state using quantum chemical and MD simulations. They discovered that the hydrogen bonding energy between trehalose and water depended on the geometries and local environments of the hydrogen bonds. Moreover, the authors discovered that the observed exothermic effect in calorimetric experiments was caused by different responses of liquid and glassy matrices to perturbations related to the addition or removal of water molecules.

All the mentioned works have produced interesting and valuable results, but there has not been a computational study covering the entire range of concentrations, i.e., the interval from 0 to 100 wt % of sugars. The main reason is the behavior of sugars at low water contents in systems considered almost dry,^33−35^ which is challenging to investigate by available computational methods. The dynamics of dehydrated systems can be very slow, even on the laboratory time scale, and in terms of computational time, it might be impossible to achieve stability during production runs with atomistic simulations, which are on the order of nanoseconds or microseconds.^36,37^ One way to reach equilibrium is to heat the systems to a temperature above their *T*_g_ and then simulate the resulting system under the desired conditions. Nevertheless, to validate or challenge results from such computational studies, they need to be compared with systems modeled at the required temperature and pressure in a “traditional” manner, i.e., by running a long simulation where only the part corresponding to equilibrium is considered for analysis.

Therefore, in this project, two main approaches were applied to investigate and compare mixtures across the entire concentration range. The first approach involves simulating randomly placed molecules. Here, the goal is to understand if any equilibrium properties can be reached^38,39^ and to determine a suitable approach for simulating more dehydrated systems.

The second approach is to use simulated annealing.^40−42^ First, the system would be equilibrated at a temperature above its *T*_g_.^43,44^ Then, the system would be slowly cooled to the desired temperature using the simulated annealing algorithm during a relatively long simulation time. Next, the cooled mixture would be simulated for the same duration as the system without annealing (a regular isothermal–isobaric run) to ensure the same statistics as in the first approach.

The main reason for employing the second approach is to obtain properly equilibrated dehydrated systems, which can be achieved by first performing isothermal–isobaric simulations at a temperature above *T*_g_ and then slowly cooling.^36,37,45^ As mentioned earlier, the dynamics of dehydrated systems are very slow, even on experimental time scales, which are impossible to reach with atomistic MD simulations for temperatures below *T*_g_ of the studied material. Therefore, the second approach can provide more reliable results for these dehydrated mixtures.

Another reason for using the second approach is to compare results from both methods and determine if there are any differences and how significant they are between the behaviors of the simulated mixtures. Additionally, identifying concentration intervals where discrepancies are observed can help in interpreting and understanding experimental results for similar samples.

Additionally, free energy calculations^46−50^ are used to complement classical MD simulations and explore the thermodynamics behind sugar aggregation and hydration, to understand if such phenomena could occur spontaneously. Another goal is to discover discrepancies in their behavior in very diluted systems.

For instance, sugar–sugar interactions are studied using umbrella sampling^50^ and well-tempered metadynamics,^46−48,51^ while sugar–water interactions for each group containing oxygen atoms are investigated using well-tempered metadynamics.

Combining all these methods will help to provide more accurate information about behavioral similarities and differences between sucrose and trehalose. Understanding the features of disaccharides that affect their behavior can inform decisions about whether to use sucrose or trehalose in a formulation.

## Methods and Models

Models for sucrose and trehalose were taken from earlier published work by Ahlgren et al.,^13^ as their use resulted in good agreement between simulated and experimental data. This models were developed using the approach from General Amber force field (GAFF).^73^ The water model, selected for the current calculations, was SPCE.^52^

The simulated systems had compositions listed in Table 1. Each system had an equal mass except for the system with 3700 water molecules. The goal of simulating 2 sugars with either 3700 or 3341 water molecules was to understand if a very small change in concentration could affect the behavior of the two sugar molecules.

**Table 1:** Compositions of Systems

number of sugar molecules	number of water molecules	mass % of water
2	3700	99.95
1	3356	99.44
2	3341	99
9	3206	95
18	3038	90
36	2700	80
53	2363	70
71	2025	60
89	1688	50
107	1350	40
124	1013	30
142	675	20
151	506	15
160	338	10
169	169	5
176	34	1
178	0	0

### Systems without Preheating

First, sugar molecules were placed randomly in each box of size 5 nm in such a way that there was a distance of at least 0.3 nm between atoms of adjacent sugar molecules. Then, water molecules were added to the boxes with sugar randomly.

Once the boxes were prepared, they were energy minimized to eliminate overlaps between molecules using the steepest descent algorithm. After that, each box was equilibrated for 10 ns and simulated for an additional 40 ns, resulting in the production run.

The software GROMACS-2022^53^ was selected for simulations. Equilibration and production runs were performed in the NPT ensemble,^54^ where the temperature was controlled by the Velocity Rescale^55^ thermostat and the C-rescale barostat^56^ was used for pressure control. The coupling scheme was isotropic. The pressure was set to 1 atm and the temperature to 298 K. The coupling constant for temperature was 0.5 ps, and for pressure, it was 10 ps. The time step was set to 2 fs. The integrator used was Leap-Frog^57^ with a cutoff scheme of Verlet.^58^ Trajectory output was recorded every 500 frames, corresponding to 1 ps. The compressibility was 4.5 × 10^–5^ bar^–1^. The algorithm for constraining bonds was LINCS^59^ with 12 iterations. The cutoff distances for Coulomb, Lennard-Jones, and short-range neighbor interactions were set to 0.9 nm. Long-range electrostatics were handled using the Particle Mesh Ewald algorithm^60^ with a Fourier spacing of 1.2 Å.

### Systems with Preheating

This set of systems was created in a different way, but the original starting configurations were taken from simulations without preheating after the first energy minimization.

First, every system was equilibrated for 30 ns at a temperature of 450 K with the pressure set to 1 atm. All other settings and algorithms were the same as in the simulations without preheating. The trajectory output was recorded every 4 ps (every 2000 frames). The reason for the less frequent output is that, from this step onward, the trajectory was not useful for this particular project.

Then, after heating, the final step was cooling to 298 K. Cooling was done as follows: to cool the system gently, simulated annealing was used.^40−42^ During the first 20 ns, the temperature decreased from 450 to 298 K. Then, for 10 ns, the temperature was maintained at 298 K. All other settings and algorithms were the same as in the previous step. The trajectory output was recorded every 2 ps (every 1000 frames, in case this would be relevant for further analysis).

For the final step (i.e., the final equilibration and production run), the input was taken in the form of final frames from the previous step. The procedure for equilibration and running the production run was exactly the same as in the case without any preheating, including all algorithms and settings.

### Umbrella Sampling and Well-Tempered Metadynamics for Sugar–Sugar Interactions

Due to the structural variety, studying sugar–sugar interactions using free energy simulations presents challenges when choosing the distance between two molecules as a collective variable. To obtain relatively accurate results, a combination of umbrella sampling and well-tempered metadynamics was used, where only one variable, the distance between the centers of mass of the sugars, was considered. For both types of calculations, the boxes containing two disaccharides and 3341 water molecules were taken from the final frames of classical MD simulations in which the systems did not undergo any preheating.

#### Settings for Umbrella Sampling

For umbrella sampling simulations, 22 windows were created along the reaction coordinate, which was the distance between the centers of mass of the sugar molecules. The pulling was performed with 5 different force constants at a rate of 0.001 nm per 1 ps. The force constants were as follows: 45, 35, 55, 25, and 15 kJ/(mol·nm^2^). Simulations were conducted in the NPT ensemble,^54^ where the pressure was controlled by the C-rescale barostat^56^ and the temperature by the Velocity Rescale thermostat.^55^ The pressure was set to 1 atm with a pressure coupling constant of 10 ps, while the temperature was set to 298 K with a coupling constant of 0.5 ps. The pressure coupling scheme was isotropic. The LINCS algorithm^59^ was used for constraining bonds with 12 iterations. The integrator for Newtonian equations was Leap-Frog^57^ with a time step of 2 fs and the cutoff scheme was Verlet.^58^ The cutoff distance for the short-range neighbor list was 0.9 nm. The compressibility was 4.5 × 10^–5^ bar^–1^. Each window was simulated for 400 ps. The MD engine utilized for simulations was GROMACS-2022.^53^ In the end, an average profile was computed over 5 umbrella sampling simulations to obtain better statistics.

#### Settings for Well-Tempered Metadynamics

Well-tempered metadynamics simulations were performed using PLUMED-2.9.0^61^ software together with GROMACS-2022.^53^ As in the case of umbrella sampling, the collective variable was the distance between the centers of mass of the sugar molecules. Since sugars are quite small molecules, the chosen bias factor was set to 50. The temperature was 298 K. The grid spacing was 0.05 nm, and sigma was equal to 0.05 nm. The Gaussian height was 1.2 kJ/mol, and Gaussian functions were deposited every 500 steps. The grid was saved every 100 000 steps (the parameter GRID_WSTRIDE). Both sugar molecules were kept as whole entities because the location of their centers of mass was of interest. The total length of each simulation was 200 ns. The settings for the MD engine were the same as for classical MD simulations, which were executed without preheating.

### Well-Tempered Metadynamics for Sugar–Water Interactions

For simulations of interactions between water molecules and hydroxyl groups or ether oxygens of sugars, the same parameters were used for PLUMED-2.9.0.^61^ The collective variables were the distances between the centers of mass of water molecules and the centers of mass of hydroxyl groups/ether oxygens. For trehalose, calculations for symmetric groups were done separately, and the average profiles for them were taken as the final ones. Each simulation was 200 ns long. The MD engine used was GROMACS-2022^53^ with the same settings as for well-tempered metadynamics with 2 sugar molecules.

## Results and Discussion

### Hydrogen Bonds

The number of hydrogen bonds can provide information about strong associations of molecules.^62−64^ Since simulations were performed for concentrations in the range 0–100% of water, it is possible to answer the question about the effects of hydration on interactions between sugars.

**Figure 2:**
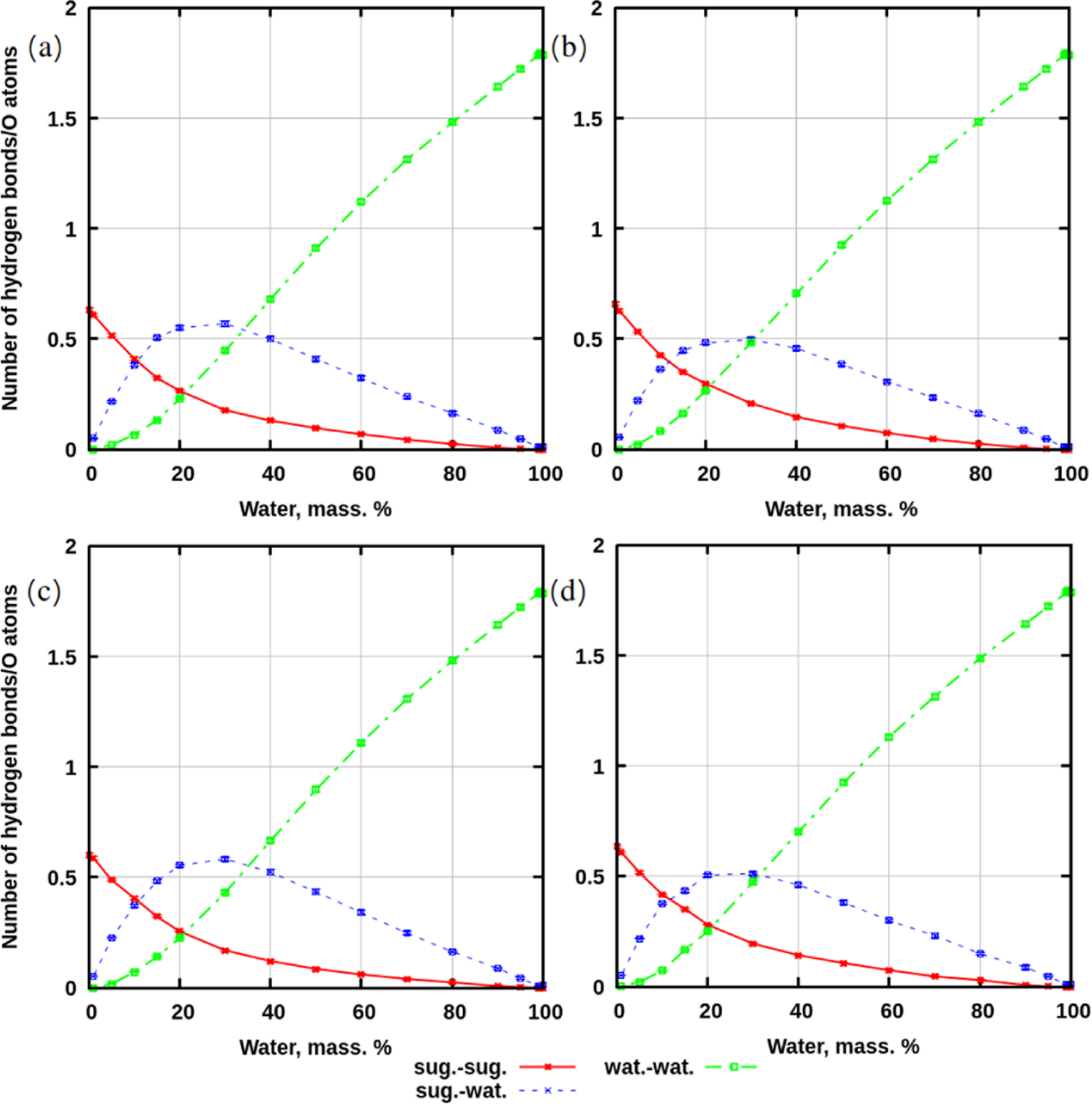
Number of hydrogen bonds, computed per number of oxygens in a system. (a) Systems with sucrose without preheating. (b) Systems with sucrose with preheating. (c) Systems with trehalose without preheating. (d) Systems with trehalose with preheating. Abbreviations in legends: “sug.”—sugar, “wat.”—water. The data containing values with standard deviations can be seen in Tables S1–S3 in electronic Supporting Information (SI).

Figure 2 demonstrates the number of hydrogen bonds per oxygen atom in each system, depending on the concentration of water in every mixture. The reason for considering the number per oxygen atom is that it is easy to compare values among all systems which contain different numbers of molecules. From the data shown in the figure, it follows that there are differences between simulations that were preheated using annealing and those that were not. In simulations (for both sucrose and trehalose) without preheating, the number of hydrogen bonds between water molecules is equal to the number of hydrogen bonds between sugar and water at a concentration of around 35% of water, while in the systems with preheating, this phenomenon can be observed at 30% of water. Additionally, the concentration of 30% of water appears to be the point at which the number of hydrogen bonds between sugar and water reaches its maximum. At 20% of water, the number of hydrogen bonds between water molecules is equal to the number of hydrogen bonds between sugars. Below this concentration and down to 0% of water, a rapid decline in sugar–water hydrogen bonds and an increase in sugar–sugar hydrogen bonds are observed. These trends are consistent across all simulated systems, regardless of the thermal process to which they were exposed.

What do these trends mean? The curve describing the number of sugar–water hydrogen bonds shows a descending trend between 30 and 100 wt % of water. However, this is a result of normalization to the total number of oxygens in the system, which increases with increasing water content. It is expected that the amount of free water in the systems (which was not explicitly computed) is increasing with the amount of water. One would expect a certain correlation with water–water hydrogen bonds, which increase with growing water content (Figure 2).

Additionally, the concentration when the number of hydrogen bonds between water molecules equals the number of hydrogen bonds between water and disaccharides is not the same for systems with and without preheating, which demonstrates the ability of annealing to remove the water from clusters of sugars (increase the amount of free water). This effect is noticeable in more dehydrated mixtures with more than 65 wt % of disaccharide.

**Figure 3:**
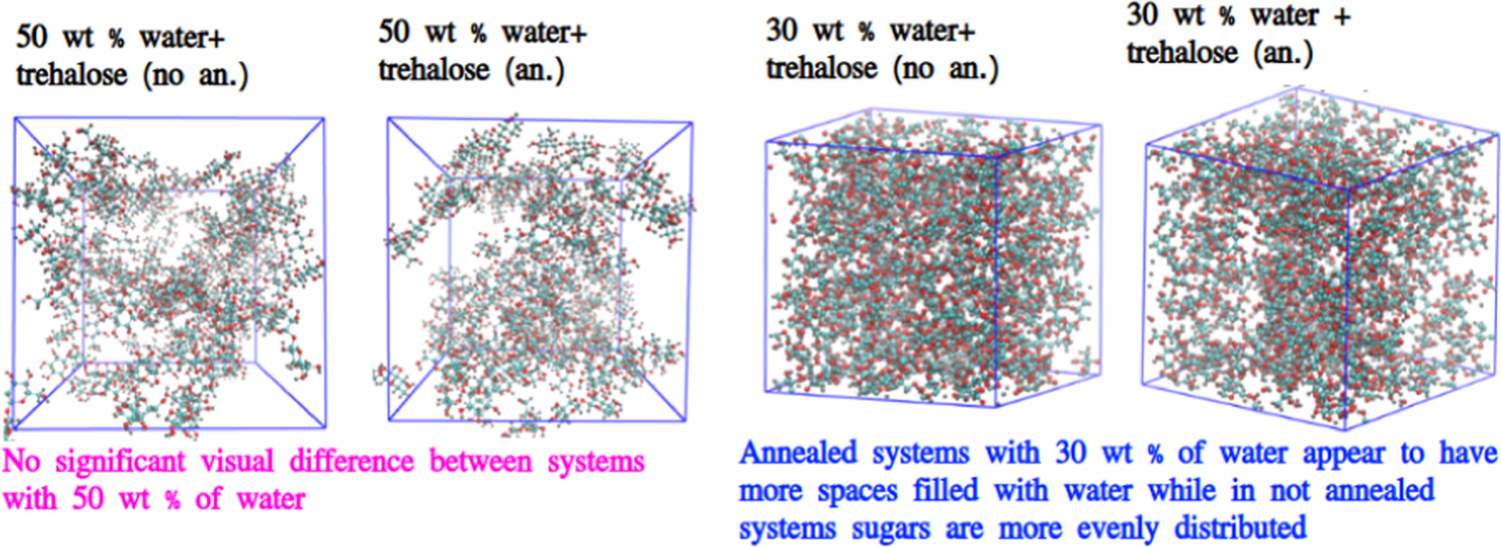
Screenshots for some systems with trehalose (water molecules were omitted for the clarity). Colors: cyan—carbon, red—oxygen, gray—hydrogen. Abbreviations in legends: “no an.”—no preheating, “an.”—with preheating.

For an illustration of such conditions, one should refer to Figure 3, where screenshots are shown for some systems with trehalose (systems with sucrose looked similar). In more diluted systems, more free water can be observed (empty spaces in the figures) than in mixtures with less water. However, for simulations with 50 wt % of water, there is no visual difference between annealed and nonannealed mixtures. The thermal treatment in more dehydrated systems induced the aggregation of sugar and released more free water (systems with 30 wt % of water), compared to the mixture without preheating.

**Figure 4:**
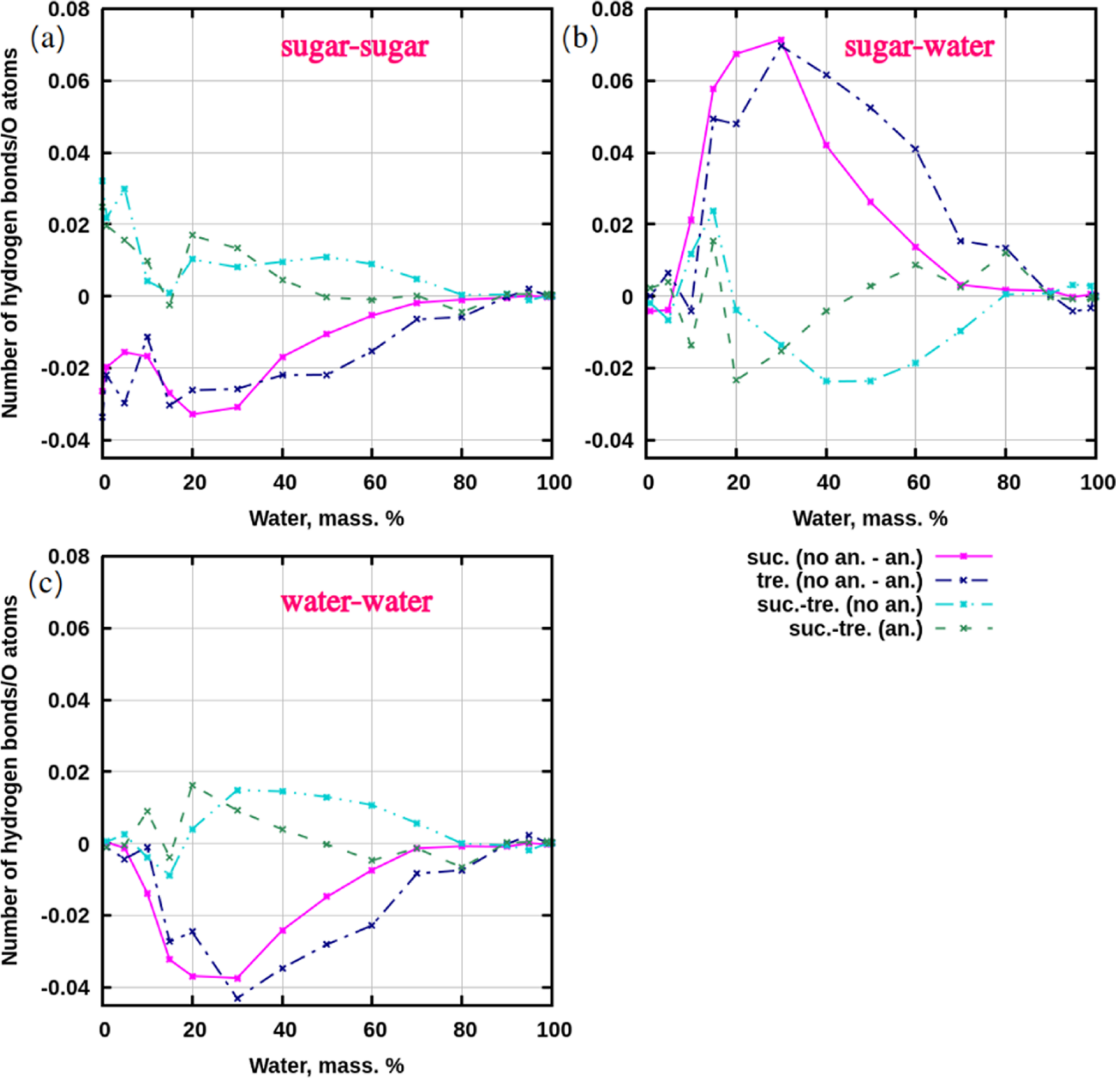
Difference profiles for average numbers of hydrogen bonds, computed per number of oxygens in a system. (a) Sugar–sugar. (b) Sugar–water. (c) Water–water. Abbreviations in legends: “suc.”—sucrose, “tre.”—trehalose, “(no an.-an.)”—numbers of bonds were subtracted for the same kind of sugar: bonds_no pre–heating_ – bonds_pre–heating_. “suc.-tre.(no an.)”—from the number of hydrogen bonds in systems with sucrose was subtracted the number hydrogen bonds in systems with trehalose and annealing was not used in the system preparation. “suc.-tre.(an.)”—from the number of hydrogen bonds in systems with sucrose was subtracted the number hydrogen bonds in systems with trehalose and annealing was used in the system preparation. The full data with standard deviations is presented in Tables S1–S3 in SI.

In order to identify discrepancies between preheated systems and simulations where annealing was not used, difference profiles were computed for the systems. Figure 4a demonstrates profiles for hydrogen bonds between sugars. There are fewer hydrogen bonds between sugars in simulations without preheating, which is indicated by negative values for both sucrose and trehalose. A comparison of differences in hydrogen bonds between sucrose and trehalose shows that there are more hydrogen bonds between sucrose molecules than between trehalose molecules (plotted values are positive).

Trends for differences in hydrogen bonds between sugars and water are distinct compared to sugar–sugar interactions. Figure 4b shows that there are more hydrogen bonds between sugars and water in systems that were not subjected to preheating (the values are positive). Considering the difference profiles for the same thermal treatment, it can be concluded that trehalose has more hydrogen bonds with water than sucrose (the curves go below zero). This statement could probably be applied to solutions with single molecules of sugars (infinite dilution condition), because, despite the standard deviation of about 1.6 hydrogen bonds, the average value for hydrogen bonds between trehalose and water was 19, while sucrose and water had 18. As in a work by Liu et al.,^23^ one cannot conclude about which disaccharide binds more water in an infinite dilution condition due to large value of a standard deviation. To make a more precise statement for this specific condition, one must consider the thermodynamics of the binding process, which is done in a section for free energy calculations.

Water–water hydrogen bonds demonstrate trends similar to those observed between sugars (Figure 4c). There are more hydrogen bonds between water molecules in simulations that were preheated compared to those that were not. For the same thermal treatment, there are more hydrogen bonds between water molecules in simulations with sucrose than in systems with trehalose.

Lastly, considering the behavior around concentrations, it can be concluded that there are clear trends for the range 100–30% of water, which change for the interval 0–30% and even become less stable, i.e., curves show fluctuations and have more intersections. These trends can be shortly described as the following relations for the range 100–30 wt % of water (here *N*_H–bonds_ is the number of hydrogen bonds):

- *N*_H–bonds_(sucrose–sucrose, no an.) > *N*_H–bonds_(trehalose–trehalose, no an.);
- *N*_H–bonds_(disaccharide–disaccharide, no an..) < *N*_H–bonds_(disaccharide–disaccharide, an.);
- *N*_H–bonds_(sucrose-water, no an.) < *N*_H–bonds_(trehalose-water, no an.);
- *N*_H–bonds_(water–water, no an.) < *N*_H–bonds_(water–water, an.).

**Figure 5:**
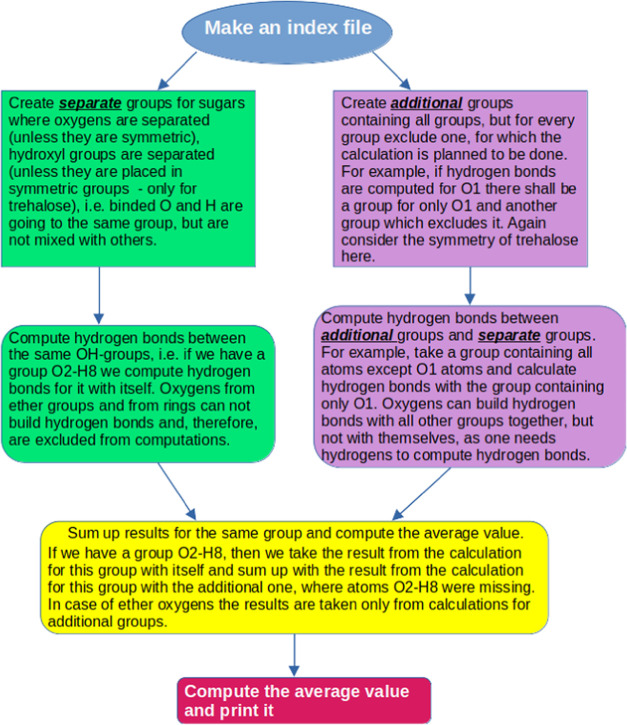
Algorithm for calculation of hydrogen bonds between sugars.

Nevertheless, knowledge about the total number of hydrogen bonds does not indicate which groups (OH-groups, ether oxygens, or oxygens from glucose/fructose rings) were involved in their formation. Such information was obtained after calculating the numbers for each group. Due to the molecular symmetry of trehalose, some groups were combined for calculations (values per group were divided by 2), while groups for sucrose remained the same (the algorithm for calculating hydrogen bonds is shown in Figure 5).

**Figure 6:**
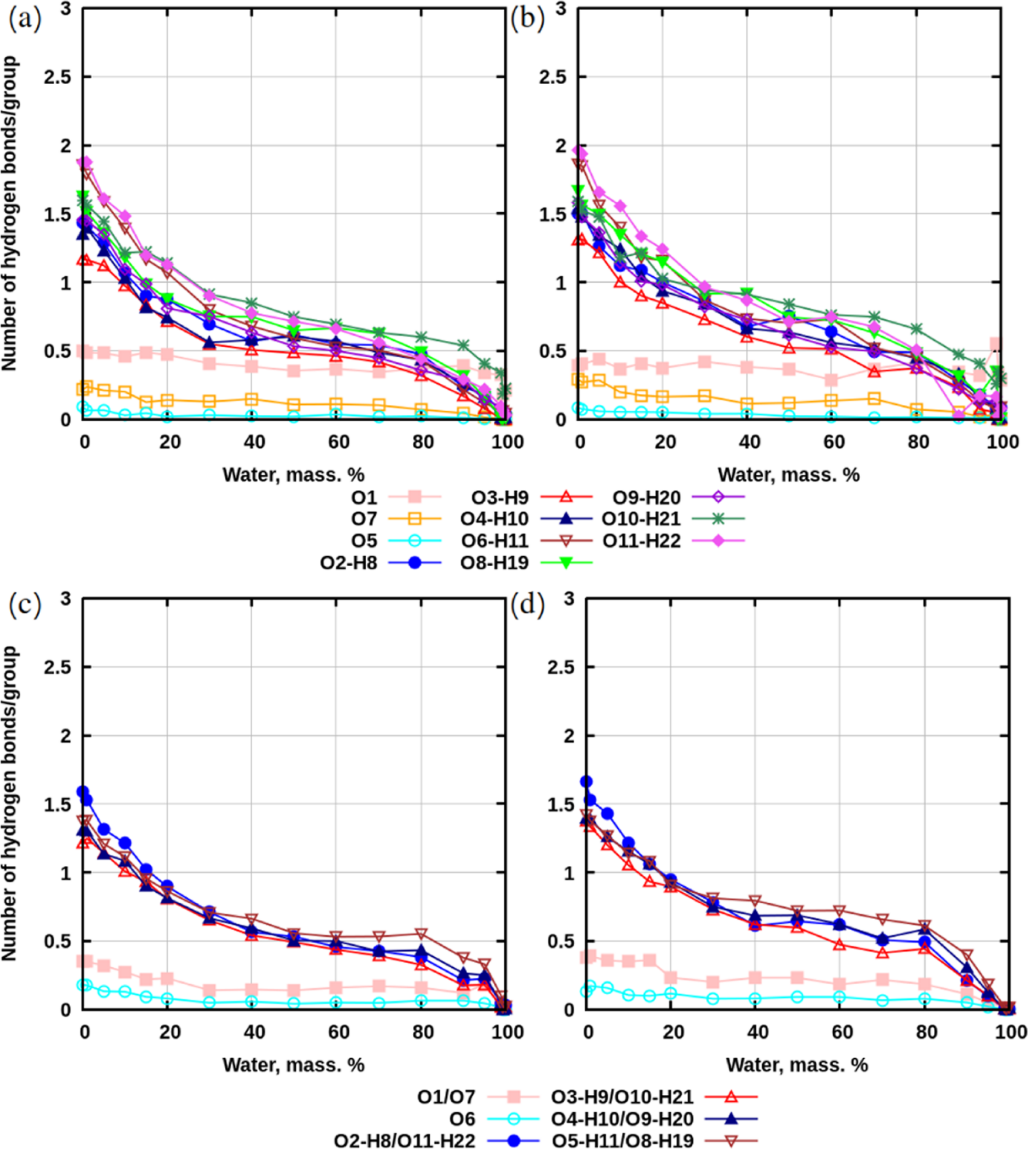
Hydrogen bonds between selected groups of atoms and all other groups in sugars, computed per 1 O- or OH-group of atoms (see Figure 1). (a) Systems with sucrose without preheating. (b) Systems with sucrose with preheating. (c) Systems with trehalose without preheating. (d) Systems with trehalose with preheating. The data with standard deviations is presented in Tables S4–S9 in SI.

Figure 6 demonstrates hydrogen bonds between sugars. Since the algorithm in GROMACS software^53^ cannot handle overlaps within the same group chosen for the calculation, the values were computed in the following way.

Ether oxygen atoms appear to be involved in a fewer number of hydrogen bonds compared to hydroxyl groups for both sucrose and trehalose. This might be explained by structural reasons: OH-groups are located at the surface of molecules, making them more accessible compared to the locations of ether oxygen atoms. The nonzero values for hydrogen bonds in simulations with single sugars indicate that they are formed due to intramolecular interactions. The general trend shows that the number of hydrogen bonds for sugar–sugar interactions increases, but this increase is slower for concentrations above 30% of water and much faster below it.

**Figure 7:**
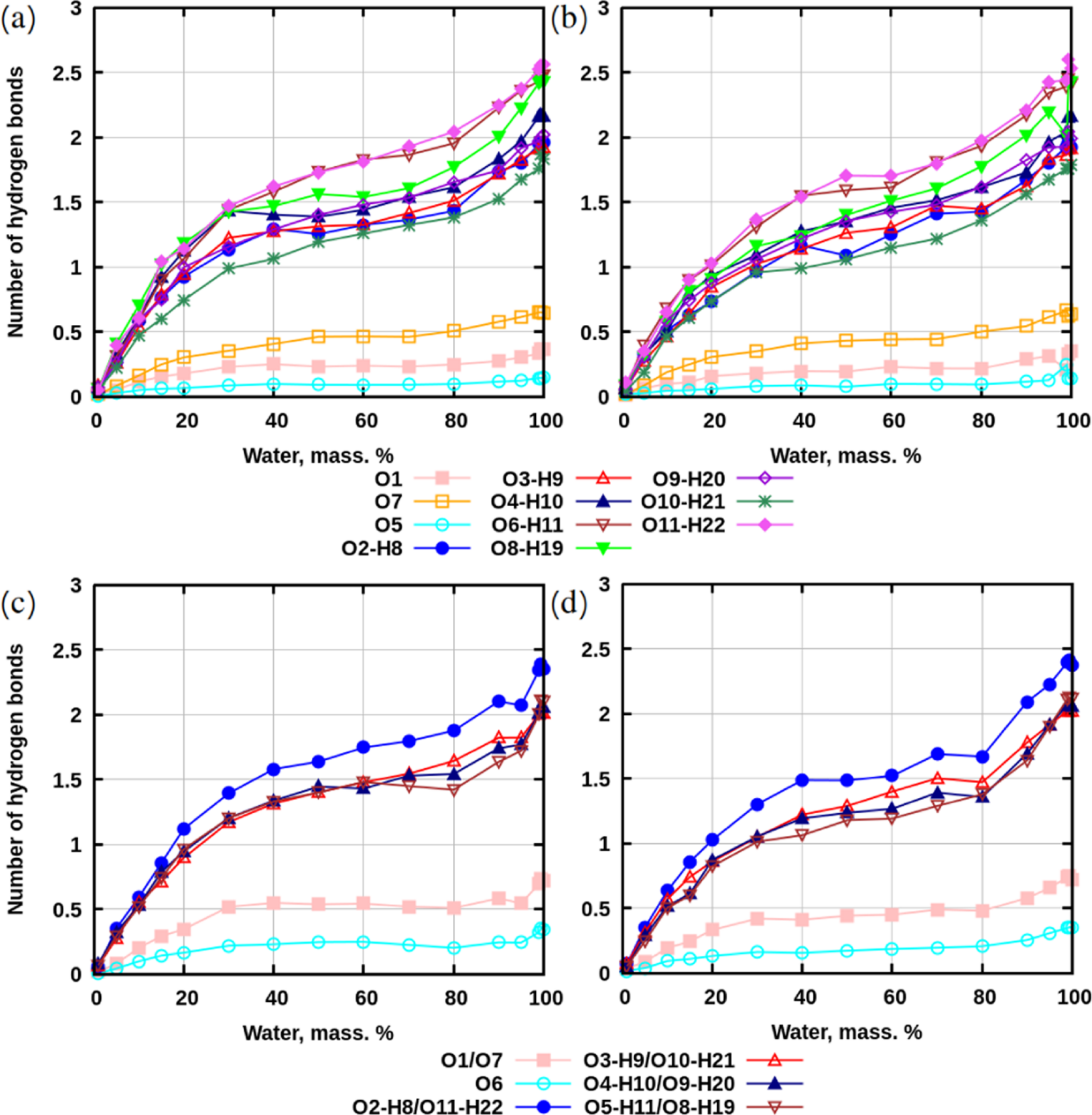
Hydrogen bonds between selected groups of atoms in sugars and water, computed per 1 O- or OH-group of atoms. (a) Systems with sucrose without preheating. (b) Systems with sucrose with preheating. (c) Systems with trehalose without preheating. (d) Systems with trehalose with preheating. The data with standard deviations is presented in Tables S10–S15 in SI.

Sugar–water interactions exhibit a different trend (Figure 7): here, the number of hydrogen bonds between water and sugars decreases, faster for concentrations below 30% and slower above it. As in the case of sugar–sugar interactions, hydroxyl groups have a higher number of hydrogen bonds with water than ether oxygens. The maximum value of hydrogen bonds per OH-group is about 2.5 at the highest concentration of water. This number is higher than the maximum number of hydrogen bonds between sugars.

**Figure 8:**
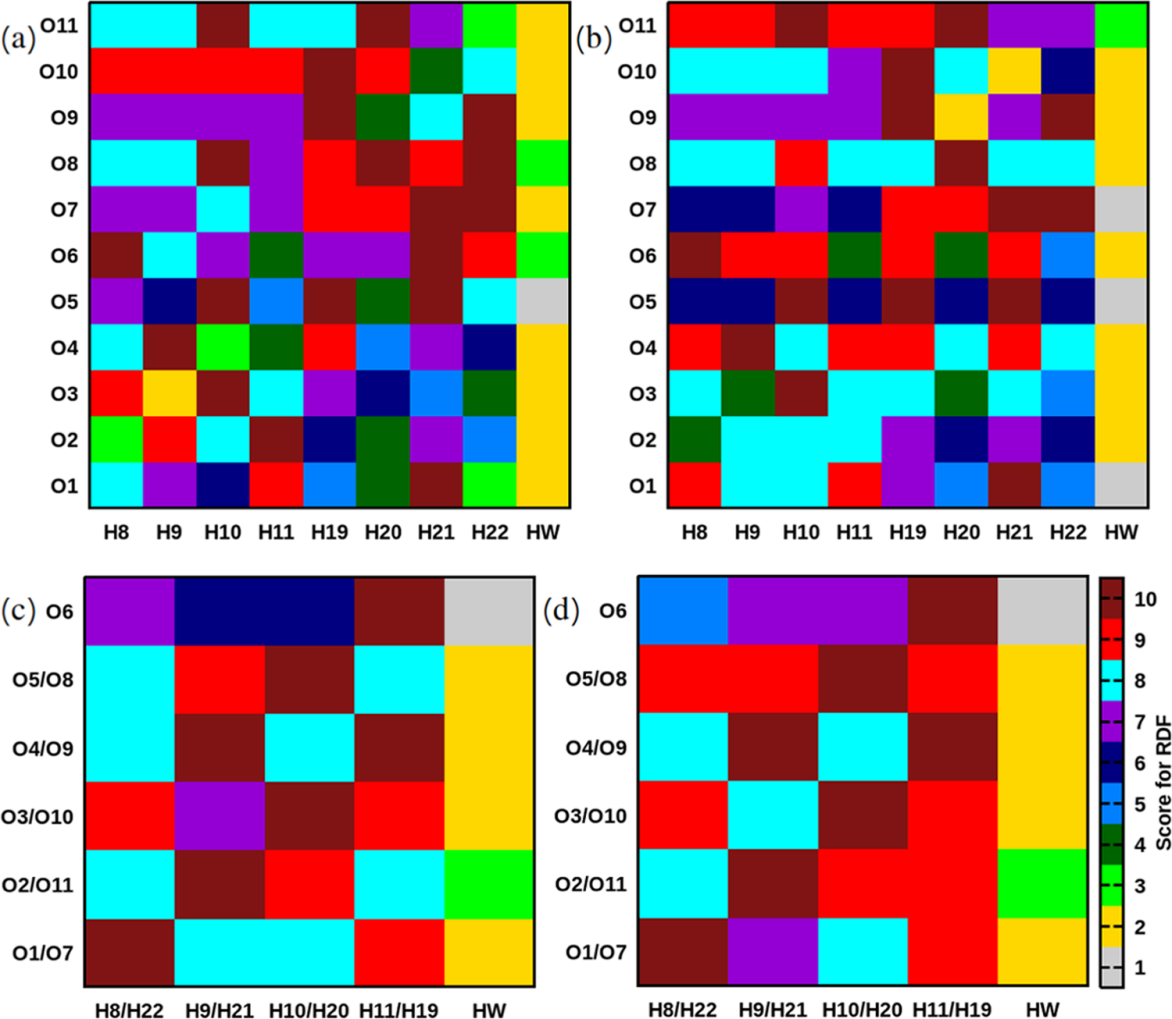
Generalized RDFs (or a contact map) for sugar–sugar (oxygen—OH-hydrogen) and sugar–water (oxygen—hydrogen of water) interactions. (a) Systems with sucrose without preheating. (b) Systems with sucrose with preheating. (c) Systems with trehalose without preheating. (d) Systems with trehalose with preheating. Images were created out of visual inspection of data for all concentrations, presented in SI in Figures S1–S37.

The number of hydrogen bonds provides an idea of how many bonds there are in total per group, but it does not indicate which specific groups in disaccharides bind to each other, leaving the structural information uncovered. The goal here is to draw an approximate contact map for sugars, which is a more general description of the strength of sugar–sugar associations compared to associations with water molecules. This information was obtained from calculations of radial distribution functions (RDFs or *g*(*r*)), as there is no specific contact map routine in software for molecules other than proteins. Figure 8 for oxygen atoms gives a generalized picture of which hydrogens they are associated with. In this figure, scores are given from 1 to 10, where 1 is assigned to atoms with the smallest values of *g*(*r*) (around 1), while 10 is assigned if the value of *g*(*r*) is several orders of magnitude higher. The scores were assigned based on visual analysis of RDFs presented in the SI (Figures S2–S37). At the same time, a relatively high value of *g*(*r*) does not necessarily mean that there is a hydrogen bond, because atoms might be found within a certain radius due to steric orientations. The reason for this visual analysis is that there are 17 RDFs for each group, which requires a general conclusion without displaying all images at once. Therefore, for a precise value for every concentration, we refer back to the figures in the SI.

Figure 8a,b demonstrates RDFs for sucrose. Ether oxygen atoms O1, O5, and O7, which are least involved in hydrogen bonding with both water and other groups of sucrose, have high scores for RDFs with hydrogens from hydroxyl groups in sucrose and lower scores with hydrogens of water. In the system with preheating, these oxygen atoms have the lowest scores with water.

RDFs for trehalose are presented in Figure 8c,d. Ether oxygens have the smallest scores with water but higher scores with hydrogens from hydroxyl groups of trehalose. The differences between systems with and without annealing are rather small.

Consequently, from these results, in more diluted systems, disaccharides are involved in a greater number of hydrogen bonds with water than with themselves. However, sugar–sugar associations are stronger than sugar–water associations.

### Dihedrals

For complementing the structural information, dihedrals were calculated. The main interest here is to uncover if there are structural differences between solutions that were preheated using simulated annealing and those that were not, since the force field was not originally developed for the temperature of 450 K.

Figure S39 in SI presents distributions of dihedrals between glucose and fructose rings of sucrose. The expected values are the same, and some insignificant differences between preheated and not preheated systems are observed for concentrations of 20–30 wt % of water and 95–100 wt %. Similar conclusions can be made for all other dihedrals in sucrose (Figures S40–S43).

In Figure S44, dihedrals in trehalose between glucose rings can be observed. As in the case of sucrose, there is an insignificant difference in distributions for concentrations of 20–30 wt % of water. Distributions of all other dihedrals do not contain any discrepancies between preheated and not preheated systems (Figures S45–S46 in SI).

These findings help us to conclude that using simulated annealing to preheat solutions up to 450 K and then cooling them to 298 K did not dramatically change the distribution of dihedrals, which also means that we have the same distributions of conformations. This is a positive observation, as the structures were not altered due to thermal treatments.

### Dynamics of OH Groups of Disaccharides

Dynamics of OH-groups is a good complement to the structural data when comparing two approaches for modeling mixtures with sucrose and trehalose. Hydroxyl groups are known to move quickly, which makes their motion convenient for investigations on shorter time-scales in the amorphous solid state.

For the purpose of studying the dynamics of OH-groups, correlation times were computed from correlation functions for each group with an exponential fit, where the groups were written as vectors. Correlation time is defined here as the average time needed for a vector to rotate by 1 rad.

**Figure 9:**
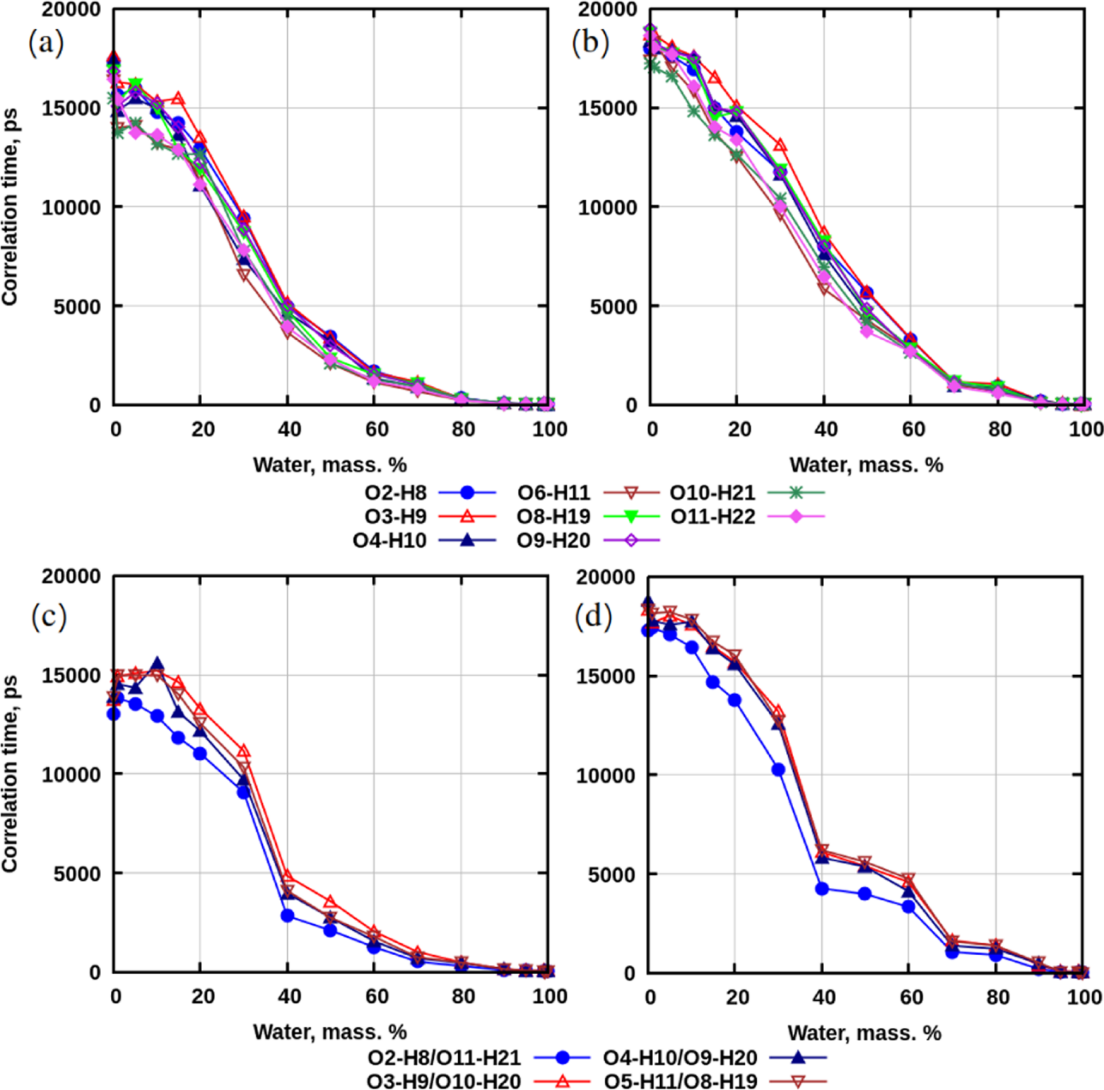
Correlation times for OH-groups of disaccharides. (a) Systems with sucrose without preheating. (b) Systems with sucrose with preheating. (c) Systems with trehalose without preheating. (d) Systems with trehalose with preheating.

Figure 9 demonstrates correlation times for all mixtures with disaccharides. For concentrations of 70–100 wt % of water, the behavior is similar for both disaccharides regardless of the use of annealing: correlation times grow slowly from picoseconds to nanoseconds. For concentrations below 70 wt % of water, discrepancies are more pronounced between sucrose and trehalose, as well as between simulations with and without preheating.

For concentrations of 40–70 wt % of water, in preheated systems with sucrose (Figure 9b), correlation times increase more rapidly to higher values with decreasing amounts of water than in nonpre-heated mixtures (Figure 9a). In the same range of concentrations for trehalose, the same phenomenon can be observed (Figures 9c,d). However, for preheated systems with trehalose, the largest rise in correlation time occurs at 60 wt % of water, and in the range of 40–60 wt %, the time grows more slowly.

The interval of 20–40 wt % of water appears to be when correlation time increases more significantly for all systems: from ≈5000 to ≈12 000 ps for mixtures that were not preheated, and from ≈5000 to ≈15 000 ps for simulations where annealing was used. The largest rise in correlation time is observed from 40 to 30 wt % of water (in the case of trehalose, the time increases by 7000 ps at 30 wt % of water).

For more dehydrated mixtures (interval 0–20 wt % of water), correlation time grows much slower to ≈15 000 ps in simulations without preheating, while in preheated systems, it rises faster to ≈18 500 ps.

Finalizing this discussion about the dynamics of OH-groups, several remarks can be made.

First, in systems with preheating at concentrations below 70 wt % of water, the dynamics of these groups is slower compared to mixtures that were not preheated. These observations can be explained by hydrogen bonding, which was discussed earlier. There were more sugar–sugar hydrogen bonds observed in preheated systems than in those that were not preheated.

Second, correlation times for more dehydrated systems with sucrose are higher than those for trehalose. Since disaccharides have slower dynamics than water, this can explain why the dynamics of OH-groups is slower in mixtures that have undergone simulated annealing. Furthermore, there are more hydrogen bonds between sucrose molecules than between trehalose molecules, which may be the reason why correlation times are slower for more dehydrated systems with sucrose.

### Free Energy Calculations

**Figure 10:**
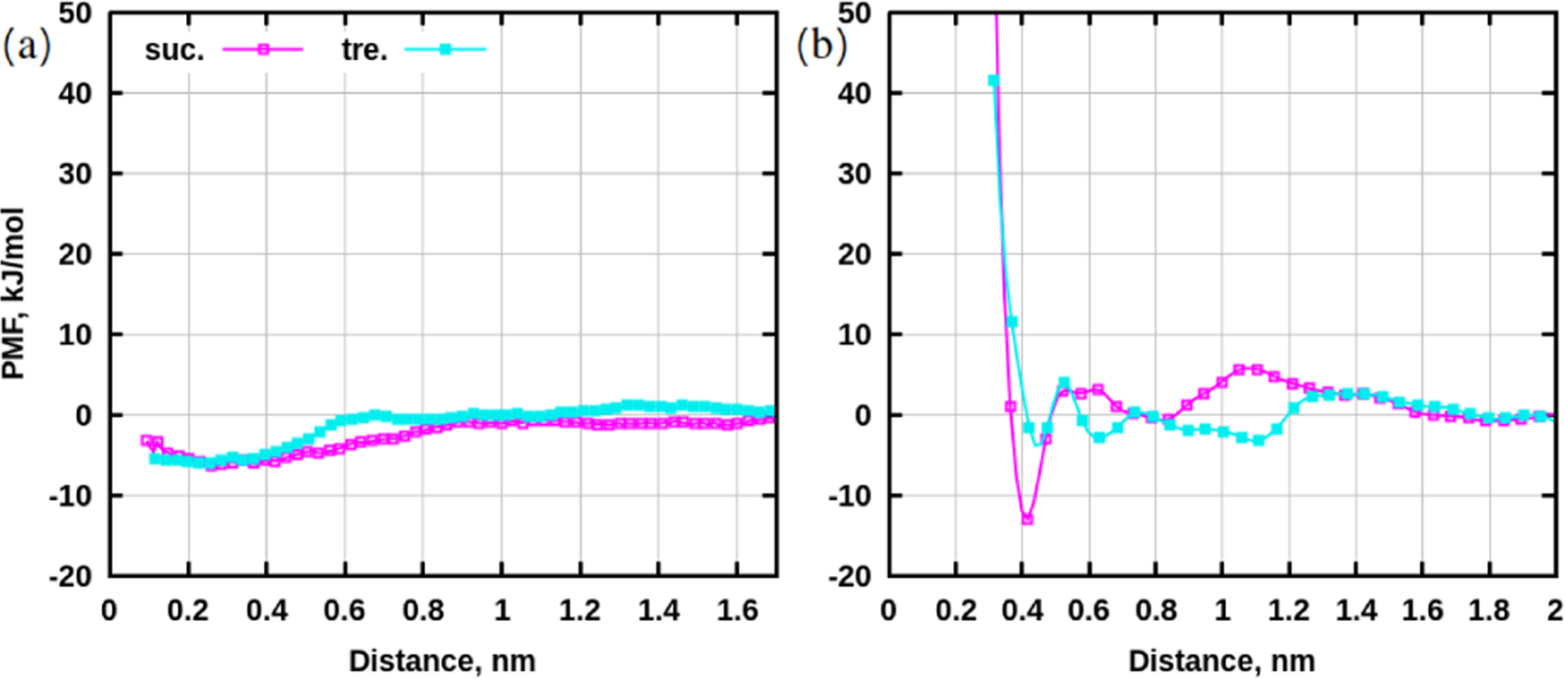
Potential of mean force profiles for sugar–sugar interactions. (a) Umbrella sampling. Potential of mean force for every k-constant are shown in Figure S47 in SI. Histograms are presented in Figure S48 in SI. (b) Well-tempered metadynamics. Gaussian height profiles and collective variables are presented in Figures S49–S50 in SI.

Figure 10 demonstrates potential of mean force profiles for free energy calculations. The depth of curves appears to be in the same energy range for both umbrella sampling (Figure 10a) and well-tempered metadynamics (Figure 10b). In the latter, the difference between disaccharides is well-pronounced: sucrose appears to have a better tendency to aggregate than trehalose, which is coherent with the data from classical MD simulations.

Potential of mean force profiles for sugar–water interactions are presented in Figure 13. The least binding groups to water are ether oxygens O5 in sucrose and O6 in trehalose due to the highest value at the distance of approximately 0.268 nm among all groups for the chosen disaccharide. This result is analogous to the findings obtained from hydrogen bonding in MD simulations.

Nevertheless, potential of mean force profiles give an idea about which molecules can bind, but more accurate information can be obtained from binding free energies, which are computed using the following eq 1:
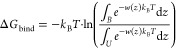
1

**Figure 11:**
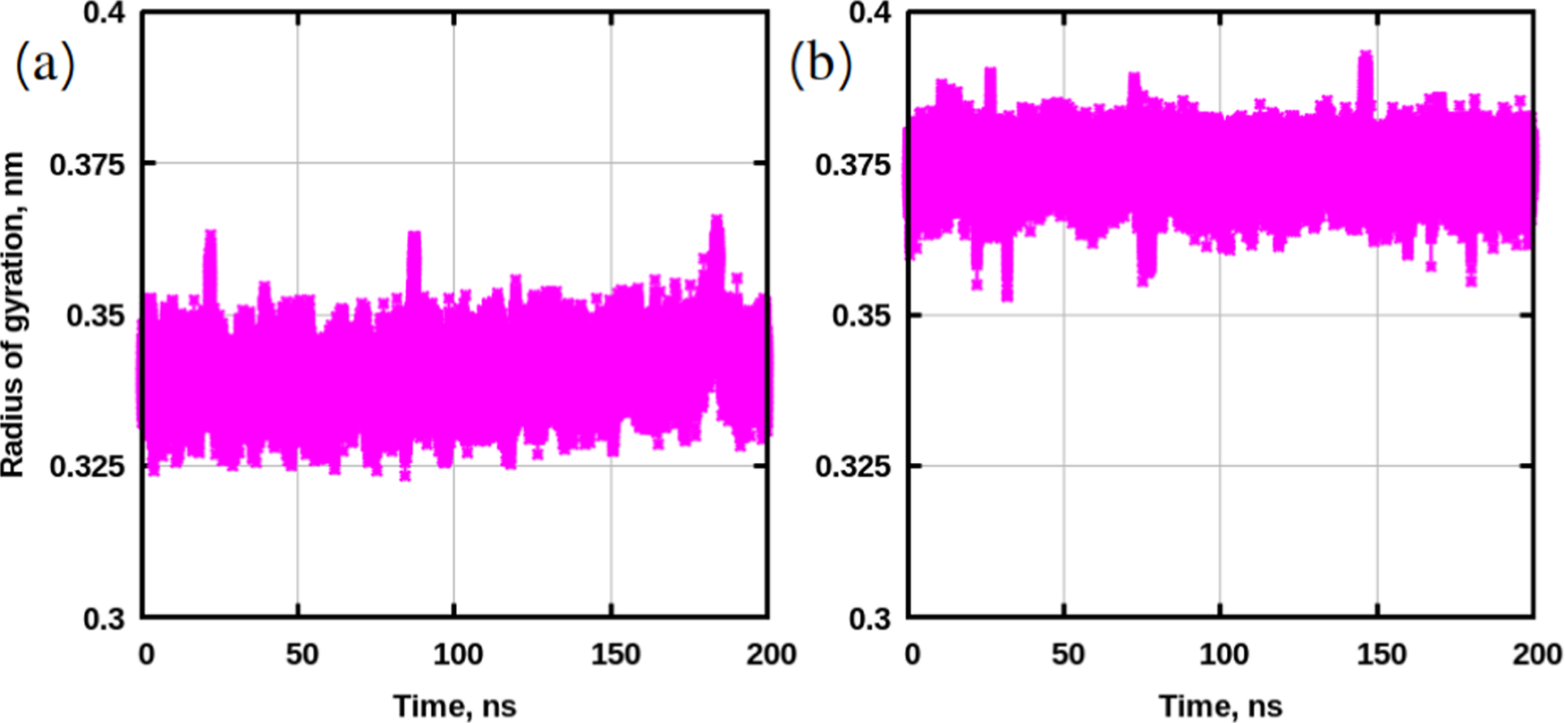
Radius of gyration for disaccharides during well-tempered metadynamics simulations. (a) Sucrose. (b) Trehalose.

Here *U* and *B* are “bound” and “unbound” states, *w*(*z*) is the value of the potential of mean force, *z* is the distance (collective variable), *T* is the temperature in K, and *k*_B_ is the Boltzmann constant. For sucrose and trehalose, the distance for the bound state was taken up to 1.5 nm, and all positions located above 1.5 nm were considered as the unbound state for the integration. Note, that the pulling for umbrella sampling was done up to a distance of 1.7 nm, since pulling to further distances resulted in failures of simulations (molecules were pulled too far). These definitions were made based on the computed radius of gyration (Figure 11) and the knowledge about the sizes of the two disaccharides.

**Figure 12:**
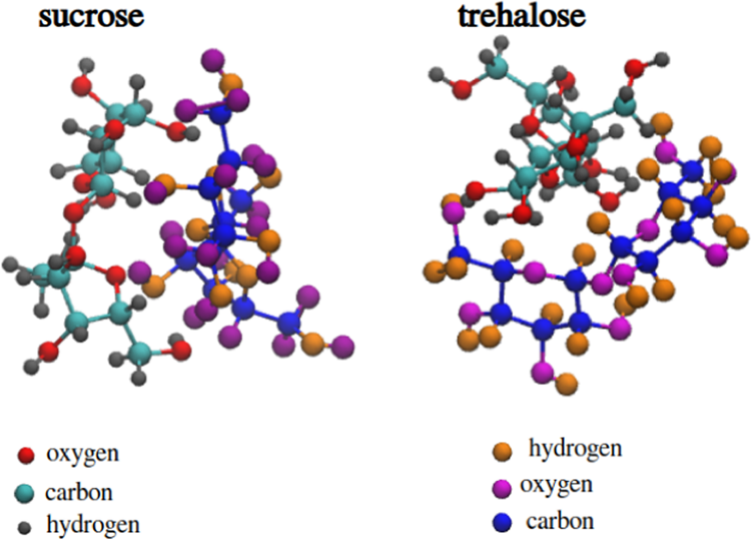
Snapshots from well-tempered metadynamics simulations for sugar–sugar interactions taken at the distance of 0.4 nm. Note: molecules 1 and 2 are shown by different colors here.

Table 2 shows binding free energies computed from the potential of mean force profiles for both sugars. Results from pulling using umbrella sampling exhibit an insignificant difference in the energies of disaccharides, while the data from well-tempered metadynamics more clearly demonstrate that the process of aggregation of sucrose molecules is more energetically favorable compared to that of trehalose. In Figure 12, snapshots taken at a distance of 0.4 nm between centers of mass of molecules can be observed. These are examples of conformations corresponding to the minimal values of the potential of mean force.

**Table 2:** Binding Free Energies for Sugar–Sugar Interactions, kJ/mol

method	sucrose–sucrose	trehalose–trehalose
umbrella sampling	–6.29 ± 0.7	–6.28 ± 0.7
well-tempered metadynamics	–6.69 ± 0.2	–3.13 ± 0.2

Then a reasonable question arises: which methodology gives a more accurate answer about binding free energies: umbrella sampling or well-tempered metadynamics? Well-tempered metadynamics simulations are more trustworthy in this case. The issue with umbrella sampling is that if the motion along the collective variable is limited by the harmonic potential, the sampling of other degrees of freedom will also be limited because two sugars cannot (or can only with difficulty) reorient themselves if the distance between centers of mass is nearly fixed and small. In well-tempered metadynamics, the two sugars can easily move away from each other, reorient, and then come close to each other in different conformations, thus sampling all possible configurations.

The case of binding of water molecules to various groups of sugars is a bit more complex than for sugar–sugar binding. The reason is that, in the case of water, one has to consider that the disaccharide is located in the same environment, and water builds a hydration shell around each selected group. This makes it difficult to define the bound state. Therefore, only potential of mean force profiles and their depths are considered for the analysis of sugar–water interactions.

**Figure 13:**
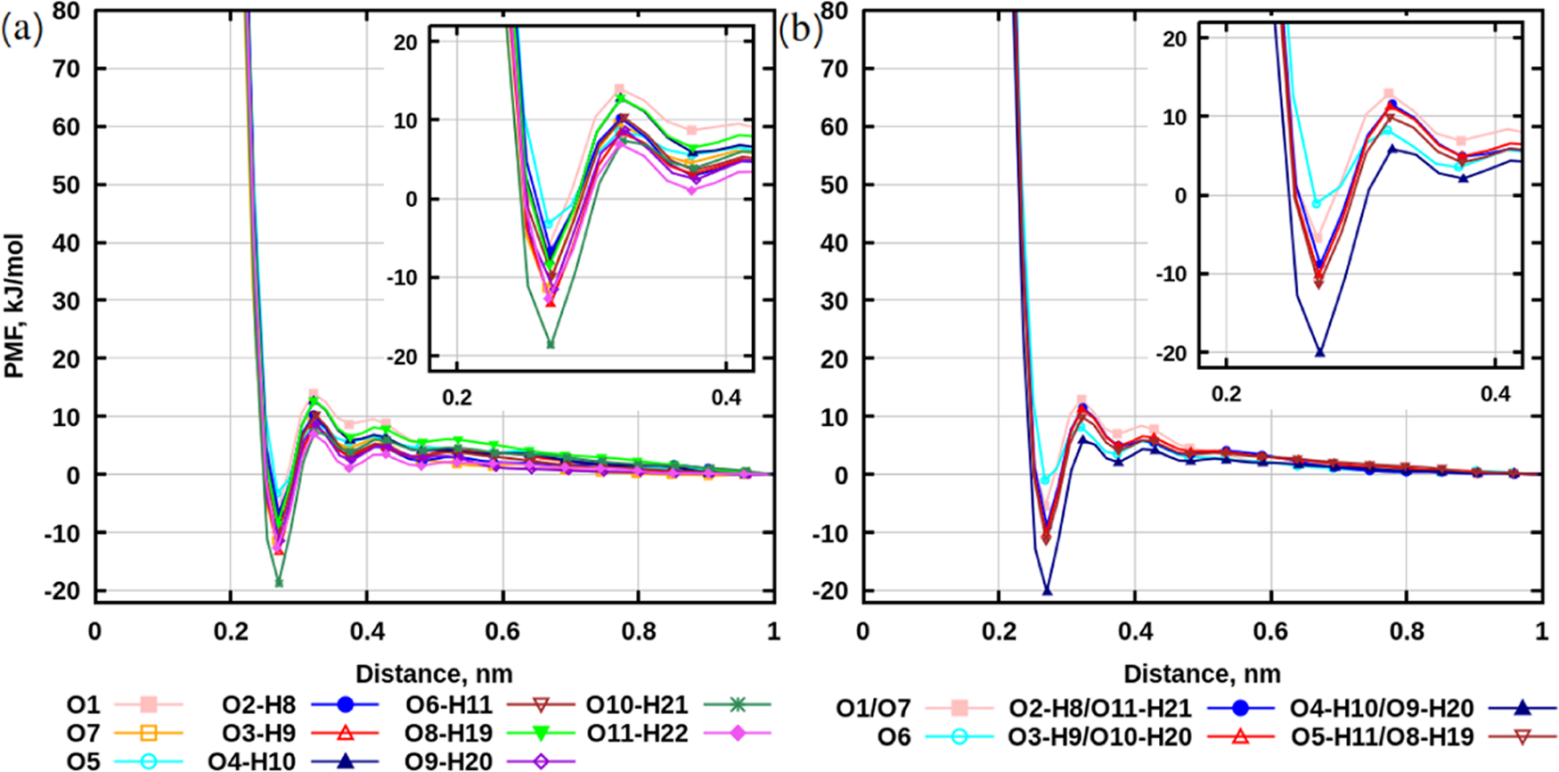
Potential of mean force profiles for sugar–water interactions. (a) Sucrose. (b) Trehalose. Gaussian height profiles and collective variables are presented in Figures S51–S54 in SI.

Hydroxyl groups O10-H21 in sucrose and O4-H10/O9-H20 in trehalose appear to be the most binding to water molecules due to the lowest values of the potential of mean force profiles and a distance of about 0.268 nm (Figure 13). In classical MD simulations with more than 9–178 molecules of disaccharides, these groups were not the most hydrated along the whole range of concentrations. However, in systems with single molecules of sugars in water, the numbers of hydrogen bonds with water for OH-groups of disaccharides were similar due to large statistical errors. Ether oxygens, O5 of sucrose and O6 of trehalose, have the highest values of the potential of mean force, making them the least binding groups in sugars. This is also consistent with results from classical MD simulations.

Since well-tempered metadynamics provide more accurate results due to good sampling and small statistical errors, the evaluation for each group in disaccharides can be done based on the depths in the potential of mean force curves. The ranking for selected groups in sucrose, in decreasing order of the potential of mean force, is as follows: O10-H21 > O3-H9 > O11-H22 > O9-H20 or O7 > O6-H11 > O8-H19 > O4-H10 > O2-H8 > O1 > O5. For the chosen groups of trehalose, the ranking is: O4-H10/O9-H20 > O5-H11/H8-H19 > O3-H9/O10-H20 > O2-H8/O11-H21 > O1/O7 > O6. This evaluation addresses the question of which groups in disaccharides associate with water the strongest.

For the explanation of observations during well-tempered metadynamics, additional comparative analysis was performed: RDFs and distributions of dihedrals were computed for sucrose and trehalose in every simulated system.

For instance, RDFs (Figures S55–S60 in SI) appeared to be very similar to those from MD simulations, but each function displayed an artifact (a peak at a distance below 0.2 nm with a value of *g*(*r*) smaller than 0.1) in the well-tempered metadynamics figures. This indicates other insignificant electrostatic interactions, not related to hydrogen bonds. These interactions might be related to differences in conformations and the appearance of unphysical states during well-tempered metadynamics calculations compared to classical MD simulations, which occur due to the applied force. It is known that the metadynamics technique can be used to scan the most interesting areas of the free energy landscape, which can also allow users to simulate rare events^65−67^ and push the system from local minima. Considering the investigated sugars, it was shown that there can be rotational barriers that might take much longer to overcome during classical MD simulations.^13^ Therefore, it is reasonable to investigate the distribution of dihedrals to either confirm or refute this hypothesis.

Figures S61–S74 of SI demonstrate distributions of chosen dihedrals in sucrose. It is important to note that dihedrals involving hydroxyl and methyl groups, as well as ether- and fructose/glucose oxygens, were considered in calculations for both sucrose and trehalose. In general, the highest probabilities were observed for dihedrals between fructose and glucose rings (approximately 60° and approximately 40° in Figures S61–S71a,b in SI, respectively) and those involving hydroxymethyl (CH_2_OH, which is going to be used further as an abbreviation) groups (Figures S72–S74 in SI), which were up to 0.035 (3.5%). For all other angles (Figures S61–S71c–j in SI), the highest values were below 0.016 (1.6%). No “exotic” conformations were detected during free energy calculations, but shifts in distributions for some dihedrals were observed, i.e., dislocations of global maximal values and swaps between local and global maxima compared to data from classical MD simulations.

Figures S75–S88 of SI show dihedral distributions for trehalose. General trends for angles with the highest probabilities are similar to those for sucrose. For dihedrals between glucose rings (Figures S75–S85a,b), the highest values around 0.035 (3.5%) were observed for the value approximately −70°. For angles involving CH_2_OH-groups, maximal probabilities did not exceed 0.025 (2.5%) for approximately −170° in classical MD simulations, while in free energy calculations, the global maximum could shift to approximately 60° (Figures S86–S88 in SI). All other dihedrals involving hydroxyl groups had lower values of maximal probabilities below 0.016 (1.6%), and no dramatic differences between results from free energy calculations and MD simulations were detected. For this reason, it can be concluded that the rotation around dihedrals involving CH_2_OH-groups plays a significant role in reaching the minimal energy geometries when computing binding free energies between ether-, glucose- oxygens, and hydroxyl groups of trehalose and water.

One question remains: which sugar can potentially bind more water in conditions similar to infinite dilution?^68^ The answer could be trehalose, as the total sum of the depths in potential of mean force profiles for this disaccharide is about −113.46 kJ/mol, while for sucrose this value is −110.18 kJ/mol (values in the first minima were used for calculating the total sum).

## Conclusions

In this work, we have conducted a thorough investigation into the behavior of sucrose and trehalose in mixtures with various water contents and depending on the thermal treatment applied in simulated systems. Several valuable conclusions can be drawn from the results presented in this project (main tabulated results are presented in Table 3).

**Table 3:** Tabulated Conclusive Data

Effect of Preheating on	Interaction/Motion	Method
• highest number of hydrogen bonds	disaccharide–disaccharide	preheating
	water–water	preheating
	disaccharide–water	no preheating
• OH-group’s rotation at below 70 wt % of water	slowest rotation	preheating

Effect of Disaccharide on	Interaction/Motion	Method
• highest number of hydrogen bonds	disaccharide–disaccharide	sucrose
	water–water	sucrose
	disaccharide–water	trehalose
• OH-group’s rotation at below 70 wt % of water	slowest rotation	sucrose

Disaccharide	Interaction/Motion	PMF, kJ/mol (Well-Tempered Metadynamics)
sucrose	disaccharide–disaccharide	–6.28
trehalose	disaccharide–disaccharide	–3.13
sucrose	disaccharide–water	–110.18
trehalose	disaccharide–water	–113.46

Trehalose appears to bind more water than sucrose. Sucrose molecules bind more effectively with each other compared to trehalose molecules. There are more hydrogen bonds between sugar and water than between sugar and sugar for concentrations up to 90 wt % of sugar. These findings are coherent with earlier experimental works by Olsson et al.^27^ and Affouard et al.^24^ This also explains earlier experimental observations of trehalose’s superior ability to inhibit ice formation in aqueous solutions with other biomolecules,^69,70^ despite that in this work the ice formation is not studied. Such ability is related to a combination of hydration and the glass transition behavior of the carbohydrate.^71^ While the statistics from hydrogen bonding describes hydration, the effect of annealing is related to the glass transition behavior.

For water contents between 0 and 30 wt %, there is a sharp increase in the number of sugar–water hydrogen bonds, corresponding to a rapid decrease in the number of sugar–sugar hydrogen bonds. In the interval of 30–70 wt % of water, the number of hydrogen bonds between disaccharides and water increases slowly, while the number of hydrogen bonds between sugars decreases slowly. This highlights discrepancies in hydrogen bonding between mixtures in their liquid state and in their amorphous solid state.

Focusing on specific parts of sugars, ether groups are the least hydrated and form fewer hydrogen bonds with sugars. Hydroxyl groups are the most hydrated and involved in sugar–sugar interactions, due to their location on the surfaces of sugar molecules, making them more exposed to the solvent, as well as the charge distribution: oxygen atoms are more polar than ether oxygens.

The effects of two different system preparations can also be highlighted. Applying preheating has been shown to affect not only the number of hydrogen bonds but also the dynamics of OH-groups in disaccharides. The effects of simulated annealing were particularly pronounced at concentrations below 70 wt % of water. For instance, there were more disaccharide–disaccharide and water–water hydrogen bonds in preheated systems than in those that were not preheated. The rotational dynamics of OH-groups in sugars was slower in preheated mixtures. These findings are also coherent with results from the work by Ekdawi-Sever et al.^72^ who demonstrated the slower dynamics of an aqueous solution of trehalose than the one of sucrose at 80 wt % of disaccharides in their respective mixtures.

However, despite the high temperature applied during annealing, it affected only insignificantly the conformational distribution, which also indicates that the GAFF^73^ is suitable for such simulations and that the thermal treatment did not alter the structures.

In conclusion, we hope this work will assist the computational community in developing further methodologies for simulating mixtures in their amorphous solid state. Due to the small sizes of the systems studied, differences and similarities between mixtures that underwent preheating and those that did not could be observed. For larger structures, more time may be required to reach equilibrium, which can be challenging due to slower molecular motions in the amorphous solid state. Therefore, heating samples above their *T*_g_, then cooling them to the desired temperature, and running the actual simulation at that temperature is a suitable approach for equilibrium studies of mixtures in their amorphous solid state.

## Supporting Information Available

Additional results of analysis of simulations (PDF)
