# Supplementary material for: Sucrose versus Trehalose: Observations from Comparative Study Using Molecular Dynamics Simulations
Source: ACS Omega. 2024 Nov 4;9(46):46323–38. doi: 10.1021/acsomega.4c07314 (PMC11579781; doi:10.1021/acsomega.4c07314)
Supplement: Supplementary file 1 — ao4c07314_si_001.pdf [file ao4c07314_si_001.pdf]

Electronic Supplementary Information for "Sucrose versus Trehalose: Observations from Comparative Study Using Molecular Dynamics Simulations"

Inna Ermilova, [a,b] Alexander Lyubartsev[c] and Vitaly Kocherbitov, [a,b]

[a] *Department of Biomedical Science, Malmö University, SE-205 06 Malmö, Sweden*
[b] *Biofilms Research Center for Biointerfaces, Faculty of Health and Society, Malmö University, SE-205 06 Malmö, Sweden*
[c] *Department of Materials and Environmental Chemistry, Stockholm's University, SE-114 18 Stockholm, Sweden*
* *Corresponding author: inna.ermilova@mau.se; ina.ermilova@gmail.com*

Contents

1 Data for hydrogen bonds with standard deviations **S2**
1.1 Number of hydrogen bonds per oxygen atom in the system . . . S2
1.2 Number of hydrogen bonds per chosen group in sugars . . . S4

2 Radial distribution functions (RDFs) **S9**
2.1 Sucrose . . . S10
2.2 Trehalose . . . S32
2.3 Water-water radial distribution functions in all all systems . . . S44

3 Distributions of dihedrals **S46**
3.1 Sucrose . . . S47
3.2 Trehalose . . . S52

4 Umbrella sampling: histograms and potential of mean force profiles **S55**

5 Well-tempered metadynamics for sugar-sugar: collective variables, Gaussians **S57**

6 Well-tempered metadynamics for sugar-water: collective variables, Gaussians **S58**

7 Comparison of RDFs for systems with 1 molecule of sugar: free energy calculations vs MD **S62**
7.1 Sucrose . . . S62
7.2 Trehalose . . . S65

8 Comparison of dihedrals for systems with 1 molecule of sugar: free energy calculations vs MD **S68**
8.1 Sucrose . . . S68
8.2 Trehalose . . . S82

1 Data for hydrogen bonds with standard deviations

1.1 Number of hydrogen bonds per oxygen atom in the system

Table S1 Number of hydrogen bonds between sugars per oxygen in the system.

Water wt %	sucrose-sucrose (no an.)	sucrose-sucrose (an.)	trehalose-trehalose (no an.)	trehalose-trehalose (an.)
99.95	0.0002 ± 0.0002	0.0003 ± 0.0002	0.0001 ± 0.0002	0.0000 ± 0.0001
99.44	0.0001 ± 0.0001	0.0001 ± 0.0002	0.0000 ± 0.0001	0.0000 ± 0.0000
99.00	0.0003 ± 0.0002	0.0005 ± 0.0004	0.0002 ± 0.0003	0.0000 ± 0.0001
95.00	0.0026 ± 0.0009	0.0025 ± 0.0008	0.0037 ± 0.0015	0.0018 ± 0.0009
90.00	0.0080 ± 0.0014	0.0084 ± 0.0015	0.0075 ± 0.0022	0.0077 ± 0.0030
80.00	0.0252 ± 0.0029	0.0261 ± 0.0024	0.0248 ± 0.0020	0.0306 ± 0.0016
70.00	0.0451 ± 0.0032	0.0469 ± 0.0038	0.0403 ± 0.0041	0.0467 ± 0.0038
60.00	0.0692 ± 0.0037	0.0746 ± 0.0036	0.0602 ± 0.0047	0.0755 ± 0.0025
50.00	0.0965 ± 0.0050	0.1071 ± 0.0035	0.0855 ± 0.0052	0.1074 ± 0.0038
40.00	0.1306 ± 0.0053	0.1475 ± 0.0041	0.1210 ± 0.0072	0.1430 ± 0.0031
30.00	0.1776 ± 0.0050	0.2086 ± 0.0037	0.1696 ± 0.0043	0.1953 ± 0.0038
20.00	0.2653 ± 0.0049	0.2981 ± 0.0037	0.2551 ± 0.0039	0.2813 ± 0.0036
15.00	0.3232 ± 0.0042	0.3503 ± 0.0046	0.3222 ± 0.0045	0.3527 ± 0.0044
10.00	0.4104 ± 0.0057	0.4272 ± 0.0045	0.4062 ± 0.0058	0.4175 ± 0.0046
5.00	0.5173 ± 0.0059	0.5329 ± 0.0054	0.4876 ± 0.0053	0.5173 ± 0.0054
1.00	0.6092 ± 0.0064	0.6290 ± 0.0055	0.5874 ± 0.0077	0.6094 ± 0.0060
0.00	0.6330 ± 0.0061	0.6596 ± 0.0058	0.6011 ± 0.0083	0.6347 ± 0.0061

Table S2 Number of hydrogen bonds between sugars and water per oxygen in the system.

Water wt %	sucrose-water (no an.)	sucrose-water (an.)	trehalose-water (no an.)	trehalose-water (an.)
99.95	0.0100 ± 0.0007	0.0098 ± 0.0007	0.0101 ± 0.0007	0.0102 ± 0.0007
99.44	0.0055 ± 0.0005	0.0055 ± 0.0005	0.0057 ± 0.0005	0.0057 ± 0.0005
99.00	0.0109 ± 0.0008	0.0104 ± 0.0010	0.0080 ± 0.0010	0.0113 ± 0.0007
95.00	0.0467 ± 0.0023	0.0469 ± 0.0021	0.0435 ± 0.0036	0.0478 ± 0.0025
90.00	0.0891 ± 0.0036	0.0876 ± 0.0038	0.0883 ± 0.0054	0.0880 ± 0.0073
80.00	0.1642 ± 0.0070	0.1624 ± 0.0057	0.1637 ± 0.0050	0.1503 ± 0.0042
70.00	0.2383 ± 0.0073	0.2351 ± 0.0089	0.2478 ± 0.0099	0.2324 ± 0.0099
60.00	0.3222 ± 0.0087	0.3084 ± 0.0086	0.3407 ± 0.0110	0.2998 ± 0.0060
50.00	0.4104 ± 0.0128	0.3843 ± 0.0073	0.4341 ± 0.0128	0.3816 ± 0.0090
40.00	0.5000 ± 0.0115	0.4579 ± 0.0083	0.5238 ± 0.0134	0.4621 ± 0.0068
30.00	0.5697 ± 0.0118	0.4982 ± 0.0074	0.5832 ± 0.0083	0.5135 ± 0.0077
20.00	0.5501 ± 0.0086	0.4826 ± 0.0061	0.5540 ± 0.0075	0.5061 ± 0.0063
15.00	0.5064 ± 0.0070	0.4486 ± 0.0065	0.4827 ± 0.0070	0.4333 ± 0.0062
10.00	0.3837 ± 0.0064	0.3625 ± 0.0048	0.3720 ± 0.0059	0.3762 ± 0.0051
5.00	0.2183 ± 0.0038	0.2221 ± 0.0043	0.2249 ± 0.0041	0.2183 ± 0.0035
1.00	0.0501 ± 0.0020	0.0543 ± 0.0017	0.0521 ± 0.0020	0.0520 ± 0.0019

Table S3 Number of hydrogen bonds between water molecules per oxygen in the system.

Water wt %	sucrose (no an.)	sucrose (an.)	trehalose (no an.)	trehalose (an.)
99.95	1.7867 ± 0.0115	1.7866 ± 0.0111	1.7866 ± 0.0115	1.7864 ± 0.0111
99.44	1.7939 ± 0.0117	1.7939 ± 0.0113	1.7938 ± 0.0117	1.7939 ± 0.0114
99.00	1.7850 ± 0.0117	1.7852 ± 0.0113	1.7849 ± 0.0117	1.7847 ± 0.0113
95.00	1.7228 ± 0.0115	1.7227 ± 0.0111	1.7246 ± 0.0116	1.7223 ± 0.0111
90.00	1.6427 ± 0.0112	1.6437 ± 0.0109	1.6433 ± 0.0114	1.6434 ± 0.0114
80.00	1.4806 ± 0.0110	1.4814 ± 0.0104	1.4806 ± 0.0106	1.4880 ± 0.0103
70.00	1.3129 ± 0.0103	1.3142 ± 0.0104	1.3073 ± 0.0109	1.3156 ± 0.0108
60.00	1.1204 ± 0.0097	1.1278 ± 0.0095	1.1097 ± 0.0103	1.1325 ± 0.0090
50.00	0.9100 ± 0.0099	0.9248 ± 0.0082	0.8970 ± 0.0101	0.9251 ± 0.0089
40.00	0.6808 ± 0.0084	0.7050 ± 0.0075	0.6663 ± 0.0088	0.7011 ± 0.0071
30.00	0.4464 ± 0.0077	0.4839 ± 0.0063	0.4316 ± 0.0062	0.4747 ± 0.0062
20.00	0.2304 ± 0.0056	0.2674 ± 0.0047	0.2265 ± 0.0047	0.2511 ± 0.0045
15.00	0.1320 ± 0.0036	0.1644 ± 0.0038	0.1408 ± 0.0040	0.1682 ± 0.0037
10.00	0.0674 ± 0.0037	0.0812 ± 0.0027	0.0714 ± 0.0033	0.0724 ± 0.0027
5.00	0.0207 ± 0.0018	0.0221 ± 0.0016	0.0181 ± 0.0019	0.0224 ± 0.0017
1.00	0.0007 ± 0.0004	0.0004 ± 0.0004	0.0002 ± 0.0003	0.0014 ± 0.0004

1.2 Number of hydrogen bonds per chosen group in sugars

Table S4 Number of hydrogen bonds between sucrose molecules for selected groups in this sugar (systems without pre-heating). Calculations are done per one group in a single molecule of sugar.

Water wt %	O5	O7	O1	O2-H8	O3-H9	O4-H10
99.95	0.0000 ± 0.0035	0.0010 ± 0.0245	0.2145 ± 0.3295	0.0390 ± 0.1340	0.0010 ± 0.0230	0.0110 ± 0.0730
99.44	0.0000 ± 0.0000	0.0010 ± 0.0240	0.1760 ± 0.3810	0.0600 ± 0.2370	0.0000 ± 0.0000	0.0040 ± 0.0610
99.00	0.0000 ± 0.0060	0.0035 ± 0.0430	0.3235 ± 0.3560	0.0535 ± 0.1560	0.0025 ± 0.0335	0.0120 ± 0.0765
95.00	0.0046 ± 0.0223	0.0146 ± 0.0394	0.3381 ± 0.1323	0.1817 ± 0.1342	0.0800 ± 0.0894	0.1558 ± 0.1464
90.00	0.0109 ± 0.0244	0.0438 ± 0.0495	0.3907 ± 0.1001	0.2357 ± 0.1031	0.1689 ± 0.0931	0.2612 ± 0.1134
80.00	0.0221 ± 0.0239	0.0713 ± 0.0459	0.3714 ± 0.0842	0.4751 ± 0.1113	0.3202 ± 0.1129	0.4331 ± 0.1119
70.00	0.0177 ± 0.0178	0.1032 ± 0.0468	0.3453 ± 0.0636	0.5415 ± 0.0842	0.4178 ± 0.0696	0.4872 ± 0.0985
60.00	0.0346 ± 0.0199	0.1100 ± 0.0306	0.3676 ± 0.0505	0.5468 ± 0.0630	0.4624 ± 0.0665	0.5681 ± 0.0642
50.00	0.0199 ± 0.0147	0.1084 ± 0.0327	0.3552 ± 0.0446	0.6159 ± 0.0560	0.4826 ± 0.0508	0.6041 ± 0.0619
40.00	0.0216 ± 0.0131	0.1482 ± 0.0309	0.3840 ± 0.0488	0.5697 ± 0.0458	0.5055 ± 0.0508	0.5794 ± 0.0622
30.00	0.0302 ± 0.0148	0.1285 ± 0.0228	0.4069 ± 0.0579	0.6963 ± 0.0409	0.5493 ± 0.0293	0.5600 ± 0.0492
20.00	0.0200 ± 0.0097	0.1412 ± 0.0181	0.4700 ± 0.0408	0.8757 ± 0.0329	0.7130 ± 0.0459	0.7428 ± 0.0497
15.00	0.0444 ± 0.0169	0.1250 ± 0.0198	0.4887 ± 0.0294	0.9002 ± 0.0288	0.8317 ± 0.0297	0.8151 ± 0.0259
10.00	0.0293 ± 0.0136	0.2009 ± 0.0193	0.4558 ± 0.0437	1.0663 ± 0.0403	0.9738 ± 0.0336	1.0268 ± 0.0297
5.00	0.0662 ± 0.0157	0.2113 ± 0.0204	0.4826 ± 0.0251	1.2864 ± 0.0289	1.1217 ± 0.0357	1.2307 ± 0.0297
1.00	0.0636 ± 0.0143	0.2371 ± 0.0183	0.4867 ± 0.0325	1.4068 ± 0.0344	1.1635 ± 0.0325	1.3955 ± 0.0375
0.00	0.0930 ± 0.0165	0.2163 ± 0.0171	0.4959 ± 0.0241	1.4323 ± 0.0294	1.1659 ± 0.0309	1.3513 ± 0.0286

Table S5 Number of hydrogen bonds between sucrose molecules for selected groups in this sugar (systems without pre-heating). Calculations are done per one group in a single molecule of sugar.

Water wt %	O8-H19	O9-H20	O10-H21	O11-H22	O6-H11
99.95	0.0105 ± 0.0725	0.0345 ± 0.1330	0.2230 ± 0.3355	0.0335 ± 0.1315	0.0430 ± 0.1405
99.44	0.0050 ± 0.0670	0.0690 ± 0.2530	0.1830 ± 0.3880	0.0660 ± 0.2480	0.0620 ± 0.2410
99.00	0.0140 ± 0.0825	0.0910 ± 0.2010	0.3365 ± 0.3600	0.0925 ± 0.2000	0.0600 ± 0.1660
95.00	0.1639 ± 0.1229	0.1432 ± 0.1391	0.4049 ± 0.1576	0.2213 ± 0.1651	0.1204 ± 0.1154
90.00	0.3196 ± 0.1299	0.2847 ± 0.1029	0.5383 ± 0.1316	0.2931 ± 0.1229	0.2157 ± 0.1091
80.00	0.4903 ± 0.1210	0.3580 ± 0.0811	0.6006 ± 0.0882	0.4414 ± 0.1078	0.4321 ± 0.1113
70.00	0.6254 ± 0.0870	0.4480 ± 0.0781	0.6323 ± 0.0838	0.5583 ± 0.0876	0.5028 ± 0.0868
60.00	0.6656 ± 0.0603	0.4989 ± 0.0739	0.6937 ± 0.0716	0.6587 ± 0.0835	0.5294 ± 0.0787
50.00	0.6478 ± 0.0648	0.5317 ± 0.0639	0.7473 ± 0.0679	0.7140 ± 0.0650	0.5919 ± 0.0562
40.00	0.7480 ± 0.0883	0.6332 ± 0.0626	0.8496 ± 0.0613	0.7748 ± 0.0707	0.6782 ± 0.0685
30.00	0.7508 ± 0.0532	0.7498 ± 0.0417	0.9112 ± 0.0485	0.9023 ± 0.0750	0.7965 ± 0.0625
20.00	0.8783 ± 0.0323	0.8100 ± 0.0304	1.1376 ± 0.0342	1.1277 ± 0.0451	1.0699 ± 0.0373
15.00	0.9894 ± 0.0329	0.9862 ± 0.0339	1.2241 ± 0.0395	1.1976 ± 0.0292	1.1672 ± 0.0333
10.00	1.1790 ± 0.0304	1.0947 ± 0.0305	1.2136 ± 0.0356	1.4843 ± 0.0376	1.3930 ± 0.0348
5.00	1.3643 ± 0.0368	1.3492 ± 0.0347	1.4452 ± 0.0391	1.6112 ± 0.0452	1.5899 ± 0.0305
1.00	1.5142 ± 0.0330	1.4481 ± 0.0368	1.5621 ± 0.0348	1.8794 ± 0.0297	1.7914 ± 0.0367
0.00	1.6278 ± 0.0304	1.4545 ± 0.0317	1.5979 ± 0.0344	1.8783 ± 0.0312	1.8574 ± 0.0319

Table S6 Number of hydrogen bonds between sucrose molecules for selected groups in this sugar (systems with pre-heating). Calculations are done per one group in a single molecule of sugar.

Water wt %	O5	O7	O1	O2-H8	O3-H9	O4-H10
99.95	0.0000 ± 0.0060	0.0010 ± 0.0240	0.2875 ± 0.2930	0.0815 ± 0.1955	0.0020 ± 0.0325	0.0115 ± 0.0745
99.44	0.0000 ± 0.0000	0.0020 ± 0.0430	0.3400 ± 0.4740	0.0400 ± 0.1960	0.0000 ± 0.0080	0.0060 ± 0.0750
99.00	0.0175 ± 0.0935	0.0345 ± 0.1320	0.5530 ± 0.3005	0.0805 ± 0.2080	0.0465 ± 0.1650	0.0810 ± 0.2275
95.00	0.0101 ± 0.0326	0.0188 ± 0.0470	0.3214 ± 0.1619	0.1801 ± 0.1272	0.0741 ± 0.0972	0.1502 ± 0.1327
90.00	0.0114 ± 0.0252	0.0528 ± 0.0558	0.3443 ± 0.1121	0.2828 ± 0.1164	0.2341 ± 0.1184	0.3339 ± 0.1110
80.00	0.0163 ± 0.0209	0.0718 ± 0.0526	0.4125 ± 0.0749	0.4883 ± 0.1057	0.3733 ± 0.0789	0.4460 ± 0.0825
70.00	0.0141 ± 0.0159	0.1487 ± 0.0420	0.3694 ± 0.0624	0.4910 ± 0.0662	0.3479 ± 0.0794	0.5184 ± 0.0928
60.00	0.0203 ± 0.0176	0.1363 ± 0.0278	0.2871 ± 0.0439	0.6404 ± 0.0920	0.5134 ± 0.0579	0.5583 ± 0.0475
50.00	0.0225 ± 0.0179	0.1197 ± 0.0274	0.3637 ± 0.0356	0.7497 ± 0.0540	0.5214 ± 0.0491	0.6348 ± 0.0603
40.00	0.0399 ± 0.0161	0.1138 ± 0.0260	0.3824 ± 0.0361	0.6749 ± 0.0546	0.6023 ± 0.0470	0.6608 ± 0.0377
30.00	0.0392 ± 0.0170	0.1704 ± 0.0219	0.4212 ± 0.0286	0.8592 ± 0.0355	0.7293 ± 0.0357	0.8384 ± 0.0387
20.00	0.0510 ± 0.0156	0.1645 ± 0.0169	0.3740 ± 0.0215	0.9998 ± 0.0312	0.8485 ± 0.0325	0.9364 ± 0.0266
15.00	0.0529 ± 0.0143	0.1745 ± 0.0176	0.4074 ± 0.0198	1.0859 ± 0.0348	0.9007 ± 0.0262	1.0429 ± 0.0339
10.00	0.0543 ± 0.0140	0.1976 ± 0.0174	0.3692 ± 0.0229	1.1181 ± 0.0325	1.0031 ± 0.0281	1.2422 ± 0.0288
5.00	0.0580 ± 0.0137	0.2853 ± 0.0217	0.4363 ± 0.0196	1.2627 ± 0.0248	1.2151 ± 0.0291	1.3403 ± 0.0293
1.00	0.0747 ± 0.0158	0.2697 ± 0.0176	0.4028 ± 0.0192	1.5027 ± 0.0283	1.3138 ± 0.0267	1.4778 ± 0.0274
0.00	0.0849 ± 0.0161	0.2918 ± 0.0201	0.3927 ± 0.0193	1.5055 ± 0.0271	1.3058 ± 0.0306	1.5366 ± 0.0290

Table S7 Number of hydrogen bonds between sucrose molecules for selected groups in this sugar (systems with pre-heating). Calculations are done per one group in a single molecule of sugar.

Water wt %	O8-H19	O9-H20	O10-H21	O11-H22	O6-H11
99.95	0.0095 ± 0.0695	0.0840 ± 0.1915	0.2990 ± 0.2975	0.0795 ± 0.1880	0.0920 ± 0.2080
99.44	0.0050 ± 0.0710	0.0230 ± 0.1510	0.3480 ± 0.4790	0.0230 ± 0.1500	0.0450 ± 0.2080
99.00	0.3515 ± 0.2830	0.1335 ± 0.2330	0.2575 ± 0.2700	0.1675 ± 0.2980	0.0725 ± 0.1990
95.00	0.1800 ± 0.1457	0.1278 ± 0.1198	0.4030 ± 0.1913	0.1721 ± 0.1277	0.1334 ± 0.1213
90.00	0.3168 ± 0.1162	0.2191 ± 0.0992	0.4696 ± 0.1239	0.0272 ± 0.0522	0.2672 ± 0.1336
80.00	0.4871 ± 0.0988	0.3720 ± 0.0856	0.6569 ± 0.1006	0.5040 ± 0.1095	0.4503 ± 0.0954
70.00	0.6301 ± 0.0717	0.4952 ± 0.0820	0.7485 ± 0.0737	0.6745 ± 0.1204	0.5219 ± 0.0840
60.00	0.7242 ± 0.0727	0.5235 ± 0.0588	0.7625 ± 0.0721	0.7471 ± 0.0782	0.7313 ± 0.0775
50.00	0.7443 ± 0.0557	0.6153 ± 0.0485	0.8385 ± 0.0541	0.7101 ± 0.0506	0.7026 ± 0.0761
40.00	0.9200 ± 0.0468	0.7244 ± 0.0515	0.9101 ± 0.0407	0.8650 ± 0.0495	0.7342 ± 0.0597
30.00	0.9165 ± 0.0363	0.8233 ± 0.0362	0.9424 ± 0.0349	0.9648 ± 0.0384	0.8702 ± 0.0388
20.00	1.1476 ± 0.0267	0.9819 ± 0.0340	1.0250 ± 0.0341	1.2411 ± 0.0323	1.1581 ± 0.0331
15.00	1.2077 ± 0.0314	1.0057 ± 0.0294	1.2167 ± 0.0341	1.3365 ± 0.0349	1.1803 ± 0.0431
10.00	1.3480 ± 0.0268	1.1431 ± 0.0281	1.1789 ± 0.0346	1.5576 ± 0.0400	1.3988 ± 0.0315
5.00	1.4948 ± 0.0249	1.3643 ± 0.0250	1.4748 ± 0.0307	1.6583 ± 0.0277	1.5627 ± 0.0297
1.00	1.5640 ± 0.0300	1.4760 ± 0.0255	1.5209 ± 0.0306	1.9372 ± 0.0321	1.8474 ± 0.0293
0.00	1.6710 ± 0.0262	1.5827 ± 0.0261	1.5892 ± 0.0313	1.9605 ± 0.0292	1.8620 ± 0.0299

Table S8 Number of hydrogen bonds between trehalose molecules for selected groups in this sugar (systems without pre-heating). Calculations are done per one group in a single molecule of sugar.

Water wt %	O1/O7	O6	O5-H11/O8-H9	O4-H10/O9-H20	O3-H9/O10-H21	O2-H8/O11-H22
99.95	0.0295 ± 0.1340	0.0005 ± 0.0190	0.0455 ± 0.1820	0.0155 ± 0.0875	0.0315 ± 0.1300	0.0560 ± 0.2310
99.44	0.0410 ± 0.1980	0.0000 ± 0.0000	0.0420 ± 0.2030	0.0000 ± 0.0070	0.0080 ± 0.0900	0.0090 ± 0.0960
99.00	0.0845 ± 0.2110	0.0155 ± 0.0875	0.1955 ± 0.3655	0.0915 ± 0.2215	0.0380 ± 0.1365	0.0935 ± 0.2350
95.00	0.3623 ± 0.1429	0.0447 ± 0.0664	0.6623 ± 0.2912	0.5007 ± 0.2732	0.3611 ± 0.2204	0.4511 ± 0.2157
90.00	0.2390 ± 0.1316	0.0617 ± 0.0634	0.7556 ± 0.2696	0.5288 ± 0.1992	0.3518 ± 0.1327	0.4251 ± 0.1717
80.00	0.3177 ± 0.0684	0.0620 ± 0.0372	1.1043 ± 0.1315	0.8642 ± 0.1264	0.6510 ± 0.1093	0.7646 ± 0.1166
70.00	0.3390 ± 0.0485	0.0477 ± 0.0272	1.0631 ± 0.1670	0.8486 ± 0.1220	0.7831 ± 0.1241	0.8521 ± 0.1164
60.00	0.3170 ± 0.0661	0.0499 ± 0.0244	1.0598 ± 0.1536	0.9973 ± 0.1051	0.8712 ± 0.1333	0.9117 ± 0.1203
50.00	0.2777 ± 0.0401	0.0423 ± 0.0213	1.1120 ± 0.1272	1.0041 ± 0.0953	0.9829 ± 0.0677	1.0613 ± 0.0848
40.00	0.2897 ± 0.0502	0.0576 ± 0.0190	1.3245 ± 0.1128	1.1833 ± 0.1083	1.0800 ± 0.0927	1.1307 ± 0.0796
30.00	0.2803 ± 0.0289	0.0493 ± 0.0172	1.4161 ± 0.0612	1.3294 ± 0.0534	1.3008 ± 0.0559	1.4216 ± 0.0473
20.00	0.4551 ± 0.0219	0.0806 ± 0.0201	1.7257 ± 0.0484	1.6263 ± 0.0643	1.6111 ± 0.0465	1.7935 ± 0.0550
15.00	0.4423 ± 0.0228	0.0933 ± 0.0192	1.9115 ± 0.0581	1.8003 ± 0.0449	1.8637 ± 0.0385	2.0411 ± 0.0449
10.00	0.5457 ± 0.0329	0.1285 ± 0.0184	2.2277 ± 0.0660	2.1688 ± 0.0580	2.0148 ± 0.0389	2.4257 ± 0.0550
5.00	0.6413 ± 0.0258	0.1329 ± 0.0214	2.4178 ± 0.0429	2.2710 ± 0.0432	2.2869 ± 0.0432	2.6275 ± 0.0461
1.00	0.6972 ± 0.0370	0.1757 ± 0.0270	2.7572 ± 0.0595	2.6083 ± 0.0494	2.4931 ± 0.0406	3.0588 ± 0.0529
0.00	0.7057 ± 0.0362	0.1798 ± 0.0312	2.7556 ± 0.0540	2.6166 ± 0.0693	2.4253 ± 0.0596	3.1779 ± 0.0598

Table S9 Number of hydrogen bonds between trehalose molecules for selected groups in this sugar (systems with pre-heating). Calculations are done per one group in a single molecule of sugar.

Water wt %	O1/O7	O6	O5-H11/O8-H9	O4-H10/O9-H20	O3-H9/O10-H21	O2-H8/O11-H22
99.95	0.0220 ± 0.1105	0.0000 ± 0.0000	0.0330 ± 0.1350	0.0200 ± 0.1060	0.0135 ± 0.0820	0.0160 ± 0.0895
99.44	0.0130 ± 0.1120	0.0000 ± 0.0000	0.0130 ± 0.1130	0.0000 ± 0.0050	0.0070 ± 0.0850	0.0080 ± 0.0860
99.00	0.0280 ± 0.1155	0.0005 ± 0.0120	0.0325 ± 0.1250	0.0045 ± 0.0470	0.0110 ± 0.0755	0.0085 ± 0.0645
95.00	0.1028 ± 0.0944	0.0213 ± 0.0468	0.3682 ± 0.2251	0.2510 ± 0.1667	0.2027 ± 0.1483	0.1962 ± 0.1422
90.00	0.2239 ± 0.1061	0.0509 ± 0.0548	0.8002 ± 0.3481	0.6143 ± 0.2817	0.4242 ± 0.1987	0.4201 ± 0.1583
80.00	0.3688 ± 0.0534	0.0788 ± 0.0413	1.2231 ± 0.0957	1.1690 ± 0.0917	0.8859 ± 0.0915	0.9846 ± 0.1220
70.00	0.4341 ± 0.0569	0.0684 ± 0.0339	1.3145 ± 0.1712	1.0389 ± 0.0938	0.8252 ± 0.0785	1.0130 ± 0.1292
60.00	0.3637 ± 0.0293	0.0896 ± 0.0278	1.4448 ± 0.0653	1.2424 ± 0.0732	0.9456 ± 0.0730	1.2332 ± 0.0771
50.00	0.4646 ± 0.0310	0.0895 ± 0.0274	1.4409 ± 0.0597	1.3739 ± 0.0632	1.1931 ± 0.0791	1.2859 ± 0.0712
40.00	0.4649 ± 0.0322	0.0813 ± 0.0224	1.5871 ± 0.0453	1.3676 ± 0.0557	1.2400 ± 0.0494	1.2204 ± 0.0610
30.00	0.4028 ± 0.0242	0.0789 ± 0.0193	1.6223 ± 0.0463	1.4980 ± 0.0442	1.4593 ± 0.0446	1.5648 ± 0.0473
20.00	0.4660 ± 0.0221	0.1163 ± 0.0181	1.8139 ± 0.0399	1.8543 ± 0.0351	1.7913 ± 0.0476	1.8950 ± 0.0392
15.00	0.7209 ± 0.0241	0.1002 ± 0.0205	2.1673 ± 0.0436	2.1219 ± 0.0373	1.8659 ± 0.0369	2.1236 ± 0.0426
10.00	0.6998 ± 0.0238	0.1052 ± 0.0194	2.2762 ± 0.0384	2.3054 ± 0.0373	2.1052 ± 0.0349	2.4303 ± 0.0383
5.00	0.7135 ± 0.0260	0.1567 ± 0.0208	2.5370 ± 0.0397	2.5160 ± 0.0366	2.3997 ± 0.0399	2.8593 ± 0.0422
1.00	0.7786 ± 0.0261	0.1725 ± 0.0222	2.7517 ± 0.0421	2.7723 ± 0.0397	2.6658 ± 0.0410	3.0542 ± 0.0411
0.00	0.7606 ± 0.0270	0.1308 ± 0.0194	2.8417 ± 0.0406	2.7936 ± 0.0398	2.7505 ± 0.0391	3.3301 ± 0.0418

Table S10 Number of hydrogen bonds between sucrose and water molecules for selected groups in this sugar (systems without pre-heating). Calculations are done per one group in a single molecule of sugar.

Water wt %	O5	O7	O1	O2-H8	O3-H9	O4-H10
99.95	0.1473 ± 0.2540	0.6472 ± 0.3453	0.3698 ± 0.3466	1.9644 ± 0.3885	1.9238 ± 0.3762	2.1629 ± 0.4505
99.44	0.1423 ± 0.3548	0.6515 ± 0.4916	0.3672 ± 0.4857	1.9444 ± 0.5686	1.9158 ± 0.5357	2.1768 ± 0.6291
99.00	0.1443 ± 0.2531	0.6507 ± 0.3482	0.3325 ± 0.3416	1.9504 ± 0.3948	1.9170 ± 0.3819	2.1641 ± 0.4501
95.00	0.1237 ± 0.1110	0.6145 ± 0.1646	0.3041 ± 0.1529	1.7996 ± 0.2194	1.8288 ± 0.2004	1.9712 ± 0.2626
90.00	0.1156 ± 0.0764	0.5776 ± 0.1210	0.2740 ± 0.1051	1.7349 ± 0.1579	1.7173 ± 0.1559	1.8320 ± 0.1932
80.00	0.0973 ± 0.0495	0.5067 ± 0.0876	0.2491 ± 0.0745	1.4336 ± 0.1408	1.5120 ± 0.1496	1.6145 ± 0.1604
70.00	0.0921 ± 0.0397	0.4614 ± 0.0667	0.2317 ± 0.0578	1.3643 ± 0.1014	1.4157 ± 0.0975	1.5404 ± 0.1461
60.00	0.0915 ± 0.0338	0.4648 ± 0.0551	0.2391 ± 0.0453	1.3245 ± 0.0811	1.3258 ± 0.0921	1.4411 ± 0.0915
50.00	0.0918 ± 0.0305	0.4624 ± 0.0589	0.2316 ± 0.0423	1.2545 ± 0.0714	1.3142 ± 0.0765	1.3874 ± 0.0887
40.00	0.0978 ± 0.0282	0.4071 ± 0.0420	0.2510 ± 0.0402	1.2932 ± 0.0612	1.2762 ± 0.0693	1.4036 ± 0.0806
30.00	0.0866 ± 0.0245	0.3546 ± 0.0379	0.2300 ± 0.0374	1.1364 ± 0.0504	1.2261 ± 0.0459	1.4325 ± 0.0572
20.00	0.0663 ± 0.0198	0.3033 ± 0.0293	0.1784 ± 0.0238	0.9256 ± 0.0402	0.9471 ± 0.0573	1.1243 ± 0.0450
15.00	0.0617 ± 0.0178	0.2495 ± 0.0264	0.1555 ± 0.0228	0.7619 ± 0.0321	0.7864 ± 0.0361	0.9233 ± 0.0381
10.00	0.0509 ± 0.0149	0.1635 ± 0.0290	0.1153 ± 0.0167	0.5876 ± 0.0302	0.5402 ± 0.0305	0.6143 ± 0.0264
5.00	0.0262 ± 0.0109	0.0843 ± 0.0153	0.0534 ± 0.0125	0.2680 ± 0.0212	0.2577 ± 0.0183	0.3293 ± 0.0204
1.00	0.0049 ± 0.0047	0.0186 ± 0.0069	0.0216 ± 0.0057	0.0509 ± 0.0095	0.0892 ± 0.0154	0.0700 ± 0.0101

Table S11 Number of hydrogen bonds between sucrose and water molecules for selected groups in this sugar (systems without pre-heating). Calculations are done per one group in a single molecule of sugar.

Water wt %	O8-H19	O9-H20	O10-H21	O11-H22	O6-H11
99.95	2.4286 ± 0.4298	2.0181 ± 0.3656	1.8327 ± 0.4883	2.5668 ± 0.4388	2.4823 ± 0.4404
99.44	2.4364 ± 0.6050	2.0023 ± 0.5268	1.8693 ± 0.6678	2.5569 ± 0.6229	2.4648 ± 0.6322
99.00	2.4215 ± 0.4299	1.9720 ± 0.3792	1.7574 ± 0.4937	2.5255 ± 0.4493	2.4339 ± 0.4481
95.00	2.2265 ± 0.2351	1.9163 ± 0.2091	1.6746 ± 0.2357	2.3717 ± 0.2562	2.3616 ± 0.2260
90.00	2.0045 ± 0.2040	1.7445 ± 0.1517	1.5251 ± 0.1829	2.2480 ± 0.1852	2.2280 ± 0.1787
80.00	1.7697 ± 0.1508	1.6554 ± 0.1095	1.3825 ± 0.1213	2.0441 ± 0.1721	1.9536 ± 0.1547
70.00	1.6071 ± 0.1267	1.5338 ± 0.1059	1.3244 ± 0.1086	1.9273 ± 0.1140	1.8652 ± 0.1159
60.00	1.5399 ± 0.0867	1.4823 ± 0.0934	1.2585 ± 0.0913	1.8131 ± 0.1059	1.8284 ± 0.1039
50.00	1.5629 ± 0.0970	1.4028 ± 0.0864	1.1910 ± 0.0889	1.7315 ± 0.0886	1.7346 ± 0.0800
40.00	1.4706 ± 0.1132	1.2905 ± 0.0739	1.0636 ± 0.0705	1.6217 ± 0.1015	1.5808 ± 0.0957
30.00	1.4267 ± 0.0648	1.1576 ± 0.0469	0.9882 ± 0.0600	1.4700 ± 0.0911	1.4424 ± 0.0728
20.00	1.1849 ± 0.0407	0.9991 ± 0.0378	0.7454 ± 0.0368	1.1431 ± 0.0526	1.0641 ± 0.0408
15.00	1.0214 ± 0.0348	0.7467 ± 0.0322	0.6021 ± 0.0353	1.0436 ± 0.0360	0.9029 ± 0.0370
10.00	0.7061 ± 0.0309	0.5920 ± 0.0282	0.4711 ± 0.0301	0.6090 ± 0.0326	0.5893 ± 0.0373
5.00	0.4098 ± 0.0266	0.2619 ± 0.0166	0.2201 ± 0.0214	0.3992 ± 0.0254	0.3114 ± 0.0190
1.00	0.0575 ± 0.0078	0.0725 ± 0.0095	0.0577 ± 0.0108	0.0496 ± 0.0127	0.0663 ± 0.0141

Table S12 Number of hydrogen bonds between sucrose and water molecules for selected groups in this sugar (systems with pre-heating). Calculations are done per one group in a single molecule of sugar.

Water wt %	O5	O7	O1	O2-H8	O3-H9	O4-H10
99.95	0.1432 ± 0.2526	0.6400 ± 0.3484	0.3497 ± 0.3355	1.9256 ± 0.4095	1.9178 ± 0.3824	2.1622 ± 0.4491
99.44	0.1419 ± 0.3543	0.6259 ± 0.4975	0.3286 ± 0.4732	1.9599 ± 0.5497	1.9153 ± 0.5370	2.1582 ± 0.6328
99.00	0.2446 ± 0.3060	0.6640 ± 0.3781	0.2087 ± 0.2769	1.9269 ± 0.4106	1.8652 ± 0.4115	2.0448 ± 0.5207
95.00	0.1274 ± 0.1128	0.6133 ± 0.1666	0.3120 ± 0.1538	1.8055 ± 0.2119	1.8341 ± 0.2028	1.9644 ± 0.2686
90.00	0.1148 ± 0.0755	0.5437 ± 0.1238	0.2876 ± 0.1089	1.6722 ± 0.1728	1.6250 ± 0.1757	1.7295 ± 0.1998
80.00	0.0940 ± 0.0494	0.5019 ± 0.0891	0.2167 ± 0.0684	1.4269 ± 0.1227	1.4480 ± 0.1178	1.6167 ± 0.1259
70.00	0.0969 ± 0.0404	0.4445 ± 0.0619	0.2171 ± 0.0536	1.4108 ± 0.0951	1.4735 ± 0.1140	1.5148 ± 0.1230
60.00	0.0980 ± 0.0347	0.4416 ± 0.0573	0.2299 ± 0.0476	1.2505 ± 0.1083	1.3051 ± 0.0798	1.4576 ± 0.0837
50.00	0.0770 ± 0.0278	0.4321 ± 0.0459	0.1936 ± 0.0365	1.0884 ± 0.0698	1.2632 ± 0.0739	1.3514 ± 0.0752
40.00	0.0872 ± 0.0260	0.4106 ± 0.0392	0.1967 ± 0.0313	1.1662 ± 0.0662	1.1387 ± 0.0622	1.2764 ± 0.0567
30.00	0.0819 ± 0.0235	0.3495 ± 0.0330	0.1796 ± 0.0256	0.9662 ± 0.0453	1.0231 ± 0.0459	1.0939 ± 0.0469
20.00	0.0602 ± 0.0177	0.3065 ± 0.0272	0.1586 ± 0.0217	0.7337 ± 0.0355	0.8460 ± 0.0384	0.9345 ± 0.0375
15.00	0.0495 ± 0.0153	0.2482 ± 0.0219	0.1105 ± 0.0189	0.6277 ± 0.0323	0.6396 ± 0.0277	0.7937 ± 0.0330
10.00	0.0461 ± 0.0145	0.1875 ± 0.0181	0.0988 ± 0.0134	0.5109 ± 0.0249	0.4630 ± 0.0235	0.4979 ± 0.0239
5.00	0.0268 ± 0.0105	0.0859 ± 0.0115	0.0655 ± 0.0116	0.3306 ± 0.0192	0.2753 ± 0.0214	0.3232 ± 0.0181
1.00	0.0178 ± 0.0048	0.0206 ± 0.0082	0.0136 ± 0.0038	0.0476 ± 0.0073	0.0660 ± 0.0084	0.0663 ± 0.0094

Table S13 Number of hydrogen bonds between sucrose and water molecules for selected groups in this sugar (systems with pre-heating). Calculations are done per one group in a single molecule of sugar.

Water wt %	O8-H19	O9-H20	O10-H21	O11-H22	O6-H11
99.95	2.4272 ± 0.4272	1.9878 ± 0.3782	1.7846 ± 0.4778	2.5346 ± 0.4453	2.4090 ± 0.4586
99.44	2.4295 ± 0.6035	2.0439 ± 0.4894	1.7577 ± 0.6847	2.5994 ± 0.6040	2.4530 ± 0.6202
99.00	2.0167 ± 0.5018	1.9259 ± 0.3877	1.7563 ± 0.4572	2.4432 ± 0.4981	2.3927 ± 0.4543
95.00	2.1949 ± 0.2610	1.9240 ± 0.1969	1.6767 ± 0.2479	2.4264 ± 0.2278	2.3433 ± 0.2295
90.00	2.0151 ± 0.1866	1.8241 ± 0.1456	1.5641 ± 0.1861	2.2113 ± 0.2021	2.1694 ± 0.2073
80.00	1.7752 ± 0.1419	1.6189 ± 0.1121	1.3589 ± 0.1299	1.9768 ± 0.1612	1.9342 ± 0.1463
70.00	1.6052 ± 0.1054	1.4817 ± 0.1049	1.2169 ± 0.0939	1.7978 ± 0.1606	1.8079 ± 0.1212
60.00	1.5122 ± 0.1027	1.4259 ± 0.0780	1.1499 ± 0.0859	1.7021 ± 0.1028	1.6144 ± 0.0952
50.00	1.4009 ± 0.0866	1.3540 ± 0.0612	1.0592 ± 0.0679	1.7052 ± 0.0751	1.5917 ± 0.0998
40.00	1.2410 ± 0.0614	1.2180 ± 0.0603	0.9887 ± 0.0540	1.5407 ± 0.0631	1.5505 ± 0.0777
30.00	1.1601 ± 0.0467	1.0594 ± 0.0434	0.9579 ± 0.0471	1.3702 ± 0.0505	1.3085 ± 0.0504
20.00	0.9087 ± 0.0328	0.8743 ± 0.0362	0.7340 ± 0.0438	1.0310 ± 0.0409	1.0153 ± 0.0415
15.00	0.8171 ± 0.0323	0.7404 ± 0.0311	0.6092 ± 0.0337	0.9039 ± 0.0366	0.8978 ± 0.0366
10.00	0.5773 ± 0.0235	0.5815 ± 0.0220	0.4631 ± 0.0297	0.6484 ± 0.0296	0.6790 ± 0.0288
5.00	0.3077 ± 0.0179	0.3076 ± 0.0147	0.1896 ± 0.0192	0.3541 ± 0.0190	0.3988 ± 0.0197
1.00	0.0852 ± 0.0082	0.0600 ± 0.0093	0.0463 ± 0.0082	0.1094 ± 0.0083	0.0752 ± 0.0095

Table S14 Number of hydrogen bonds between trehalose and water molecules for selected groups in this sugar (systems without pre-heating). Calculations are done per one group in a single molecule of sugar.

Water wt %	O1/O7	O6	O5-H11/O8-H9	O4-H10/O9-H20	O3-H9/O10-H21	O2-H8/O11-H22
99.95	0.7185 ± 0.3424	0.3434 ± 0.3672	2.1060 ± 0.3318	2.0591 ± 0.2692	2.0157 ± 0.2688	2.3530 ± 0.3459
99.44	0.7338 ± 0.4853	0.3536 ± 0.5213	2.1106 ± 0.4548	2.0662 ± 0.3765	2.0241 ± 0.3736	2.3928 ± 0.4456
99.00	0.6965 ± 0.3491	0.3227 ± 0.3597	2.0037 ± 0.3729	2.0133 ± 0.2862	2.0048 ± 0.2697	2.3448 ± 0.3556
95.00	0.5453 ± 0.1632	0.2425 ± 0.1552	1.7220 ± 0.2338	1.7701 ± 0.1990	1.8238 ± 0.1664	2.0730 ± 0.1904
90.00	0.5838 ± 0.1255	0.2445 ± 0.1154	1.6365 ± 0.2092	1.7414 ± 0.1443	1.8226 ± 0.1094	2.1022 ± 0.1373
80.00	0.5094 ± 0.0793	0.2021 ± 0.0691	1.4213 ± 0.0958	1.5433 ± 0.0849	1.6433 ± 0.0811	1.8770 ± 0.0984
70.00	0.5198 ± 0.0669	0.2248 ± 0.0606	1.4497 ± 0.1112	1.5293 ± 0.0868	1.5453 ± 0.0902	1.7961 ± 0.0890
60.00	0.5431 ± 0.0650	0.2481 ± 0.0543	1.4808 ± 0.0968	1.4310 ± 0.0684	1.4793 ± 0.0871	1.7477 ± 0.0789
50.00	0.5379 ± 0.0561	0.2463 ± 0.0480	1.4006 ± 0.0947	1.4459 ± 0.0592	1.4026 ± 0.0480	1.6385 ± 0.0643
40.00	0.5491 ± 0.0432	0.2300 ± 0.0423	1.3314 ± 0.0620	1.3381 ± 0.0620	1.3152 ± 0.0571	1.5808 ± 0.0529
30.00	0.5182 ± 0.0388	0.2184 ± 0.0357	1.2032 ± 0.0362	1.2042 ± 0.0369	1.1727 ± 0.0338	1.3942 ± 0.0364
20.00	0.3463 ± 0.0305	0.1659 ± 0.0279	0.9635 ± 0.0301	0.9458 ± 0.0289	0.8980 ± 0.0275	1.1208 ± 0.0447
15.00	0.2916 ± 0.0320	0.1423 ± 0.0261	0.7445 ± 0.0281	0.7868 ± 0.0235	0.7133 ± 0.0226	0.8579 ± 0.0246
10.00	0.2027 ± 0.0226	0.0981 ± 0.0227	0.5198 ± 0.0246	0.5301 ± 0.0215	0.5528 ± 0.0174	0.5924 ± 0.0228
5.00	0.0794 ± 0.0123	0.0427 ± 0.0132	0.2949 ± 0.0195	0.3228 ± 0.0173	0.2746 ± 0.0135	0.3531 ± 0.0208
1.00	0.0184 ± 0.0068	0.0064 ± 0.0056	0.0670 ± 0.0087	0.0805 ± 0.0128	0.0660 ± 0.0069	0.0568 ± 0.0072

Table S15 Number of hydrogen bonds between trehalose and water molecules for selected groups in this sugar (systems with pre-heating). Calculations are done per one group in a single molecule of sugar.

Water wt %	O1/O7	O6	O5-H11/O8-H9	O4-H10/O9-H20	O3-H9/O10-H21	O2-H8/O11-H22
99.95	0.7212 ± 0.3406	0.3510 ± 0.3694	2.1181 ± 0.3194	2.0572 ± 0.2723	2.0227 ± 0.2669	2.3725 ± 0.3174
99.44	0.7466 ± 0.4699	0.3540 ± 0.5258	2.1271 ± 0.4443	2.0627 ± 0.3718	2.0245 ± 0.3723	2.4117 ± 0.4445
99.00	0.7405 ± 0.3410	0.3494 ± 0.3700	2.1154 ± 0.3152	2.0689 ± 0.2678	2.0240 ± 0.2642	2.4000 ± 0.3142
95.00	0.6563 ± 0.1699	0.3078 ± 0.1670	1.9046 ± 0.1930	1.9141 ± 0.1546	1.9156 ± 0.1465	2.2271 ± 0.1757
90.00	0.5787 ± 0.1291	0.2547 ± 0.1141	1.6405 ± 0.2203	1.6940 ± 0.1940	1.7783 ± 0.1389	2.0926 ± 0.1451
80.00	0.4809 ± 0.0747	0.2073 ± 0.0670	1.3789 ± 0.0732	1.3575 ± 0.0754	1.4715 ± 0.0663	1.6698 ± 0.0893
70.00	0.4894 ± 0.0620	0.1952 ± 0.0597	1.2888 ± 0.1080	1.3918 ± 0.0621	1.5023 ± 0.0630	1.6890 ± 0.0932
60.00	0.4500 ± 0.0505	0.1862 ± 0.0445	1.1921 ± 0.0482	1.2669 ± 0.0458	1.4001 ± 0.0521	1.5219 ± 0.0565
50.00	0.4419 ± 0.0437	0.1731 ± 0.0388	1.1792 ± 0.0429	1.2357 ± 0.0409	1.2886 ± 0.0515	1.4859 ± 0.0556
40.00	0.4115 ± 0.0378	0.1563 ± 0.0342	1.0635 ± 0.0345	1.1937 ± 0.0361	1.2220 ± 0.0397	1.4876 ± 0.0464
30.00	0.4199 ± 0.0318	0.1628 ± 0.0309	1.0136 ± 0.0316	1.0537 ± 0.0301	1.0498 ± 0.0302	1.3031 ± 0.0360
20.00	0.3342 ± 0.0245	0.1322 ± 0.0252	0.8242 ± 0.0245	0.8734 ± 0.0230	0.8615 ± 0.0238	1.0270 ± 0.0279
15.00	0.2438 ± 0.0215	0.1087 ± 0.0218	0.5986 ± 0.0216	0.6131 ± 0.0194	0.7419 ± 0.0194	0.8576 ± 0.0270
10.00	0.1935 ± 0.0175	0.0929 ± 0.0187	0.5047 ± 0.0170	0.5161 ± 0.0160	0.5717 ± 0.0157	0.6343 ± 0.0212
5.00	0.0877 ± 0.0123	0.0391 ± 0.0116	0.2455 ± 0.0111	0.2890 ± 0.0124	0.3157 ± 0.0128	0.3524 ± 0.0126
1.00	0.0346 ± 0.0071	0.0161 ± 0.0077	0.0536 ± 0.0061	0.0508 ± 0.0051	0.0733 ± 0.0061	0.0707 ± 0.0064

2 Radial distribution functions (RDFs)

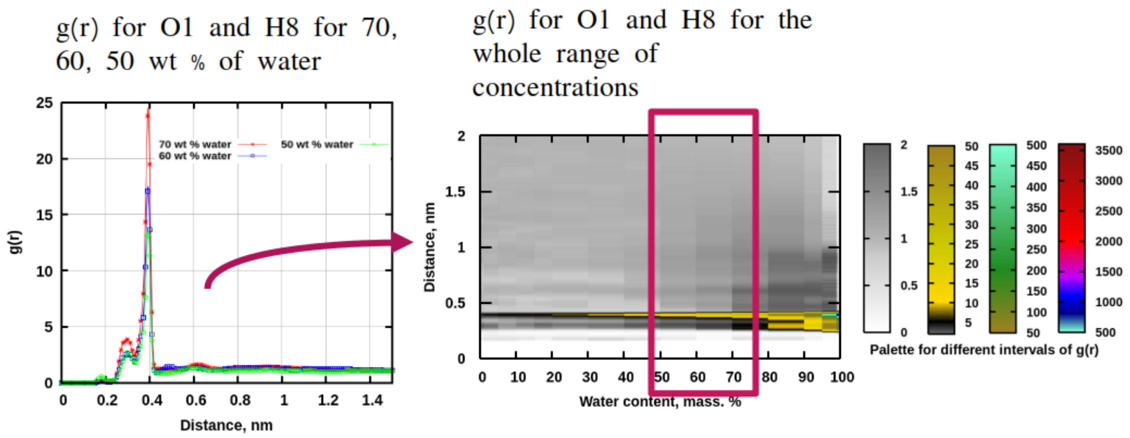

Figure S1 Illustration for radial distribution functions plotted in figures S2-S37

Here you may find the graphical explanation of how radial distribution functions were converted into color-maps.

2.1 Sucrose

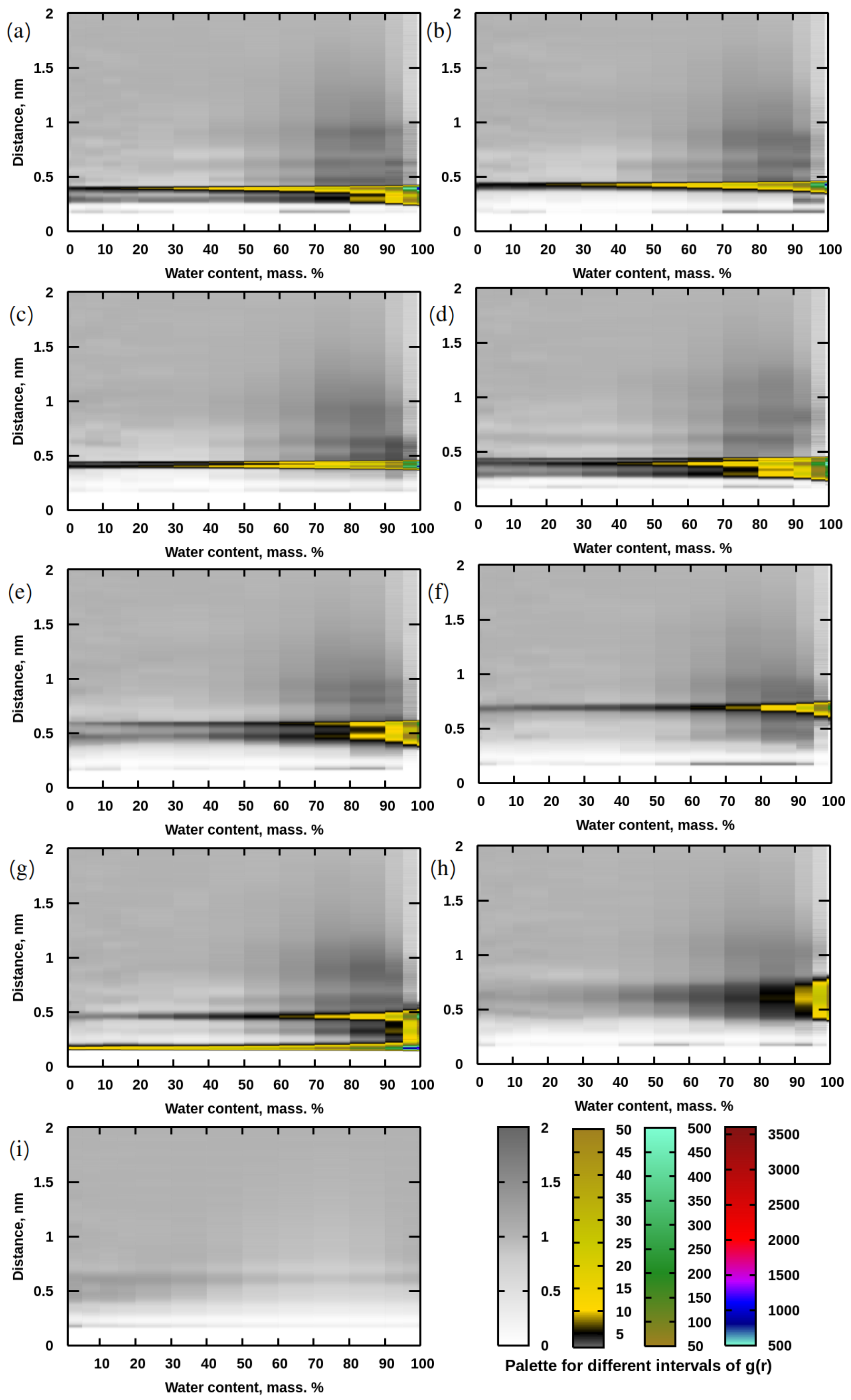

Figure S2 RDFs between oxygen O1 of sucrose and hydrogens in hydroxyl groups of sucrose and water (systems without pre-heating). (a) H8 (b) H9 (c) H10 (d) H11 (e) H19 (f) H20 (g) H21 (h) H22 (i) hydrogens in water.

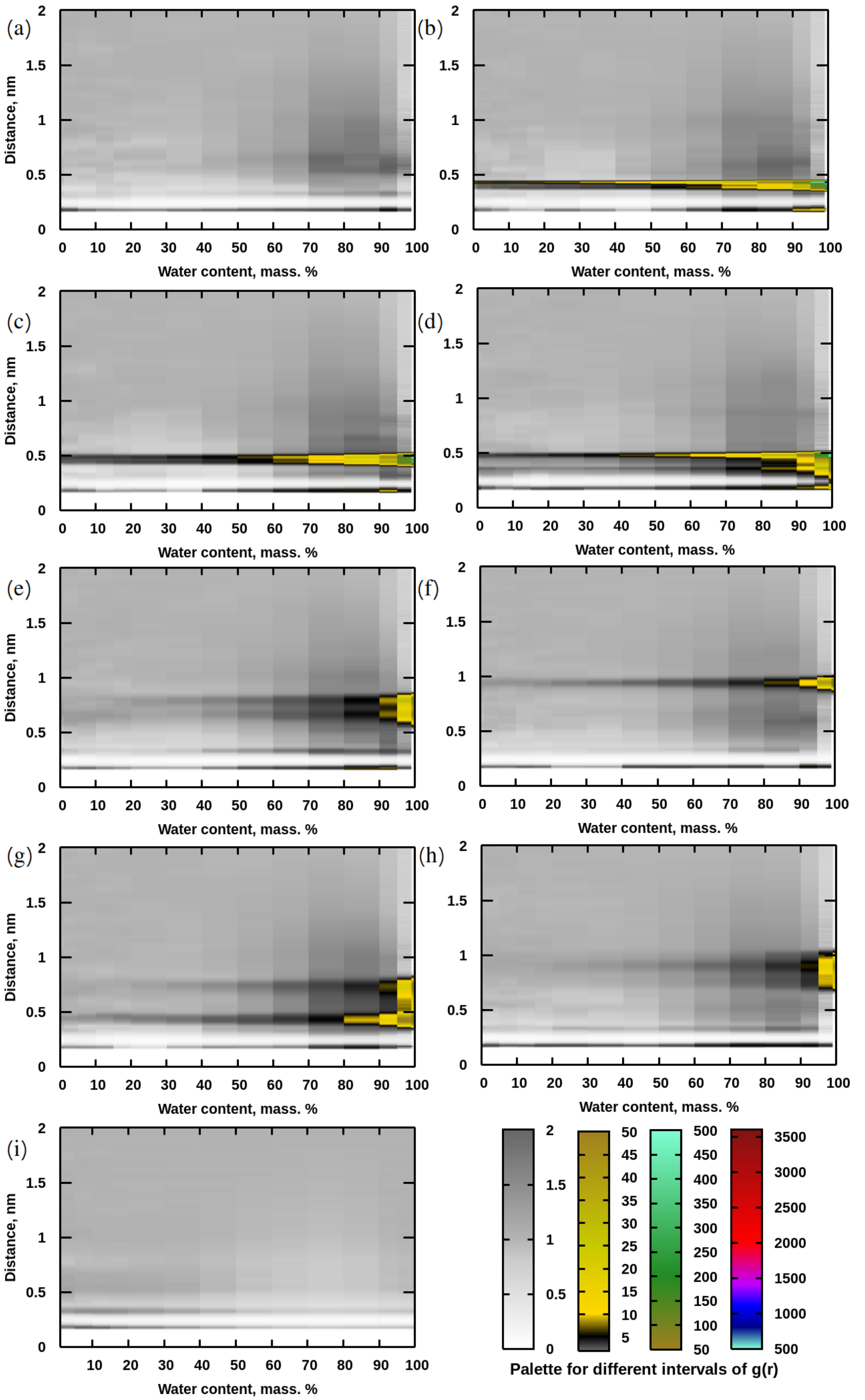

Figure S3 RDFs between oxygen O2 of sucrose and hydrogens in hydroxyl groups of sucrose and water (systems without pre-heating). (a) H8 (b) H9 (c) H10 (d) H11 (e) H19 (f) H20 (g) H21 (h) H22 (i) hydrogens in water.

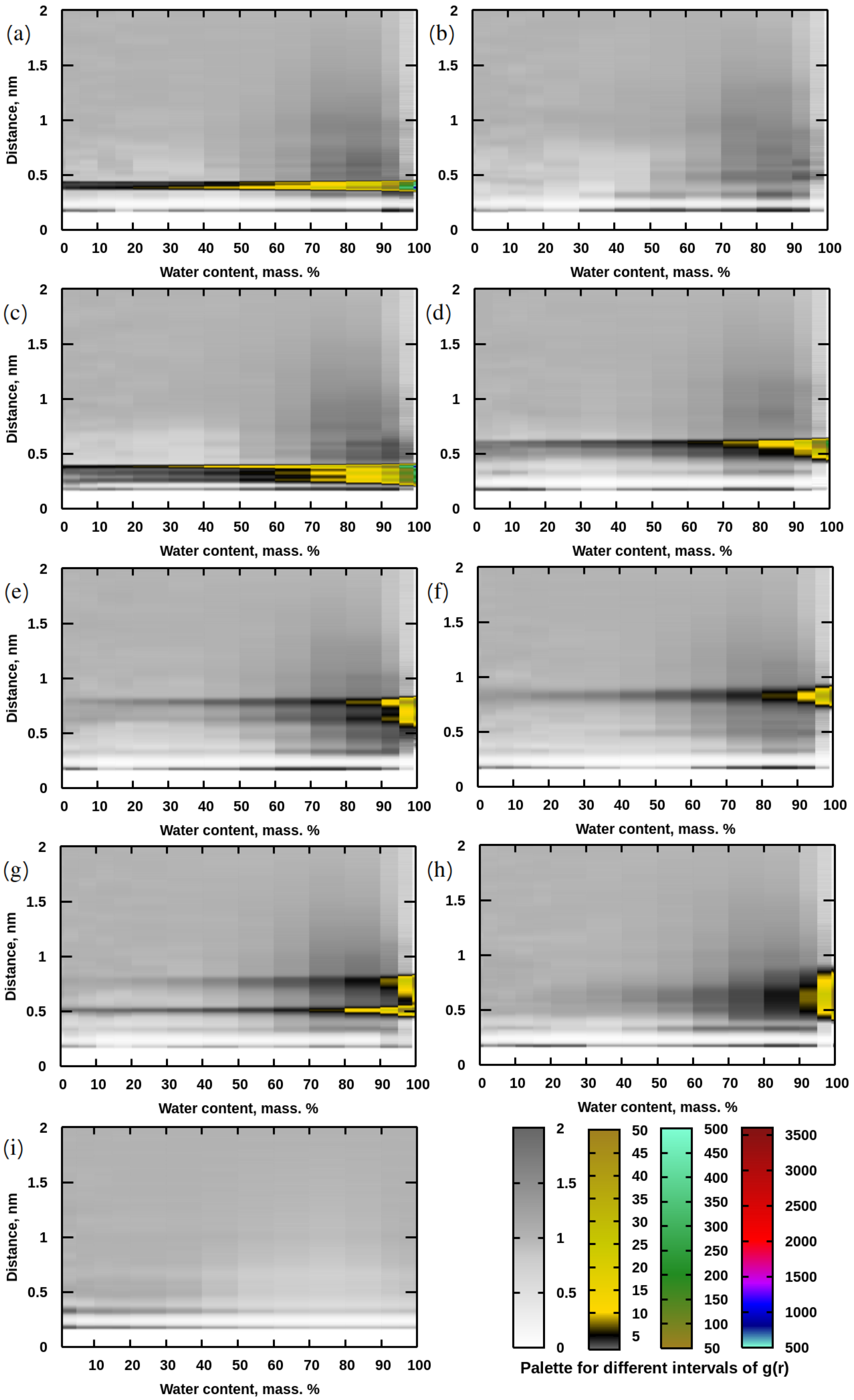

Figure S4 RDFs between oxygen O3 of sucrose and hydrogens in hydroxyl groups of sucrose and water (systems without pre-heating). (a) H8 (b) H9 (c) H10 (d) H11 (e) H19 (f) H20 (g) H21 (h) H22 (i) hydrogens in water.

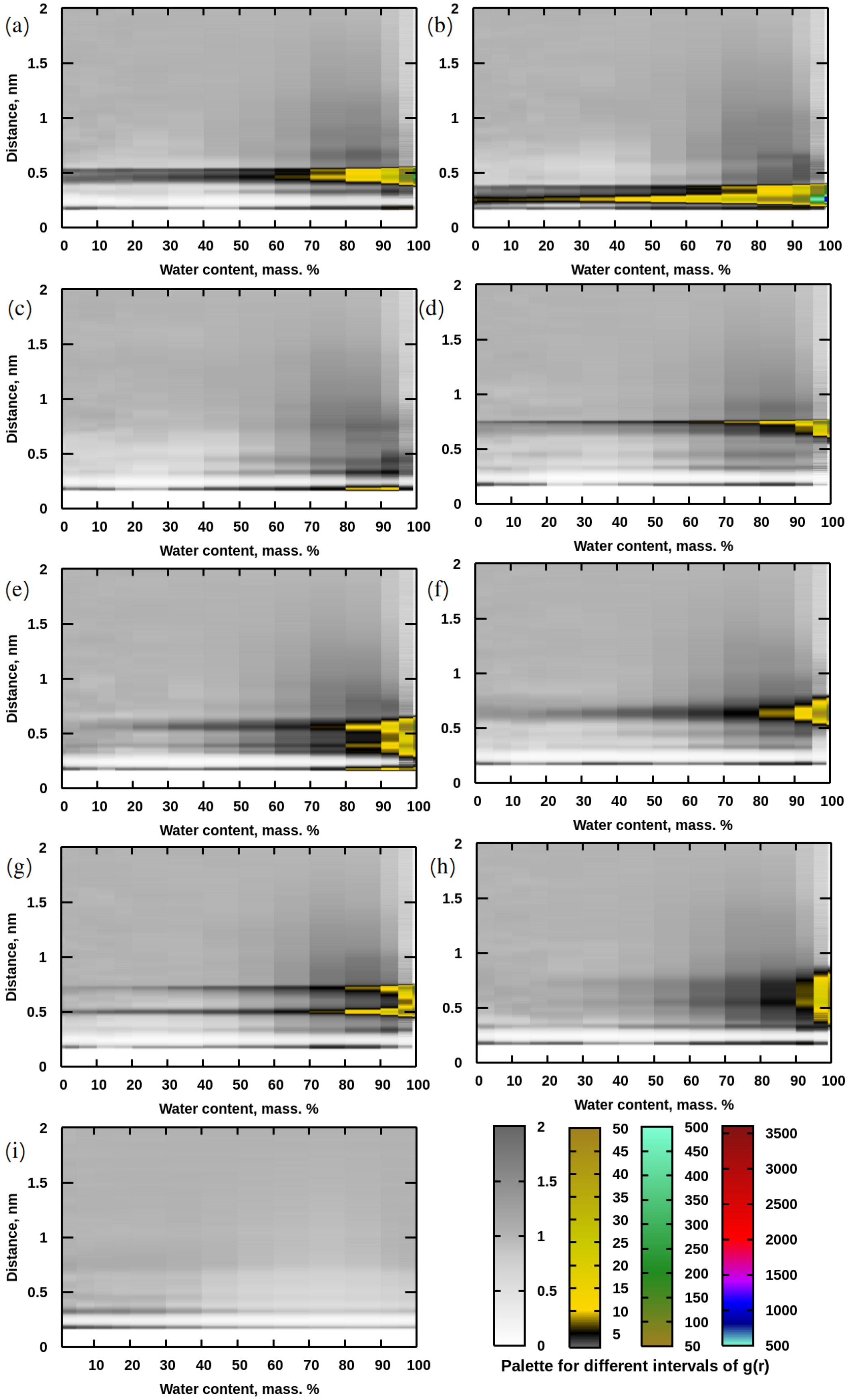

Figure S5 RDFs between oxygen O4 of sucrose and hydrogens in hydroxyl groups of sucrose and water (systems without pre-heating). (a) H8 (b) H9 (c) H10 (d) H11 (e) H19 (f) H20 (g) H21 (h) H22 (i) hydrogens in water.

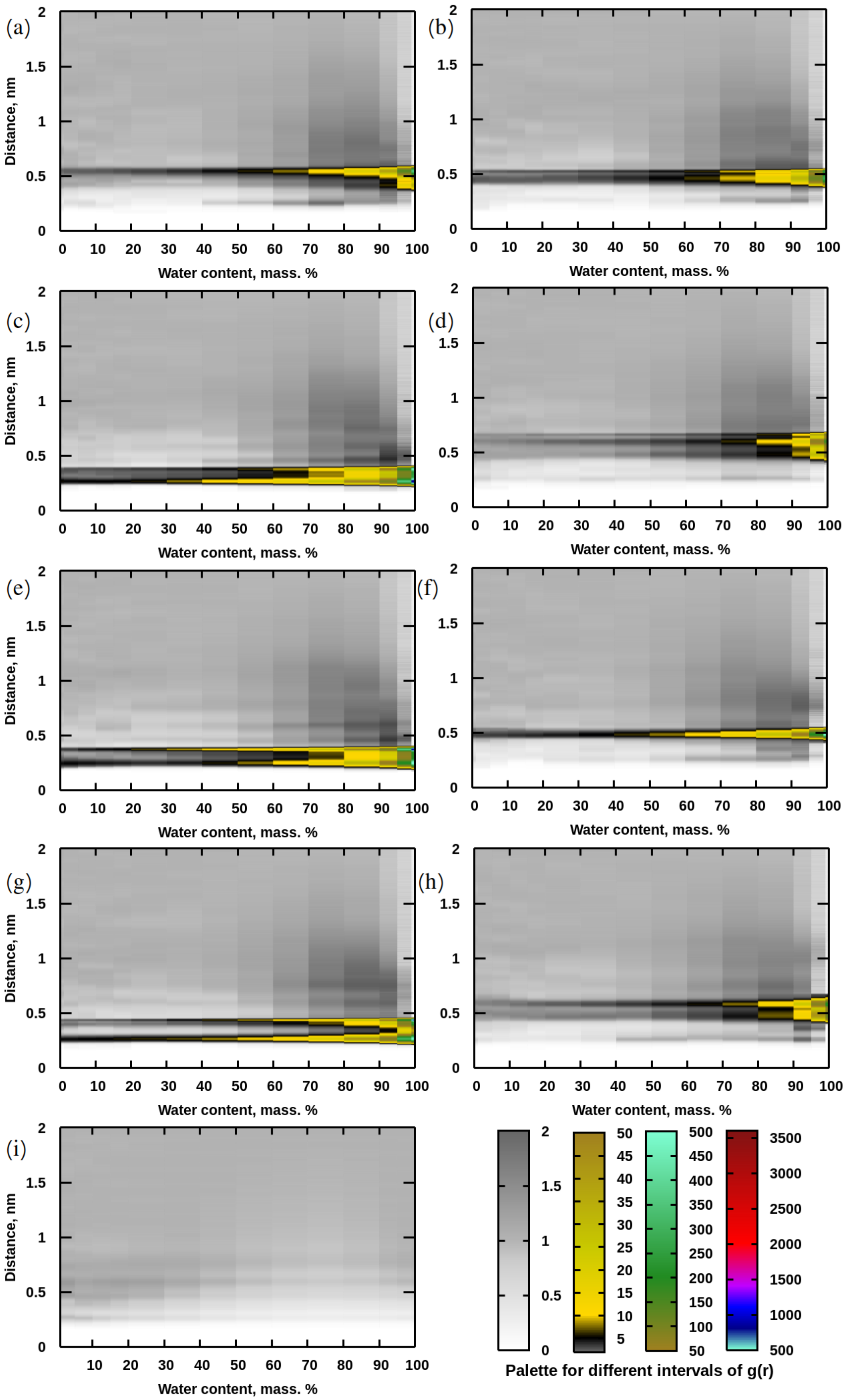

Figure S6 RDFs between oxygen O5 of sucrose and hydrogens in hydroxyl groups of sucrose and water (systems without pre-heating). (a) H8 (b) H9 (c) H10 (d) H11 (e) H19 (f) H20 (g) H21 (h) H22 (i) hydrogens in water.

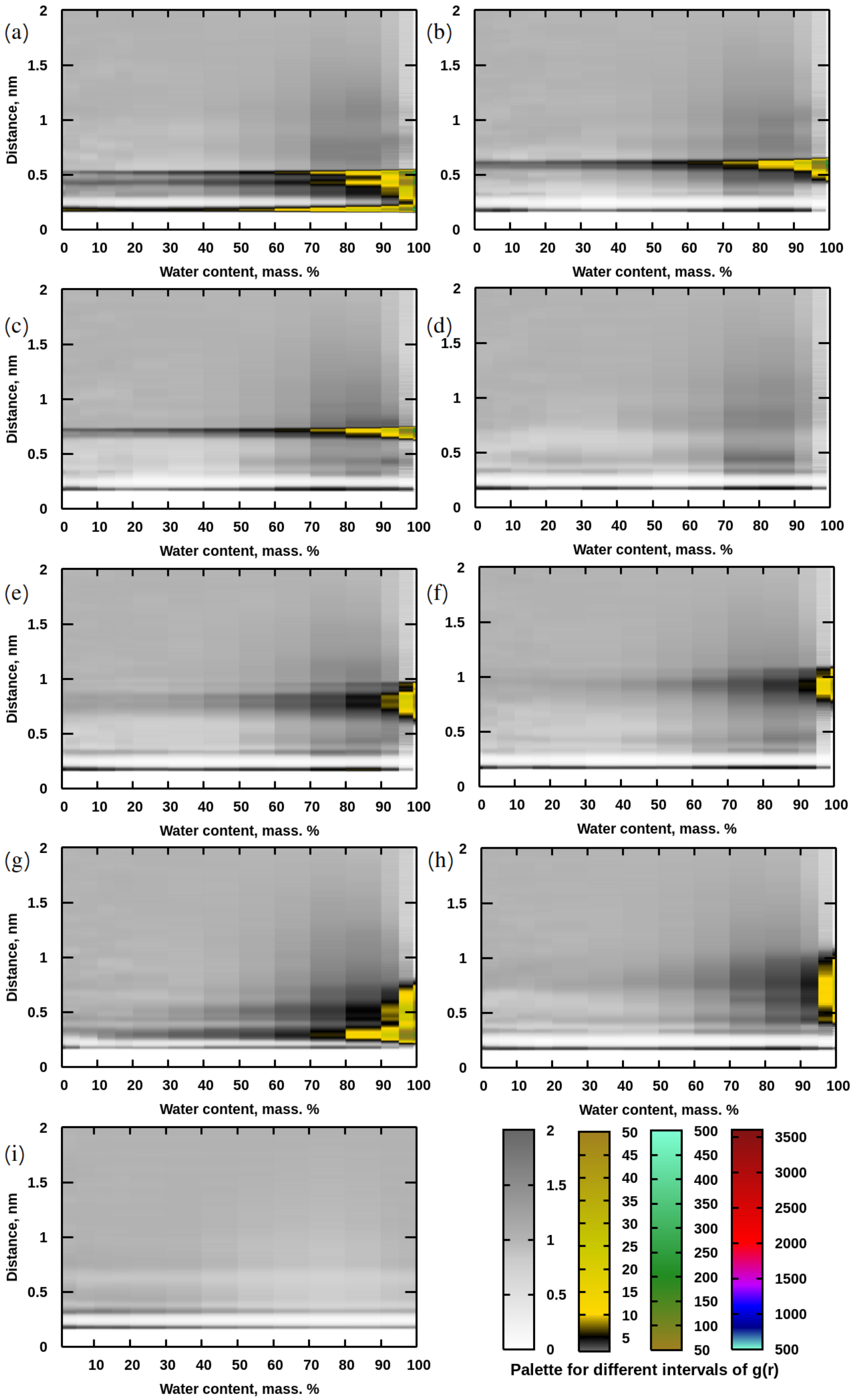

Figure S7 RDFs between oxygen O6 of sucrose and hydrogens in hydroxyl groups of sucrose and water (systems without pre-heating). (a) H8 (b) H9 (c) H10 (d) H11 (e) H19 (f) H20 (g) H21 (h) H22 (i) hydrogens in water.

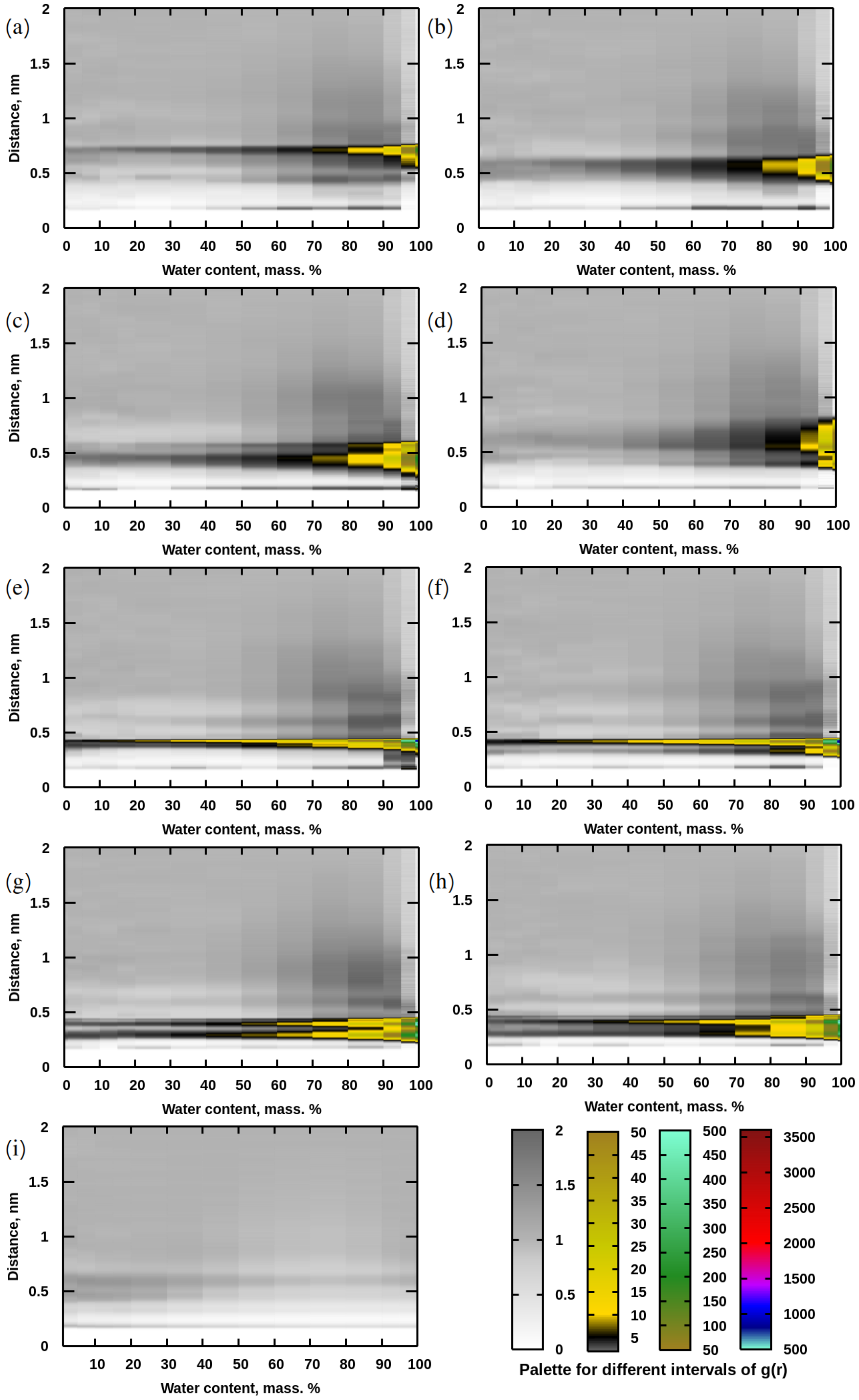

Figure S8 RDFs between oxygen O7 of sucrose and hydrogens in hydroxyl groups of sucrose and water (systems without pre-heating). (a) H8 (b) H9 (c) H10 (d) H11 (e) H19 (f) H20 (g) H21 (h) H22 (i) hydrogens in water.

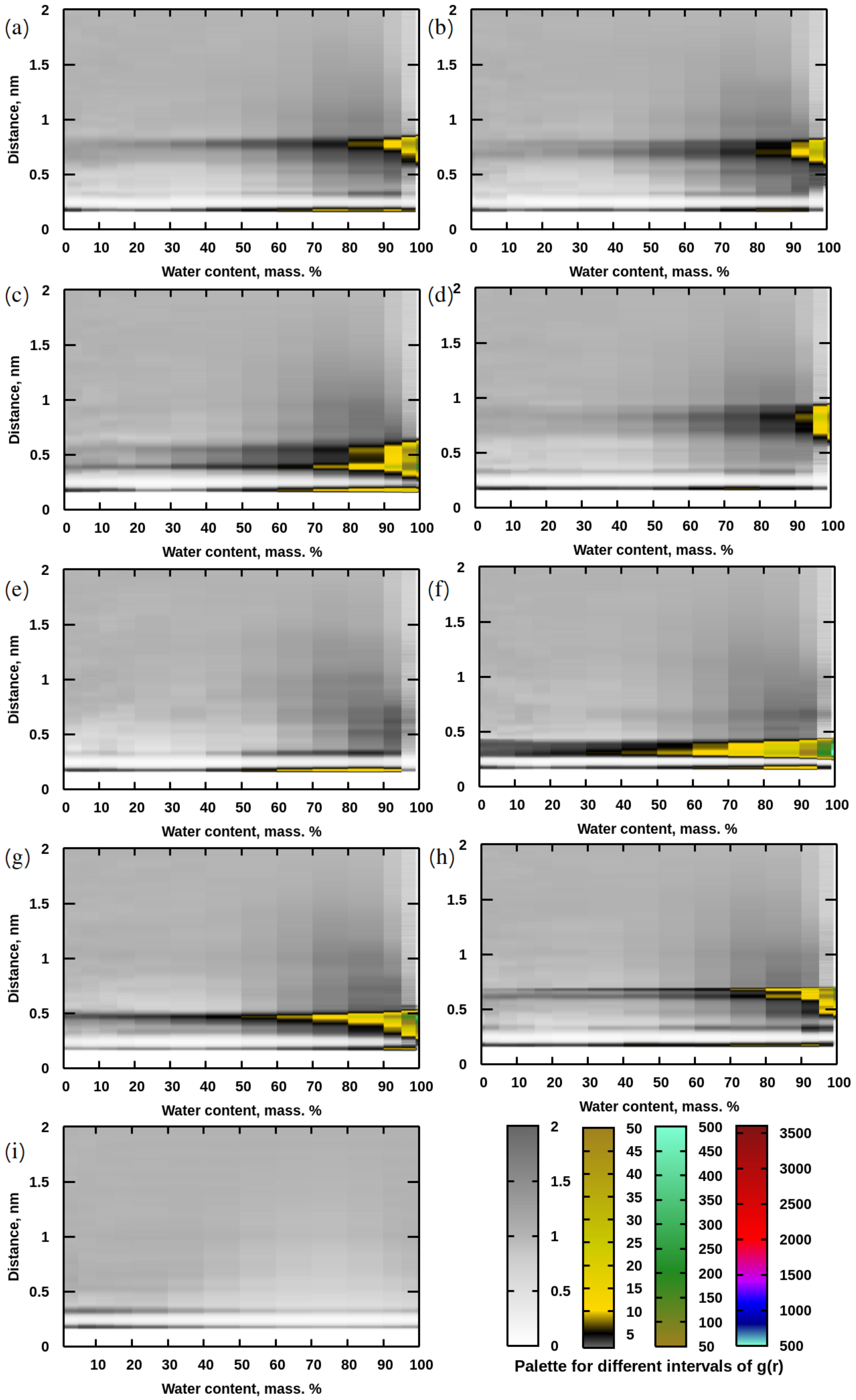

Figure S9 RDFs between oxygen O8 of sucrose and hydrogens in hydroxyl groups of sucrose and water (systems without pre-heating). (a) H8 (b) H9 (c) H10 (d) H11 (e) H19 (f) H20 (g) H21 (h) H22 (i) hydrogens in water.

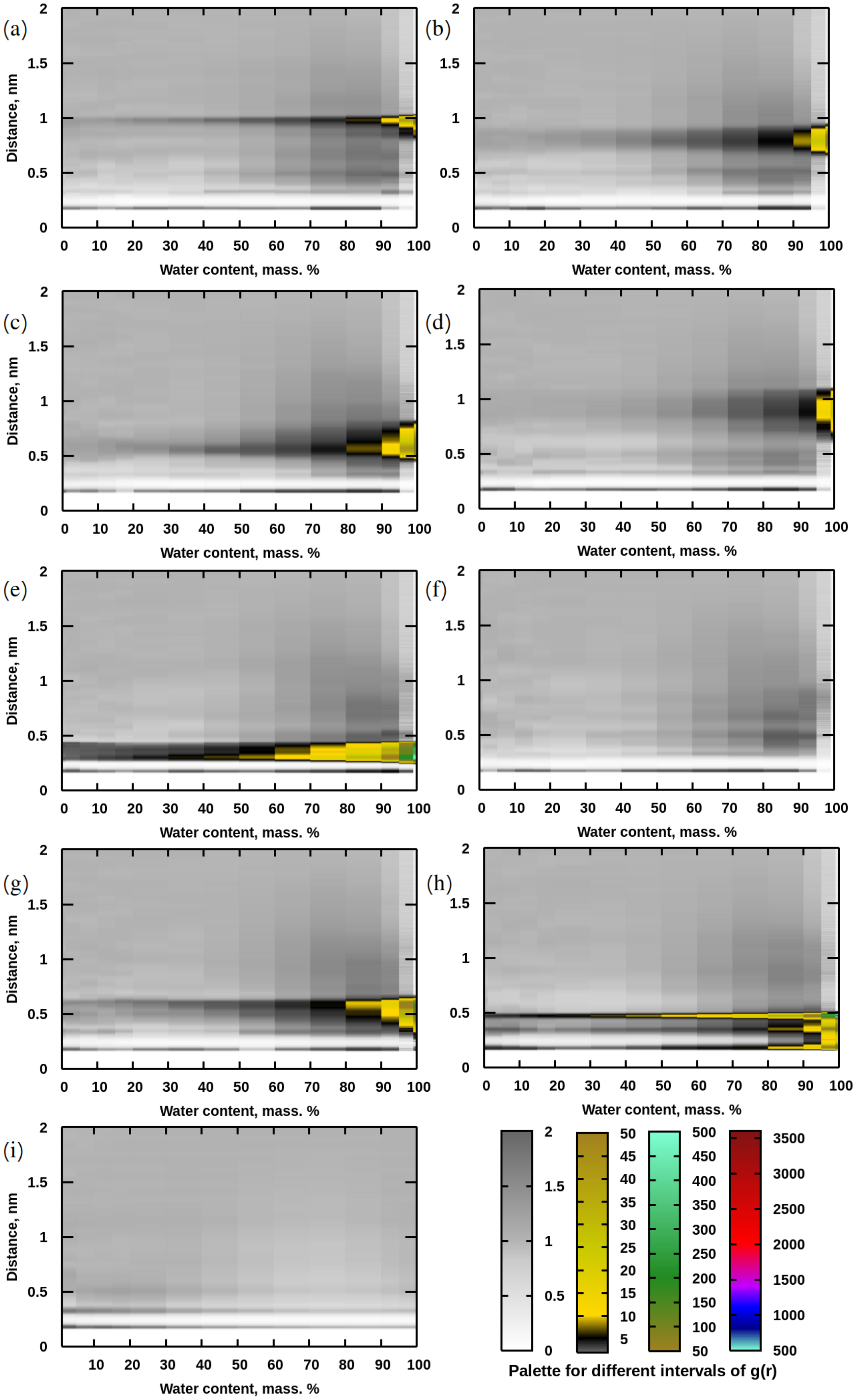

Figure S10 RDFs between oxygen O9 of sucrose and hydrogens in hydroxyl groups of sucrose and water (systems without pre-heating). (a) H8 (b) H9 (c) H10 (d) H11 (e) H19 (f) H20 (g) H21 (h) H22 (i) hydrogens in water.

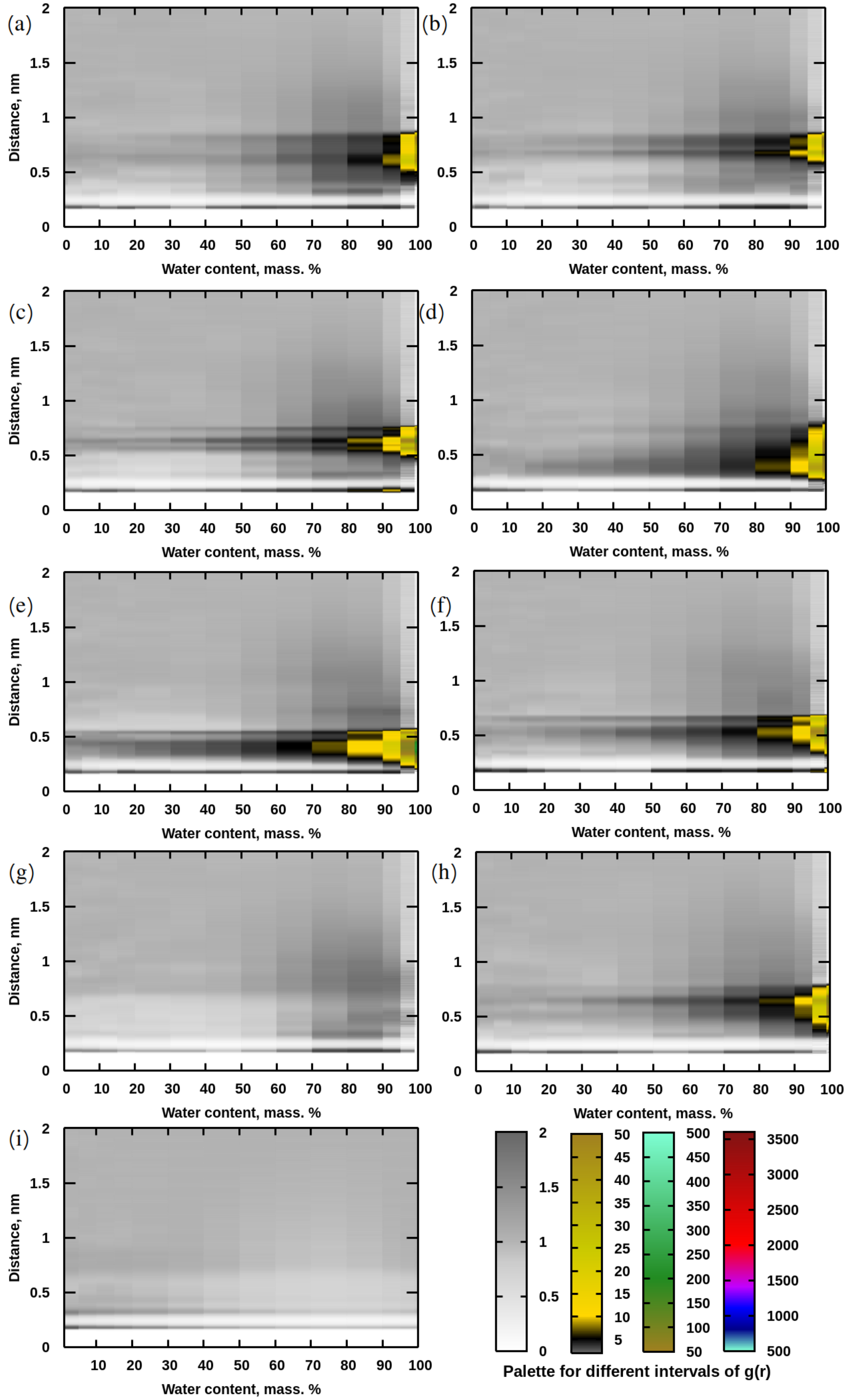

Figure S11 RDFs between oxygen O10 of sucrose and hydrogens in hydroxyl groups of sucrose and water (systems without pre-heating). (a) H8 (b) H9 (c) H10 (d) H11 (e) H19 (f) H20 (g) H21 (h) H22 (i) hydrogens in water.

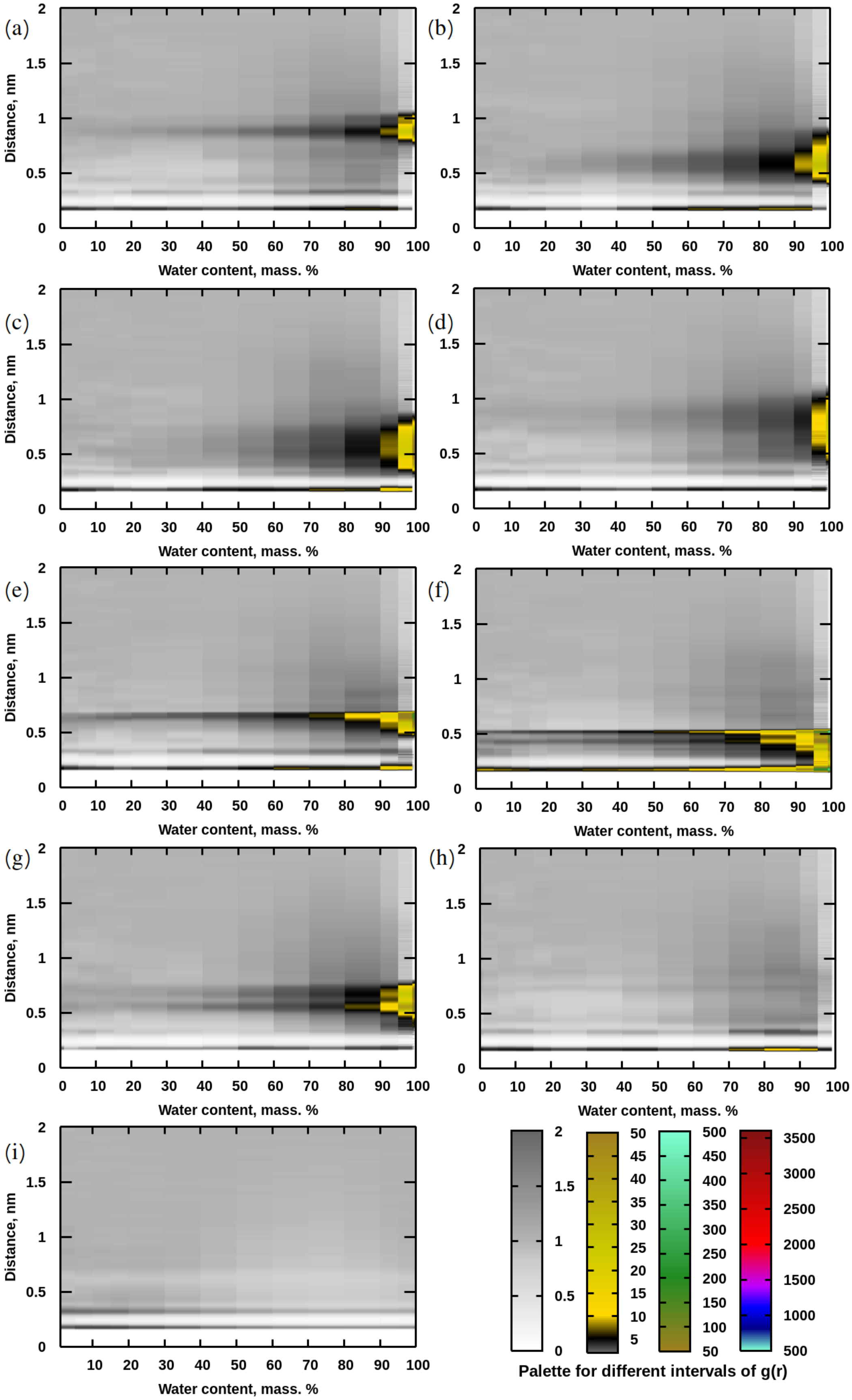

Figure S12 RDFs between oxygen O11 of sucrose and hydrogens in hydroxyl groups of sucrose and water (systems without pre-heating). (a) H8 (b) H9 (c) H10 (d) H11 (e) H19 (f) H20 (g) H21 (h) H22 (i) hydrogens in water.

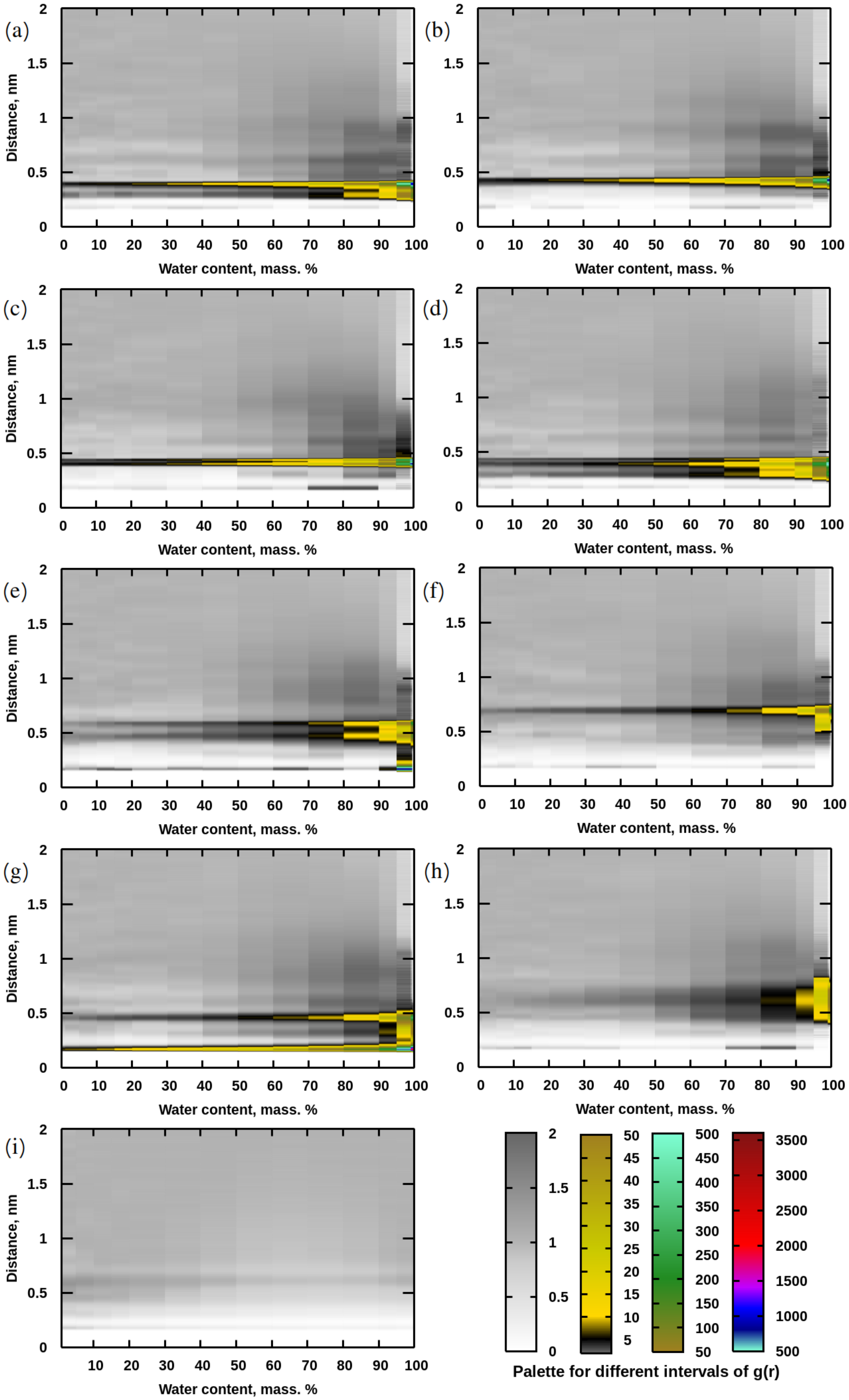

Figure S13 RDFs between oxygen O1 of sucrose and hydrogens in hydroxyl groups of sucrose and water (systems with pre-heating). (a) H8 (b) H9 (c) H10 (d) H11 (e) H19 (f) H20 (g) H21 (h) H22 (i) hydrogens in water.

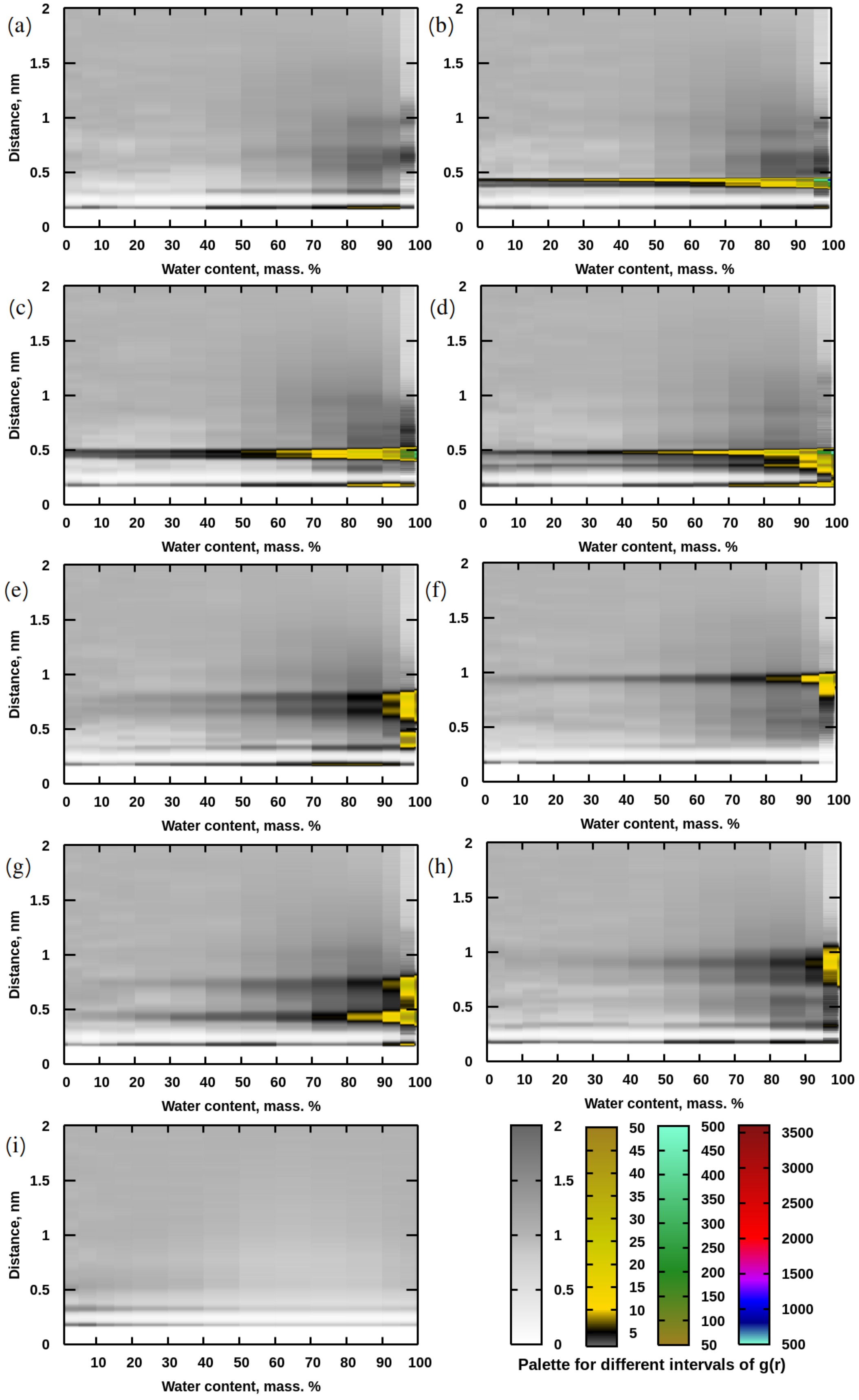

Figure S14 RDFs between oxygen O2 of sucrose and hydrogens in hydroxyl groups of sucrose and water (systems with pre-heating). (a) H8 (b) H9 (c) H10 (d) H11 (e) H19 (f) H20 (g) H21 (h) H22 (i) hydrogens in water.

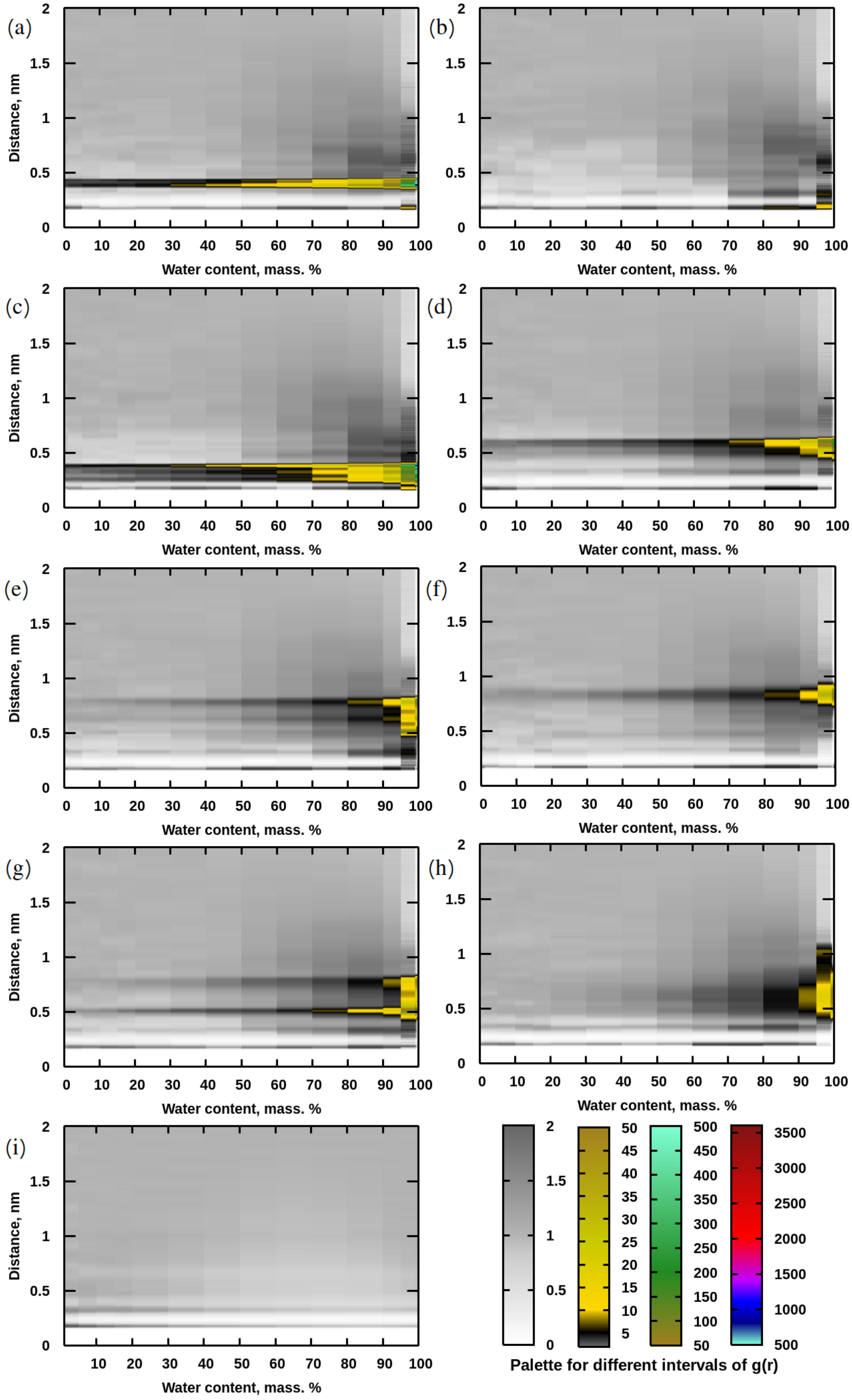

Figure S15 RDFs between oxygen O3 of sucrose and hydrogens in hydroxyl groups of sucrose and water (systems with pre-heating). (a) H8 (b) H9 (c) H10 (d) H11 (e) H19 (f) H20 (g) H21 (h) H22 (i) hydrogens in water.

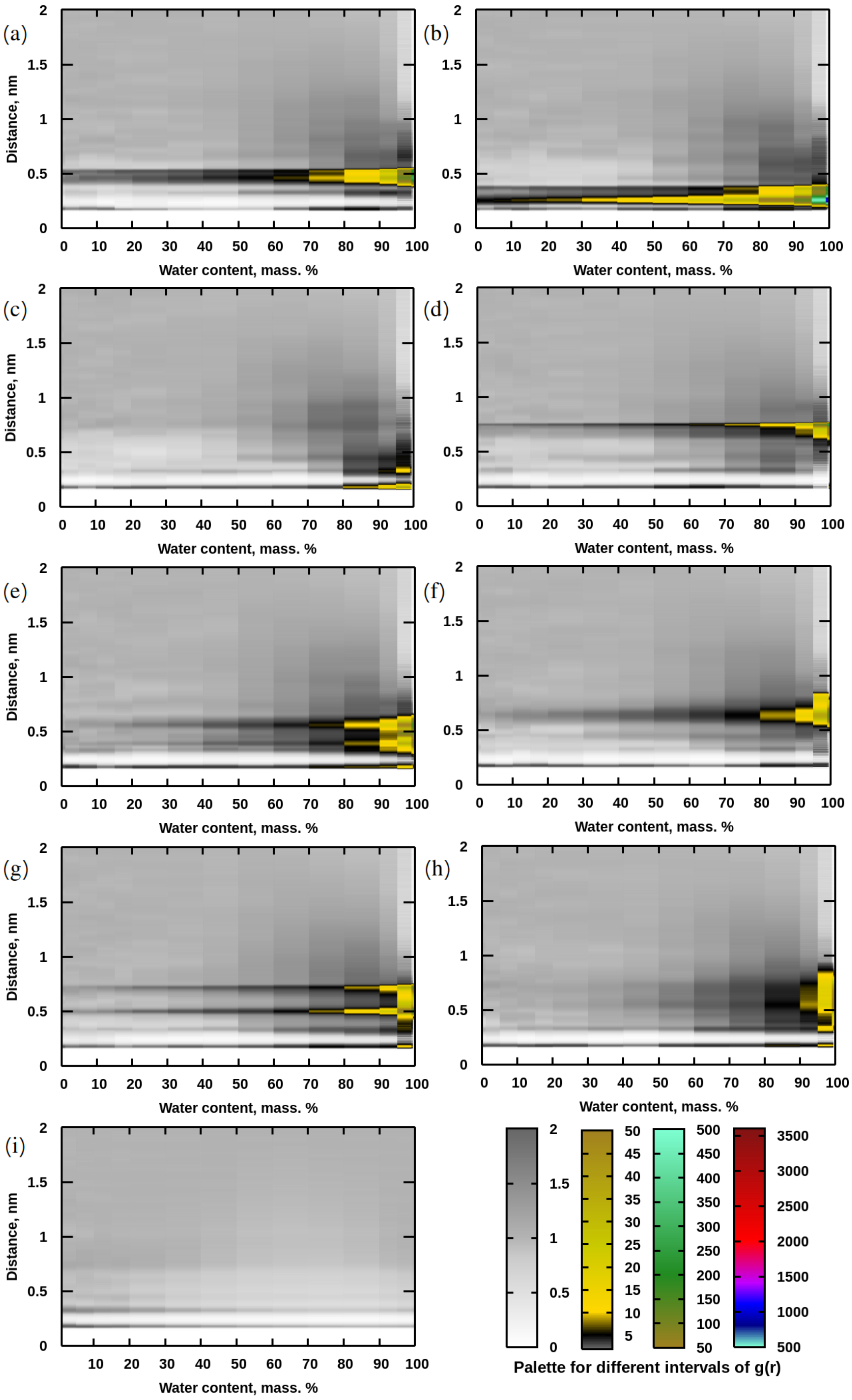

Figure S16 RDFs between oxygen O4 of sucrose and hydrogens in hydroxyl groups of sucrose and water (systems with pre-heating). (a) H8 (b) H9 (c) H10 (d) H11 (e) H19 (f) H20 (g) H21 (h) H22 (i) hydrogens in water.

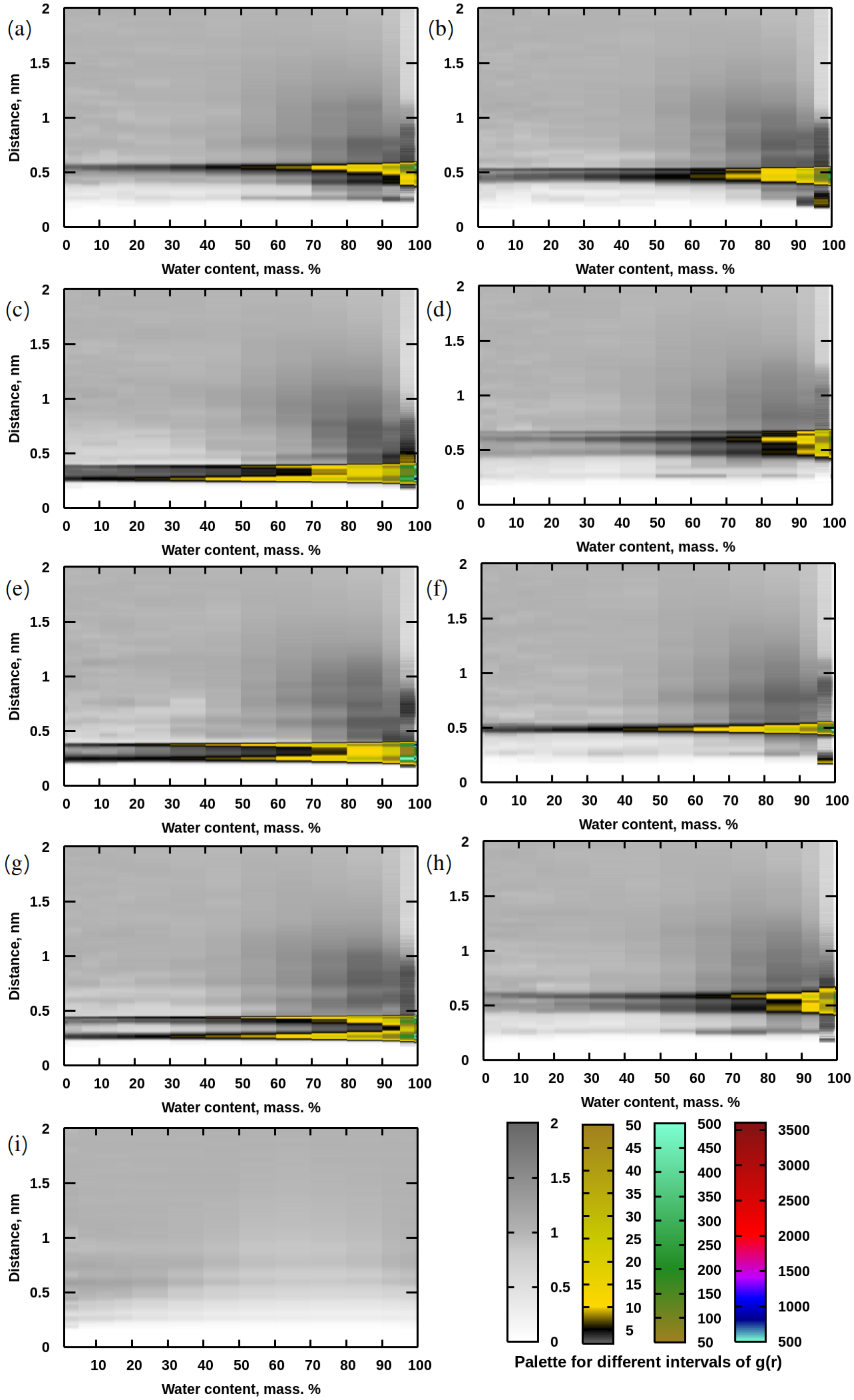

Figure S17 RDFs between oxygen O5 of sucrose and hydrogens in hydroxyl groups of sucrose and water (systems with pre-heating). (a) H8 (b) H9 (c) H10 (d) H11 (e) H19 (f) H20 (g) H21 (h) H22 (i) hydrogens in water.

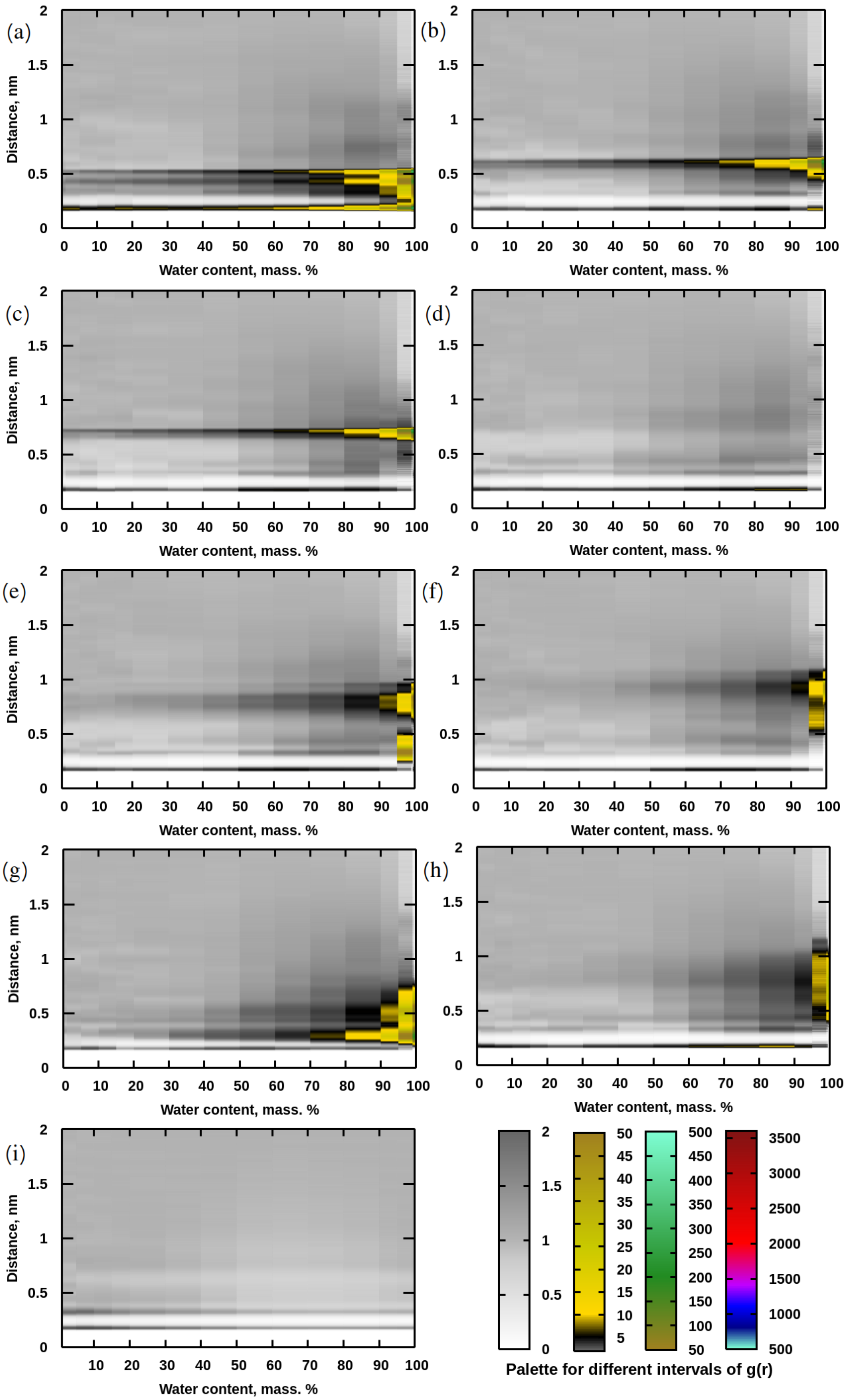

Figure S18 RDFs between oxygen O6 of sucrose and hydrogens in hydroxyl groups of sucrose and water (systems with pre-heating). (a) H8 (b) H9 (c) H10 (d) H11 (e) H19 (f) H20 (g) H21 (h) H22 (i) hydrogens in water.

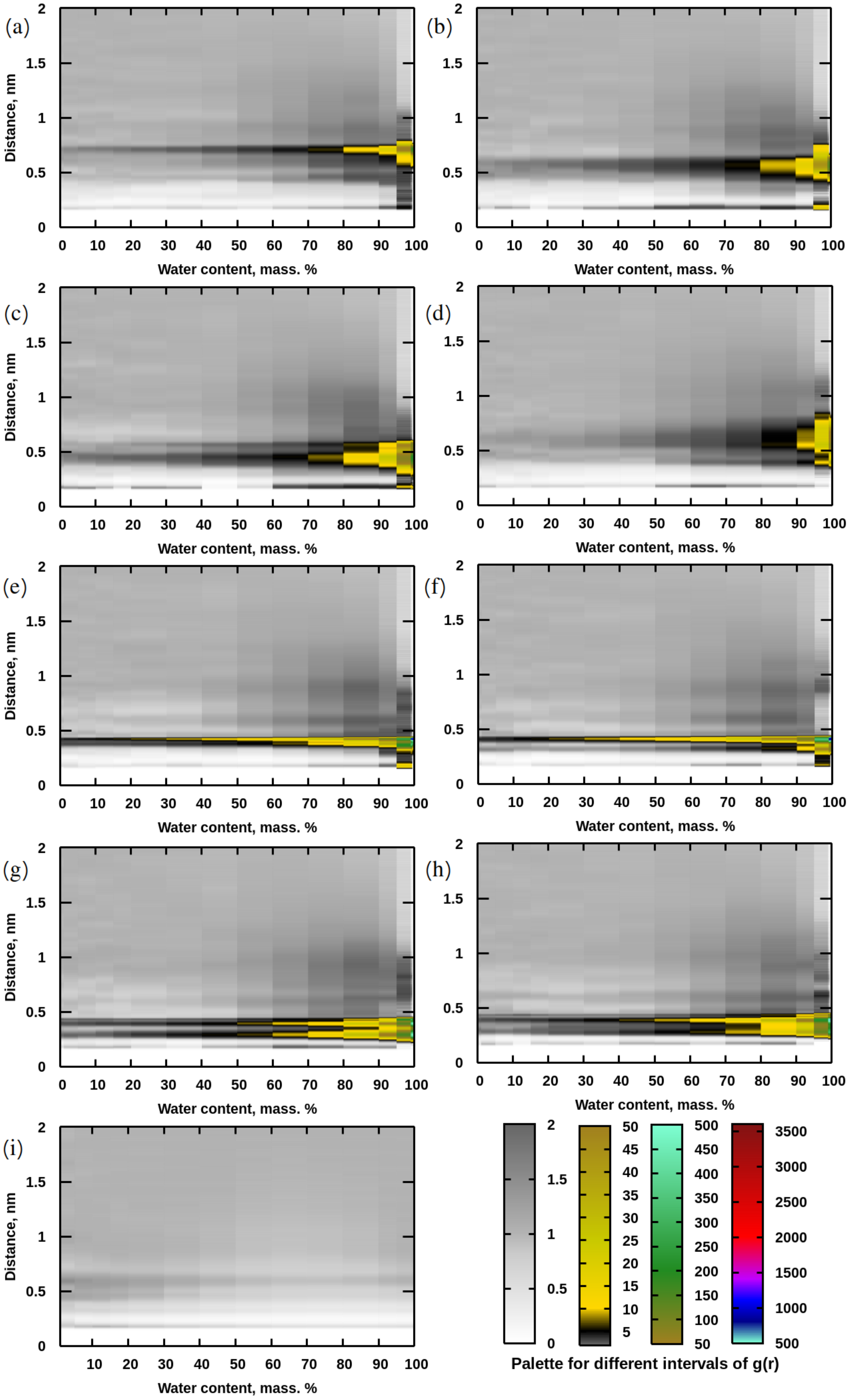

Figure S19 RDFs between oxygen O7 of sucrose and hydrogens in hydroxyl groups of sucrose and water (systems with pre-heating). (a) H8 (b) H9 (c) H10 (d) H11 (e) H19 (f) H20 (g) H21 (h) H22 (i) hydrogens in water.

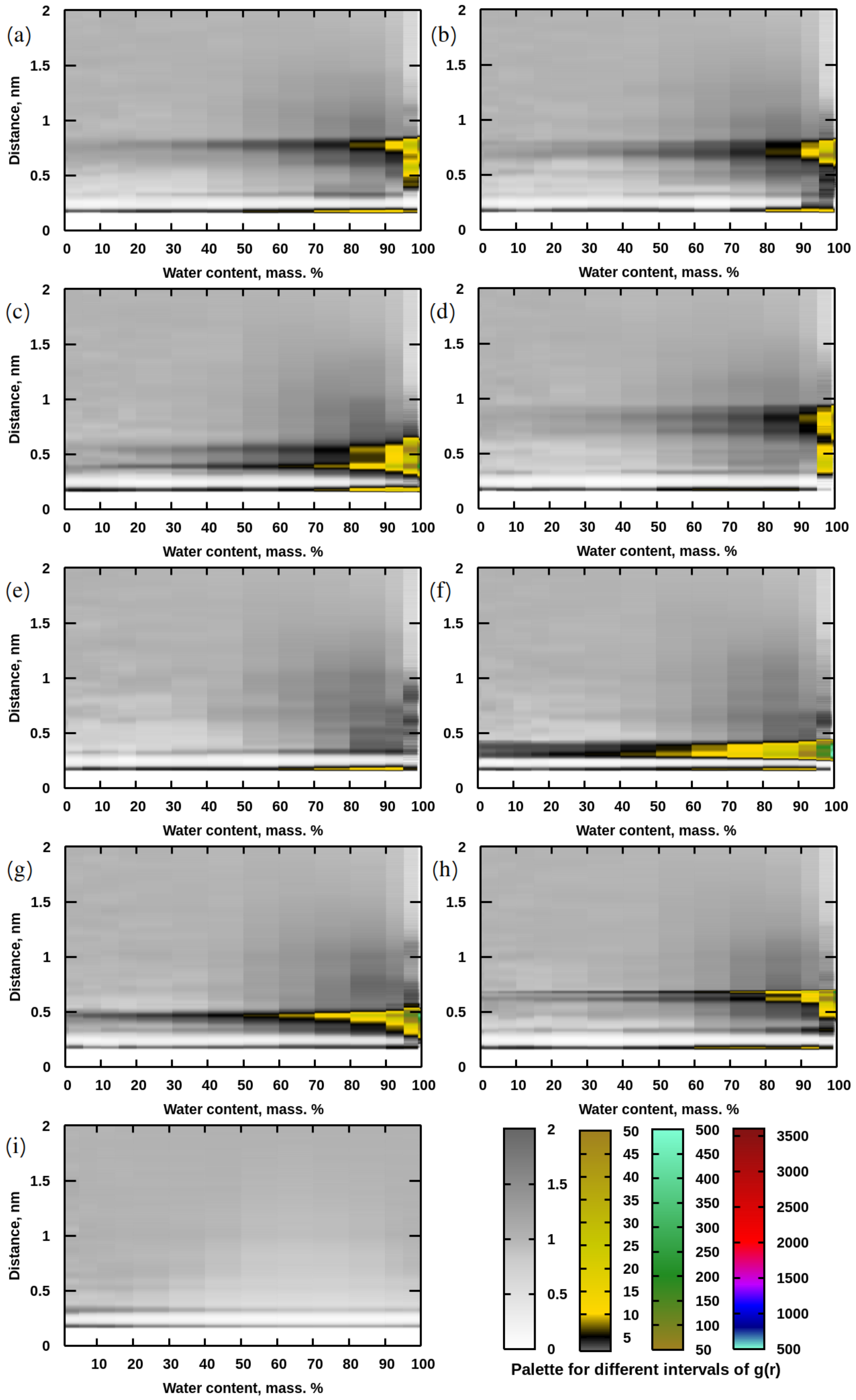

Figure S20 RDFs between oxygen O8 of sucrose and hydrogens in hydroxyl groups of sucrose and water (systems with pre-heating). (a) H8 (b) H9 (c) H10 (d) H11 (e) H19 (f) H20 (g) H21 (h) H22 (i) hydrogens in water.

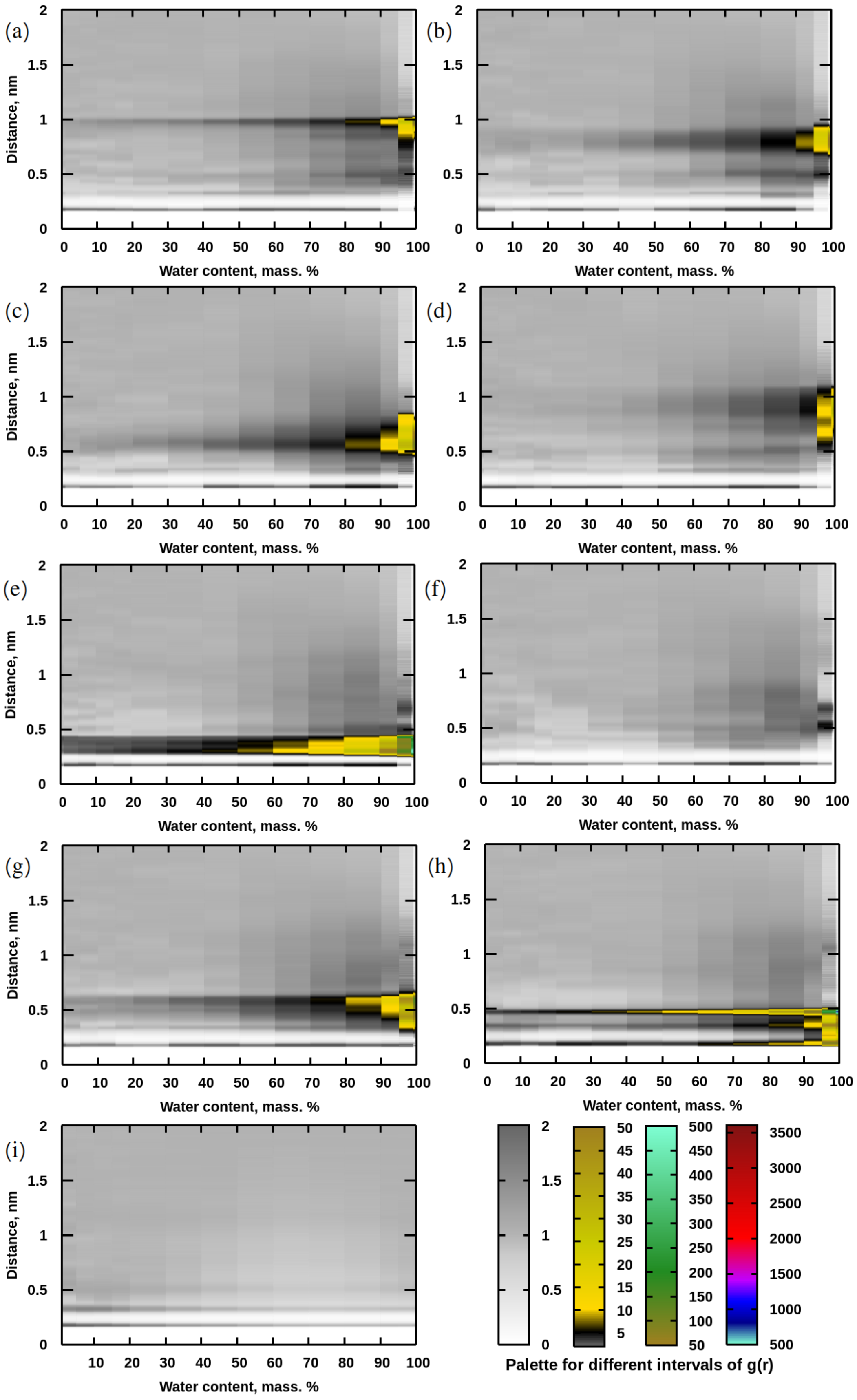

Figure S21 RDFs between oxygen O9 of sucrose and hydrogens in hydroxyl groups of sucrose and water (systems with pre-heating). (a) H8 (b) H9 (c) H10 (d) H11 (e) H19 (f) H20 (g) H21 (h) H22 (i) hydrogens in water.

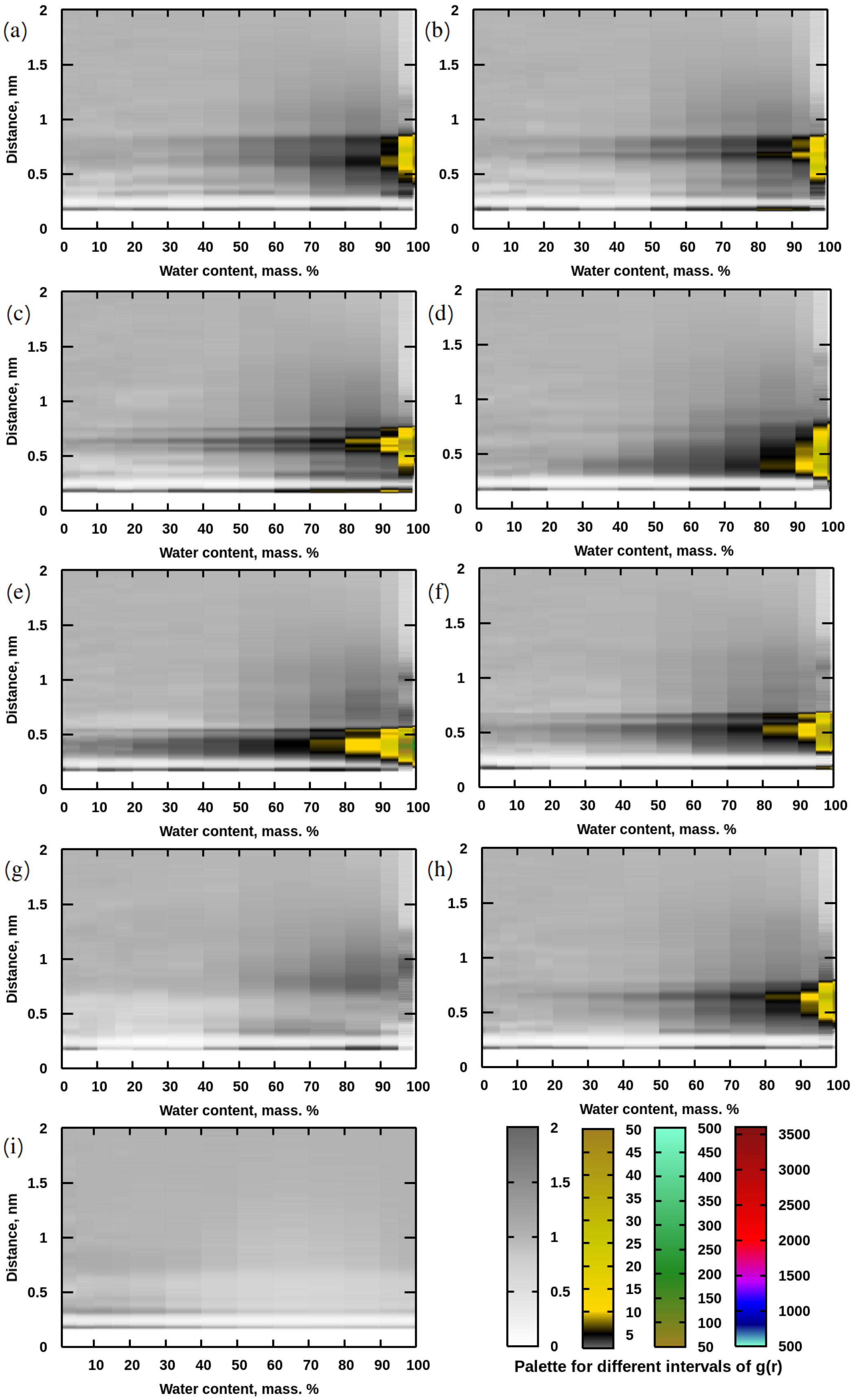

Figure S22 RDFs between oxygen O10 of sucrose and hydrogens in hydroxyl groups of sucrose and water (systems with pre-heating). (a) H8 (b) H9 (c) H10 (d) H11 (e) H19 (f) H20 (g) H21 (h) H22 (i) hydrogens in water.

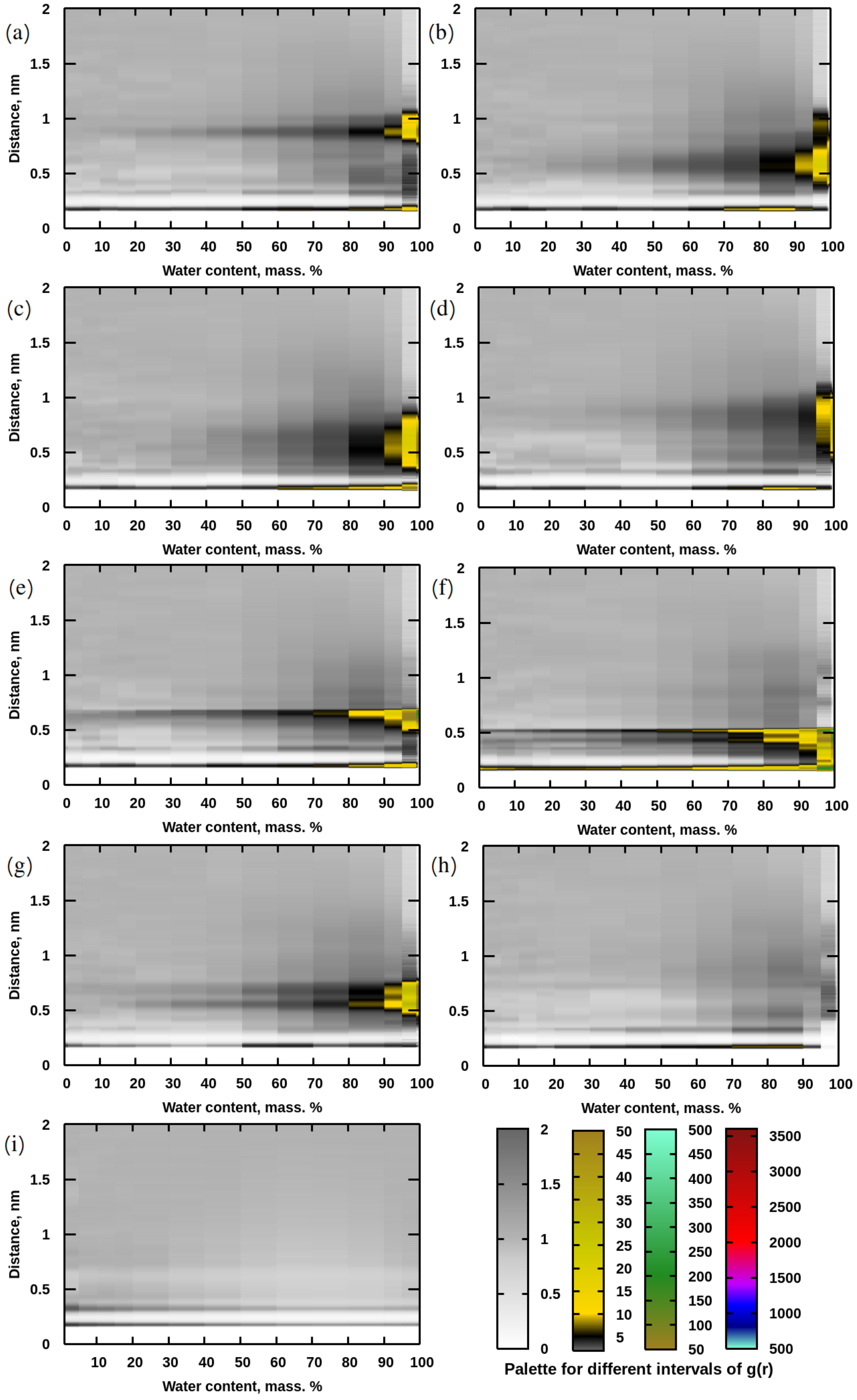

Figure S23 RDFs between oxygen O11 of sucrose and hydrogens in hydroxyl groups of sucrose and water (systems with pre-heating). (a) H8 (b) H9 (c) H10 (d) H11 (e) H19 (f) H20 (g) H21 (h) H22 (i) hydrogens in water.

2.2 Trehalose

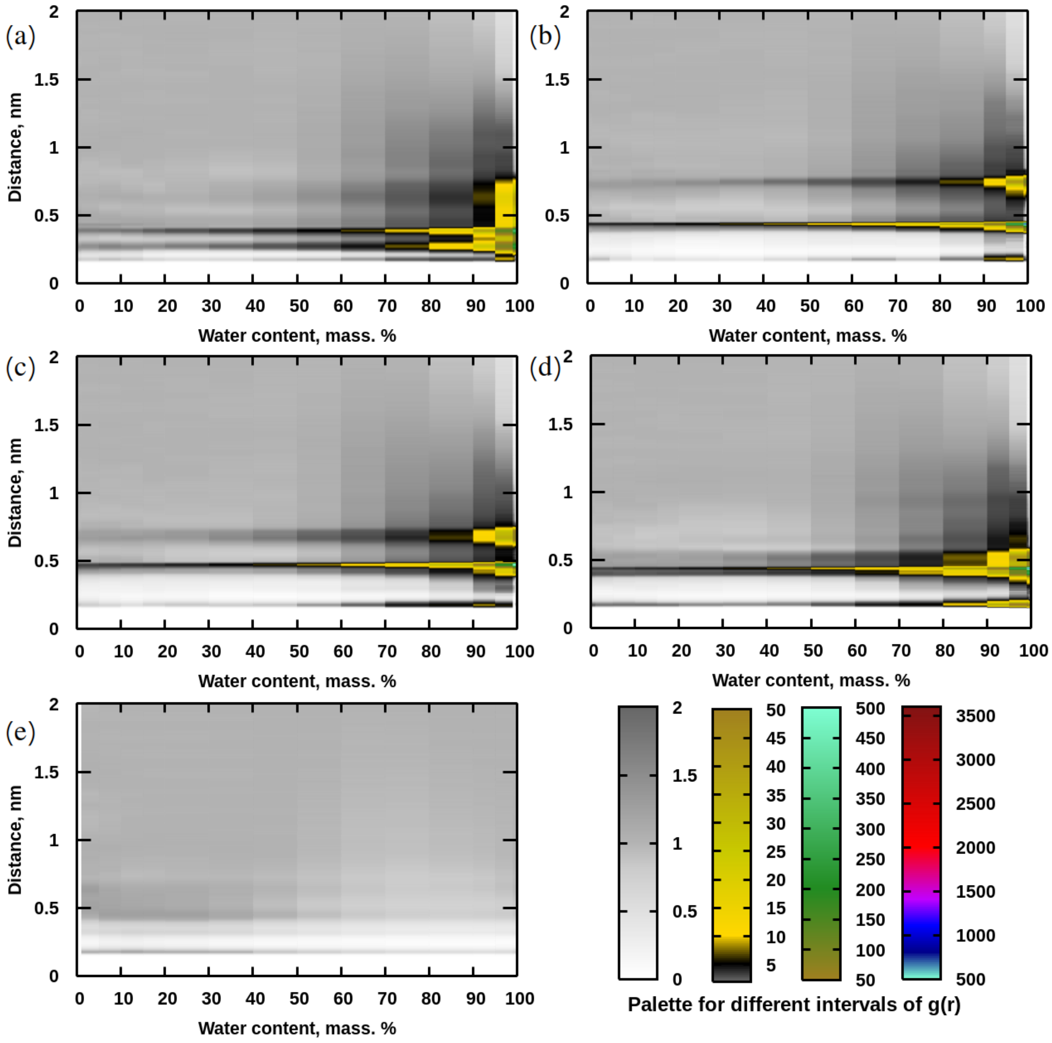

Figure S24 RDFs between oxygens O1/O7 of trehalose and hydrogens in hydroxyl groups of trehalose and water (systems without pre-heating). (a) H8/H22 (b) H9/H21 (c) H10/H20 (d) H11/H19 (e) hydrogens in water.

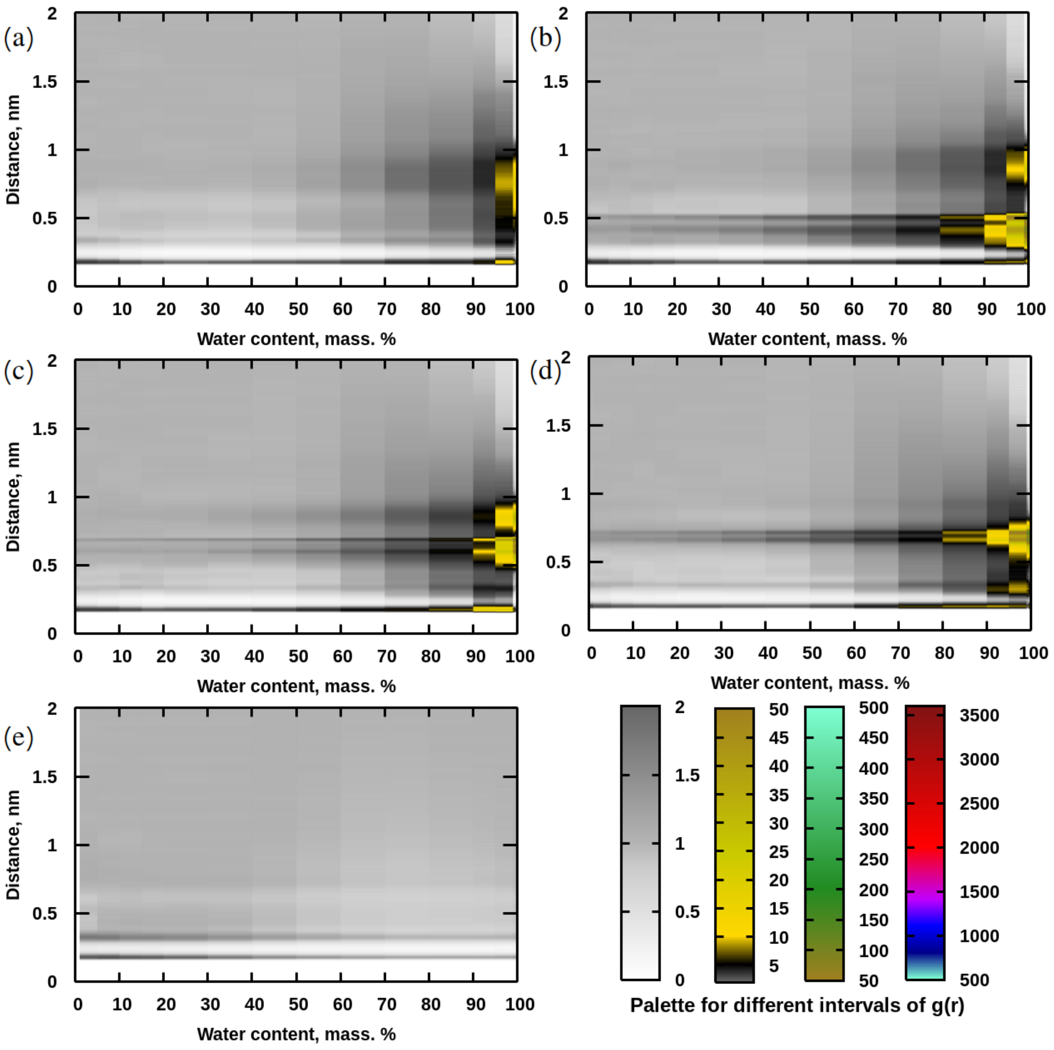

Figure S25 RDFs between oxygens O2/O11 of trehalose and hydrogens in hydroxyl groups of trehalose and water (systems without pre-heating). (a) H8/H22 (b) H9/H21 (c) H10/H20 (d) H11/H19 (e) hydrogens in water.

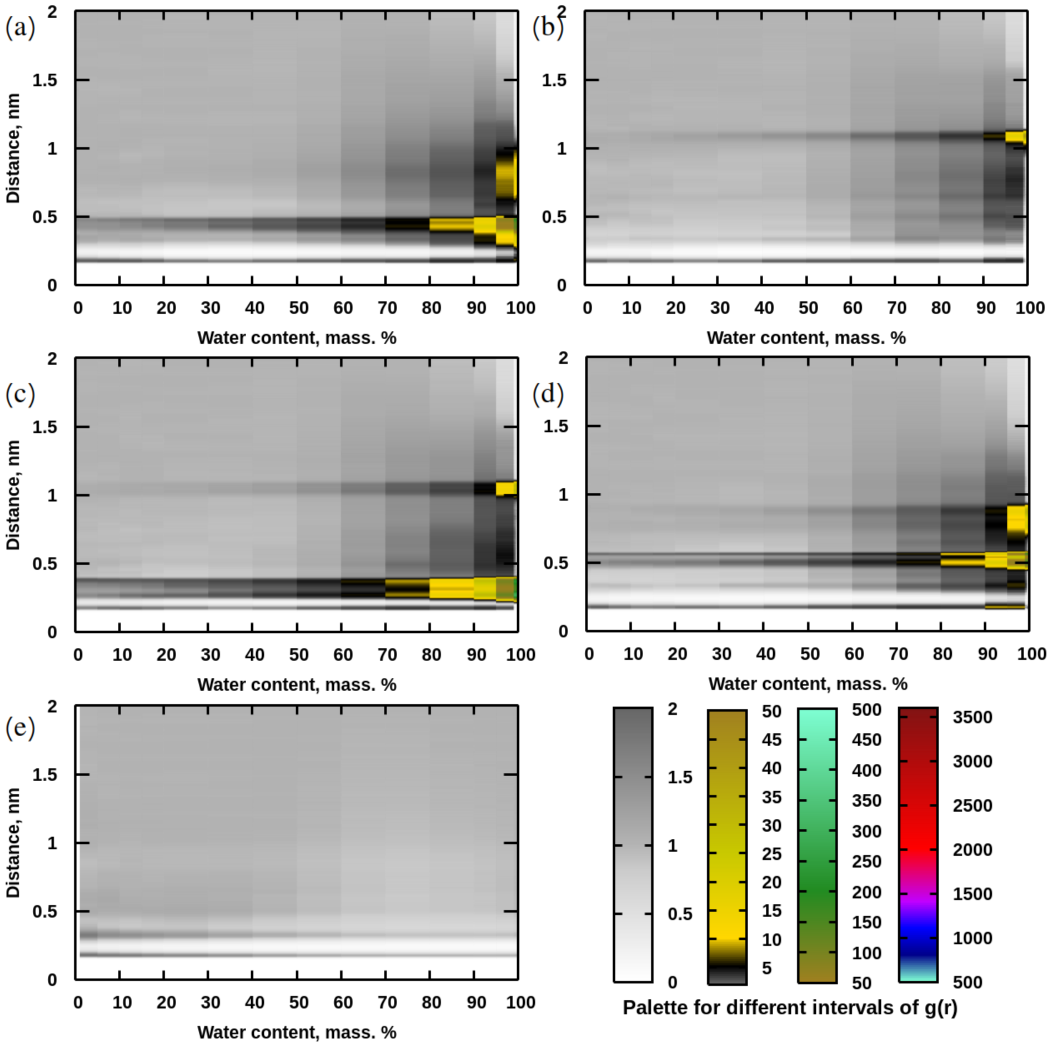

Figure S26 RDFs between oxygens O3/O10 of trehalose and hydrogens in hydroxyl groups of trehalose and water (systems without pre-heating). (a) H8/H22 (b) H9/H21 (c) H10/H20 (d) H11/H19 (e) hydrogens in water.

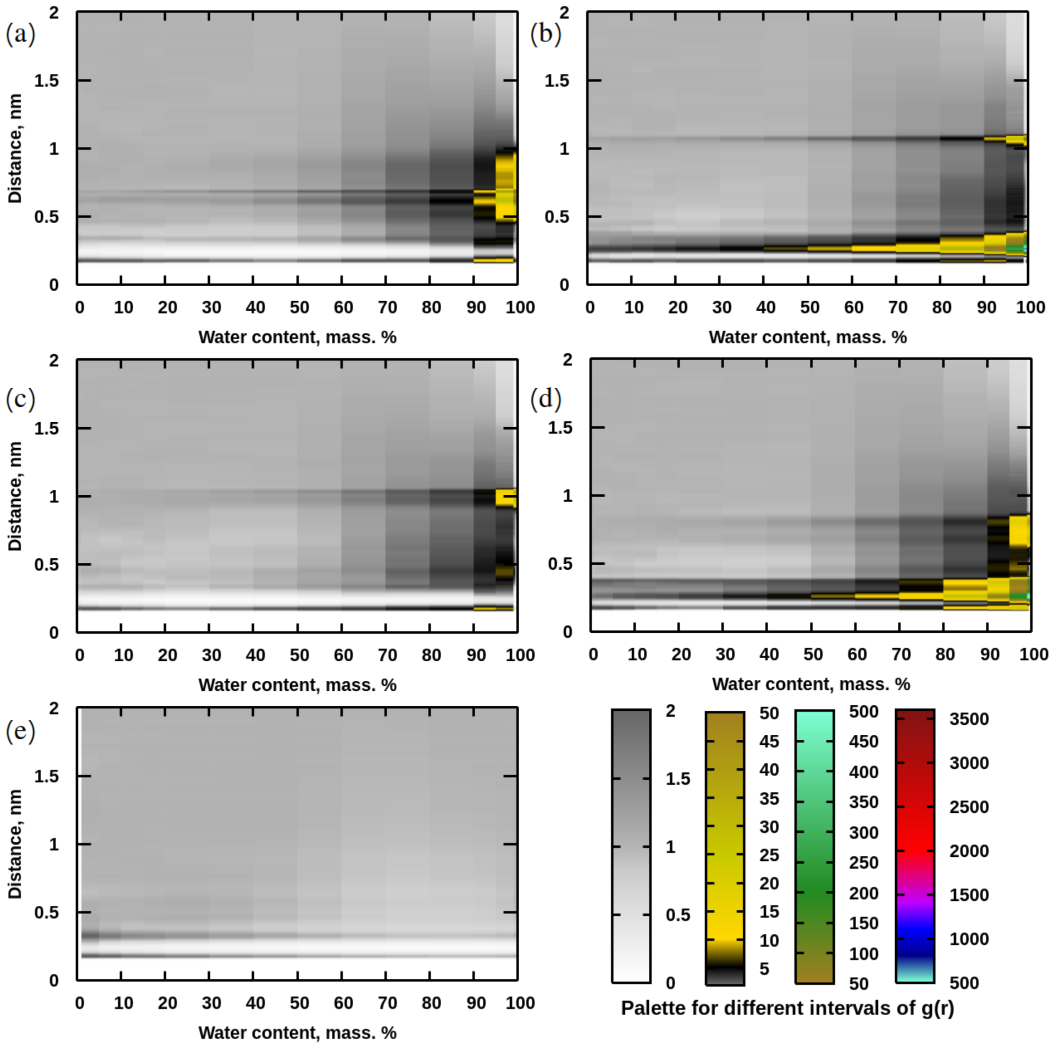

Figure S27 RDFs between oxygens O4/O9 of trehalose and hydrogens in hydroxyl groups of trehalose and water (systems without pre-heating). (a) H8/H22 (b) H9/H21 (c) H10/H20 (d) H11/H19 (e) hydrogens in water.

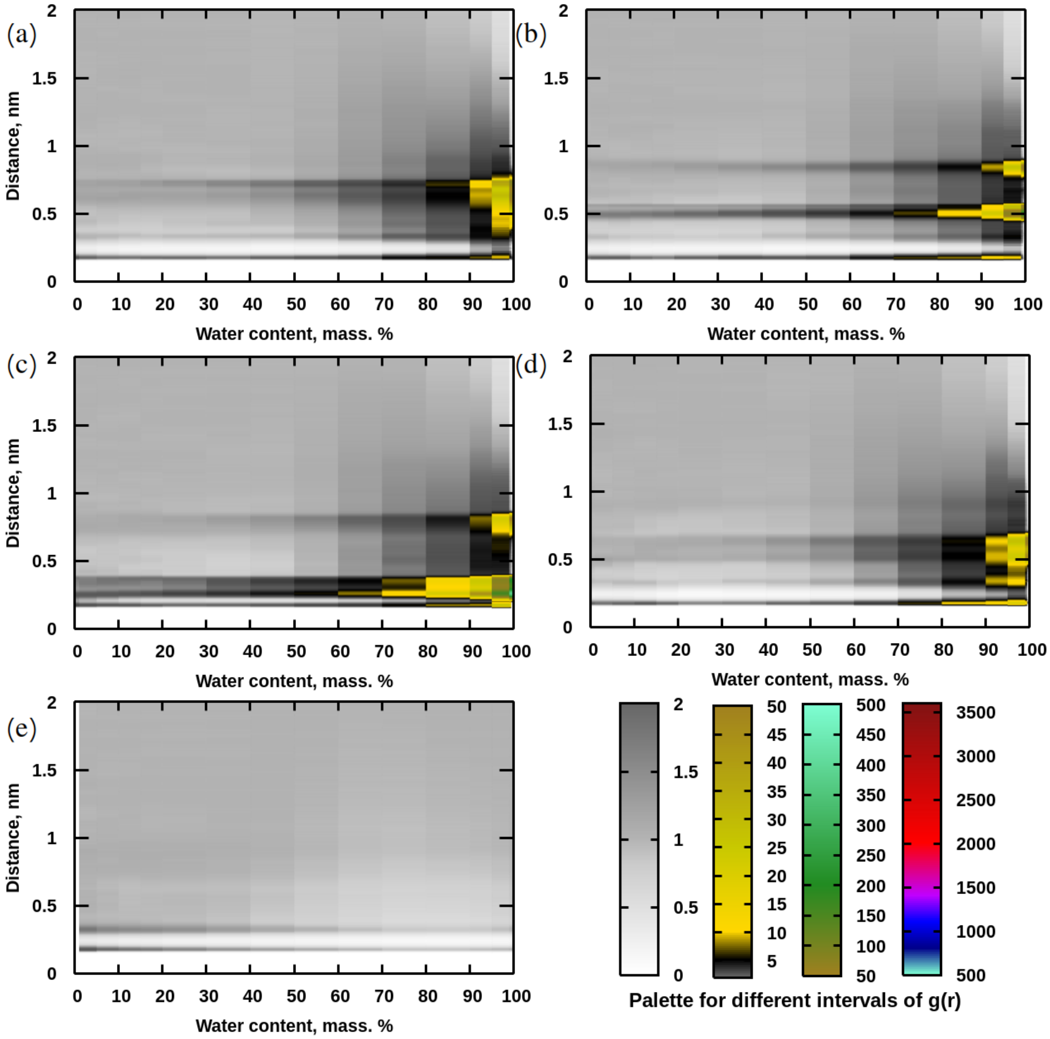

Figure S28 RDFs between oxygens O5/O8 of trehalose and hydrogens in hydroxyl groups of trehalose and water (systems without pre-heating). (a) H8/H22 (b) H9/H21 (c) H10/H20 (d) H11/H19 (e) hydrogens in water.

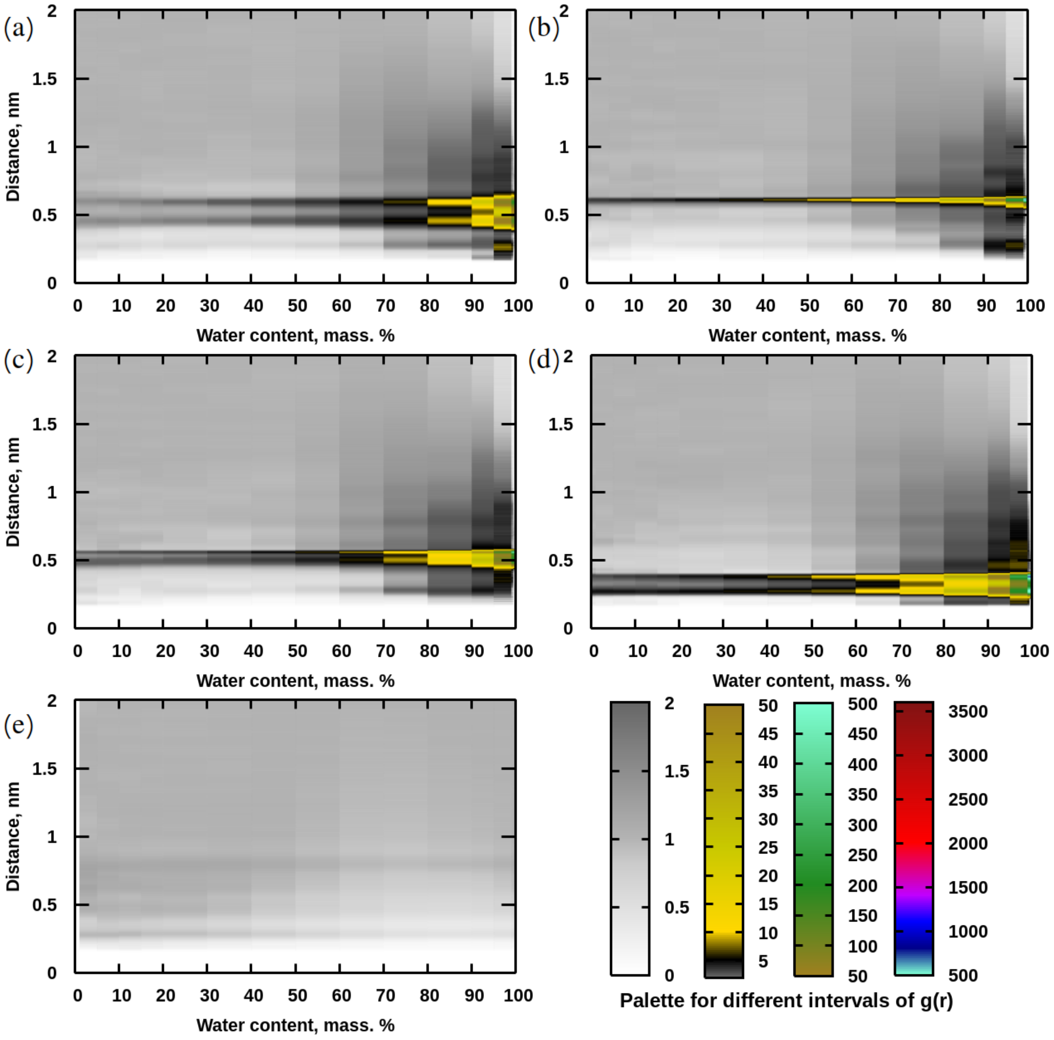

Figure S29 RDFs between oxygen O6 of trehalose and hydrogens in hydroxyl groups of trehalose and water (systems without pre-heating). (a) H8/H22 (b) H9/H21 (c) H10/H20 (d) H11/H19 (e) hydrogens in water.

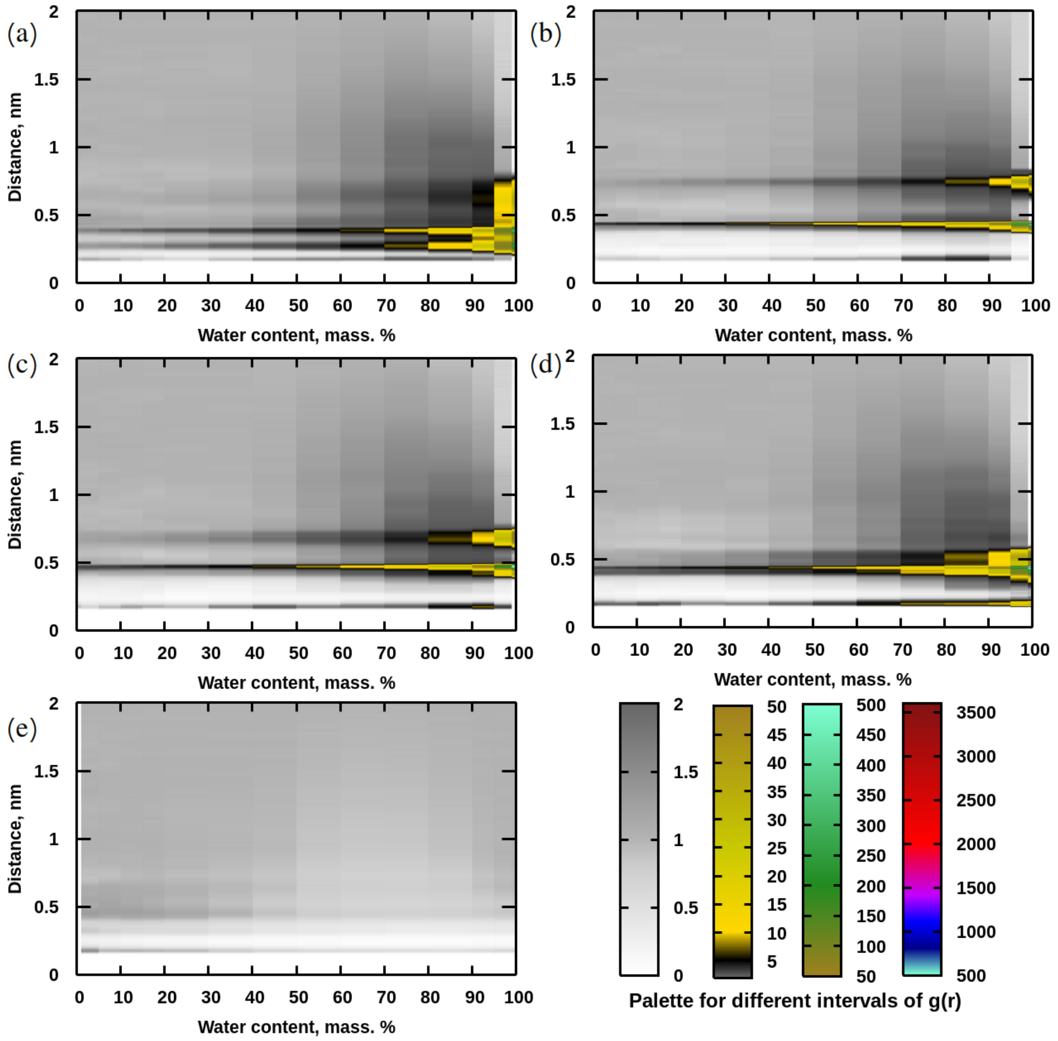

Figure S30 RDFs between oxygens O1/O7 of trehalose and hydrogens in hydroxyl groups of trehalose and water (systems with pre-heating). (a) H8/H22 (b) H9/H21 (c) H10/H20 (d) H11/H19 (e) hydrogens in water.

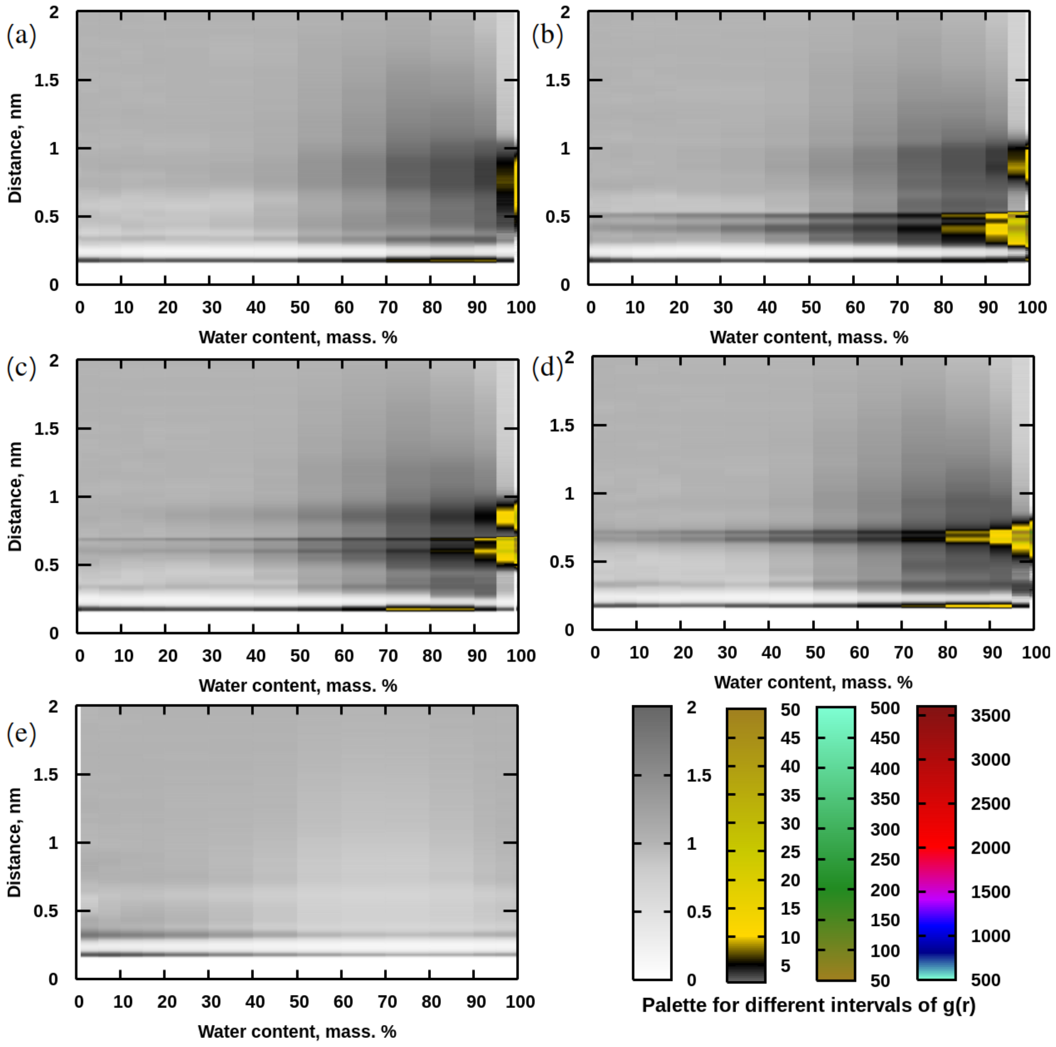

Figure S31 RDFs between oxygens O2/O11 of trehalose and hydrogens in hydroxyl groups of trehalose and water (systems with pre-heating). (a) H8/H22 (b) H9/H21 (c) H10/H20 (d) H11/H19 (e) hydrogens in water.

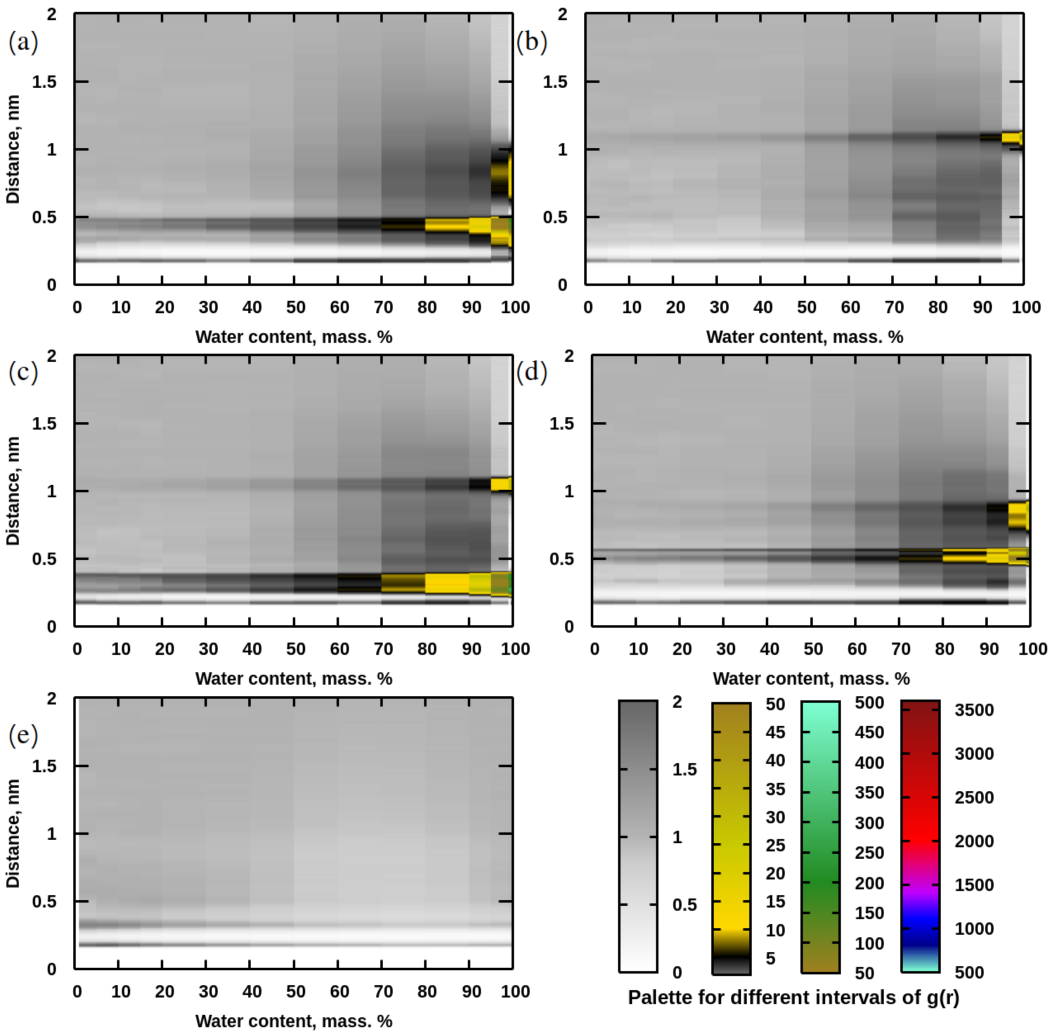

Figure S32 RDFs between oxygens O3/O10 of trehalose and hydrogens in hydroxyl groups of trehalose and water (systems with pre-heating). (a) H8/H22 (b) H9/H21 (c) H10/H20 (d) H11/H19 (e) hydrogens in water.

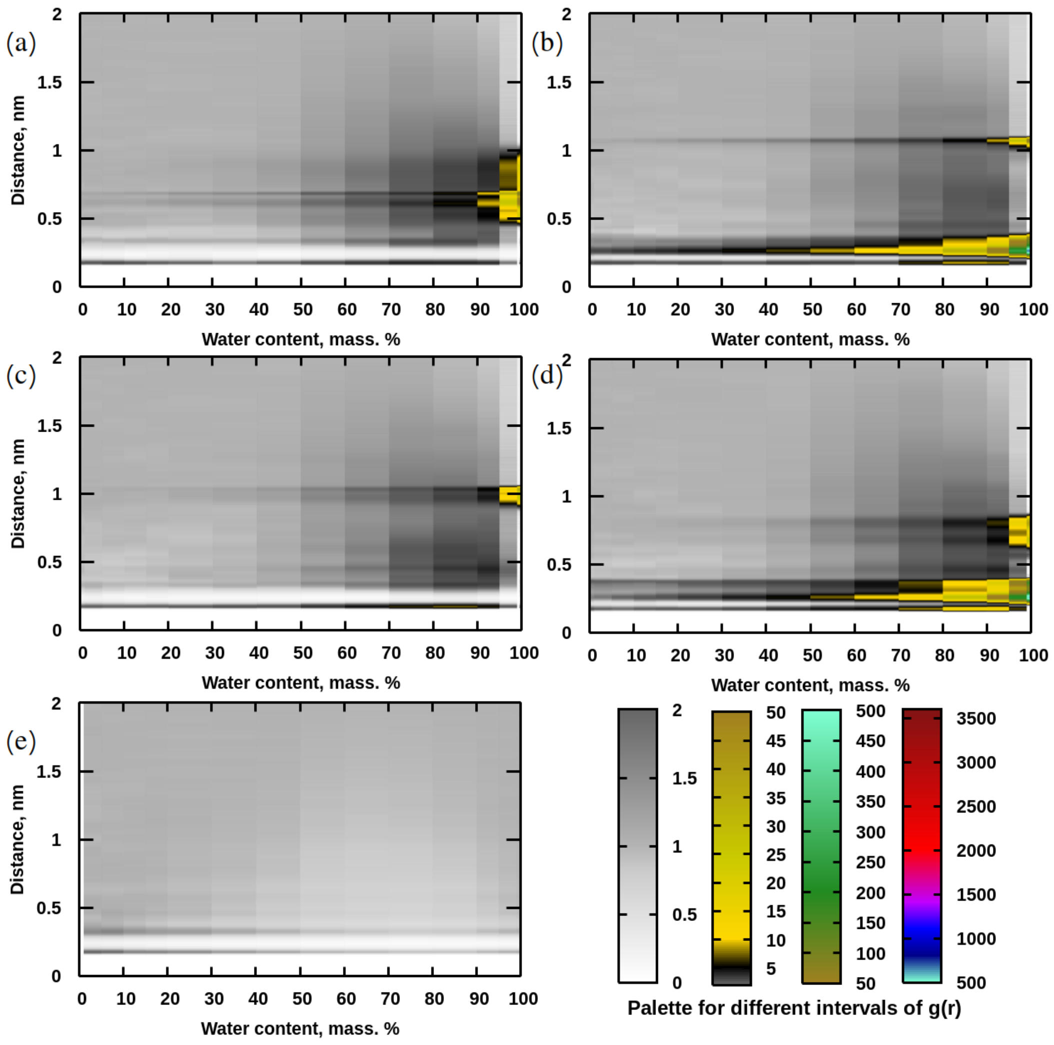

Figure S33 RDFs between oxygens O4/O9 of trehalose and hydrogens in hydroxyl groups of trehalose and water (systems with pre-heating). (a) H8/H22 (b) H9/H21 (c) H10/H20 (d) H11/H19 (e) hydrogens in water.

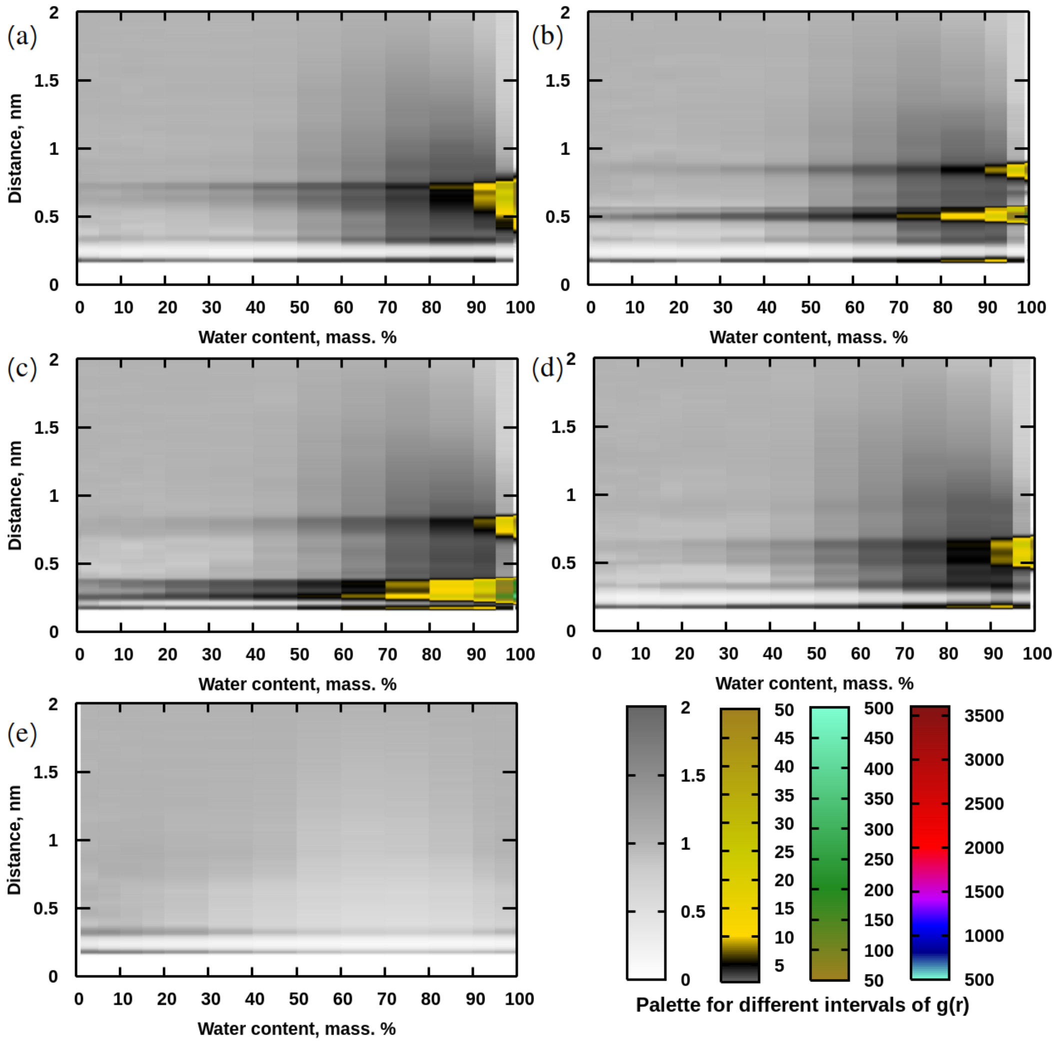

Figure S34 RDFs between oxygens O5/O8 of trehalose and hydrogens in hydroxyl groups of trehalose and water (systems with pre-heating). (a) H8/H22 (b) H9/H21 (c) H10/H20 (d) H11/H19 (e) hydrogens in water.

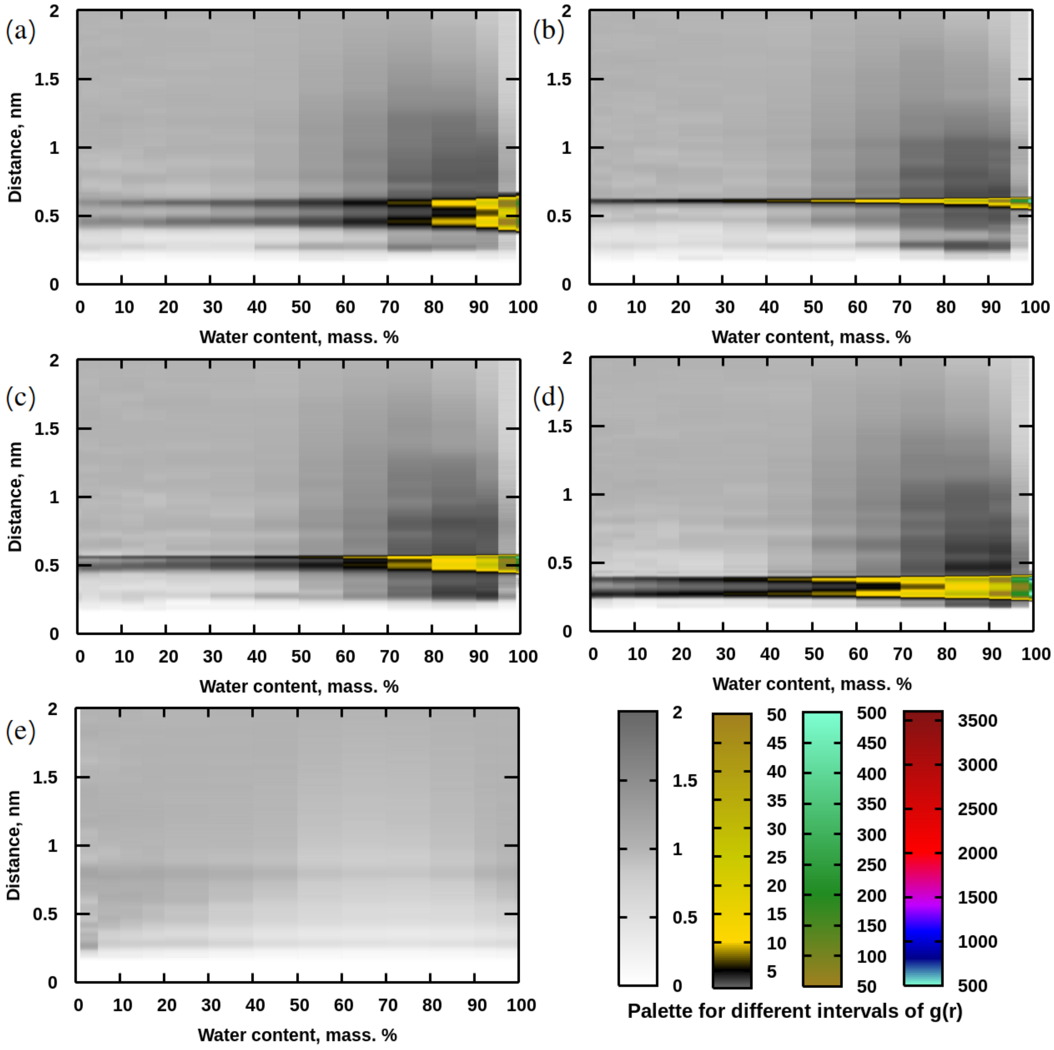

Figure S35 RDFs between oxygen O6 of trehalose and hydrogens in hydroxyl groups of trehalose and water (systems with pre-heating). (a) H8/H22 (b) H9/H21 (c) H10/H20 (d) H11/H19 (e) hydrogens in water.

2.3 Water-water radial distribution functions in all all systems

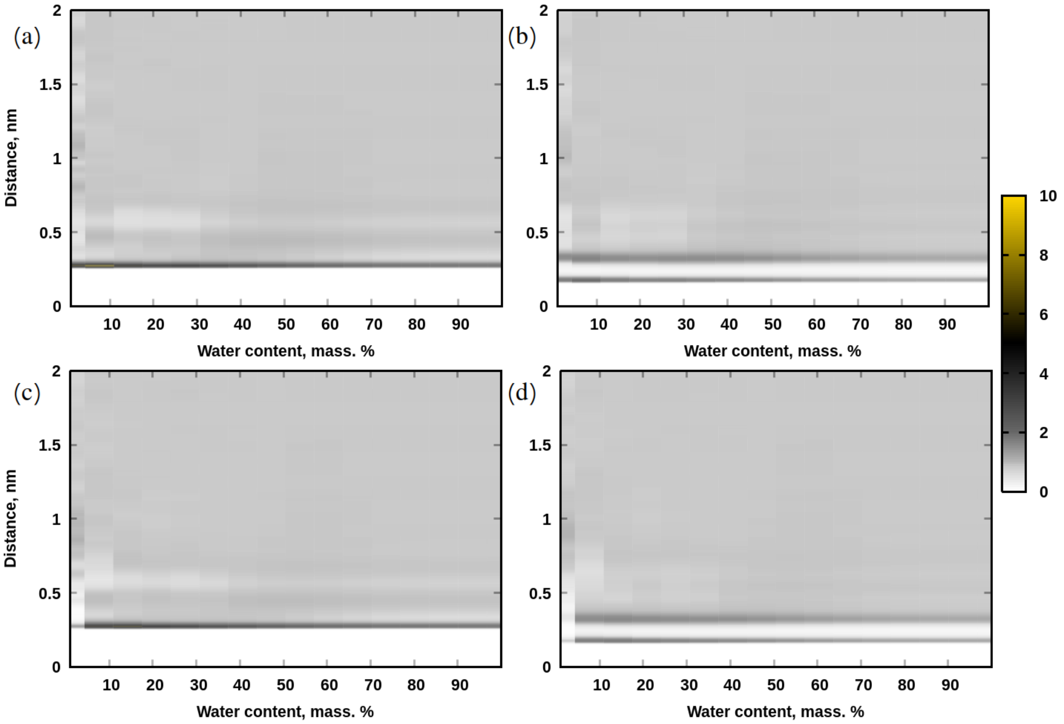

Figure S36 RDFs for associations of water molecules. (a) Hydrogen-oxygen associations in systems with sucrose (without pre-heating). (b) Oxygen-oxygen associations in systems with sucrose (without pre-heating). (c) Hydrogen-oxygen associations in systems with trehalose (without pre-heating). (d) Oxygen-oxygen associations in systems with trehalose (without pre-heating).

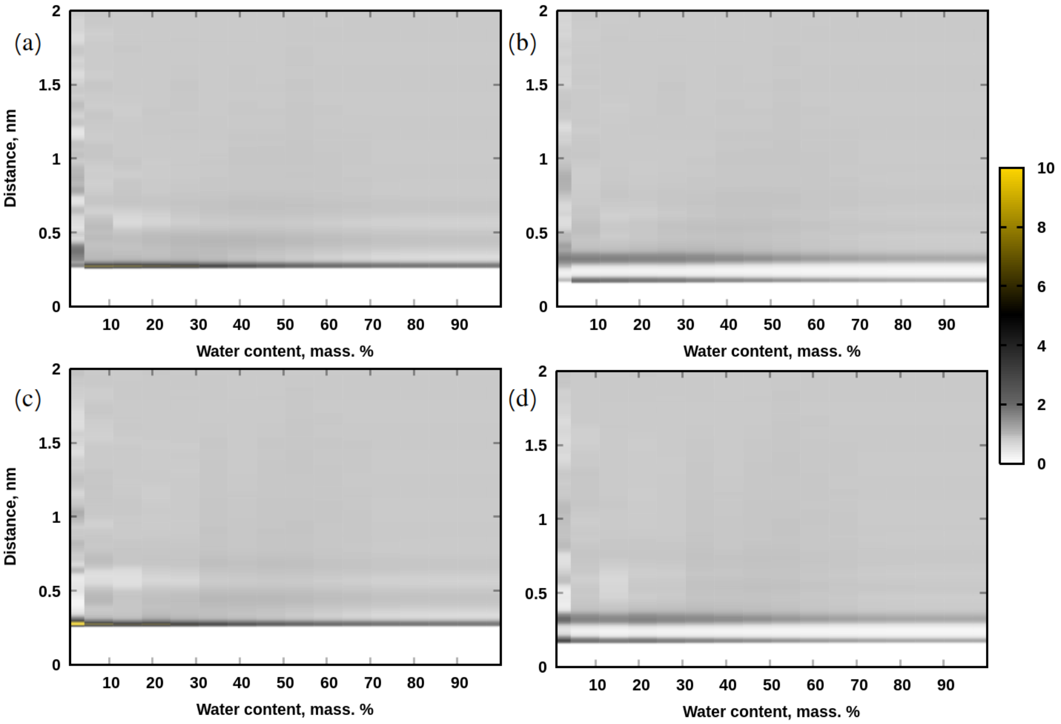

Figure S37 RDFs for associations of water molecules. (a) Hydrogen-oxygen associations in systems with sucrose (with pre-heating). (b) Oxygen-oxygen associations in systems with sucrose (with pre-heating). (c) Hydrogen-oxygen associations in systems with trehalose (with pre-heating). (d) Oxygen-oxygen associations in systems with trehalose (with pre-heating).

3 Distributions of dihedrals

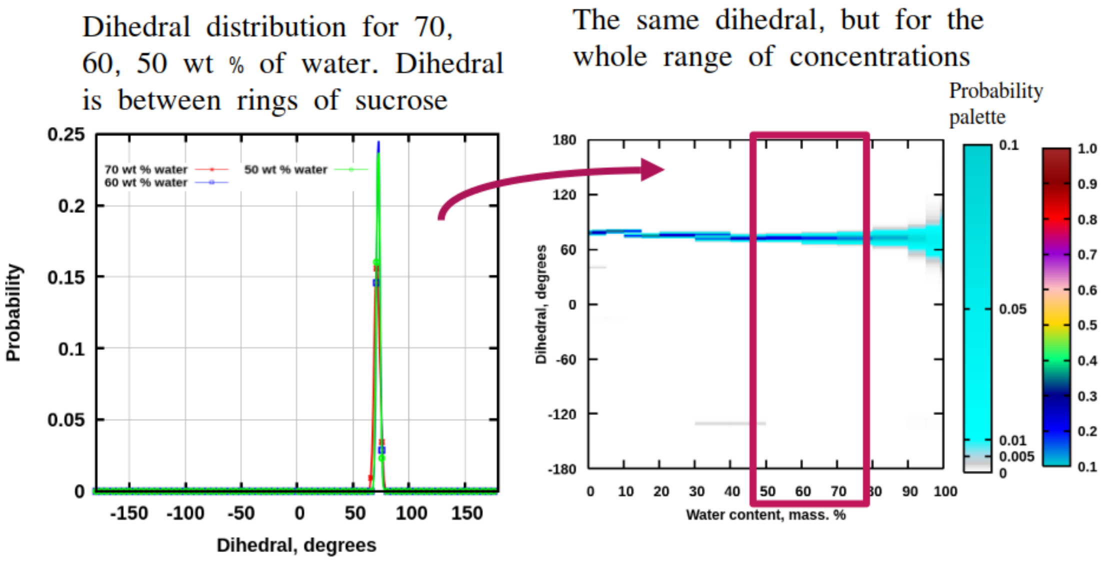

Figure S38 Illustration for distribution of dihedrals for figures S39-S46.

3.1 Sucrose

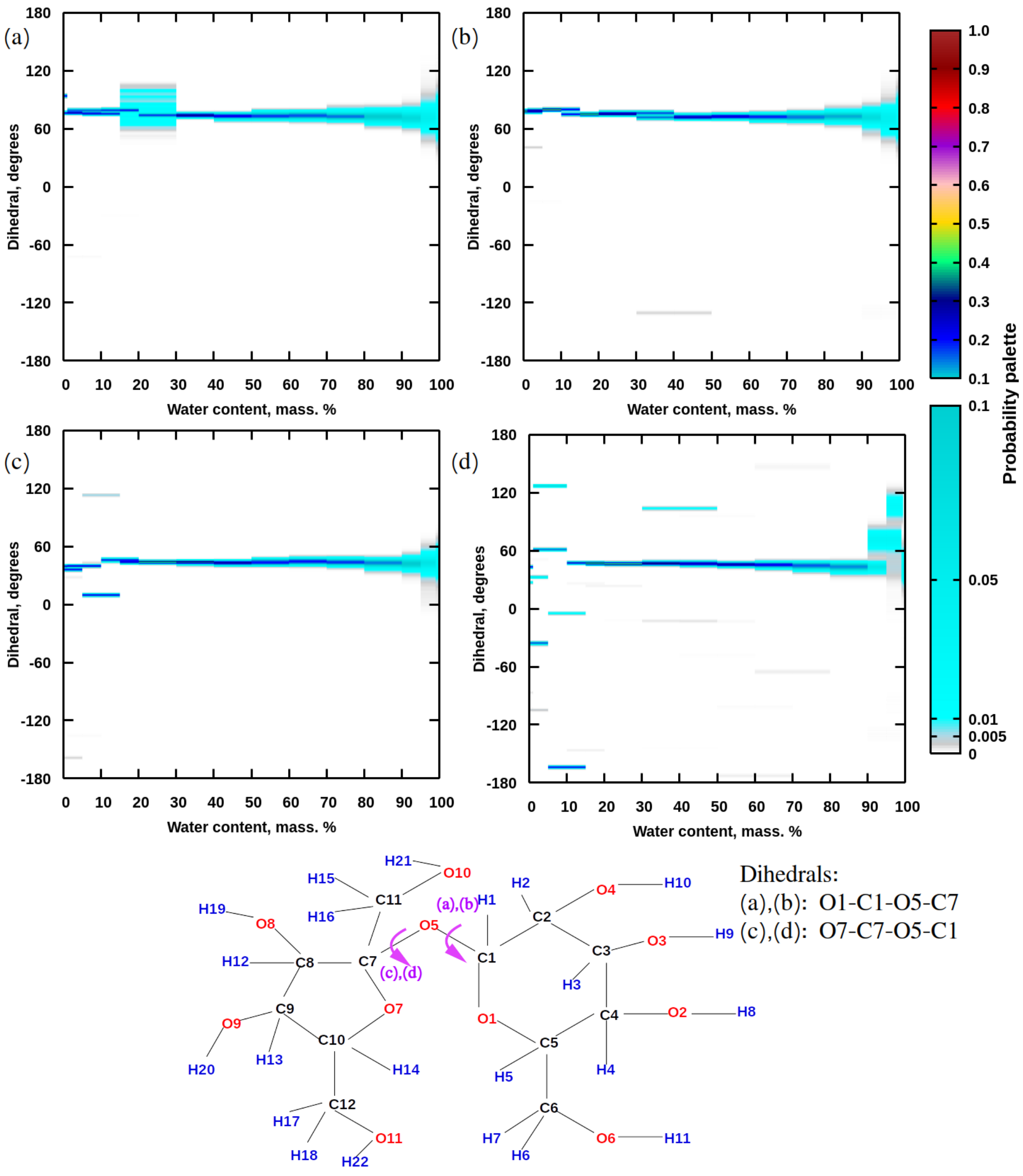

Figure S39 Distributions of dihedrals for sucrose. (a) and (c) are systems without pre-heating. (b) and (d) are systems with pre-heating.

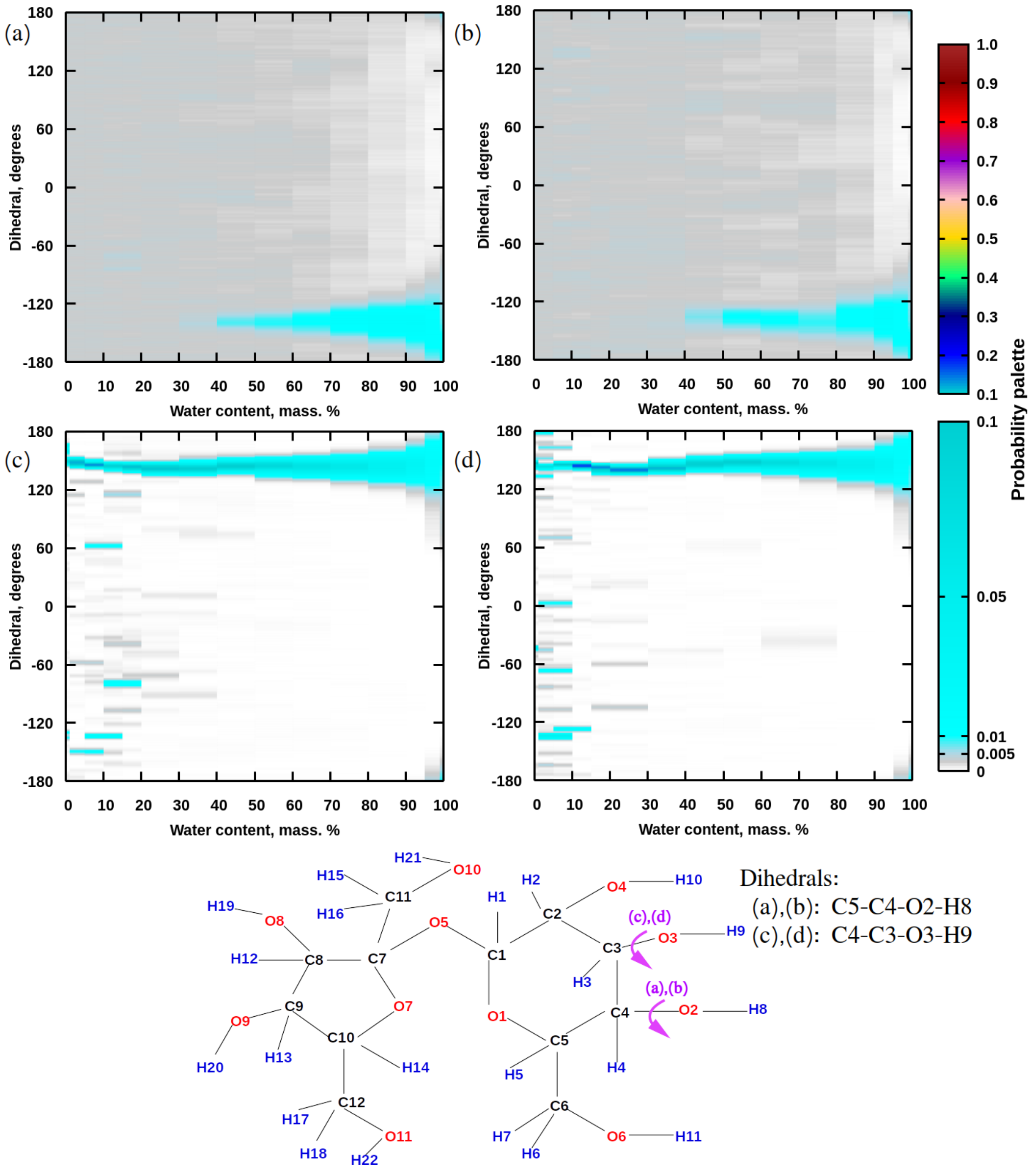

Figure S40 Distributions of dihedrals for sucrose. (a) and (c) are systems without pre-heating. (b) and (d) are systems with pre-heating.

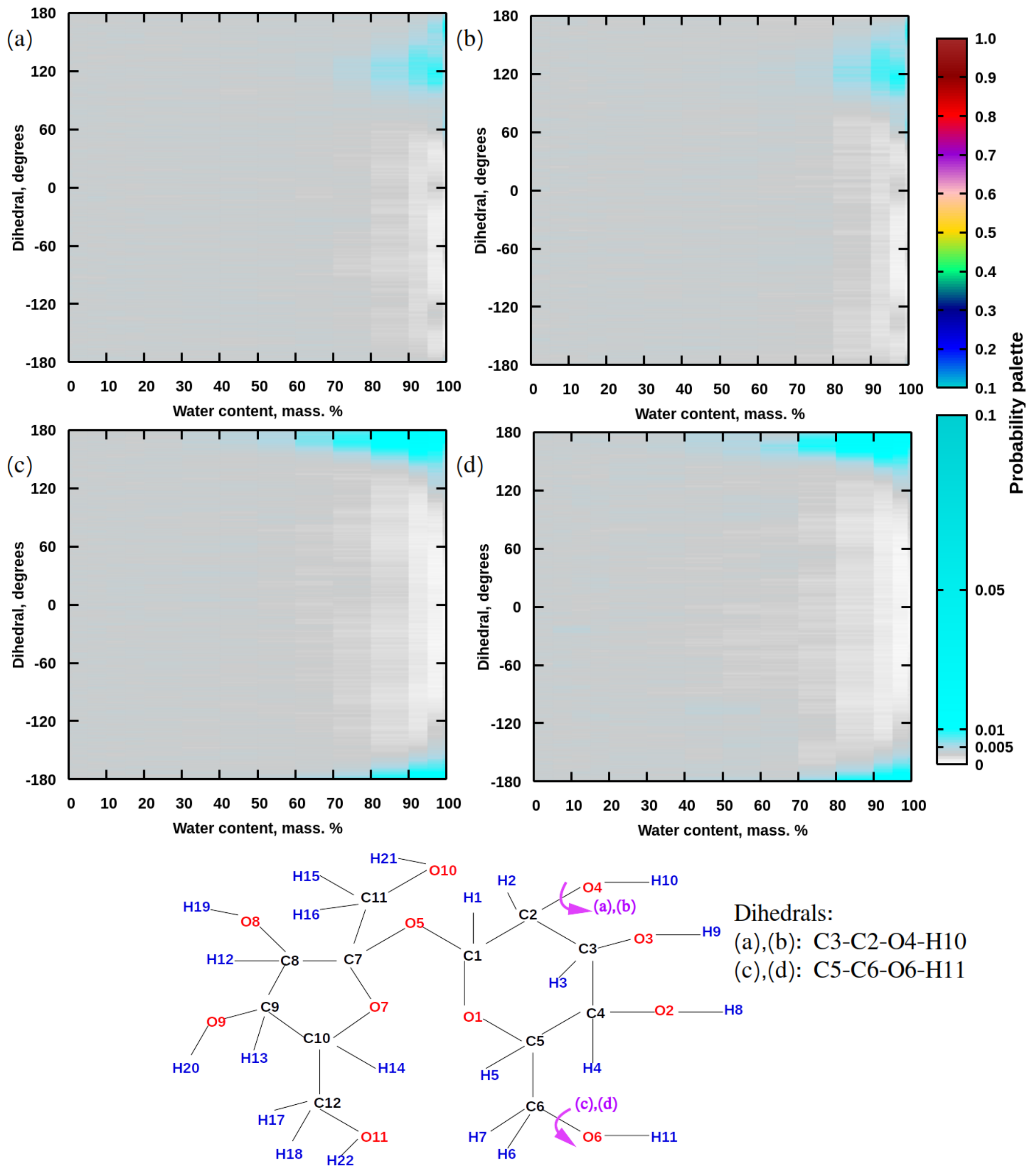

Figure S41 Distributions of dihedrals for sucrose. (a) and (c) are systems without pre-heating. (b) and (d) are systems with pre-heating.

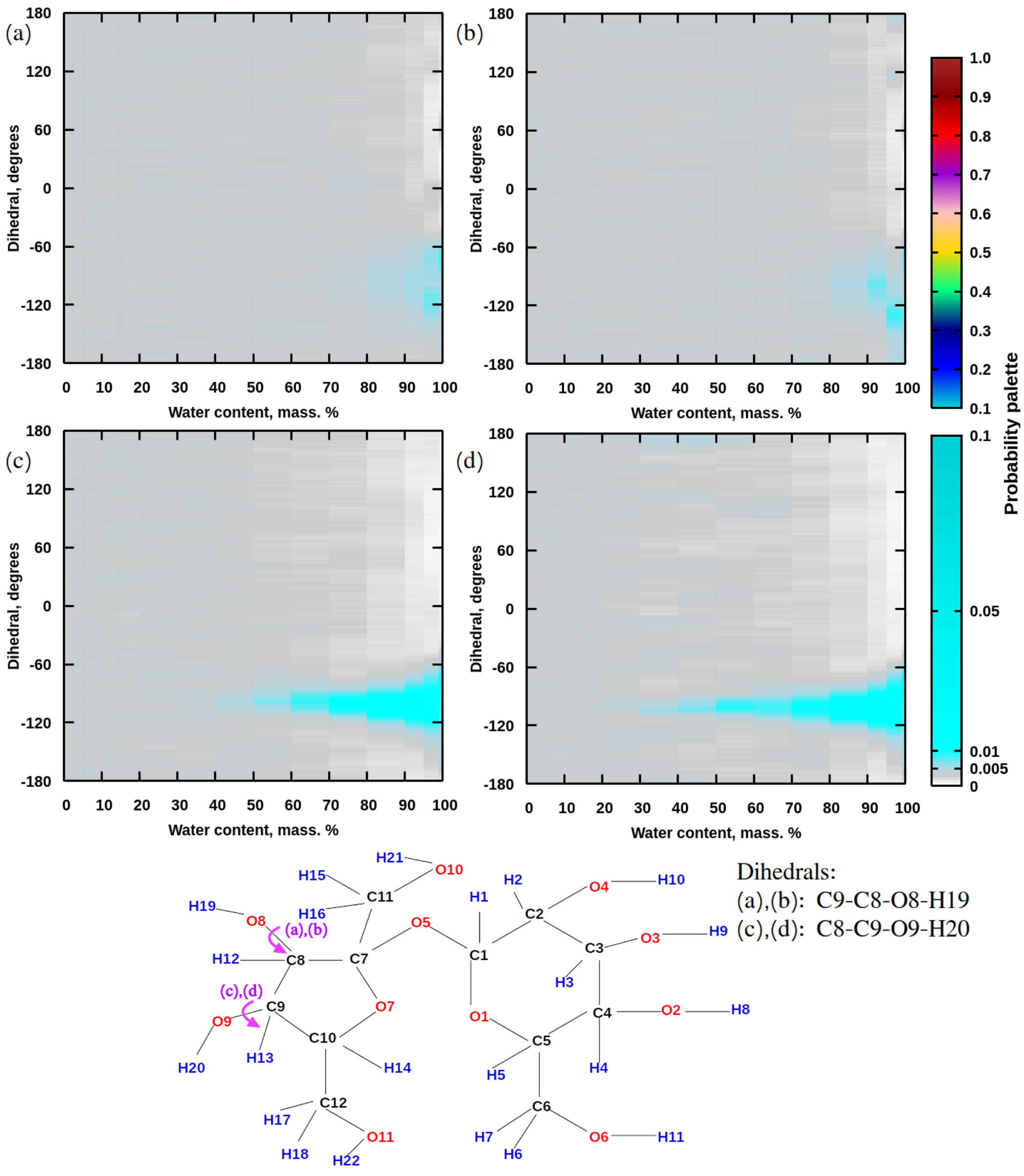

Figure S42 Distributions of dihedrals for sucrose. (a) and (c) are systems without pre-heating. (b) and (d) are systems with pre-heating.

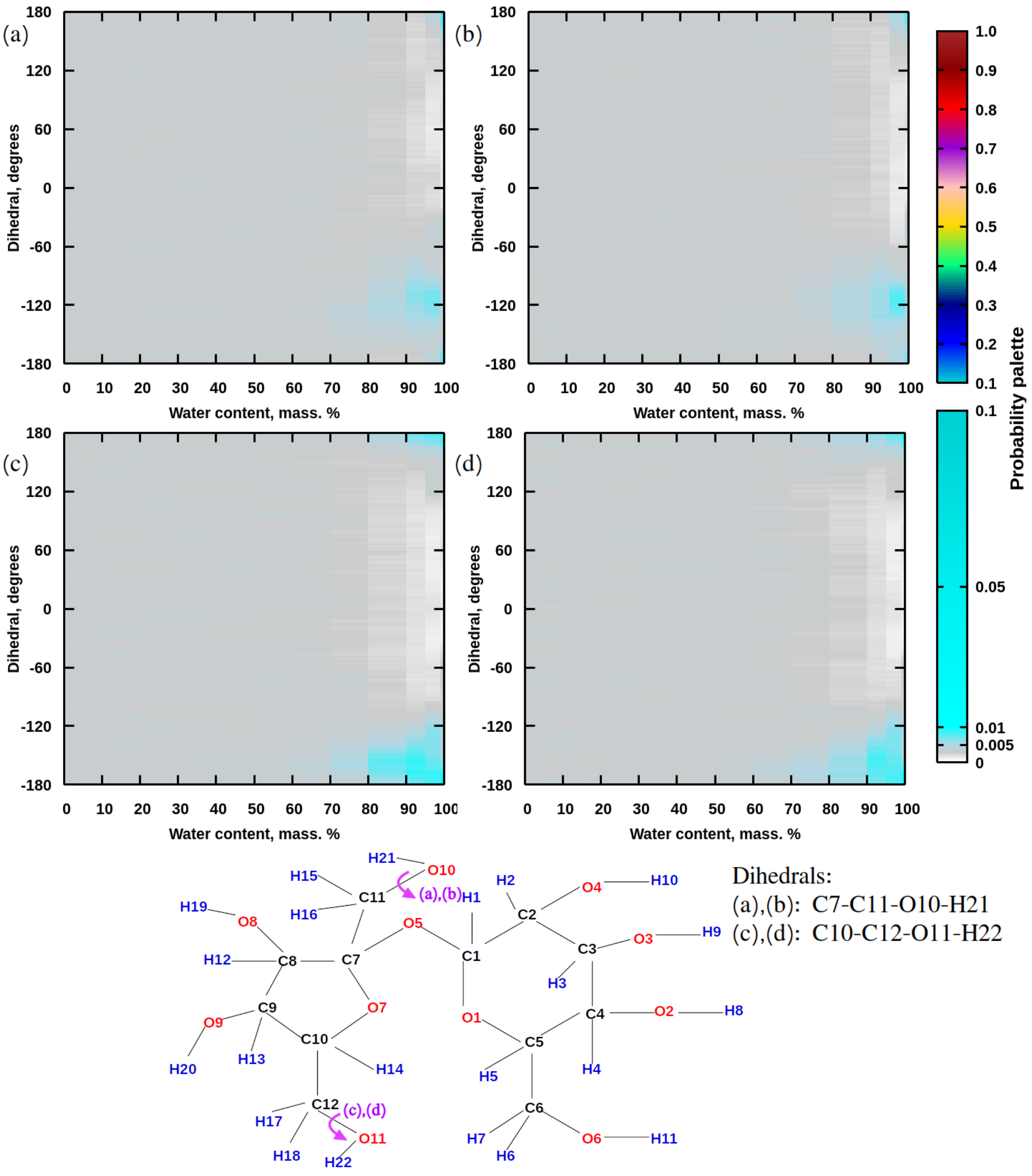

Figure S43 Distributions of dihedrals for sucrose. (a) and (c) are systems without pre-heating. (b) and (d) are systems with pre-heating.

3.2 Trehalose

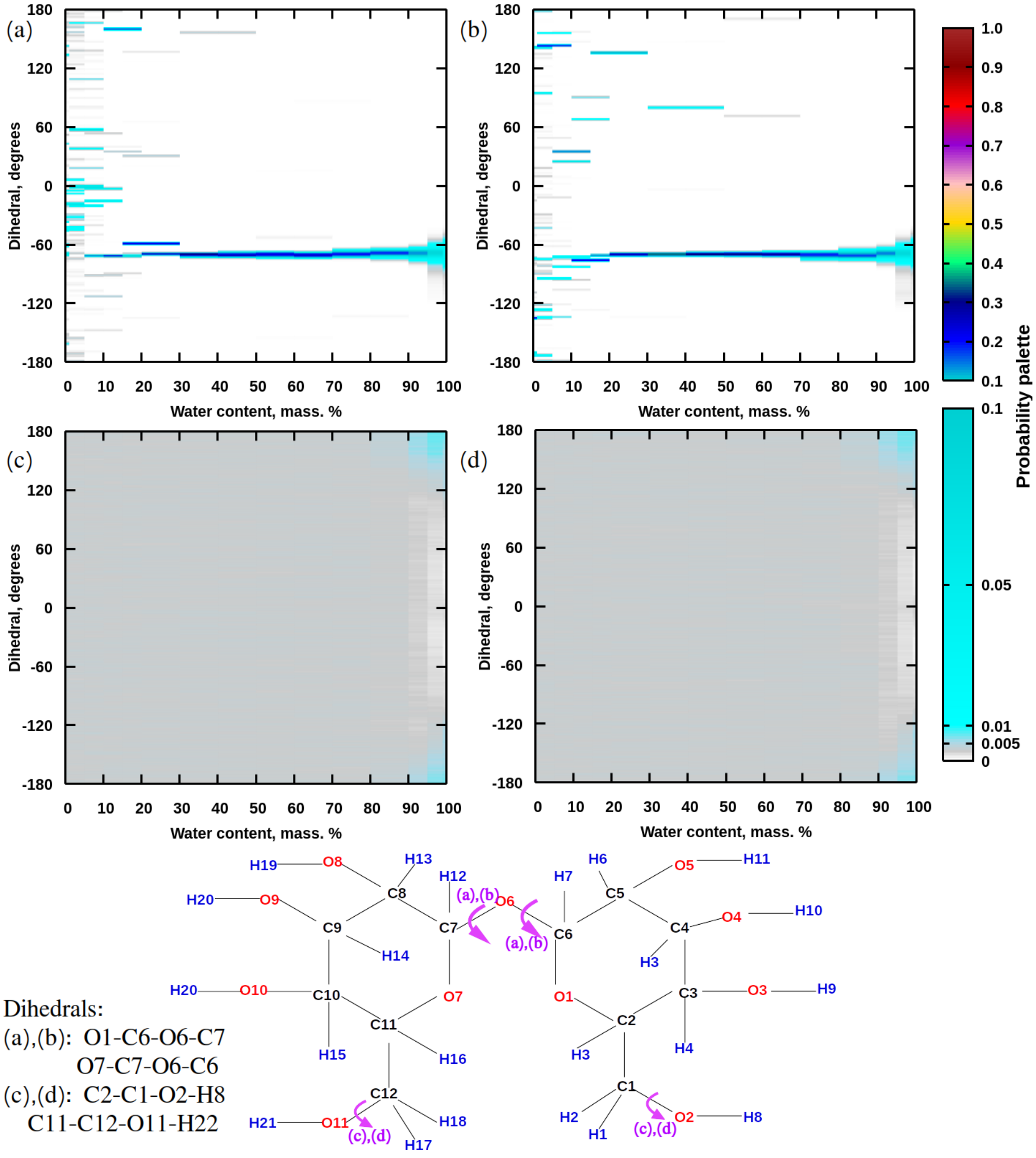

Figure S44 Distributions of dihedrals for trehalose. (a) and (c) are systems without pre-heating. (b) and (d) are systems with pre-heating.

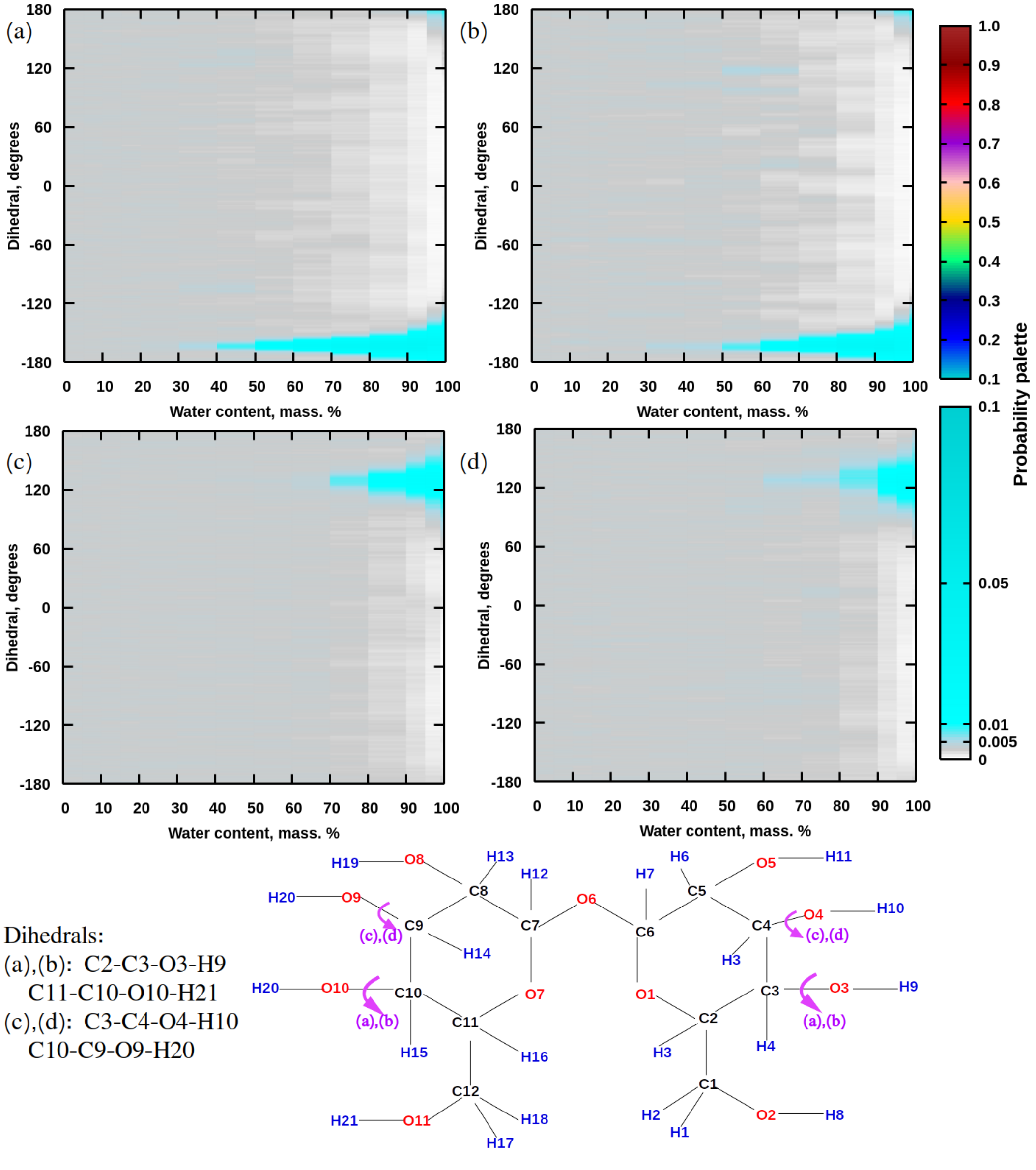

Figure S45 Distributions of dihedrals for trehalose. (a) and (c) are systems without pre-heating. (b) and (d) are systems with pre-heating.

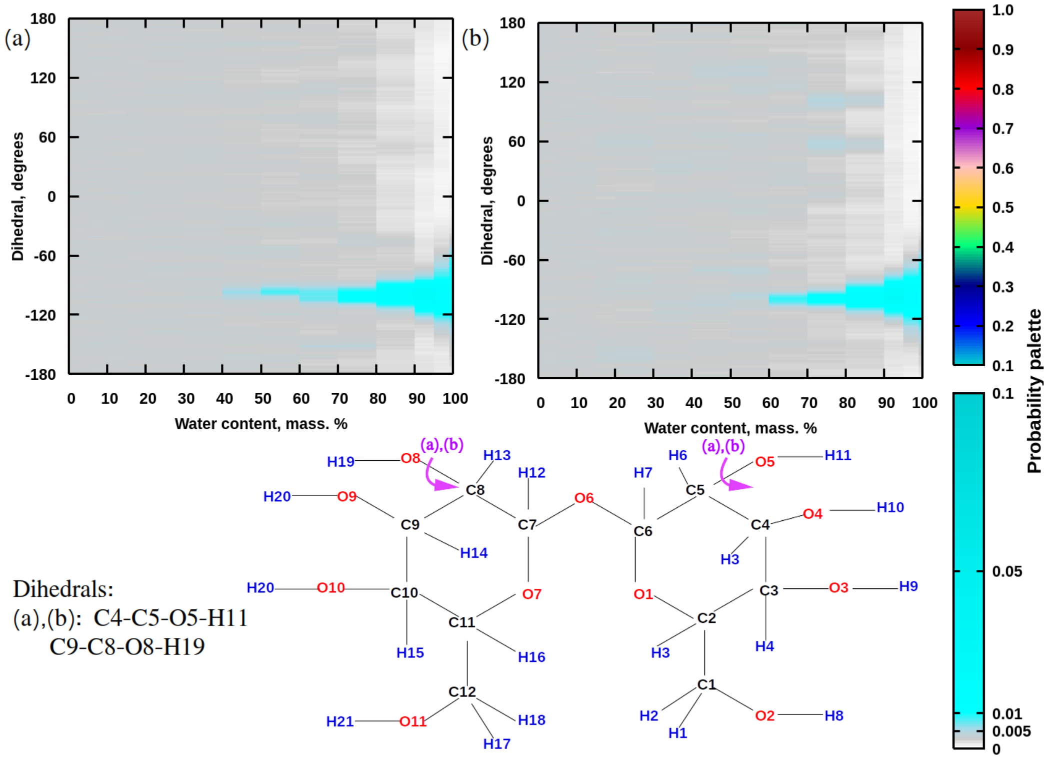

Figure S46 Distributions of dihedrals for trehalose. (a) are systems without pre-heating. (b) are systems with pre-heating.

4 Umbrella sampling: histograms and potential of mean force profiles

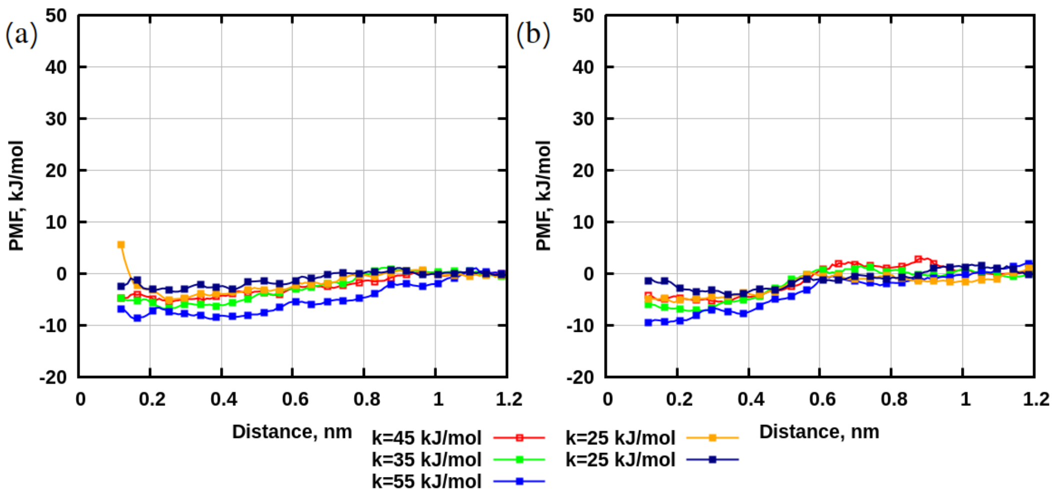

Figure S47 Potential of mean force profiles. (a) Sucrose. (b) Trehalose. Force constants are mentioned in the legend.

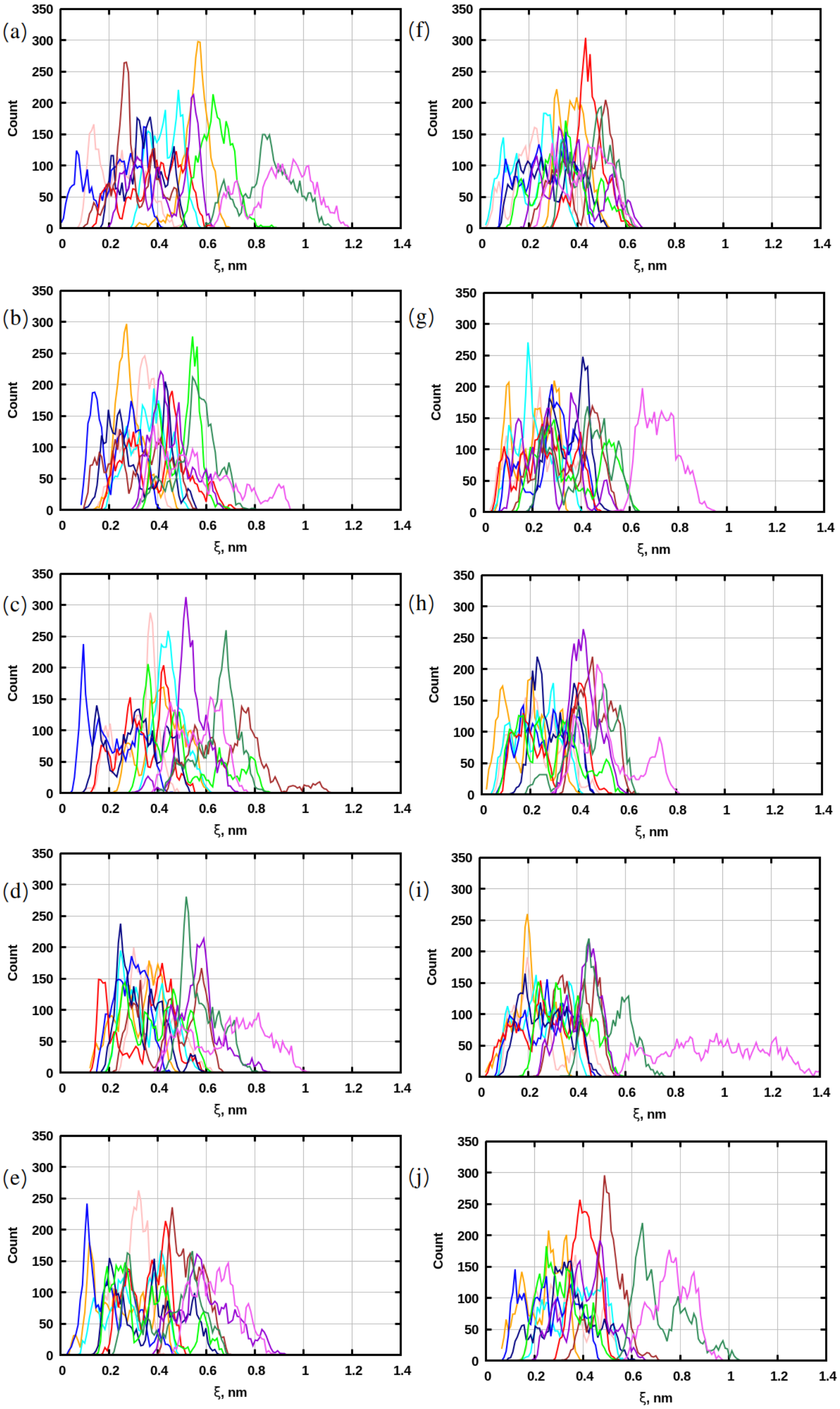

Figure S48 Histograms for umbrella sampling simulations with sucrose and trehalose. (a) Sucrose, k=45 kJ/mol. (b) Sucrose, k=35 kJ/mol. (c) Sucrose, k=55 kJ/mol. (d) Sucrose, k=25 kJ/mol. (e) Sucrose, k=15 kJ/mol. (f) Trehalose, k=45 kJ/mol. (g) Trehalose, k=35 kJ/mol. (h) Trehalose, k=55 kJ/mol. (i) Trehalose, k=25 kJ/mol. (j) Trehalose, k=15 kJ/mol.

5 Well-tempered metadynamics for sugar-sugar: collective variables, Gaussians

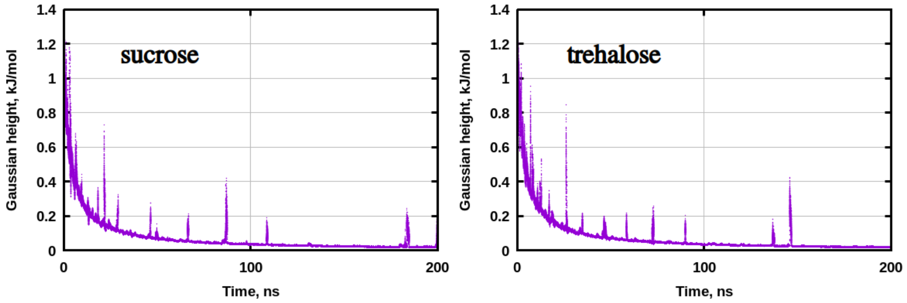

Figure S49 Evolution of Gaussian heights for simulations with 2 molecules of sugars in water.

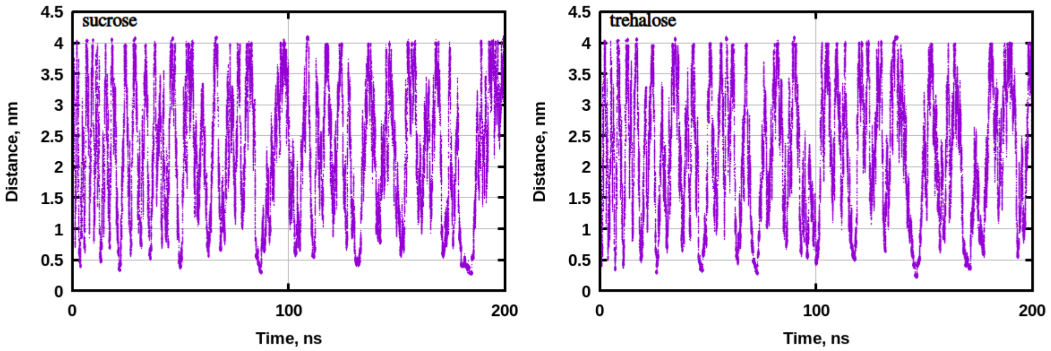

Figure S50 Evolution of collective variables (distance between centers of mass of sucrose/trehalose) for simulations with 2 molecules of sugars in water.

6 Well-tempered metadynamics for sugar-water: collective variables, Gaussians

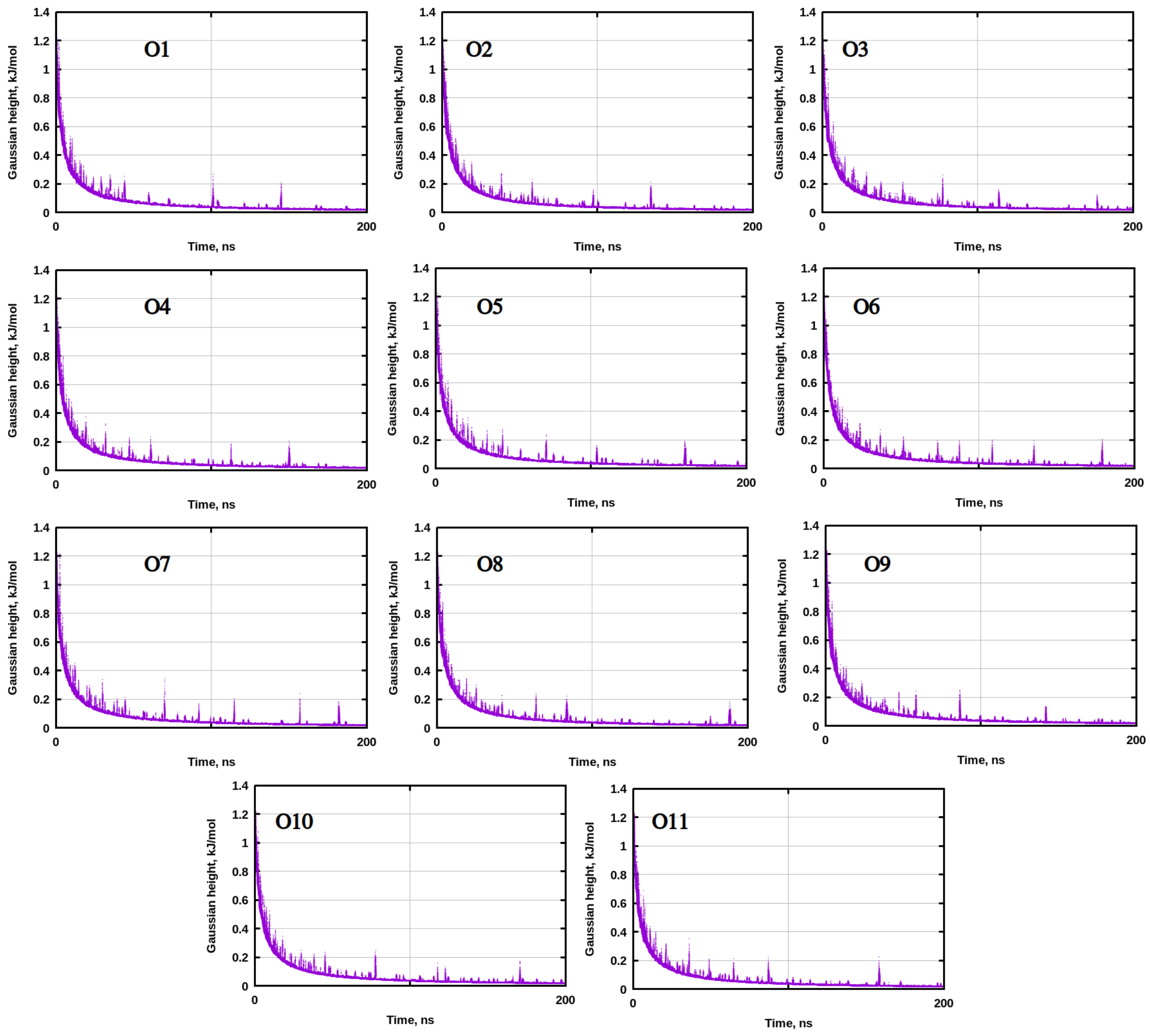

Figure S51 Evolution of Gaussian heights for simulations with single molecule of sucrose in water. Every group is labeled by the oxygen atom present in it.

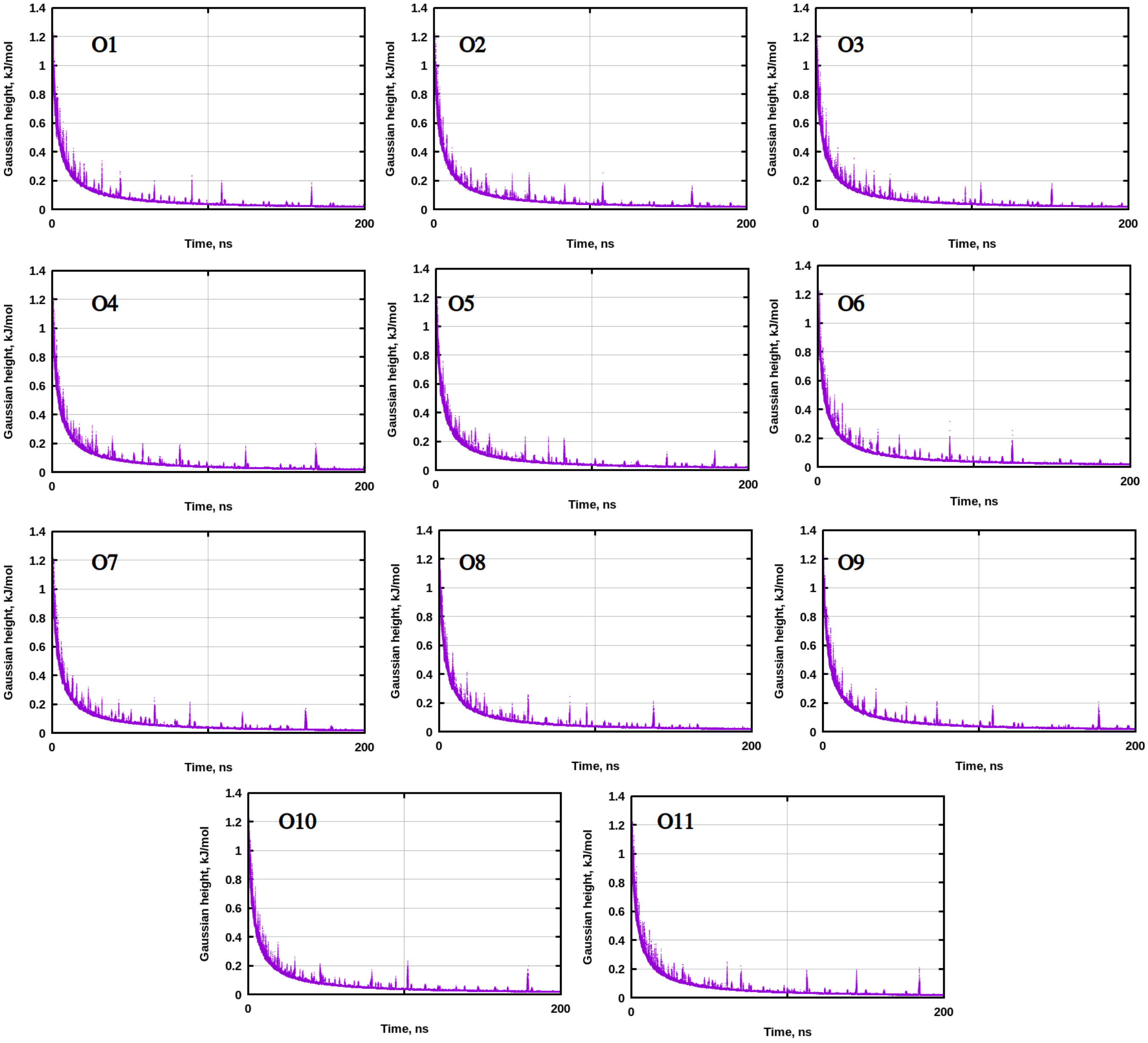

Figure S52 Evolution of Gaussian heights for simulations with single molecule of trehalose in water. Every group is labeled by the oxygen atom present in it.

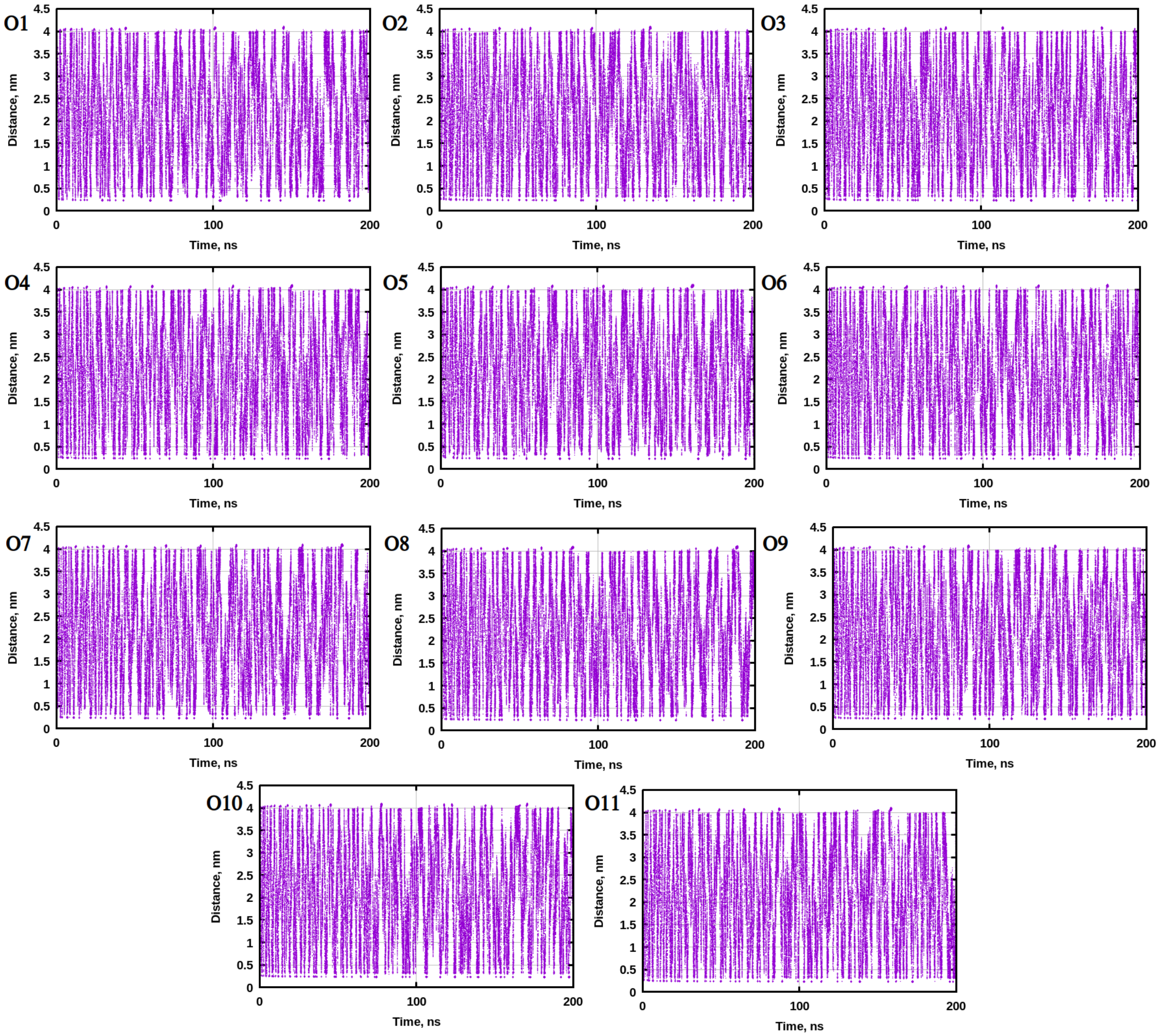

Figure S53 Evolution of collective variables (distance between center of mass of groups and the water molecule) for simulations with single molecule of sucrose in water. Every group is labeled by the oxygen atom present in it.

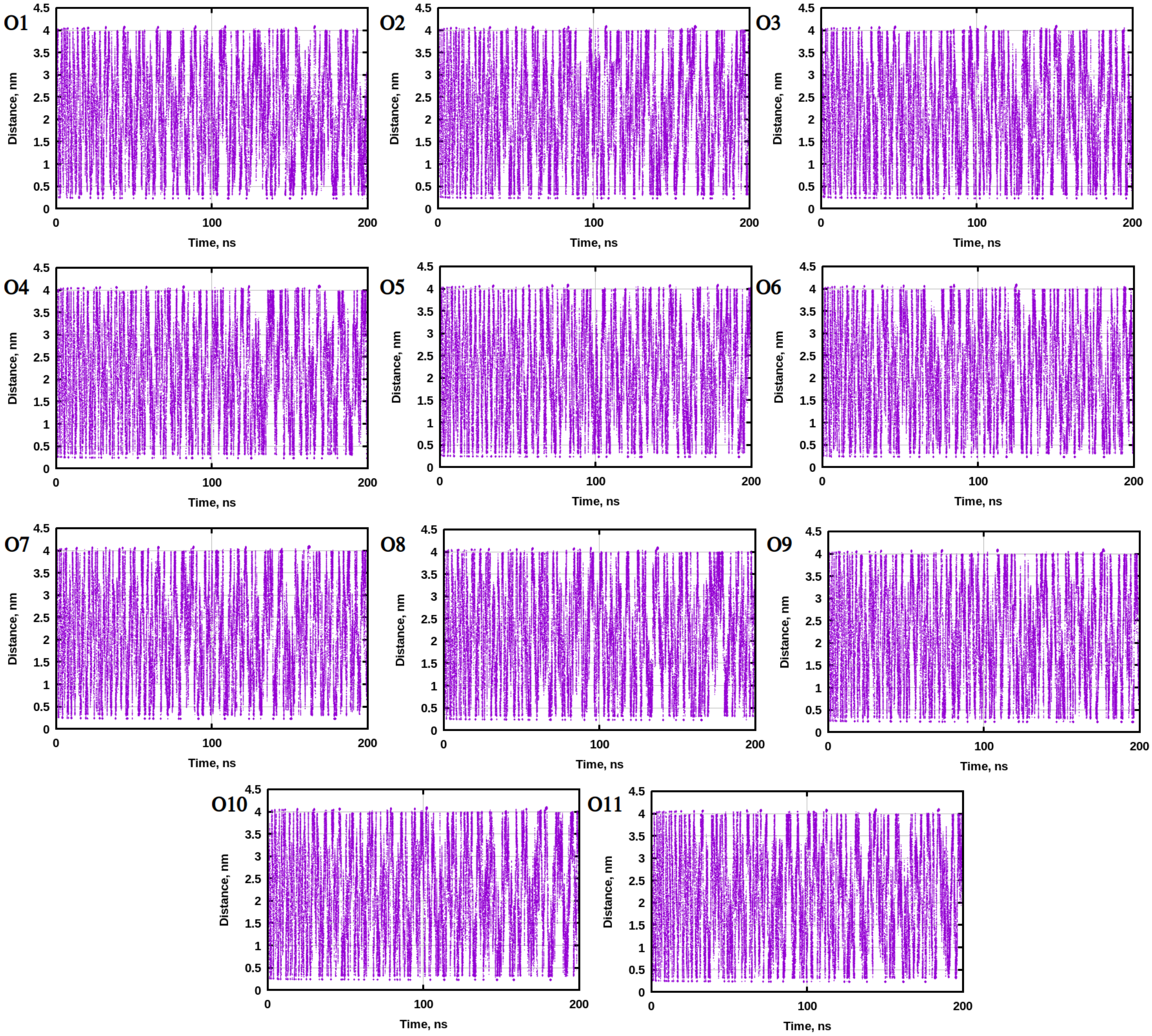

Figure S54 Evolution of collective variables (distance between center of mass of groups and the water molecule) for simulations with single molecule of trehalose in water. Every group is labeled by the oxygen atom present in it.

7 Comparison of RDFs for systems with 1 molecule of sugar: free energy calculations vs MD

RDFs were computed between groups which were used for biasing from the respective free-energy calculation with this group.

7.1 Sucrose

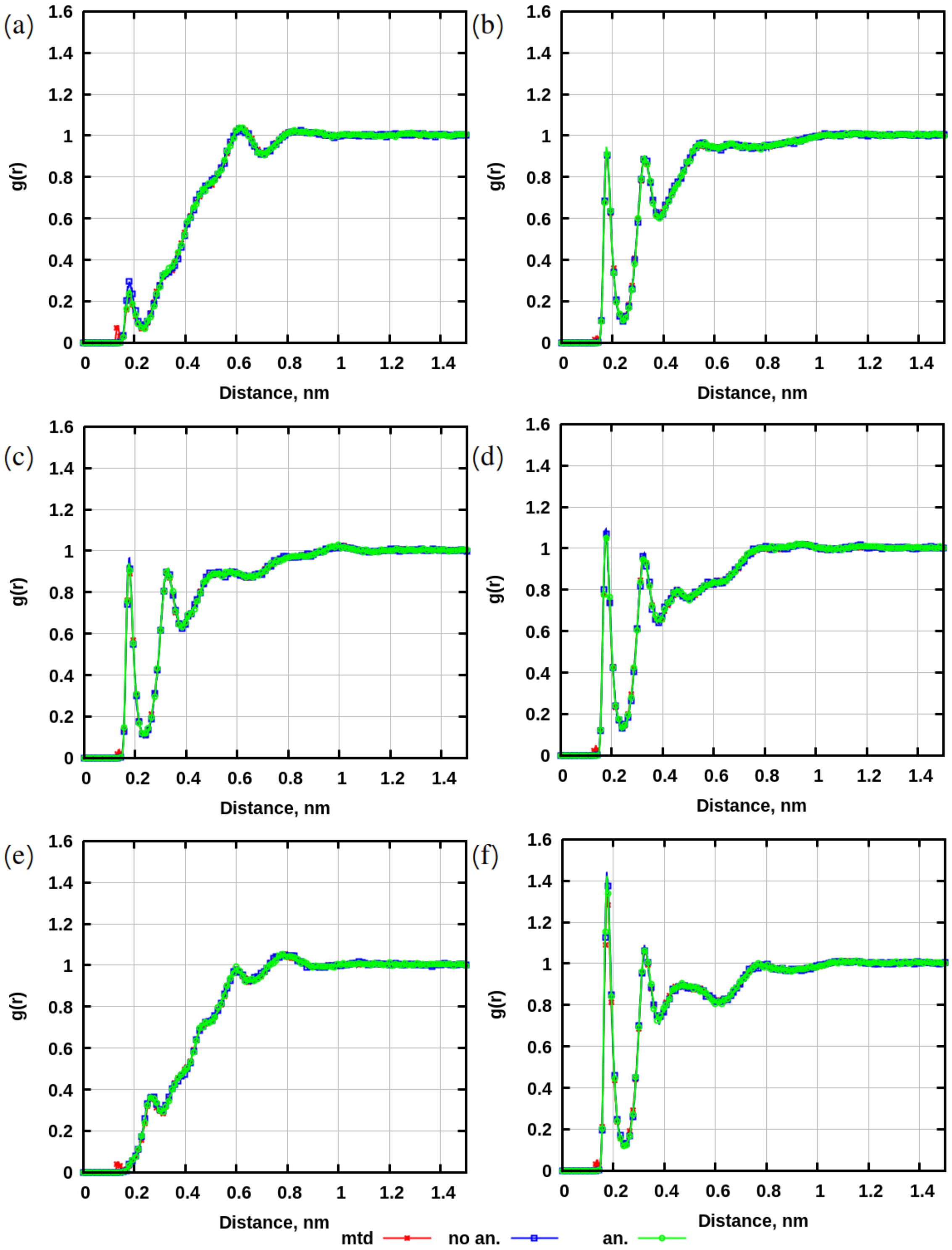

Figure S55 RDFs between oxygen atoms of sucrose and hydrogen atoms of water. (a) O1. (b) O2. (c) O3. (d) O4. (e) O5. (f) O6. Abbreviations: "mtd" - well-tempered metadynamics, "no an." - system simulated without pre-heating, "an." - system simulated with pre-heating.

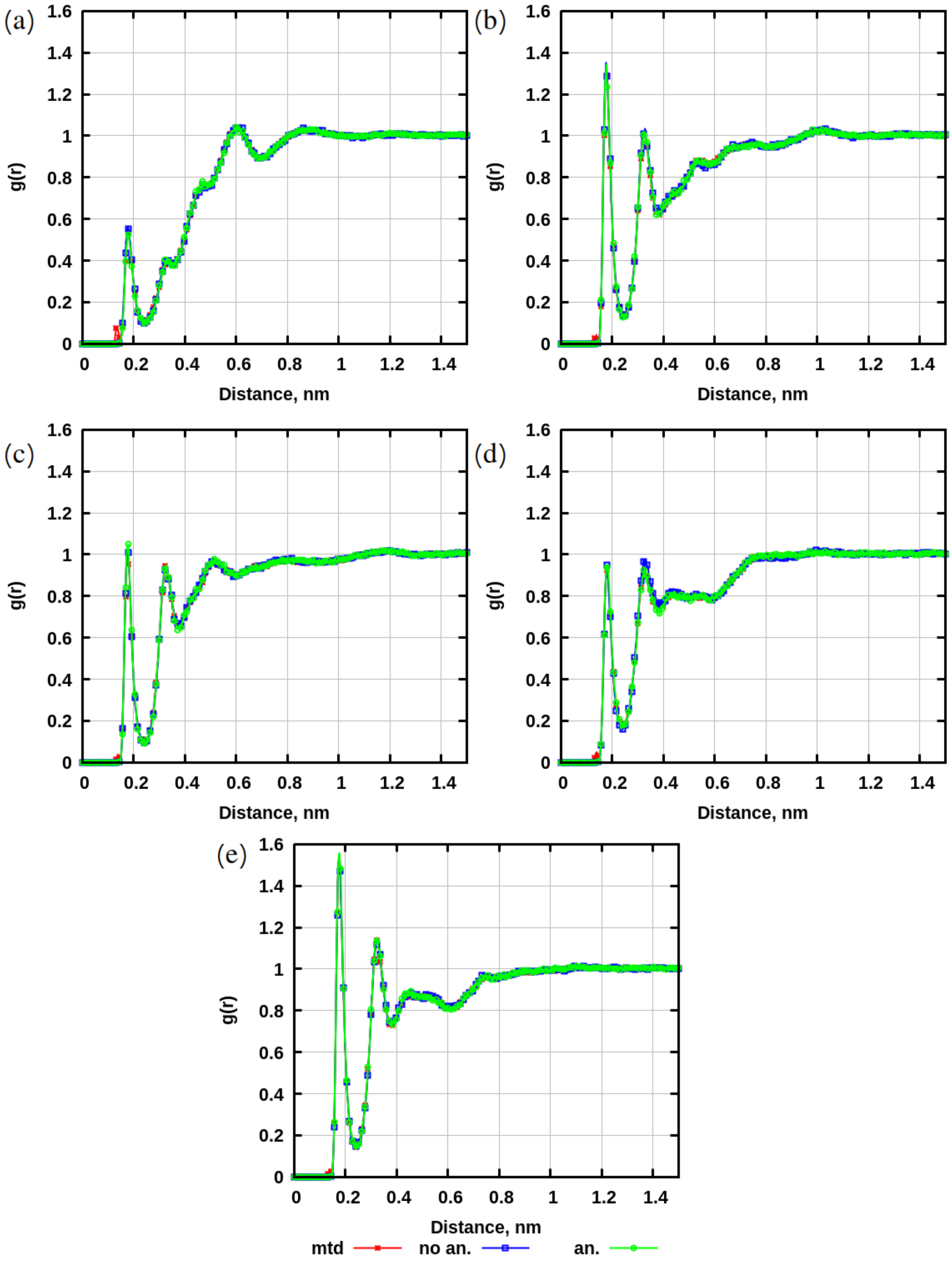

Figure S56 RDFs between oxygen atoms of sucrose and hydrogen atoms of water. (a) O7. (b) O8. (c) O9. (d) O10. (e) O11. Abbreviations: "mtd" - well-tempered metadynamics, "no an." - system simulated without pre-heating, "an." - system simulated with pre-heating.

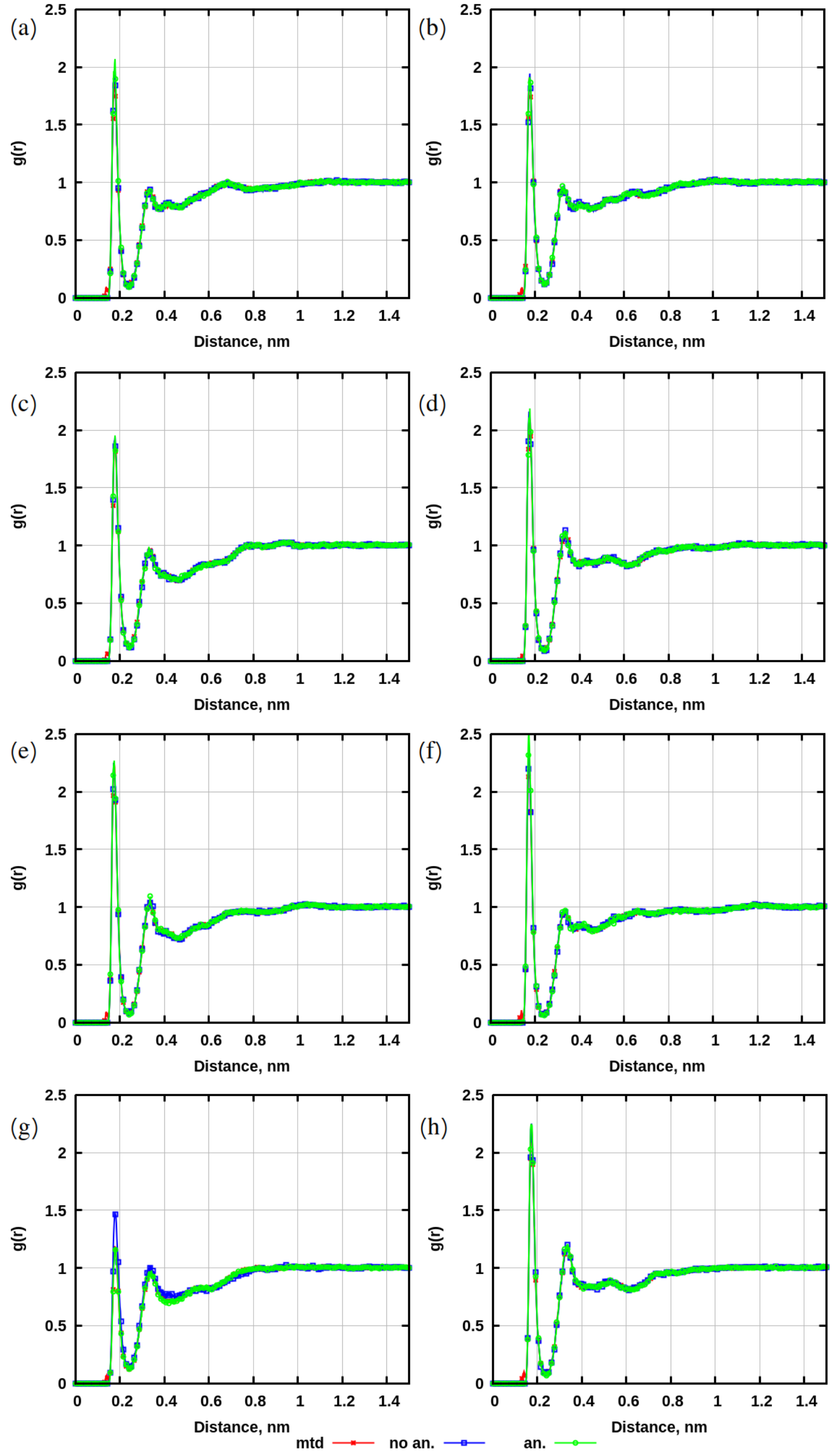

Figure S57 RDFs between hydrogen atoms of sucrose and oxygen atoms of water. (a) H8. (b) H9. (c) H10. (d) H11. (e) H19. (f) H20. (g) H21. (h) H22. Abbreviations: "mtd" - well-tempered metadynamics, "no an." - system simulated without pre-heating, "an." - system simulated with pre-heating.

7.2 Trehalose

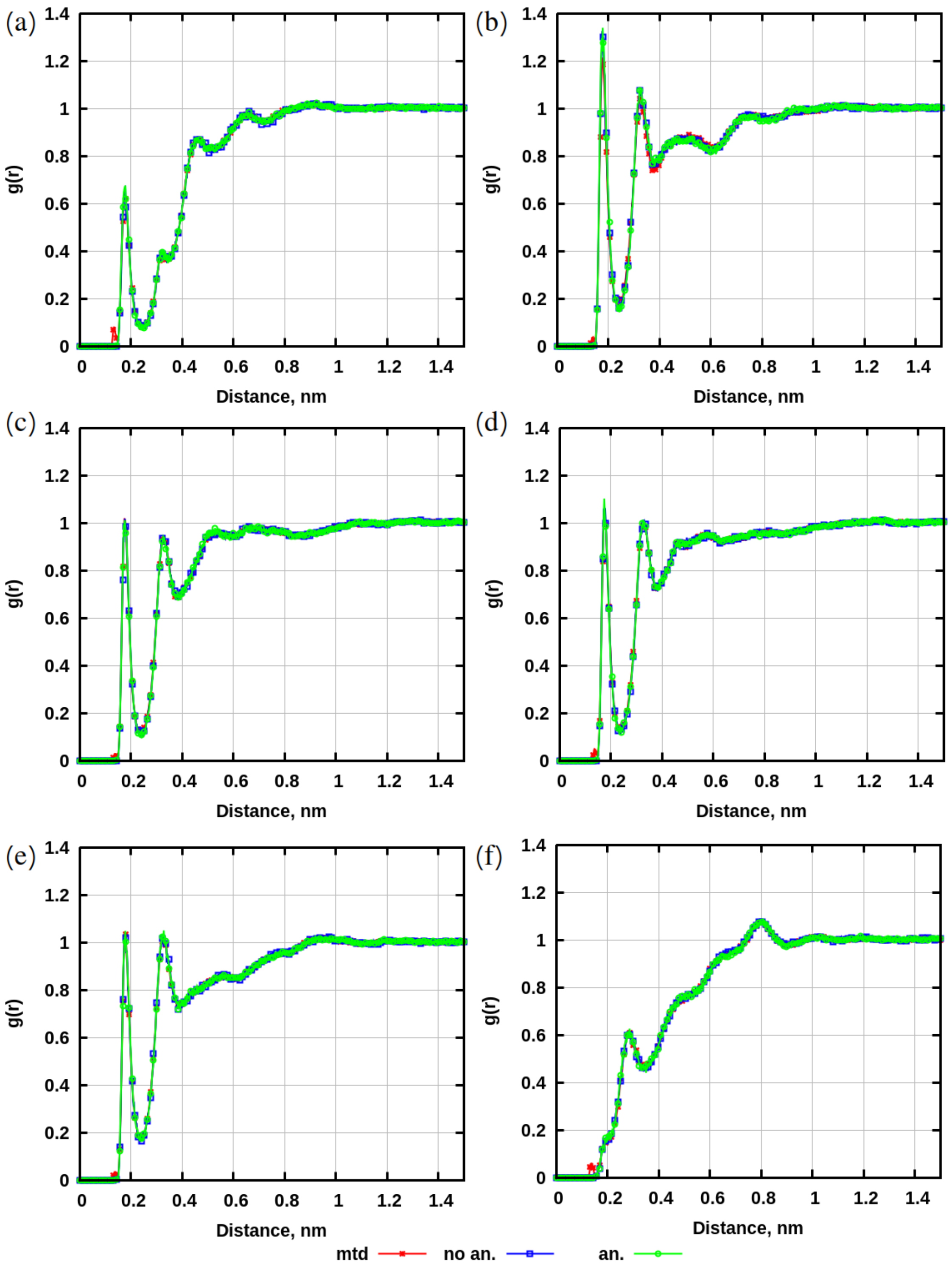

Figure S58 RDFs between oxygen atoms of trehalose and hydrogen atoms of water. (a) O1. (b) O2. (c) O3. (d) O4. (e) O5. (f) O6. Abbreviations: "mtd" - well-tempered metadynamics, "no an." - system simulated without pre-heating, "an." - system simulated with pre-heating.

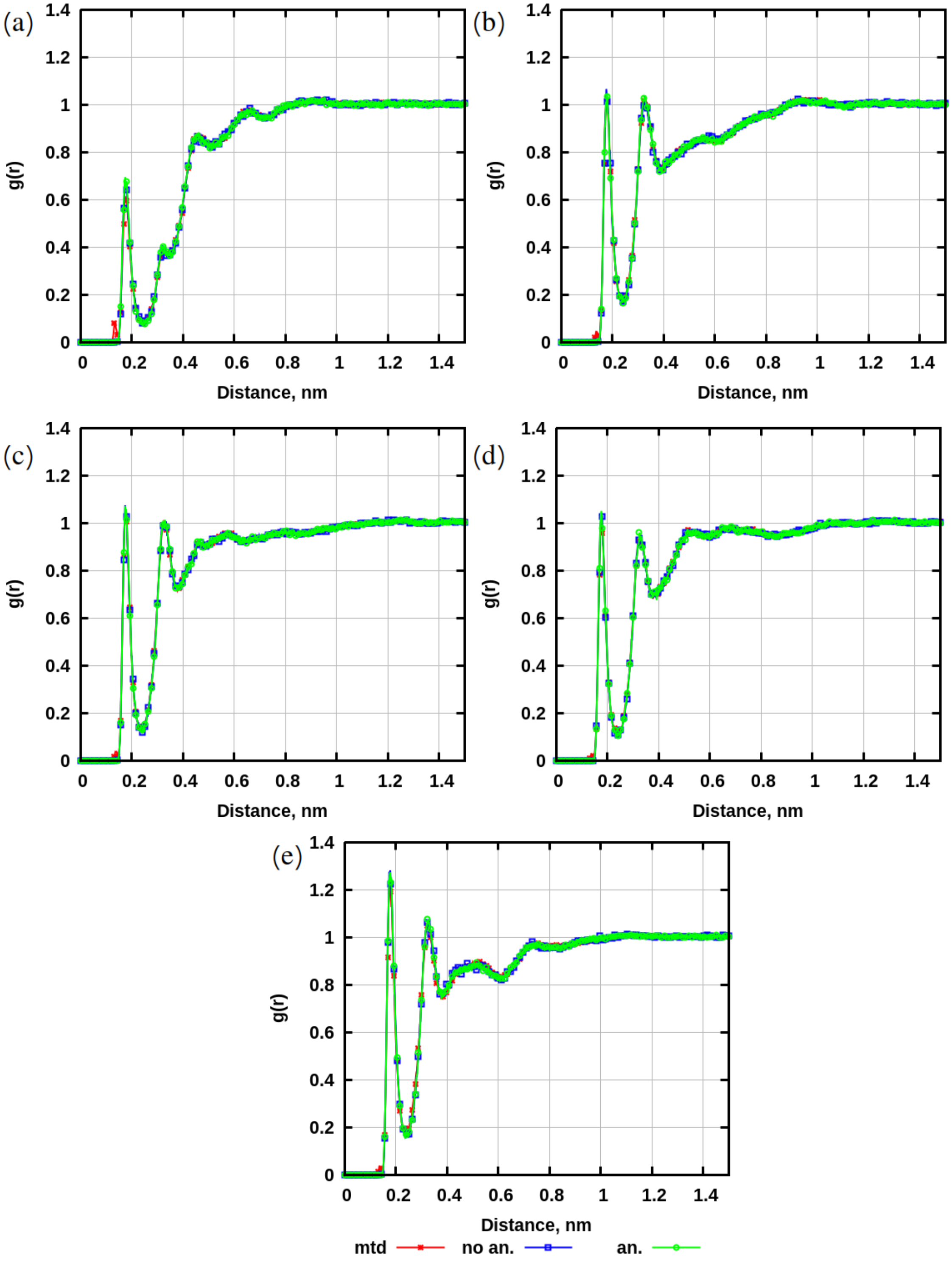

Figure S59 RDFs between oxygen atoms of trehalose and hydrogen atoms of water. (a) O7. (b) O8. (c) O9. (d) O10. (e) O11. Abbreviations: "mtd" - well-tempered metadynamics, "no an." - system simulated without pre-heating, "an." - system simulated with pre-heating.

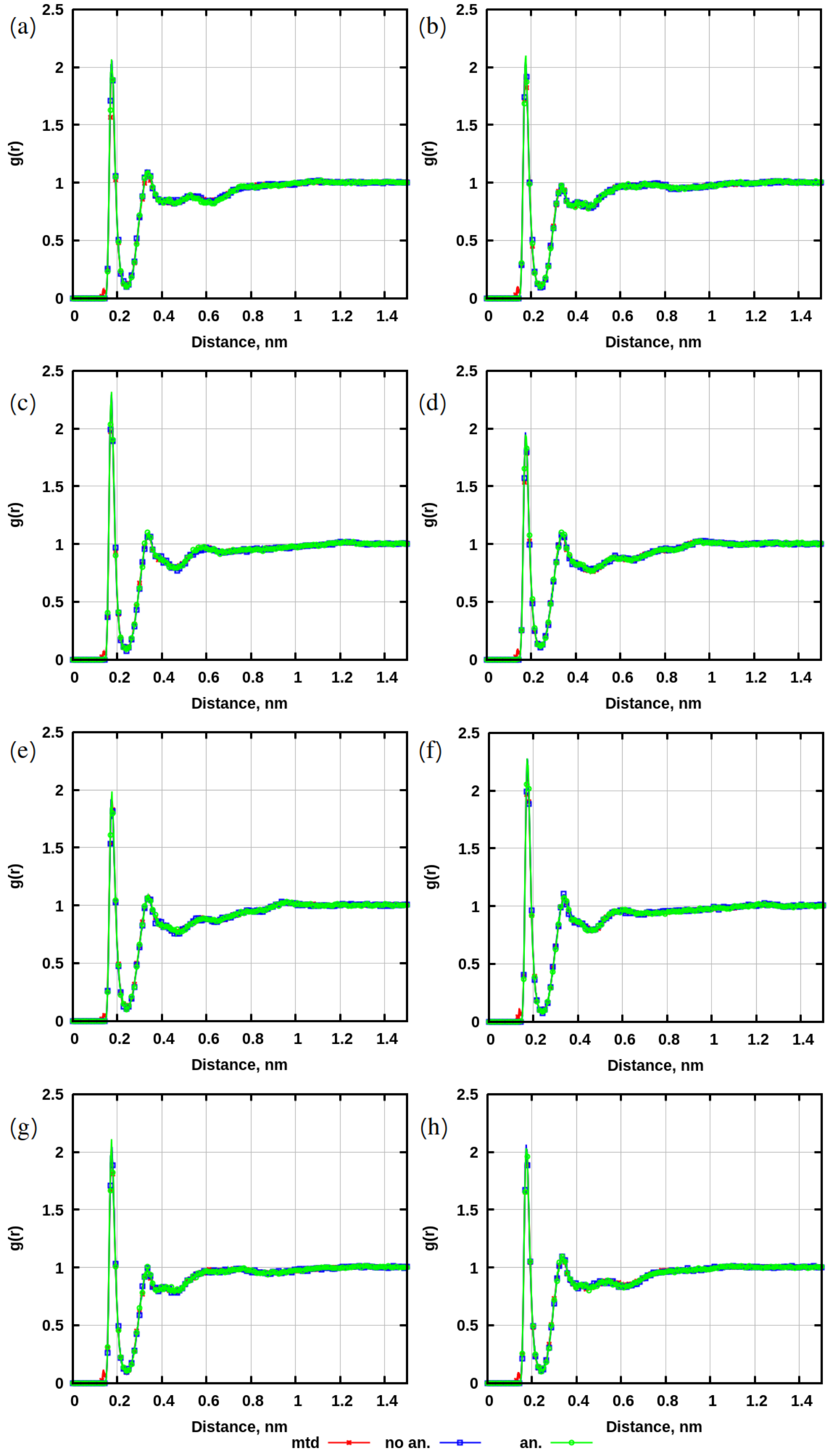

Figure S60 RDFs between hydrogen atoms of trehalose and oxygen atoms of water. (a) H8. (b) H9. (c) H10. (d) H11. (e) H19. (f) H20. (g) H21. (h) H22. Abbreviations: "mtd" - well-tempered metadynamics, "no an." - system simulated without pre-heating, "an." - system simulated with pre-heating.

8 Comparison of dihedrals for systems with 1 molecule of sugar: free energy calculations vs MD

8.1 Sucrose

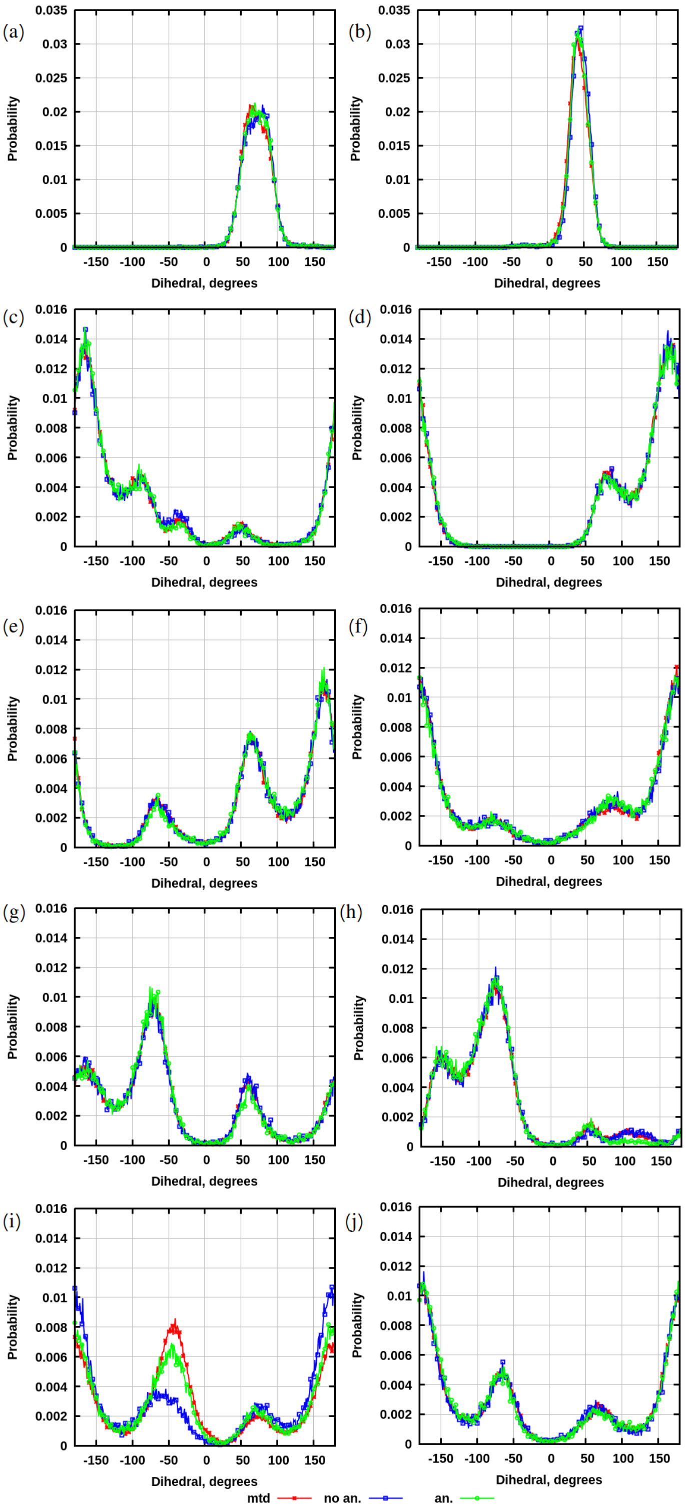

Figure S61 Distribution of dihedrals for simulations with single sucrose in water. Here the calculations were done for the group O1. (a) O1-C1-O5-C7. (b) O7-C7-O5-C1. (c) C5-C4-O2-H8. (d) C4-C3-O3-H9. (e) C3-C2-O4-H10. (f) C5-C6-O6-H11. (g) C9-C8-O8-H19. (h) C8-C9-O9-H20. (i) C7-C11-O10-H21. (j) C10-C12-O11-H22. Abbreviations: "mtd" - well-tempered metadynamics, "no an." - system simulated without pre-heating, "an." - system simulated with pre-heating.

,

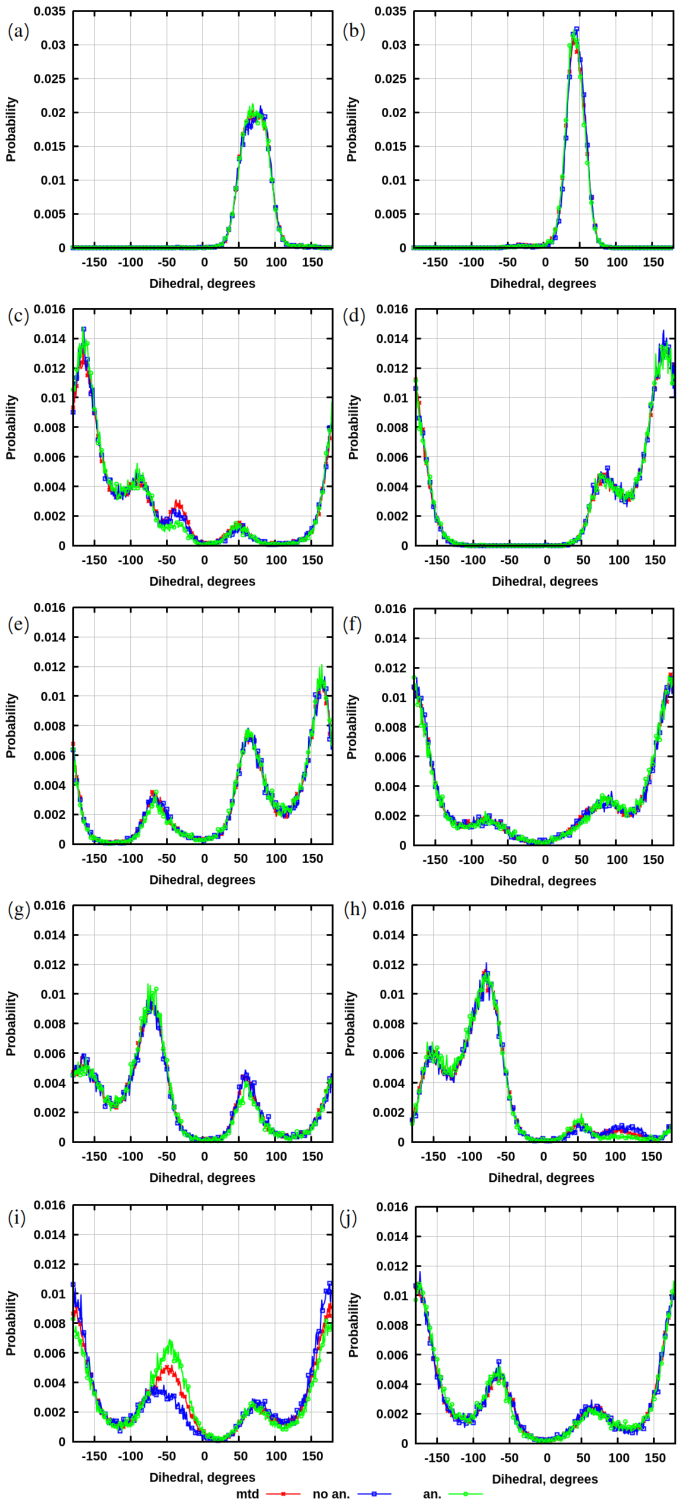

Figure S62 Distribution of dihedrals for simulations with single sucrose in water. Here the calculations were done for the group O2-H8. (a) O1-C1-O5-C7. (b) O7-C7-O5-C1. (c) C5-C4-O2-H8. (d) C4-C3-O3-H9. (e) C3-C2-O4-H10. (f) C5-C6-O6-H11. (g) C9-C8-O8-H19. (h) C8-C9-O9-H20. (i) C7-C11-O10-H21. (j) C10-C12-O11-H22. Abbreviations: "mtd" - well-tempered metadynamics, "no an." - system simulated without pre-heating, "an." - system simulated with pre-heating.

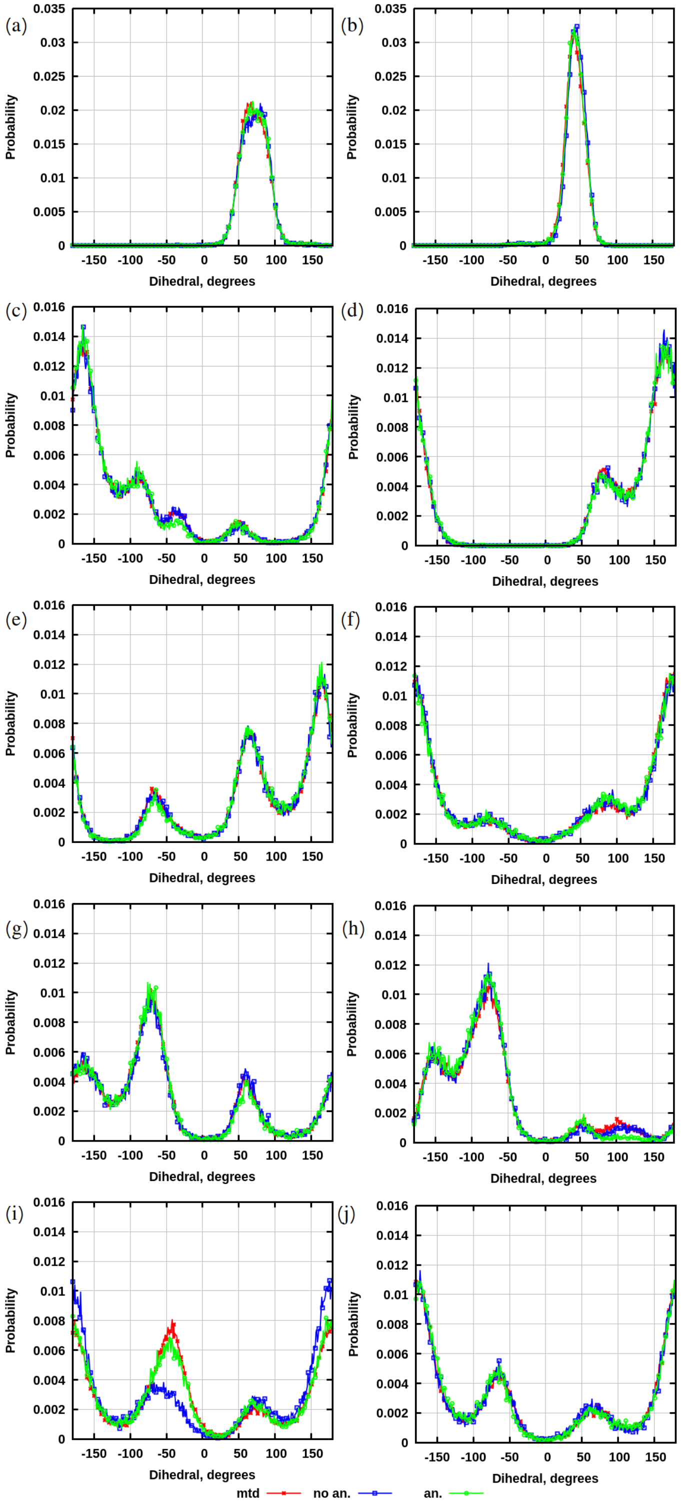

Figure S63 Distribution of dihedrals for simulations with single sucrose in water. Here the calculations were done for the group O3-H9. (a) O1-C1-O5-C7. (b) O7-C7-O5-C1. (c) C5-C4-O2-H8. (d) C4-C3-O3-H9. (e) C3-C2-O4-H10. (f) C5-C6-O6-H11. (g) C9-C8-O8-H19. (h) C8-C9-O9-H20. (i) C7-C11-O10-H21. (j) C10-C12-O11-H22. Abbreviations: "mtd" - well-tempered metadynamics, "no an." - system simulated without pre-heating, "an." - system simulated with pre-heating.

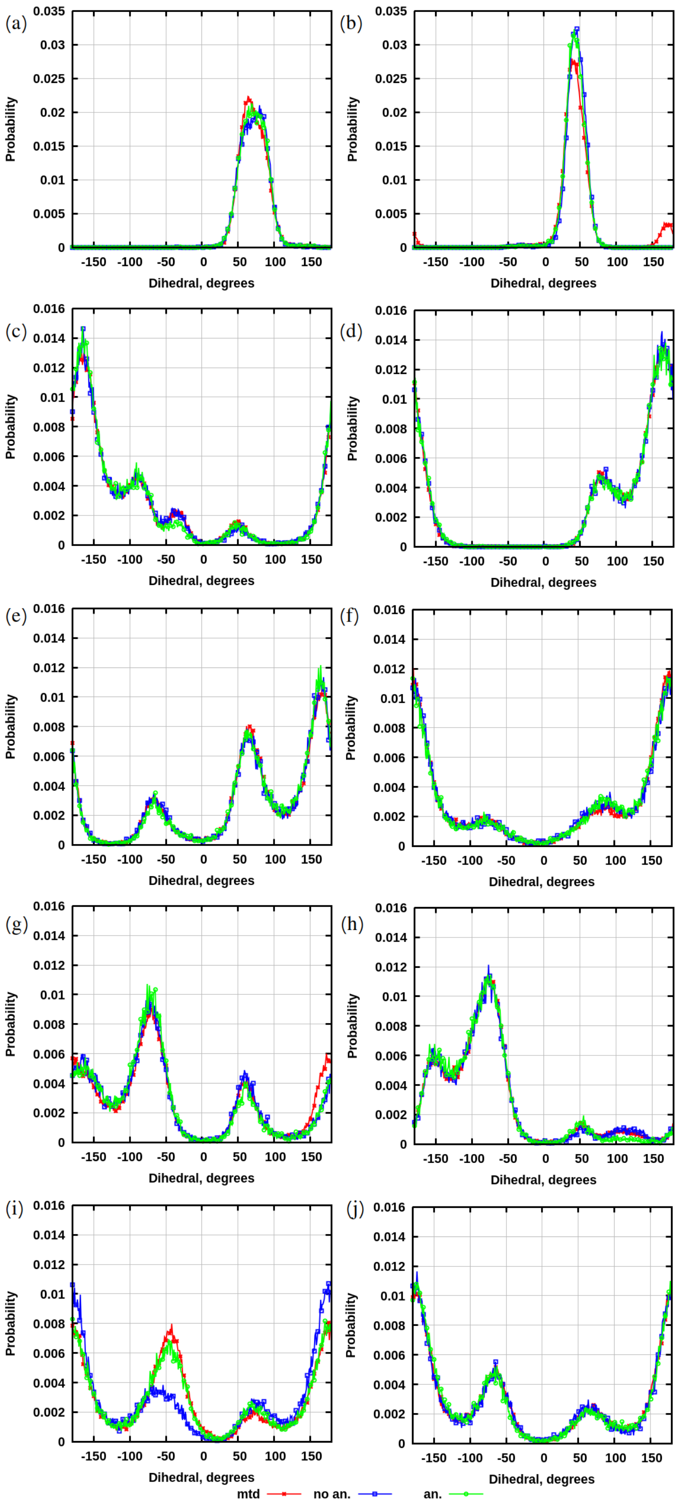

Figure S64 Distribution of dihedrals for simulations with single sucrose in water. Here the calculations were done for the group O4-H10. (a) O1-C1-O5-C7. (b) O7-C7-O5-C1. (c) C5-C4-O2-H8. (d) C4-C3-O3-H9. (e) C3-C2-O4-H10. (f) C5-C6-O6-H11. (g) C9-C8-O8-H19. (h) C8-C9-O9-H20. (i) C7-C11-O10-H21. (j) C10-C12-O11-H22. Abbreviations: "mtd" - well-tempered metadynamics, "no an." - system simulated without pre-heating, "an." - system simulated with pre-heating.

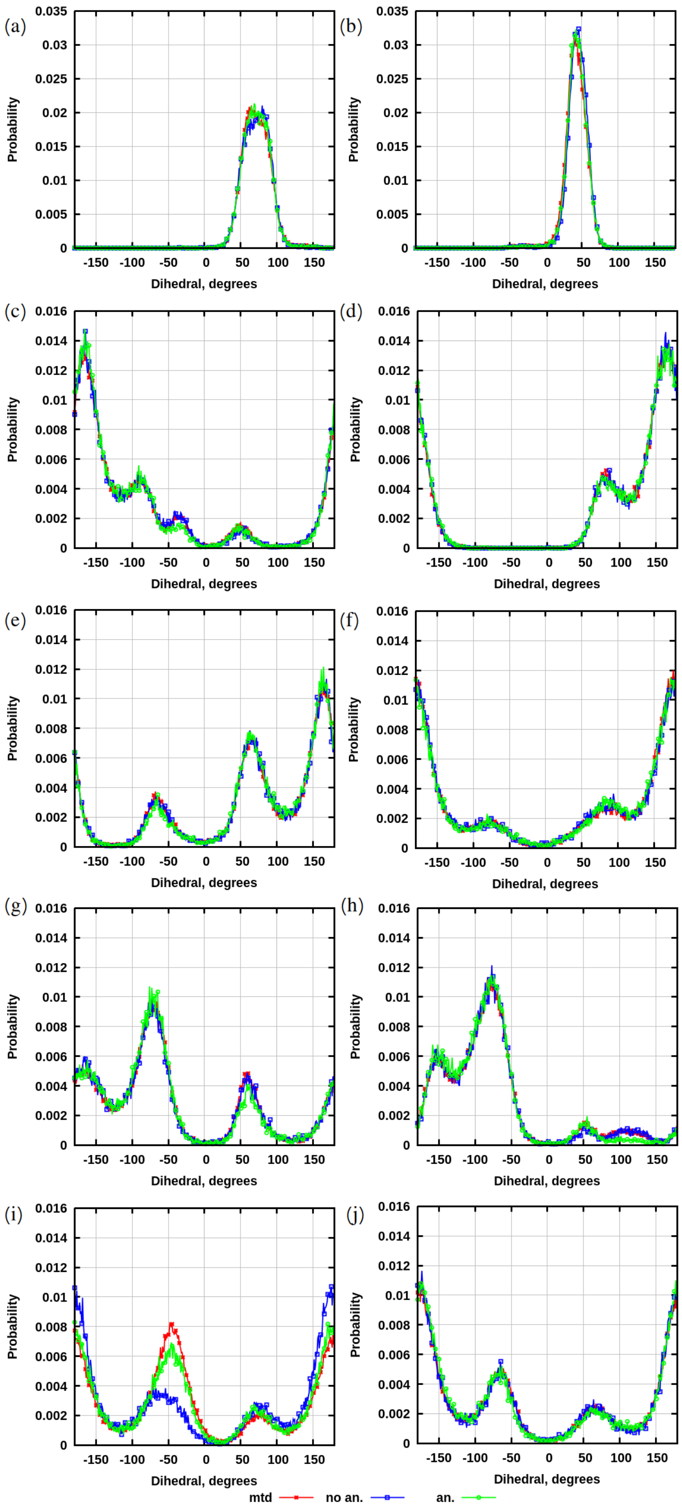

Figure S65 Distribution of dihedrals for simulations with single sucrose in water. Here the calculations were done for the group O5. (a) O1-C1-O5-C7. (b) O7-C7-O5-C1. (c) C5-C4-O2-H8. (d) C4-C3-O3-H9. (e) C3-C2-O4-H10. (f) C5-C6-O6-H11. (g) C9-C8-O8-H19. (h) C8-C9-O9-H20. (i) C7-C11-O10-H21. (j) C10-C12-O11-H22. Abbreviations: "mtd" - well-tempered metadynamics, "no an." - system simulated without pre-heating, "an." - system simulated with pre-heating.

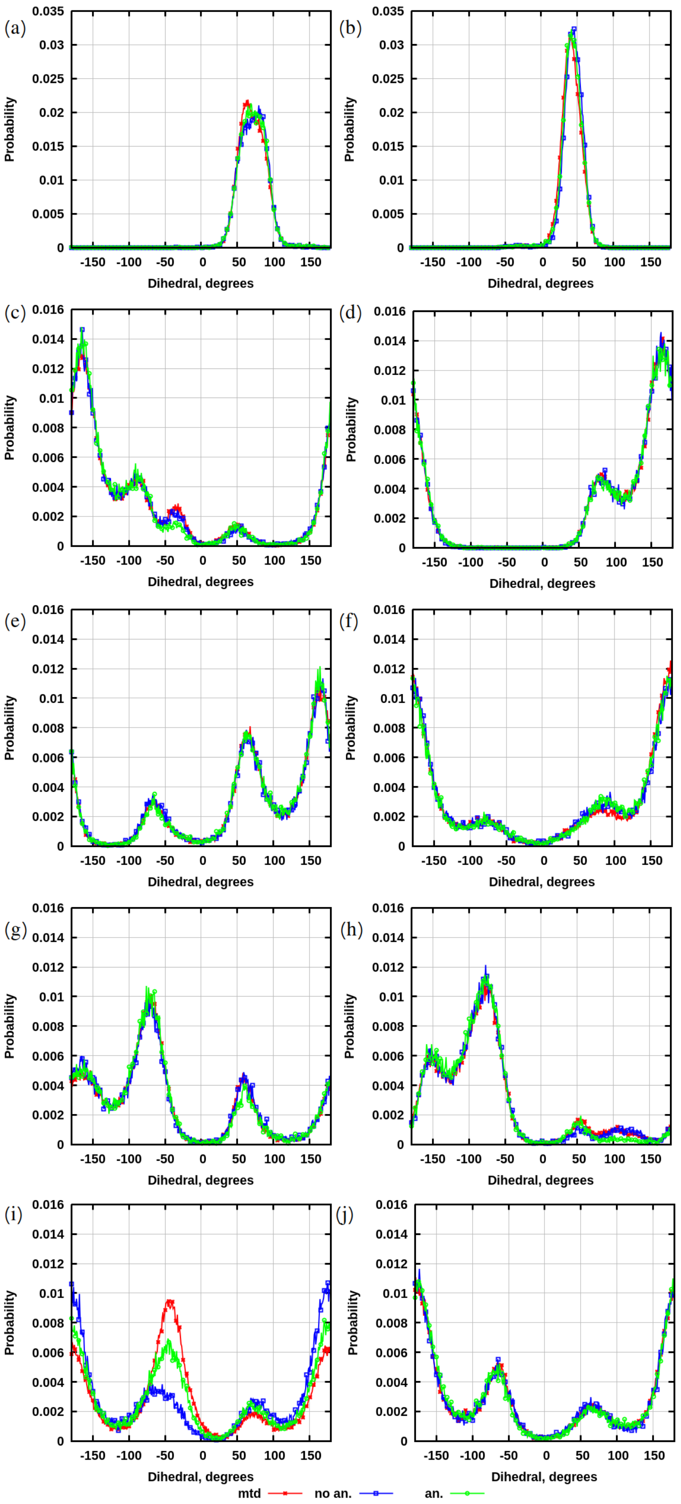

Figure S66 Distribution of dihedrals for simulations with single sucrose in water. Here the calculations were done for the group O6-H11. (a) O1-C1-O5-C7. (b) O7-C7-O5-C1. (c) C5-C4-O2-H8. (d) C4-C3-O3-H9. (e) C3-C2-O4-H10. (f) C5-C6-O6-H11. (g) C9-C8-O8-H19. (h) C8-C9-O9-H20. (i) C7-C11-O10-H21. (j) C10-C12-O11-H22. Abbreviations: "mtd" - well-tempered metadynamics, "no an." - system simulated without pre-heating, "an." - system simulated with pre-heating.

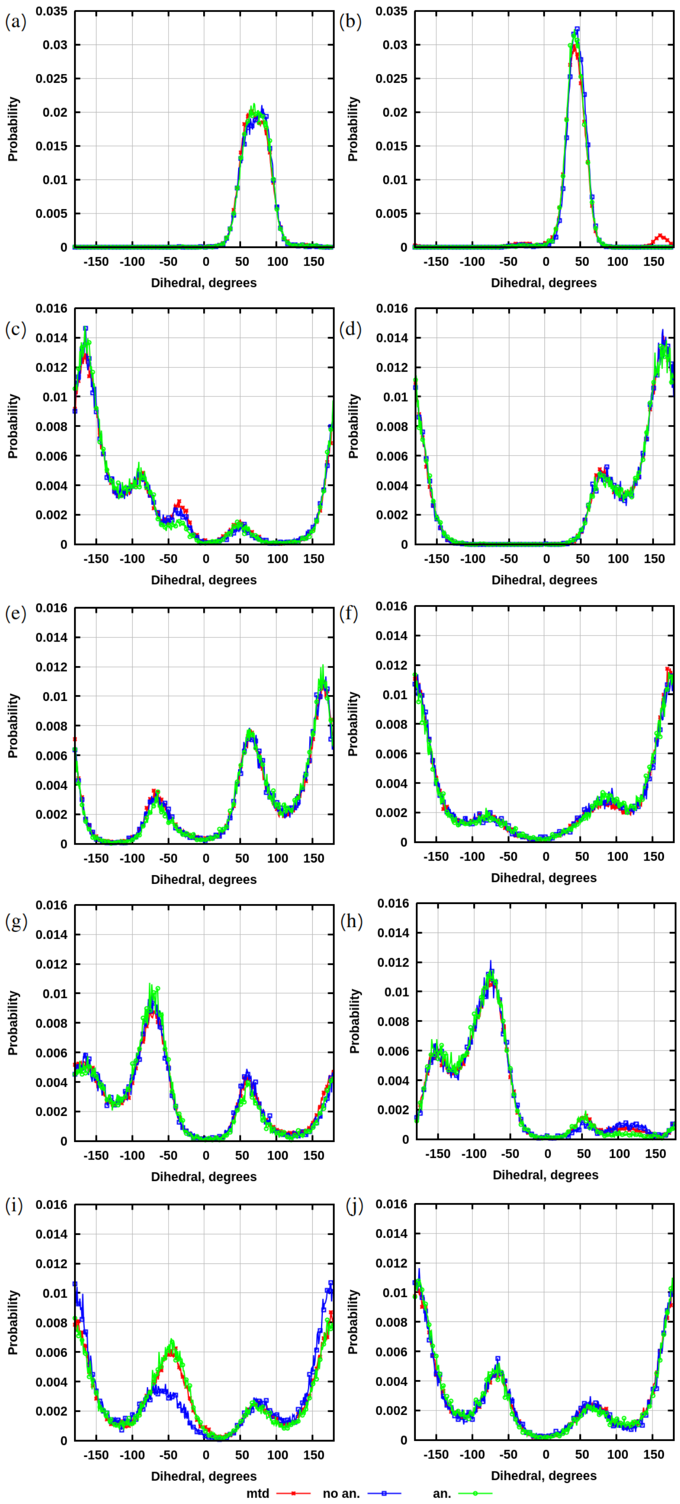

Figure S67 Distribution of dihedrals for simulations with single sucrose in water. Here the calculations were done for the group O7. (a) O1-C1-O5-C7. (b) O7-C7-O5-C1. (c) C5-C4-O2-H8. (d) C4-C3-O3-H9. (e) C3-C2-O4-H10. (f) C5-C6-O6-H11. (g) C9-C8-O8-H19. (h) C8-C9-O9-H20. (i) C7-C11-O10-H21. (j) C10-C12-O11-H22. Abbreviations: "mtd" - well-tempered metadynamics, "no an." - system simulated without pre-heating, "an." - system simulated with pre-heating.

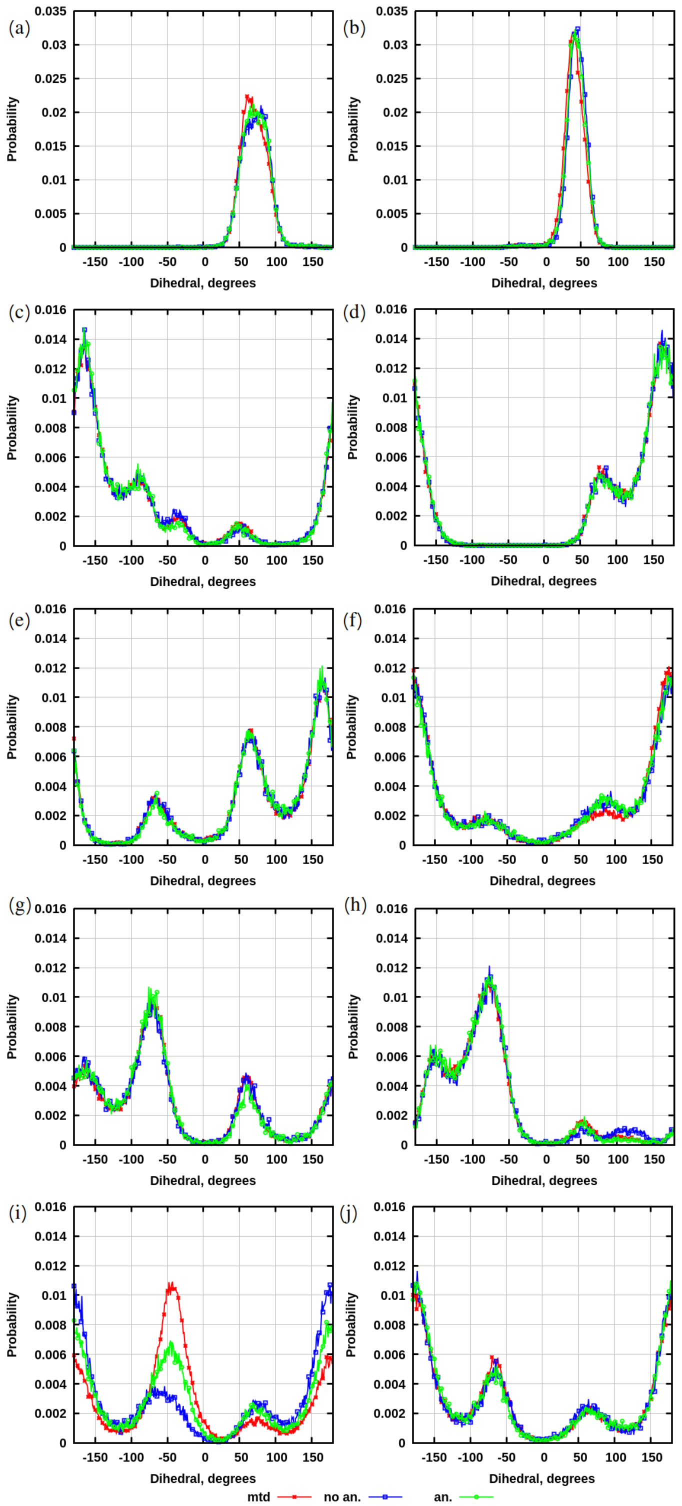

Figure S68 Distribution of dihedrals for simulations with single sucrose in water. Here the calculations were done for the group O8-H19. (a) O1-C1-O5-C7. (b) O7-C7-O5-C1. (c) C5-C4-O2-H8. (d) C4-C3-O3-H9. (e) C3-C2-O4-H10. (f) C5-C6-O6-H11. (g) C9-C8-O8-H19. (h) C8-C9-O9-H20. (i) C7-C11-O10-H21. (j) C10-C12-O11-H22. Abbreviations: "mtd" - well-tempered metadynamics, "no an." - system simulated without pre-heating, "an." - system simulated with pre-heating.

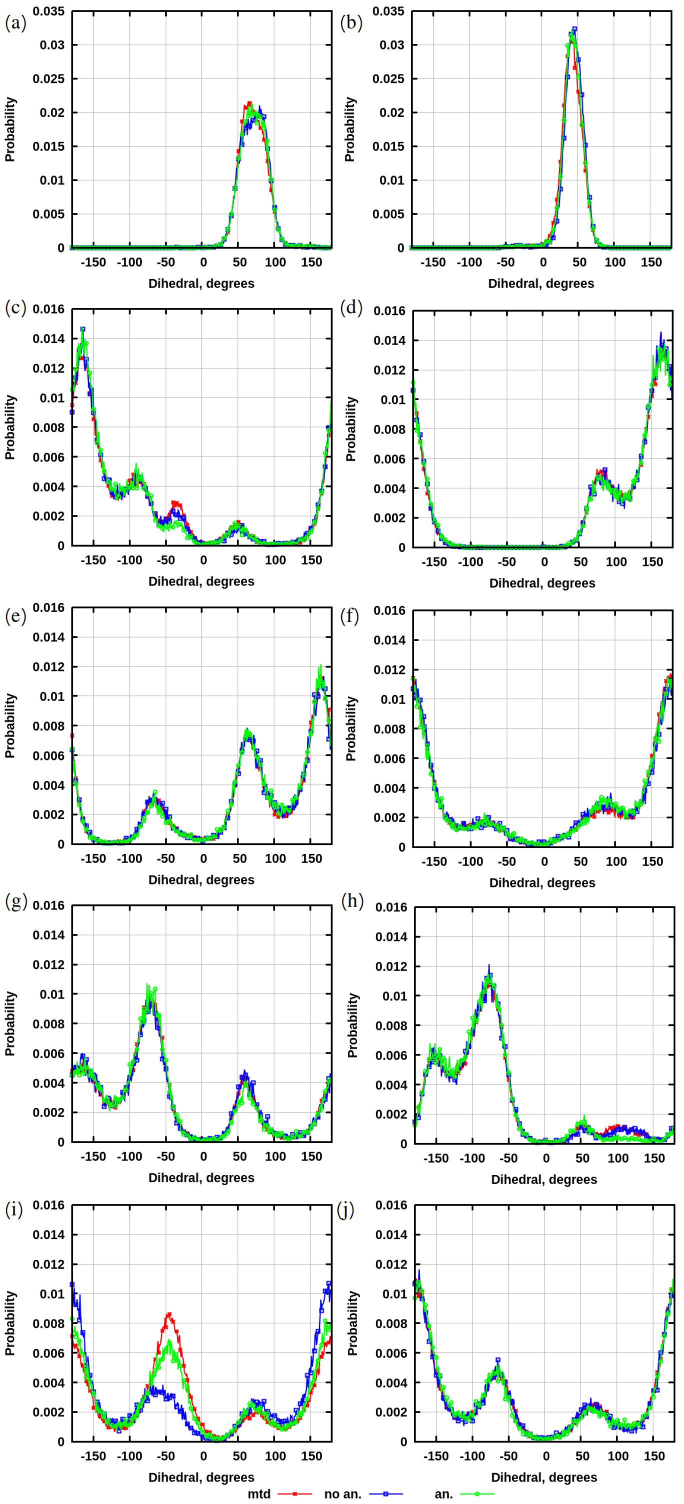

Figure S69 Distribution of dihedrals for simulations with single sucrose in water. Here the calculations were done for the group O9-H20. (a) O1-C1-O5-C7. (b) O7-C7-O5-C1. (c) C5-C4-O2-H8. (d) C4-C3-O3-H9. (e) C3-C2-O4-H10. (f) C5-C6-O6-H11. (g) C9-C8-O8-H19. (h) C8-C9-O9-H20. (i) C7-C11-O10-H21. (j) C10-C12-O11-H22. Abbreviations: "mtd" - well-tempered metadynamics, "no an." - system simulated without pre-heating, "an." - system simulated with pre-heating.

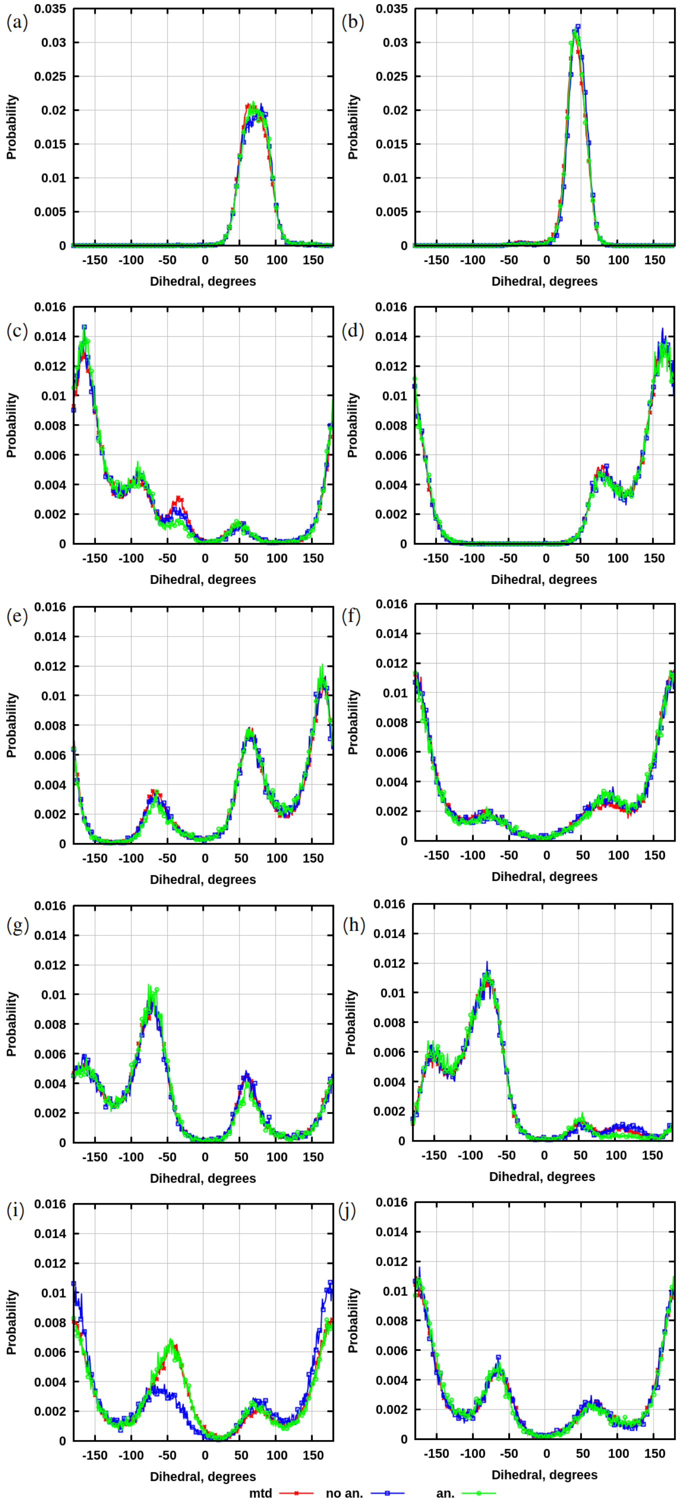

Figure S70 Distribution of dihedrals for simulations with single sucrose in water. Here the calculations were done for the group O10-H21. (a) O1-C1-O5-C7. (b) O7-C7-O5-C1. (c) C5-C4-O2-H8. (d) C4-C3-O3-H9. (e) C3-C2-O4-H10. (f) C5-C6-O6-H11. (g) C9-C8-O8-H19. (h) C8-C9-O9-H20. (i) C7-C11-O10-H21. (j) C10-C12-O11-H22. Abbreviations: "mtd" - well-tempered metadynamics, "no an." - system simulated without pre-heating, "an." - system simulated with pre-heating.

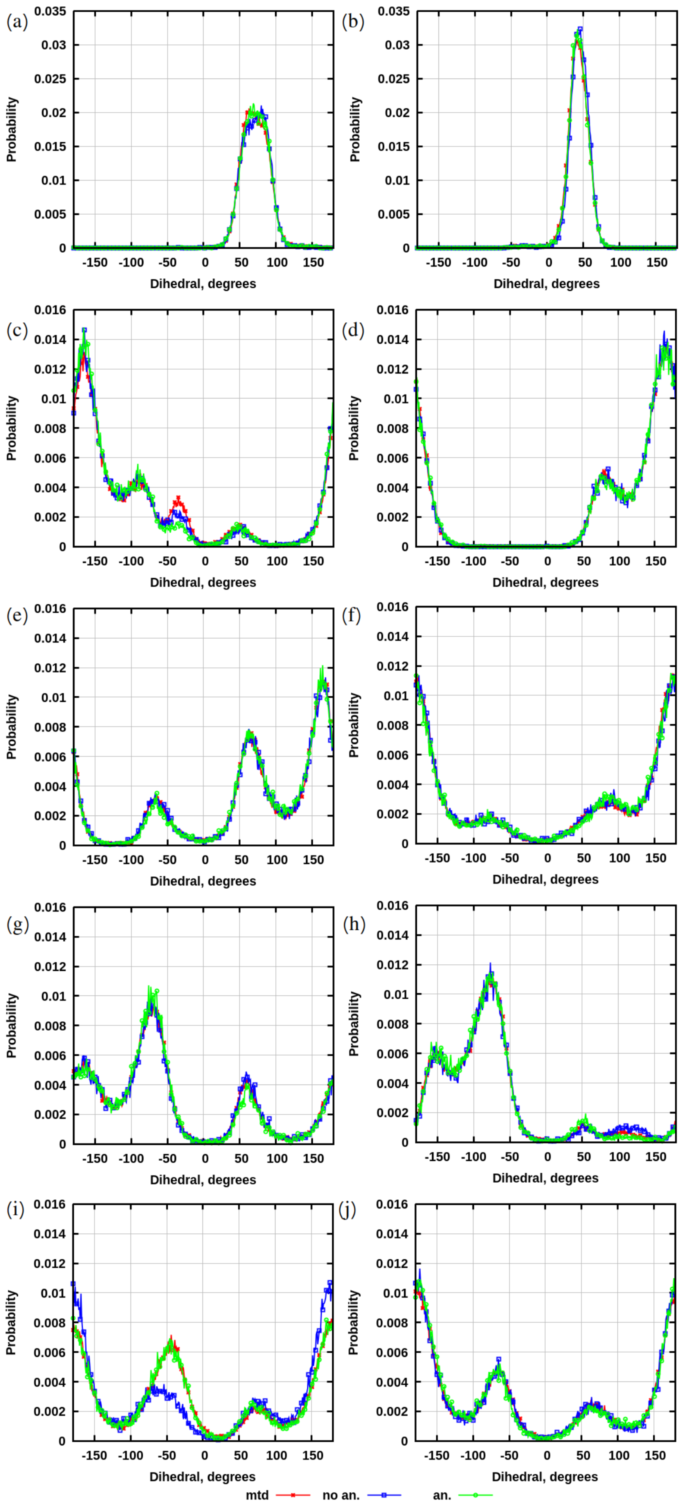

Figure S71 Distribution of dihedrals for simulations with single sucrose in water. Here the calculations were done for the group O11-H22. (a) O1-C1-O5-C7. (b) O7-C7-O5-C1. (c) C5-C4-O2-H8. (d) C4-C3-O3-H9. (e) C3-C2-O4-H10. (f) C5-C6-O6-H11. (g) C9-C8-O8-H19. (h) C8-C9-O9-H20. (i) C7-C11-O10-H21. (j) C10-C12-O11-H22. Abbreviations: "mtd" - well-tempered metadynamics, "no an." - system simulated without pre-heating, "an." - system simulated with pre-heating.

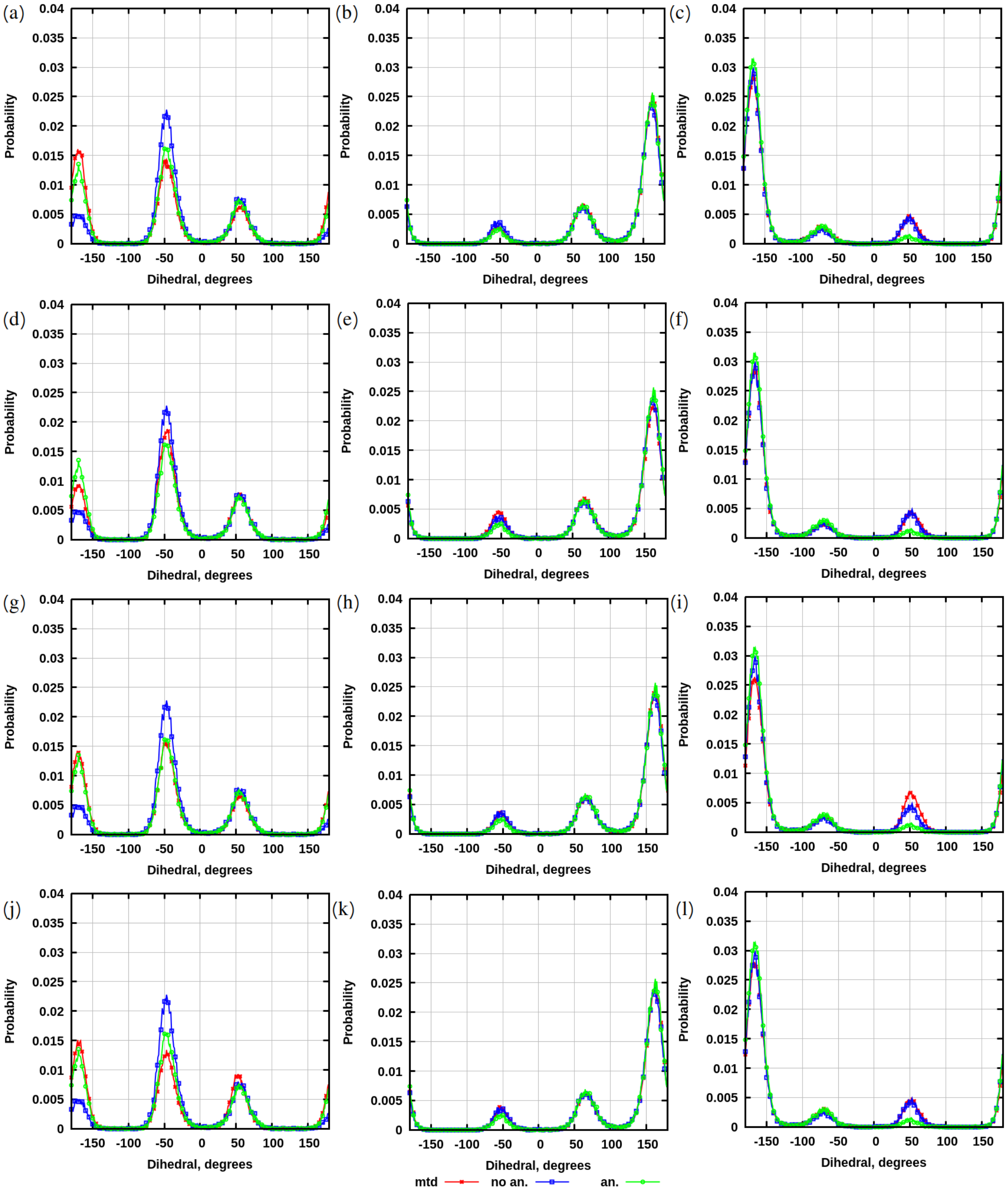

Figure S72 Distribution of dihedrals for simulations with single sucrose in water. (a) C8-C7-C11-O10, group O1. (b) C4-C5-C6-O6, group O1. (c) C9-C10-C12-O11, group O1. (d) C8-C7-C11-O10, group O2-H8. (e) C4-C5-C6-O6, group O2-H8. (f) C9-C10-C12-O11, group O2-H8. (g) C8-C7-C11-O10, group O3-H9. (h) C4-C5-C6-O6, group O3-H9. (i) C9-C10-C12-O11, group O3-H9. (j) C8-C7-C11-O10, group O4-H10. (k) C4-C5-C6-O6, group O4-H10. (l) C9-C10-C12-O11, group O4-H10. Abbreviations: "mtd" - well-tempered metadynamics, "no an." - system simulated without pre-heating, "an." - system simulated with pre-heating.

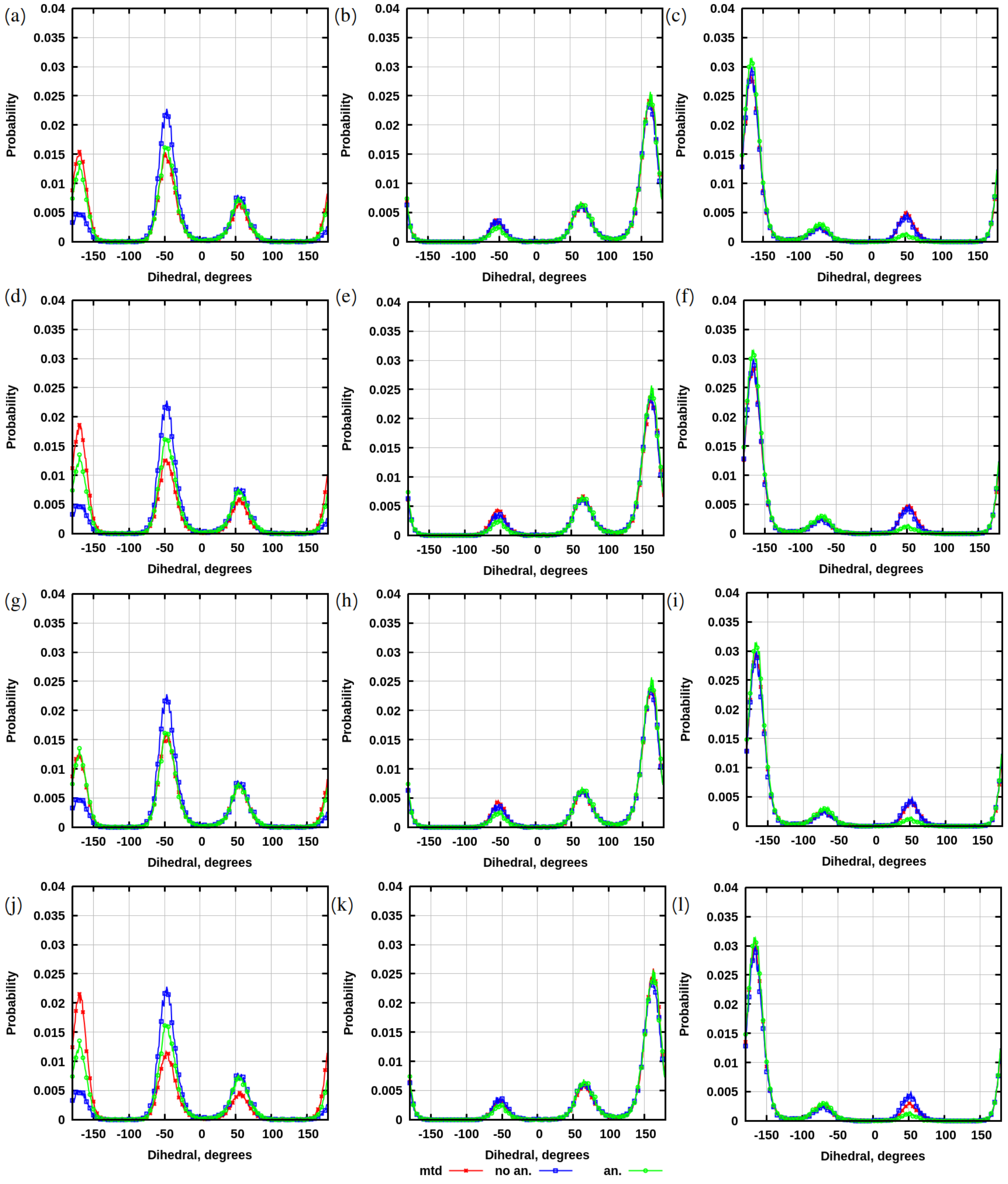

Figure S73 Distribution of dihedrals for simulations with single sucrose in water. (a) C8-C7-C11-O10, group O5. (b) C4-C5-C6-O6, group O5. (c) C9-C10-C12-O11, group O5. (d) C8-C7-C11-O10, group O6-H11. (e) C4-C5-C6-O6, group O6-H11. (f) C9-C10-C12-O11, group O6-H11. (g) C8-C7-C11-O10, group O7. (h) C4-C5-C6-O6, group O7. (i) C9-C10-C12-O11, group O7. (j) C8-C7-C11-O10, group O8-H19. (k) C4-C5-C6-O6, group O8-H19. (l) C9-C10-C12-O11, group O8-H19. Abbreviations: "mtd" - well-tempered metadynamics, "no an." - system simulated without pre-heating, "an." - system simulated with pre-heating.

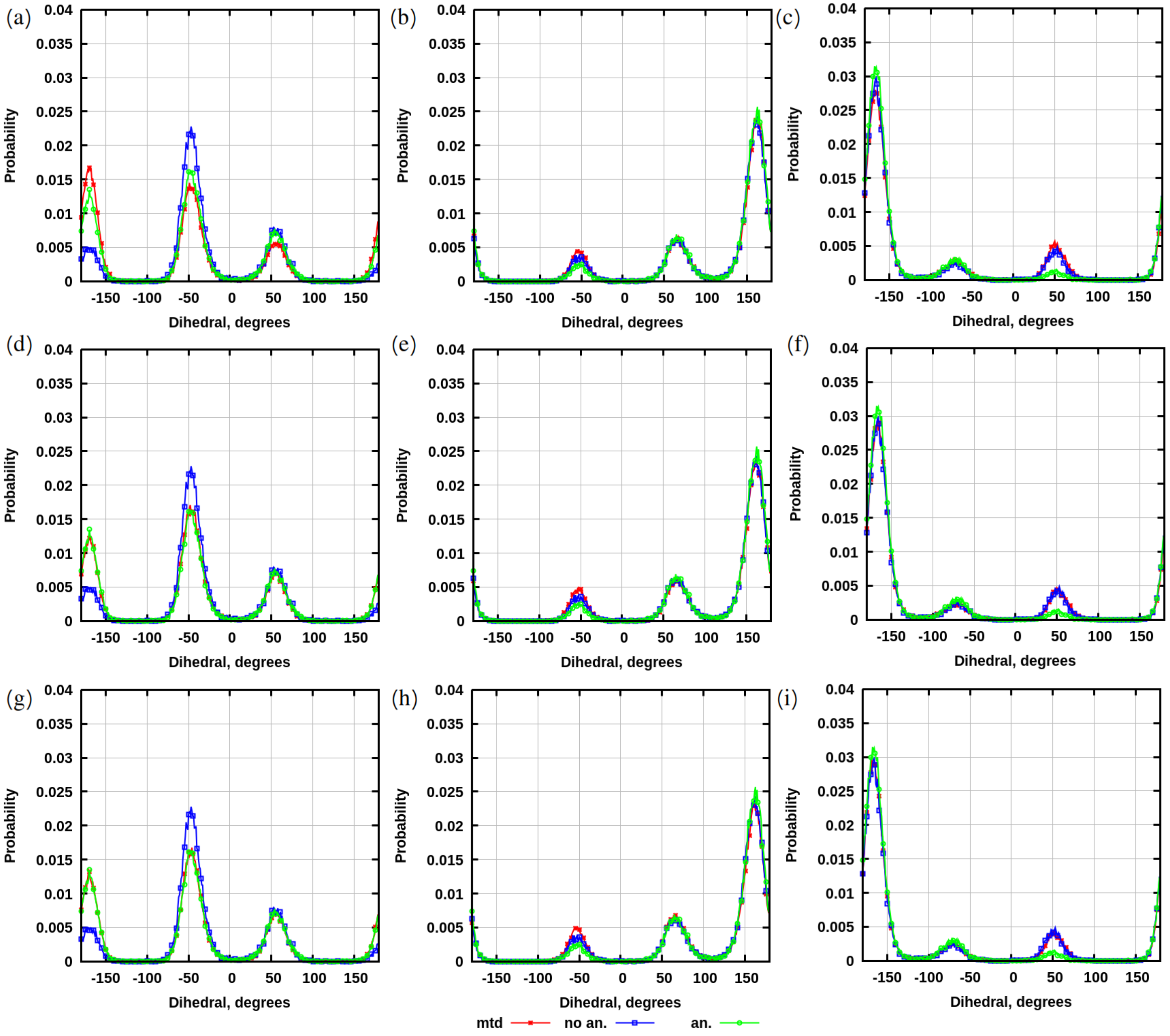

Figure S74 Distribution of dihedrals for simulations with single sucrose in water. Here the calculations were done for the group O11-H22. (a) C8-C7-C11-O10, group O9-H20. (b) C4-C5-C6-O6, group O9-H20. (c) C9-C10-C12-O11, group O9-H20. (d) C8-C7-C11-O10, group O10-H21. (e) C4-C5-C6-O6, group O10-H21. (f) C9-C10-C12-O11, group O10-H21. (g) C8-C7-C11-O10, group O11-H22. (h) C4-C5-C6-O6, group O11-H22. (i) C9-C10-C12-O11, group O11-H22. Abbreviations: "mtd" - well-tempered metadynamics, "no an." - system simulated without pre-heating, "an." - system simulated with pre-heating.

8.2 Trehalose

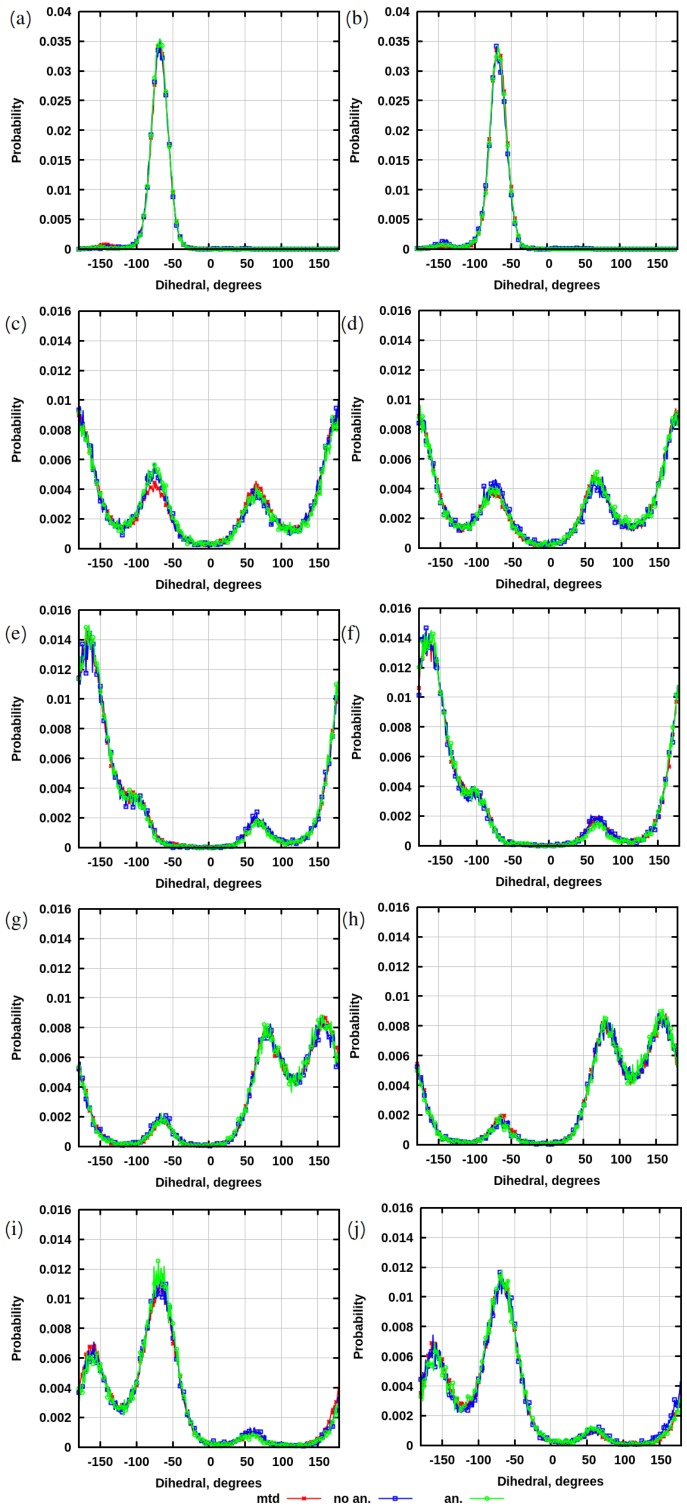

Figure S75 Distribution of dihedrals for simulations with single trehalose in water. Here the calculations were done for the group O1. (a) O1-C6-O6-C7. (b) O7-C7-O6-C6. (c) C2-C1-O2-H8. (d) C11-C12-O11-H22. (e) C2-C3-O3-H9. (f) C11-C10-O10-H21. (g) C3-C4-O4-H10. (h) C10-C9-O9-H20. (i) C4-C5-O5-H11. (j) C9-C8-O8-H19. Abbreviations: "mtd" - well-tempered metadynamics, "no an." - system simulated without pre-heating, "an." - system simulated with pre-heating.

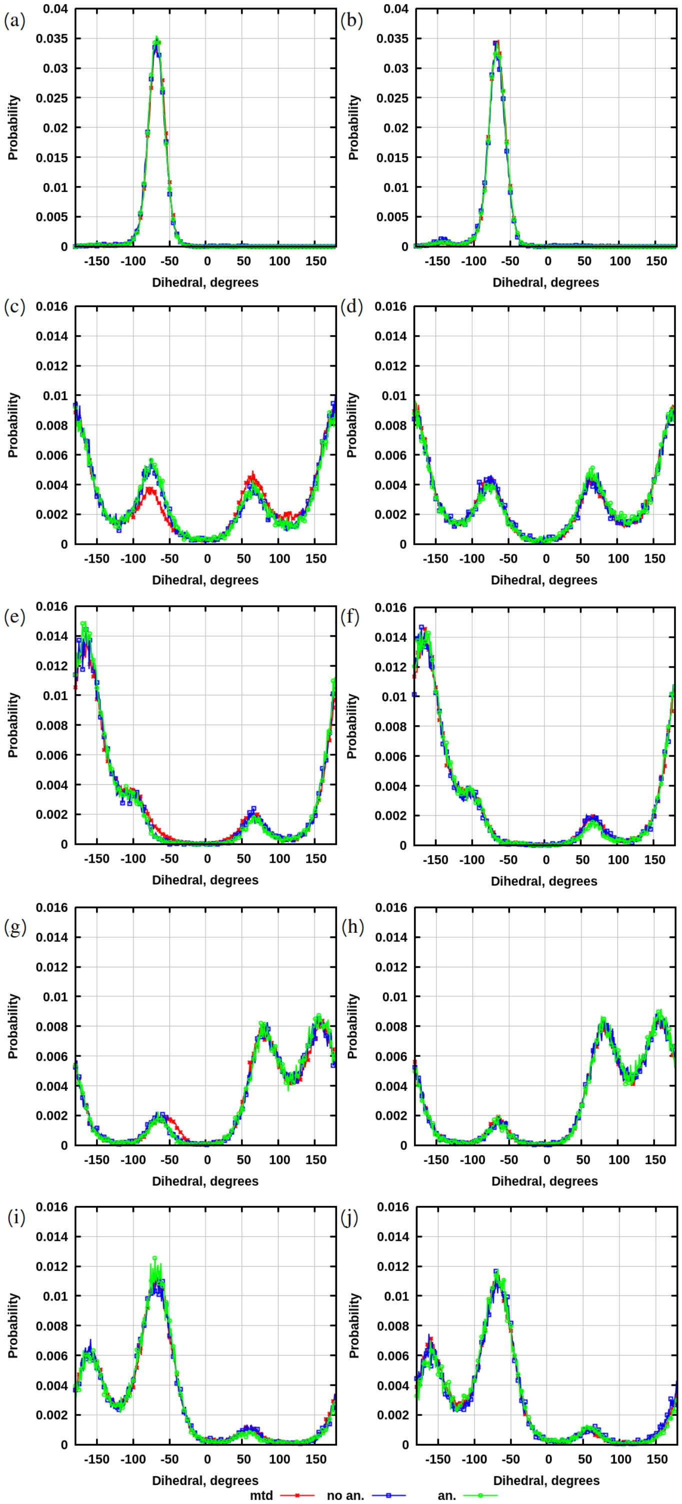

Figure S76 Distribution of dihedrals for simulations with single trehalose in water. Here the calculations were done for the group O2-H8. (a) O1-C6-O6-C7. (b) O7-C7-O6-C6. (c) C2-C1-O2-H8. (d) C11-C12-O11-H22. (e) C2-C3-O3-H9. (f) C11-C10-O10-H21. (g) C3-C4-O4-H10. (h) C10-C9-O9-H20. (i) C4-C5-O5-H11. (j) C9-C8-O8-H19. Abbreviations: "mtd" - well-tempered metadynamics, "no an." - system simulated without pre-heating, "an." - system simulated with pre-heating.

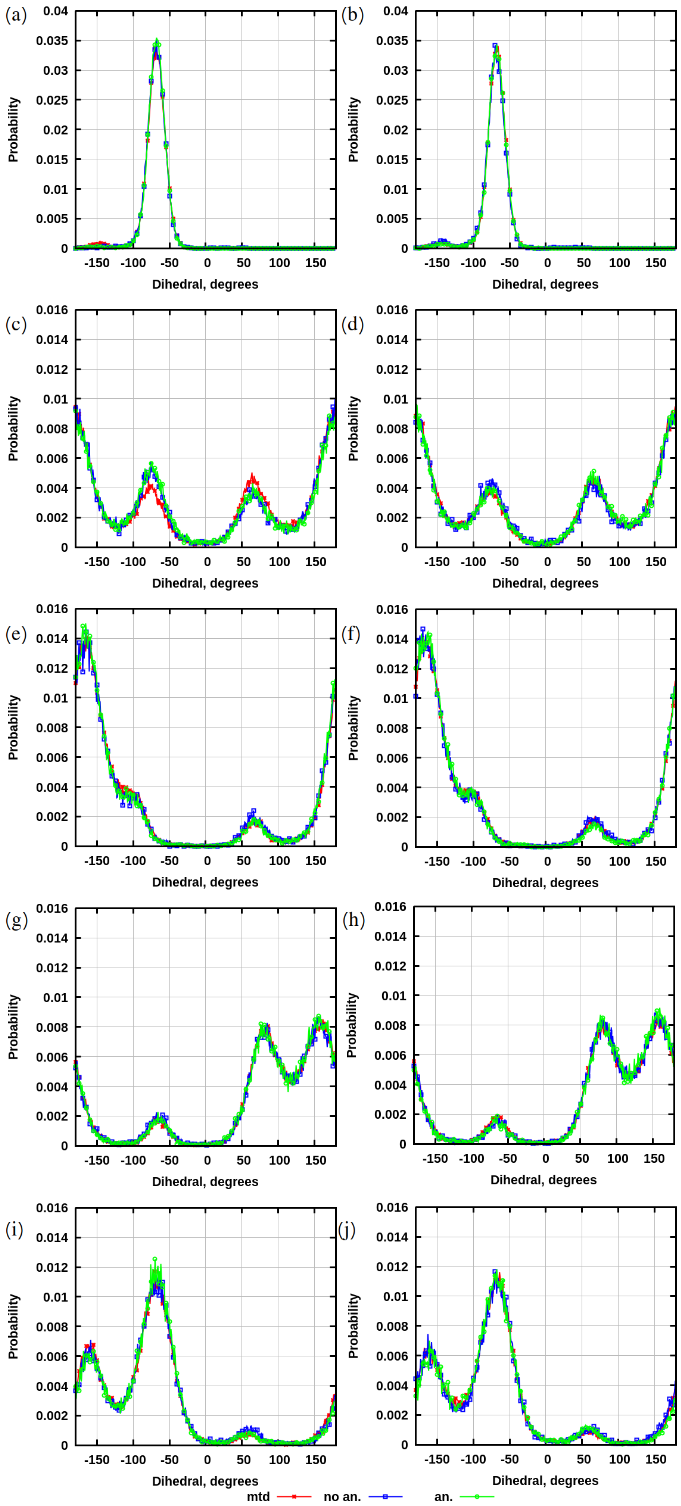

Figure S77 Distribution of dihedrals for simulations with single trehalose in water. Here the calculations were done for the group O3-H9. (a) O1-C6-O6-C7. (b) O7-C7-O6-C6. (c) C2-C1-O2-H8. (d) C11-C12-O11-H22. (e) C2-C3-O3-H9. (f) C11-C10-O10-H21. (g) C3-C4-O4-H10. (h) C10-C9-O9-H20. (i) C4-C5-O5-H11. (j) C9-C8-O8-H19. Abbreviations: "mtd" - well-tempered metadynamics, "no an." - system simulated without pre-heating, "an." - system simulated with pre-heating.

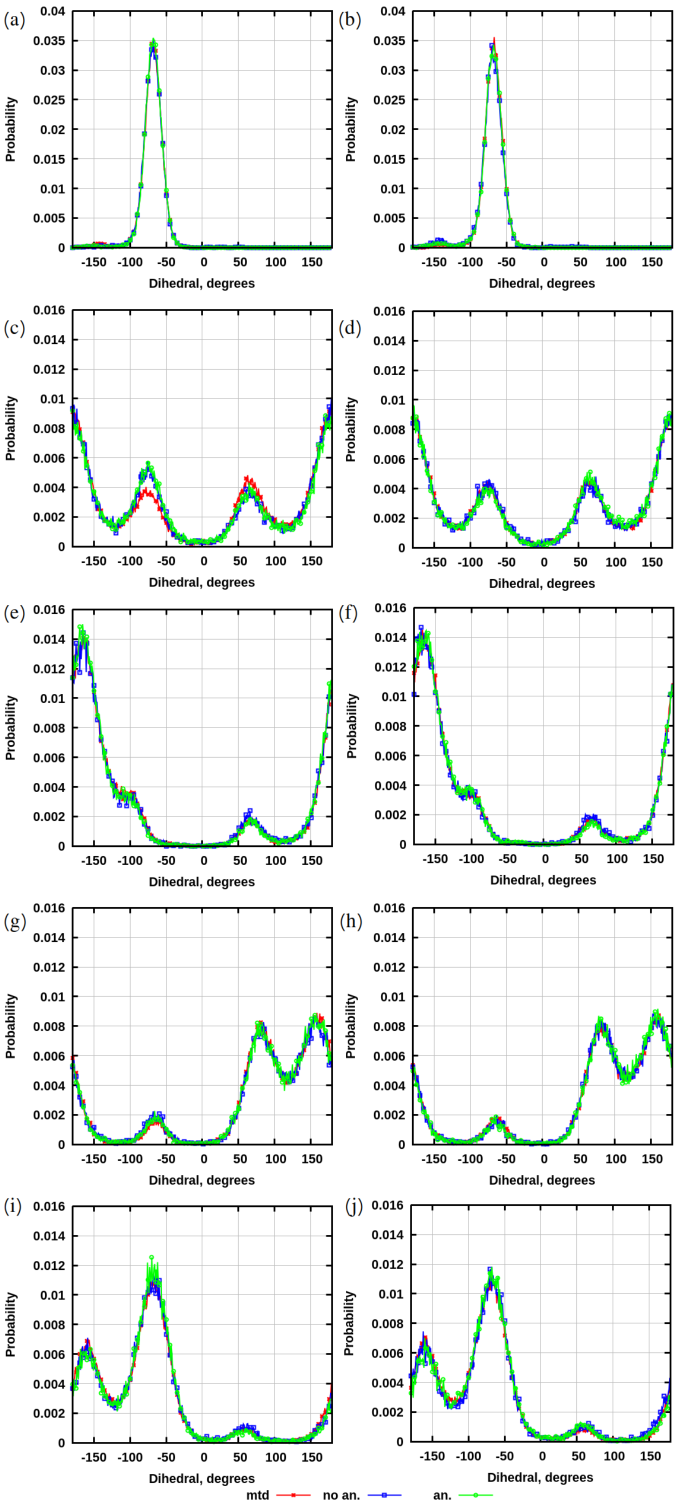

Figure S78 Distribution of dihedrals for simulations with single trehalose in water. Here the calculations were done for the group O4-H10. (a) O1-C6-O6-C7. (b) O7-C7-O6-C6. (c) C2-C1-O2-H8. (d) C11-C12-O11-H22. (e) C2-C3-O3-H9. (f) C11-C10-O10-H21. (g) C3-C4-O4-H10. (h) C10-C9-O9-H20. (i) C4-C5-O5-H11. (j) C9-C8-O8-H19. Abbreviations: "mtd" - well-tempered metadynamics, "no an." - system simulated without pre-heating, "an." - system simulated with pre-heating.

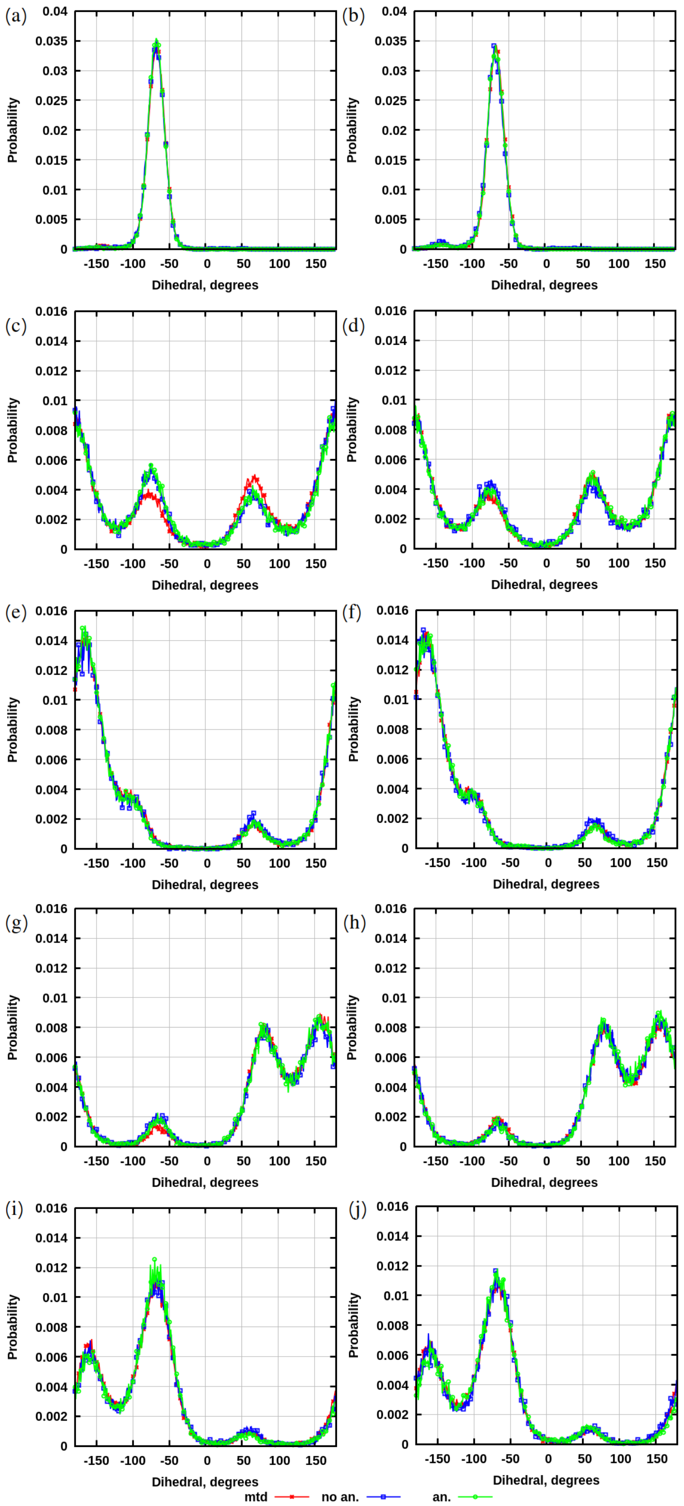

Figure S79 Distribution of dihedrals for simulations with single trehalose in water. Here the calculations were done for the group O5-H11. (a) O1-C6-O6-C7. (b) O7-C7-O6-C6. (c) C2-C1-O2-H8. (d) C11-C12-O11-H22. (e) C2-C3-O3-H9. (f) C11-C10-O10-H21. (g) C3-C4-O4-H10. (h) C10-C9-O9-H20. (i) C4-C5-O5-H11. (j) C9-C8-O8-H19. Abbreviations: "mtd" - well-tempered metadynamics, "no an." - system simulated without pre-heating, "an." - system simulated with pre-heating.

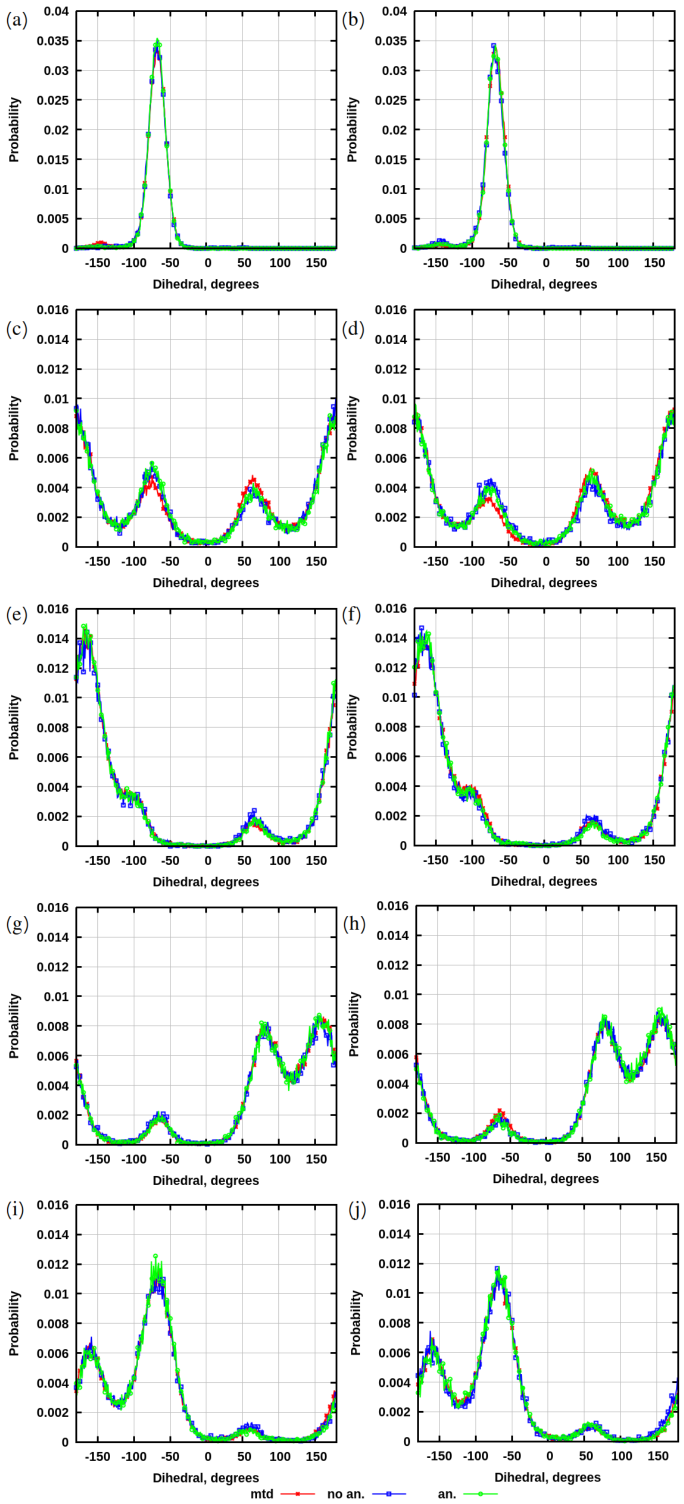

Figure S80 Distribution of dihedrals for simulations with single trehalose in water. Here the calculations were done for the group O6. (a) O1-C6-O6-C7. (b) O7-C7-O6-C6. (c) C2-C1-O2-H8. (d) C11-C12-O11-H22. (e) C2-C3-O3-H9. (f) C11-C10-O10-H21. (g) C3-C4-O4-H10. (h) C10-C9-O9-H20. (i) C4-C5-O5-H11. (j) C9-C8-O8-H19. Abbreviations: "mtd" - well-tempered metadynamics, "no an." - system simulated without pre-heating, "an." - system simulated with pre-heating.

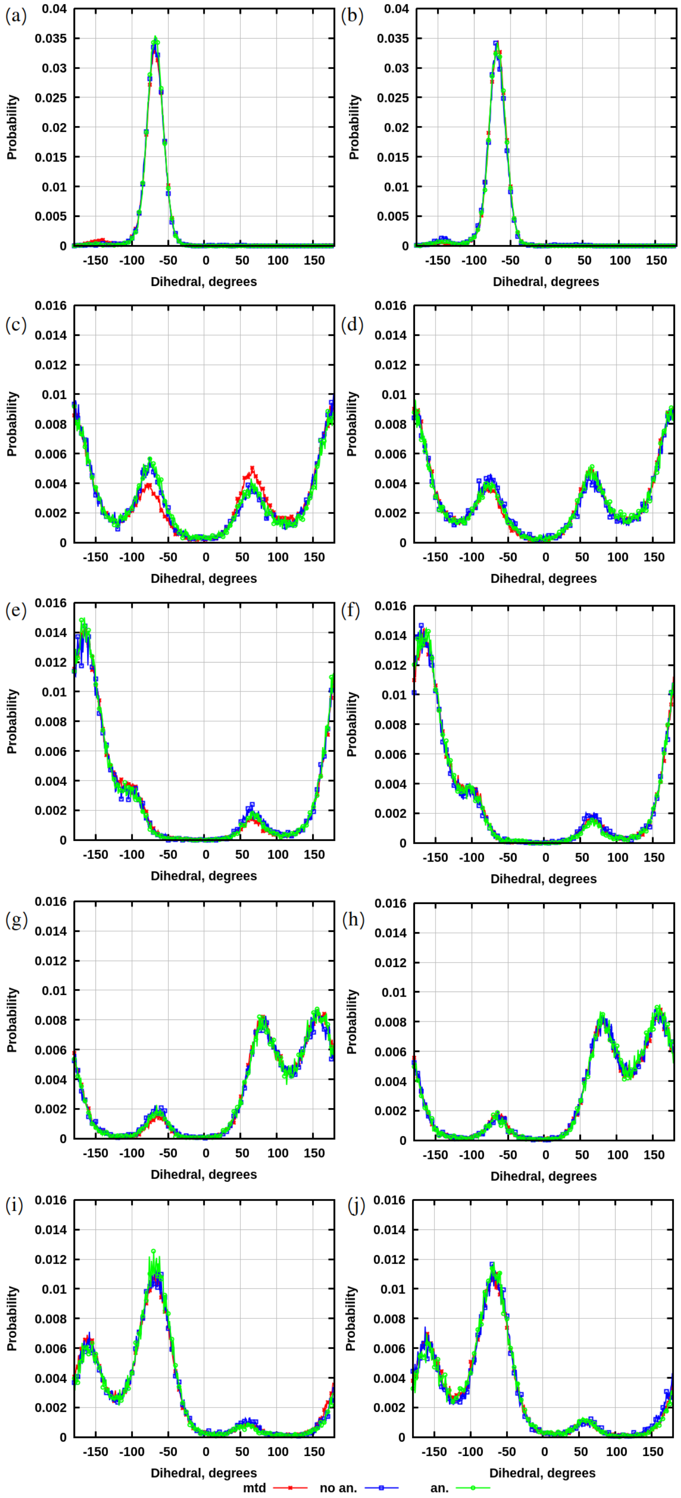

Figure S81 Distribution of dihedrals for simulations with single trehalose in water. Here the calculations were done for the group O7. (a) O1-C6-O6-C7. (b) O7-C7-O6-C6. (c) C2-C1-O2-H8. (d) C11-C12-O11-H22. (e) C2-C3-O3-H9. (f) C11-C10-O10-H21. (g) C3-C4-O4-H10. (h) C10-C9-O9-H20. (i) C4-C5-O5-H11. (j) C9-C8-O8-H19. Abbreviations: "mtd" - well-tempered metadynamics, "no an." - system simulated without pre-heating, "an." - system simulated with pre-heating.

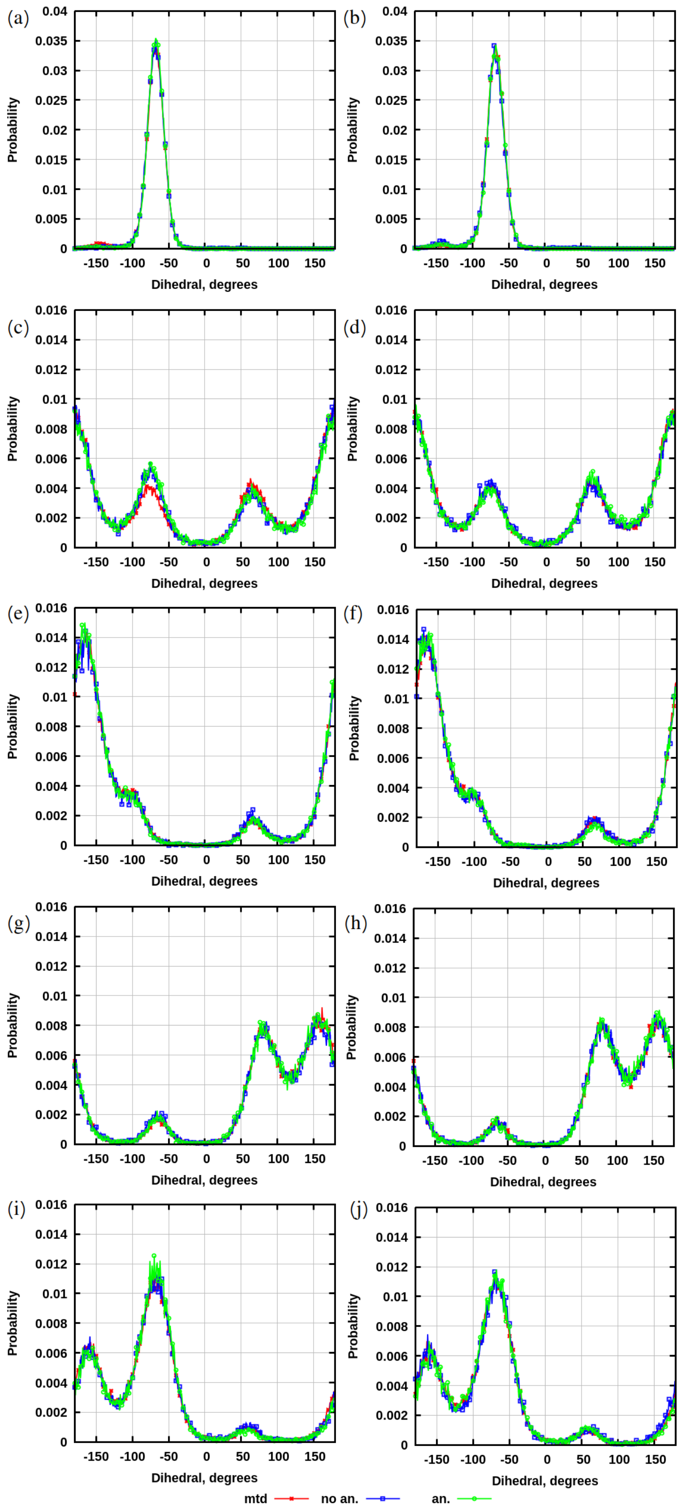

Figure S82 Distribution of dihedrals for simulations with single trehalose in water. Here the calculations were done for the group O8-H19. (a) O1-C6-O6-C7. (b) O7-C7-O6-C6. (c) C2-C1-O2-H8. (d) C11-C12-O11-H22. (e) C2-C3-O3-H9. (f) C11-C10-O10-H21. (g) C3-C4-O4-H10. (h) C10-C9-O9-H20. (i) C4-C5-O5-H11. (j) C9-C8-O8-H19. Abbreviations: "mtd" - well-tempered metadynamics, "no an." - system simulated without pre-heating, "an." - system simulated with pre-heating.

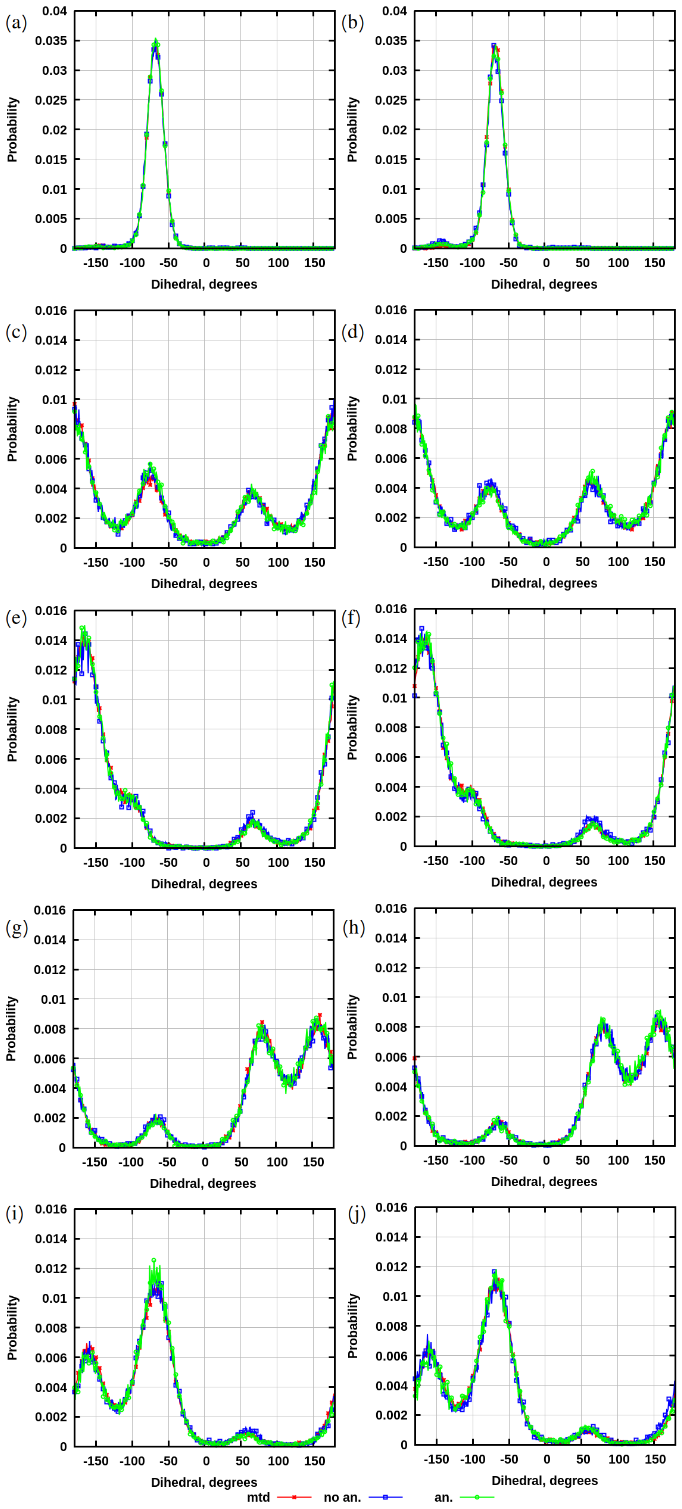

Figure S83 Distribution of dihedrals for simulations with single trehalose in water. Here the calculations were done for the group O9-H20. (a) O1-C6-O6-C7. (b) O7-C7-O6-C6. (c) C2-C1-O2-H8. (d) C11-C12-O11-H22. (e) C2-C3-O3-H9. (f) C11-C10-O10-H21. (g) C3-C4-O4-H10. (h) C10-C9-O9-H20. (i) C4-C5-O5-H11. (j) C9-C8-O8-H19. Abbreviations: "mtd" - well-tempered metadynamics, "no an." - system simulated without pre-heating, "an." - system simulated with pre-heating.

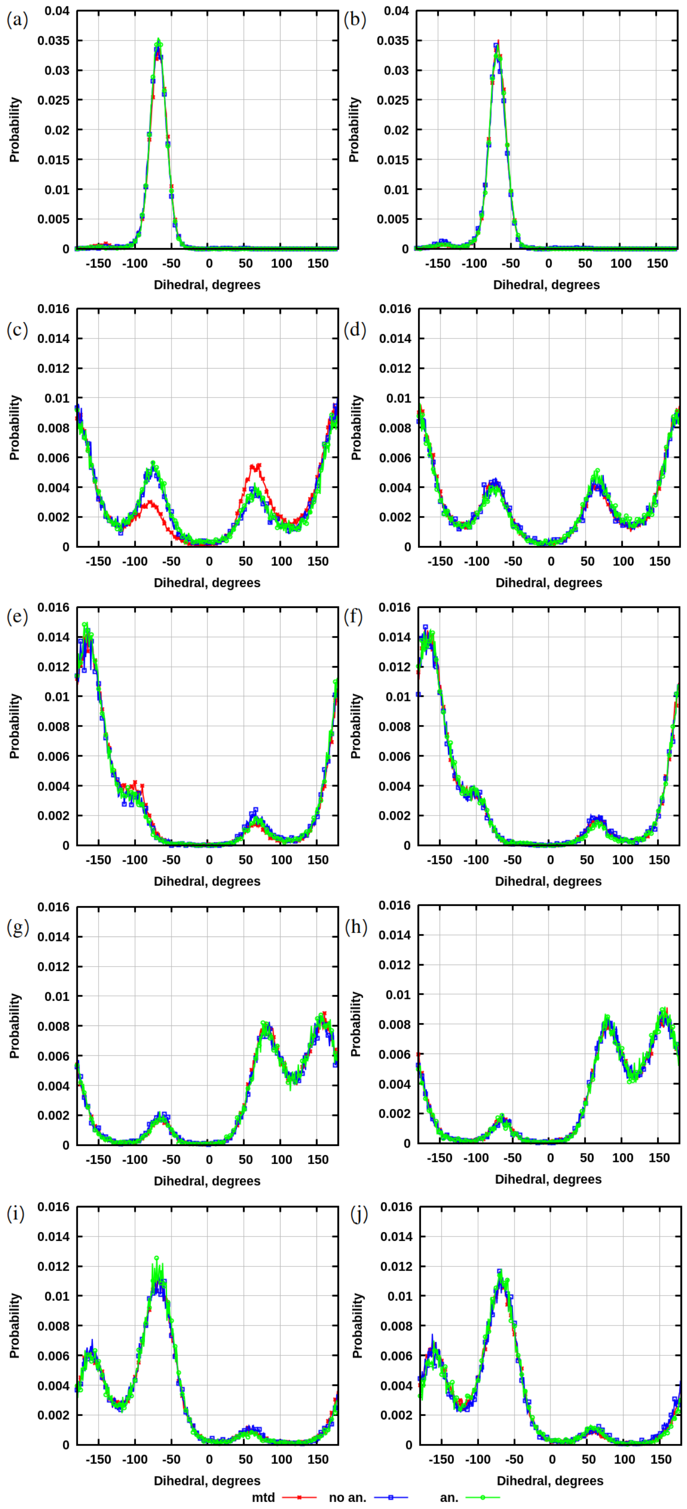

Figure S84 Distribution of dihedrals for simulations with single trehalose in water. Here the calculations were done for the group O10-H21. (a) O1-C6-O6-C7. (b) O7-C7-O6-C6. (c) C2-C1-O2-H8. (d) C11-C12-O11-H22. (e) C2-C3-O3-H9. (f) C11-C10-O10-H21. (g) C3-C4-O4-H10. (h) C10-C9-O9-H20. (i) C4-C5-O5-H11. (j) C9-C8-O8-H19. Abbreviations: "mtd" - well-tempered metadynamics, "no an." - system simulated without pre-heating, "an." - system simulated with pre-heating.

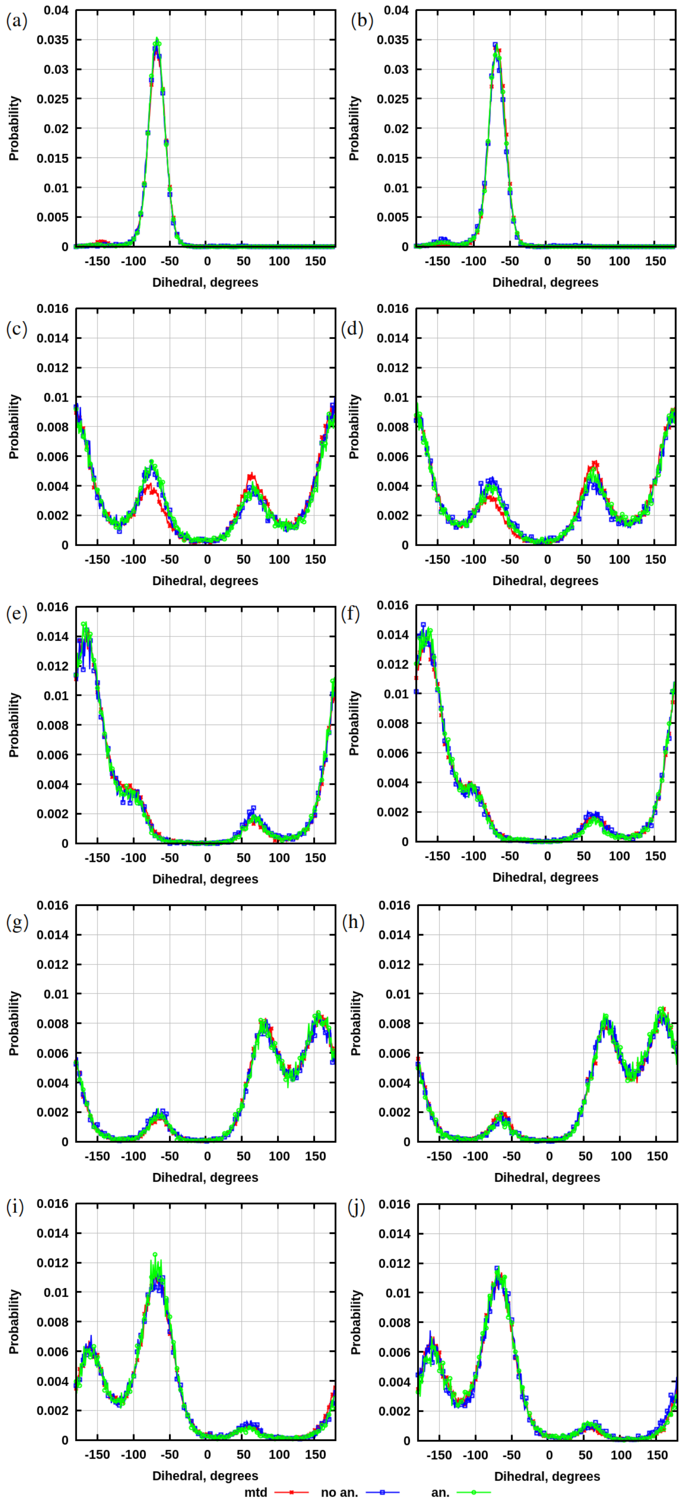

Figure S85 Distribution of dihedrals for simulations with single trehalose in water. Here the calculations were done for the group O11-H22. (a) O1-C6-O6-C7. (b) O7-C7-O6-C6. (c) C2-C1-O2-H8. (d) C11-C12-O11-H22. (e) C2-C3-O3-H9. (f) C11-C10-O10-H21. (g) C3-C4-O4-H10. (h) C10-C9-O9-H20. (i) C4-C5-O5-H11. (j) C9-C8-O8-H19. Abbreviations: "mtd" - well-tempered metadynamics, "no an." - system simulated without pre-heating, "an." - system simulated with pre-heating.

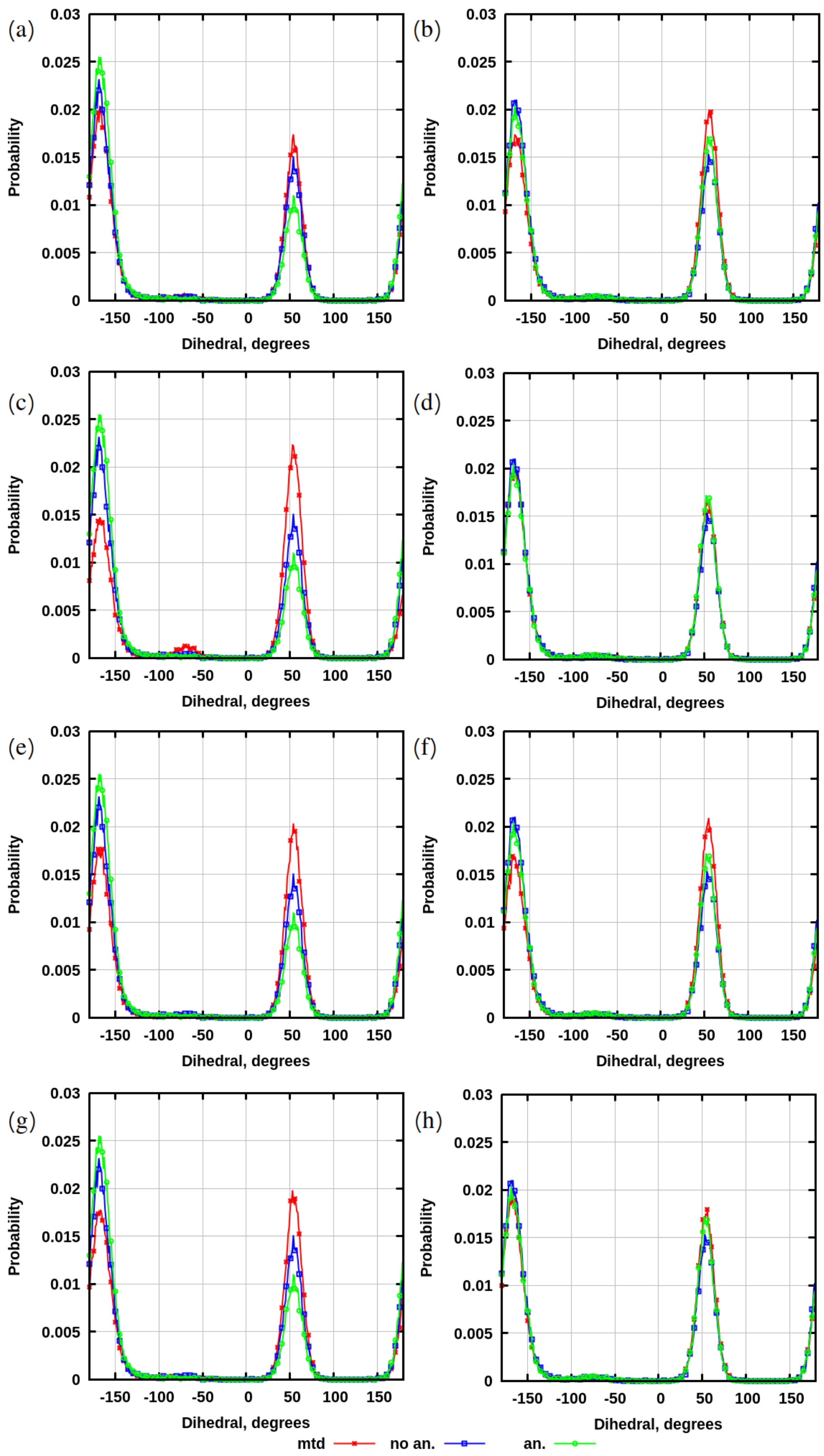

Figure S86 Distribution of dihedrals for simulations with single trehalose in water. (a) C3-C2-C1-O2, group O1. (b) C10-C11-C12-O11, group O1. (c) C3-C2-C1-O2, group O2-H8. (d) C10-C11-C12-O11, group O2-H8. (e) C3-C2-C1-O2, group O3-H9. (f) C10-C11-C12-O11, group O3-H9. (g) C3-C2-C1-O2, group O4-H10. (h) C10-C11-C12-O11, group O4-H10. Abbreviations: "mtd" - well-tempered metadynamics, "no an." - system simulated without pre-heating, "an." - system simulated with pre-heating.

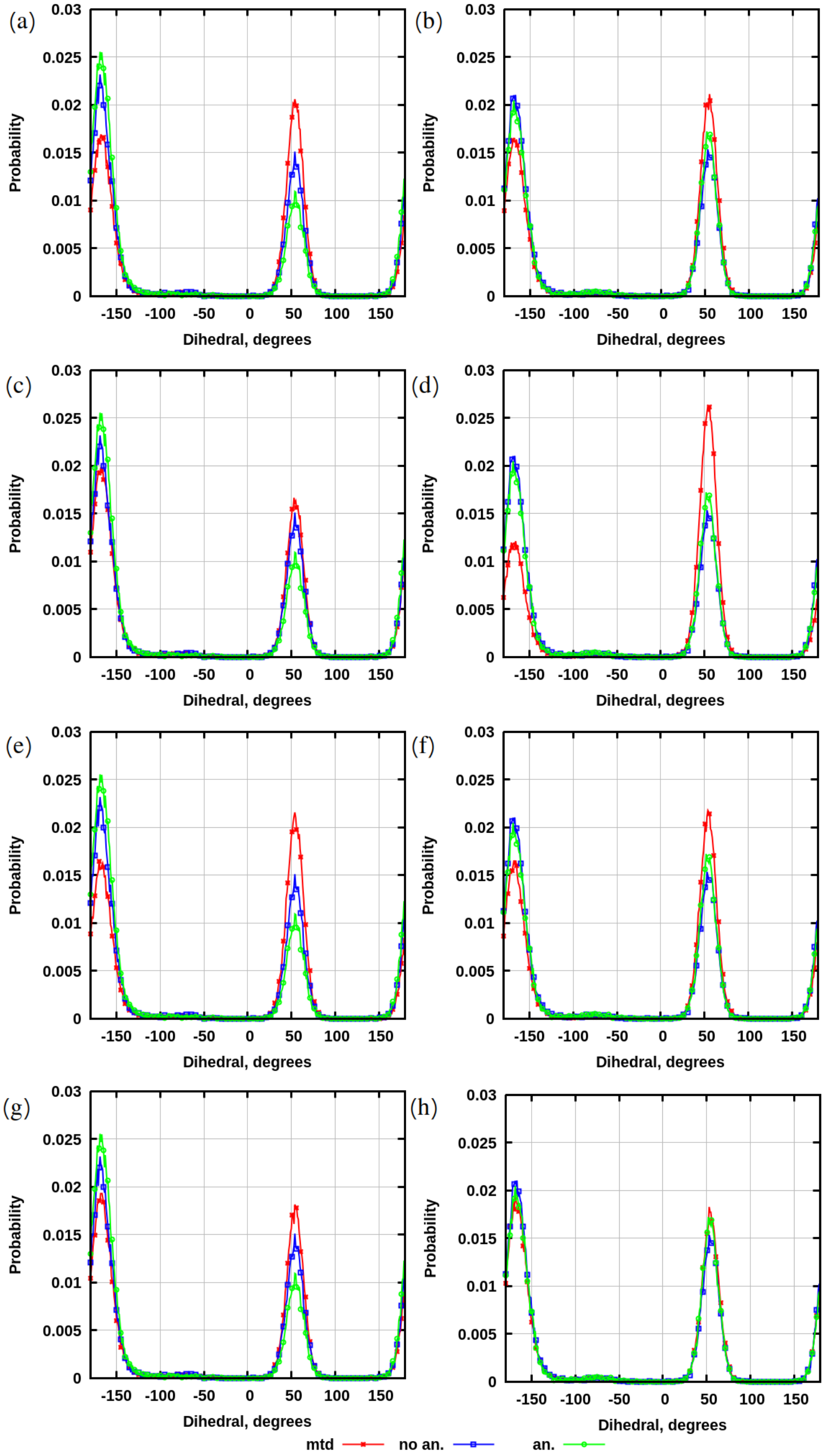

Figure S87 Distribution of dihedrals for simulations with single trehalose in water. (a) C3-C2-C1-O2, group O5-H11. (b) C10-C11-C12-O11, group O5-H11. (c) C3-C2-C1-O2, group O6. (d) C10-C11-C12-O11, group O6.(e) C3-C2-C1-O2, group O7. (f) C10-C11-C12-O11, group O7. (g) C3-C2-C1-O2, group O8-H19. (h) C10-C11-C12-O11, group O8-H19. Abbreviations: "mtd" - well-tempered metadynamics, "no an." - system simulated without pre-heating, "an." - system simulated with pre-heating.

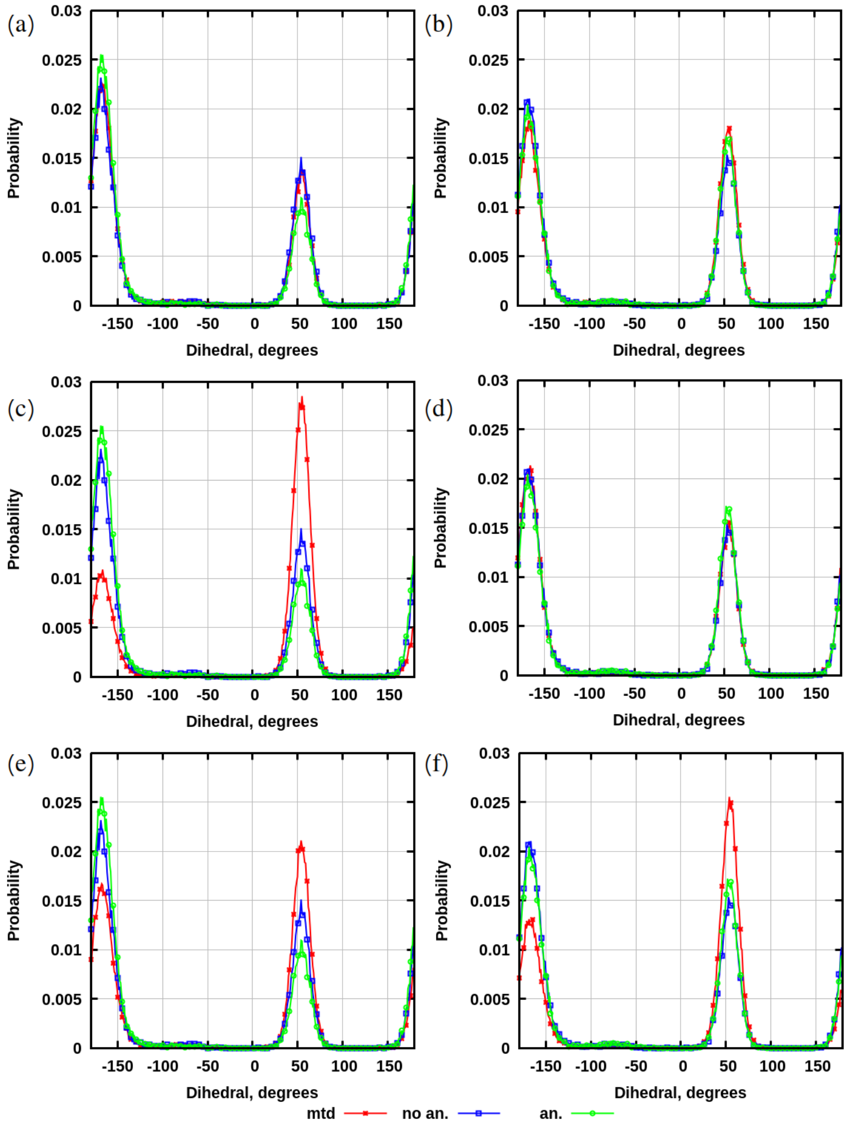

Figure S88 Distribution of dihedrals for simulations with single trehalose in water. (a) C3-C2-C1-O2, group O9-H20. (b) C10-C11-C12-O11, group O9-H20. (c) C3-C2-C1-O2, group O10-H21. (d) C10-C11-C12-O11, group O10-H21. (e) C3-C2-C1-O2, group O11-H22. (f) C10-C11-C12-O11, group O11-H22. Abbreviations: "mtd" - well-tempered metadynamics, "no an." - system simulated without pre-heating, "an." - system simulated with pre-heating.
